# Increase in Unemployment over the 2000’s: Comparison between People Living with HIV and the French General Population

**DOI:** 10.1371/journal.pone.0165634

**Published:** 2016-11-04

**Authors:** Margot Annequin, France Lert, Bruno Spire, Rosemary Dray-Spira, and the ANRS-Vespa2 Study Group

**Affiliations:** 1 Sorbonne Universités, UPMC Univ Paris 06, INSERM, Institut Pierre Louis d’Epidémiologie et de Santé Publique (IPLESP UMRS 1136), Department of Social Epidemiology, F75012, Paris, France; 2 INSERM, U1018, Center for Research in Epidemiology and Population Health, Villejuif, France; 3 INSERM, UMR912, Economics and Social Sciences Applied to Health & Analysis of Medical Information (SESSTIM), Marseille, France; 4 Aix Marseille University, UMR_S912, IRD, Marseille, France; 5 ORS PACA, Southeastern Health Regional Observatory, Marseille, France; University of Groningen, University Medical Center Groningen, NETHERLANDS

## Abstract

**Background:**

Despite improved health, unemployment has increased among people living with HIV (PlwHIV) over the last decade. However, since the economic recession of 2008, unemployment also increased in the French general population. This paper aimed to determine if the increase in the unemployment rate in the HIV population was higher than that in the French general population.

**Methods:**

We used data from the ANRS-Vespa study, a repeated cross-sectional survey among two national representative samples of PlwHIV followed at hospitals in France in 2003 and 2011. We compared employment and unemployment rates between HIV-infected people (overall and according to period of HIV diagnosis) and the French general population in 2003 and 2011, using multivariate Poisson regressions adjusted for individual sociodemographic characteristics.

**Results:**

The employment rate among PlwHIV was consistently lower than that in the general population in 2003 and 2011. In contrast, there was a trend of an increasing unemployment rate difference between PlwHIV and the general population: PlwHIV’s unemployment rate was 1.48 (95% confidence interval [CI]: 1.16–1.90) times higher than that of the general population in 2003, versus 1.62 (95% CI: 1.34–1.96) times higher in 2011. This unemployment rate difference was the highest for PlwHIV diagnosed in or after 2008 (adjusted prevalence rate ratio: 2.06; 95% CI: 1.59–2.67).

**Conclusions:**

These results suggest that in time of economic recession, an increasing proportion of PlwHIV may be excluded from the labor market although they are willing to re-enter it. This constitutes a major issue relative to social consequences of chronic disease.

## Introduction

Work and its absence are major determinants of individuals’ living condition by providing them–or lacking—with financial support, social status and identity[1]. Employment status and health are also intertwined processes[2]: working conditions and organization are major social determinant of health for those employed [3]; while unemployment has also been shown to cause a deterioration of health for those unemployed[4]. Furthermore, health-related selection operates at entry on the labor market for chronically ill individuals, marginalizing their participation in the labor market[5–7].

Regarding HIV-infection, studies conducted in various Western countries in the early 2000s provided evidence that, early in the combination antiretroviral therapy (cART) era, HIV infection negatively affected an individual’s chances of accessing, maintaining or returning to employment[8–12]. Disease severity and HIV-related discrimination were found to be the mechanisms at play in the negative effect of HIV-infection on employment [13], and this effect was shown to occur as early as the first few months following HIV diagnosis [14]. In addition, as in the general population, PlwHIV’s position in the labor market is also determined by sociodemographic characteristics of individuals such as age, sex, education or country of citizenship [11,15].

Major advances in treatment over the past decade have resulted in an increased life expectancy for HIV-positive individuals and a reduction in treatment side effects [16,17]. Initial treatments and their subsequent improvements have reframed HIV as a chronic disease, a ‘manageable’ disease [18]. In addition to PlwHIV’s aging, in the past decade, changes in the HIV epidemic in Europe have resulted in an increasing proportion of women and individuals originating from a country of Sub-Saharan Africa among the HIV population[19,20]. Notwithstanding these changes, HIV still predominantly affects a population which is mostly of working age.

Despite the improved health of PlwHIV, the unemployment rate has increased in the past decade in France among PlwHIV [21]. Furthermore, individuals diagnosed in the most recent years were shown to be at a higher risk for job loss and to have less of a chance of re-entering the labor market than those diagnosed in the early 2000s [21]. Nevertheless, it has not been determined whether this increase in unemployment is similar to that reported in the general population. Indeed France, like other European countries, faces an increase of unemployment since the economic crisis of 2008, so it is not clear if HIV-infection has worsened or moderated the impact of the economic crisis on the employment situation of HIV-infected individuals.

This paper aimed to determine if the increase in the unemployment rate in the French HIV population was higher than that in the French general population, overall and according to period of HIV-diagnosis.

## Materials and Methods

### Data sources

We used data from a large repeated cross-sectional study conducted in 2003 (n = 2 942) and 2011(n = 3 022). The ANRS-Vespa study [VIH, enquête sur les personnes atteintes—HIV, survey on people living with HIV] was a repeated cross-sectional survey conducted among two large, nationally representative samples of HIV-infected people followed at hospitals in France in 2003 and 2011, with the primary aim of assessing the various dimensions of the socioeconomic conditions and health of PlwHIV in France. Full details regarding the study design are available elsewhere[22,23].

Briefly, the surveys were conducted among random samples of hospital outpatients aged 18 or older who had a diagnosis of an HIV-infection for at least 6 months and who were either French citizens or immigrants having lived in France for at least 6 months. In both surveys, participants answered a standardized questionnaire in French, on socioeconomic status, living conditions, health status and healthcare use, which was administered face-to-face by a trained interviewer. A number of key questions were kept with the same wording between ANRS-Vespa1 and ANRS-Vespa2 surveys in order to conduct comparison. The ANRS-Vespa1 survey was conducted between December 2002 and September 2003 among 2932 PlwHIV recruited in 73 hospital departments in mainland France. The ANRS-Vespa2 survey was conducted between April 2011 and January 2012 among 3022 PlwHIV recruited in 73 hospital departments in mainland France, using a similar design. In both surveys and each participating hospital department, a sample of eligible patients (5080 in 2003 and 5617 in 2011), randomly selected according to the order of their appointment, were invited to participate by their physician[22]. In the ANRS-Vespa1 and ANRS-Vespa2 studies, after having being informed of the survey by their physician, eligible patients who agreed to participate signed a letter of informed consent in duplicate. Among eligible patients, 1767 in 2003 and 2217 in 2011 declined to participate. Both in 2003 and in 2011, non-participants were more likely than participants to have been HIV-infected through a way other than homo/bisexual contacts, to be employed at the time of the study and to have an impaired immunological status (CD4 cell count<500/mm^3^). In addition, in 2011 non-participants were more likely than participants to be non-French citizens. In both surveys data were weighted to account for the sampling design and participation bias. Details regarding the weighting procedure are provided elsewhere [22]. Both surveys received approval from the French Advisory Committee on Information Processing in Material Research in the Field of Health (CCTIRS) and met the ethical requirements of the French National Commission for Computing and Liberties (CNIL).

Information on the general population were obtained from the French Labor Survey, which is conducted each year by the National Institute of Statistics and Economic Studies (INSEE) among a national representative sample of individuals who live in private households[24]. Estimates are generalizable to residents in private households within the French metropolitan territory [25].For the present analyses, we used data form the 2003 (n = 281 541) and 2011 (n = 426 597) surveys.

### Measures

Employment status at the time of the interview was documented for all the participants, using similar questions in both surveys. In line with the International Labor Organization’s definition, employment status was categorized in two steps. First, participants were considered employed if they reported having a job (either paid or voluntary), regardless of their occupational status (self-employed or employed) and working hours (full time/part time), or if they had been on sick leave for less than 6 months. Next, the participants who were not employed were categorized as unemployed if they were available to work and reported having actively sought work within the previous 3 months. Otherwise, individuals not at work and not unemployed were categorized as inactive, including students, retirees, people on disability, and those with family responsibilities.

Data on sociodemographic characteristics, including age, sex, citizenship, educational level and household composition, were collected in both surveys. The following indicators were collected using the same sets of questions: age, which was categorized in three groups of approximately the same size according to the distribution of age of PlwHIV in 2011 (25–39, 40–49 and 50–64 years); citizenship, also categorized into three groups (French, sub-Saharan African countries and other) considering that foreign citizens from other regions that Sub-Saharan Africa account for a minority and heterogeneous group of PlwHIV in France; educational level, which was the highest attained level of education in Vespa1-2003 and the highest degree obtained in Vespa2-2011, and which was dichotomized as low (primary or secondary education in Vespa1-2003; high school diploma or lower in Vespa2-2011) or high (higher education in Vespa1-2003; tertiary degree in Vespa2-2011); and household composition, which was categorized into five groups (single with no children; single with children; cohabiting partner, no children; cohabiting partner with children; and other cohabiting adults).

Information pertaining to discrimination was available only in the Vespa2-2011 survey, which asked about workplace discrimination during the previous two years (yes/no) and discrimination in job-seeking (yes/no). Details on the definition of the discrimination variables are provided elsewhere[26].

### Statistical Analysis

The study population included HIV and non-HIV individuals of working age (25–64 years) at the time of the survey. Only HIV-individuals who had been diagnosed with HIV-infection in or after 1996, i.e., when cART was available, were included. Individuals with missing information for variables with < 1% missing values were excluded.

Differences in employment and unemployment rates between the general population and HIV-population were measured using adjusted prevalence rate ratios (aPRRs) comparing overall employment and unemployment rates between HIV-infected individuals and the general population in 2003 and 2011. Because our interest was to assess if the increase of unemployment rate in the HIV population was of same magnitude to that of the general population we ran different models to compare the probability of being employed (vs. unemployed and inactive) and unemployed (vs. employed or inactive) in the HIV population versus the general population. Prevalence rate ratio were computed with multivariate Poisson regression models with a robust variance estimator [27]. We estimated the parameters for 2003 and 2011 with Eq 1 below:

$$Y_{it}=\beta_{0}+\beta_{1}{HIV}_{it}+x\prime \beta_{2}X_{it}+\varepsilon_{it}$$ (eq 1)

The units of observation are individuals (i) in years (t) 2003 or 2011. Where *Y* is the outcome (employment or unemployment); *HIV* represents a variable that equals 0 for the general population and 1 for the HIV population; *X* represents a vector of control variables (age, sex, citizenship and household composition) and *ε* residual error.

In a second step to investigate changes over time and to determine whether the differences in unemployment and employment rates between the populations were different in 2003 and 2011, we estimated the previous model adding an interaction term with Eq 2.

$$Y_{i}=\beta_{0}+\beta_{1}{HIV}_{i}+\beta_{2}{year}_{i}+\beta_{3}{HIV}_{i}*{year}_{i}+x\prime \beta_{4}X_{i}+\varepsilon_{i}$$ (eq 2)

Where *Y* is the outcome, *HIV* represents a variable that takes 0 for general population and 1 for HIV population; *year* takes 0 for survey year 2003 and 1 for survey year 2011, and HIV*year is an interaction term between type of population (general population vs. HIV population) and survey years (2003 vs. 2011). *X* represents a vector of control variables (age, sex, citizenship and household composition) and *ε* residual error. The p-value of the interaction term determines if the magnitude of the difference of unemployment rate between the HIV population and general population is different in 2003 and 2011. Table 2 reports the aPRR for the variable HIV of Eq 1 for 2003 and 2011 and the aPRR of control variables are those from Eq 2.

To investigate differences according to the period of HIV diagnosis, PlwHIV included in the Vespa2-2011 study were categorized into three subgroups based on individuals’ date of HIV diagnosis as reported in their medical record: PlwHIV diagnosed in 1996–2003, i.e. during the early era of combined antiretroviral treatment; PlwHIV diagnosed in 2004–2007; and PlwHIV diagnosed in 2008–2011, i.e. during the first years of economic recession in France. In a model restricted to data collected in 2011, we compared the probability of being unemployed between each of these three subgroups of PlwHIV and the general population, controlling for sociodemographic characteristics. Wald tests were then used to compare the magnitude of the difference in unemployment rates between PlwHIV and the general population across the three subgroups of PlwHIV.

The choice of controlling variables was guided by the need to take into account major sociodemographic differences between survey populations and major determinants of employment situation. However, the choice was restricted in that they needed to be common to both the Vespa and the Labor Force Survey datasets. This resulted in models adjusted for sex, age, citizenship, educational level and household composition. Sex was introduced as a control variable because figures show a weaker participation of women in the labor market, due in part to their more recent incorporation into the French labor market. Young individuals are disadvantaged compared to older individuals due to inferior work experiences. Higher level of education increases chances of being employed due to higher qualifications. French citizens are also better integrated compared to non-nationals partly due to restriction according to citizenship on labor market and to discrimination; and single individuals are less integrated into the labor market, especially singles with children due to difficulties of conciliation between work and family lives.

Finally, sensitivity analyses were conducted to estimate the extent to which differences in the proportion and characteristics of African nationals between PlwHIV and the general population may explain our findings: 1) We adjusted the models for immigrant status rather than citizenships, as foreign citizenship can be a proxy of a more recent immigration; and 2) We restricted the analysis to French citizens.

All the analyses were performed using Stata 12® (Stata Corporation, College Station, TX) and accounted for the complex sampling design and the data weighting so that the estimates are representative of the entire population of HIV-infected individuals followed at hospitals in France.

## Results

### Characteristics of population

Three out of a total of 2 676 participants (1 010 participants in Vespa1-2003 and 1 666 participants in Vespa2-2011) who were of working age at the time of the interview and who had been diagnosed with HIV-infection in or after 1996 were excluded because of missing data on their educational level. The sample of the general population included 175 648 and 265 697 individuals in 2003 and 2011, aged between 25 and 64 years old.

Compared to the general population (Table 1), in 2003 and 2011, PlwHIV were more frequently men (67.9% and 62.2% versus 49.1% and 48.9%), were more often living alone (37.7% and 37.1% versus 12.8% and 15.6%) and more often had sub-Saharan citizenship (22.9% and 31.1% versus 0.7% and 0.9%). In 2003, they were younger than the general population (median age: 39 versus 43), and in 2011, they were of similar age (median age: 43 versus 45).

**Table 1:** Participants’ sociodemographic determinants of labor market position in 2003 and 2011 across people living with HIV and the French general population.

	2003			2011		
	PlwHIV	General Pop	*P*-value**	PlwHIV	General Pop	*P*-value**
	(N = 1 010)	(N = 175 648)		(N = 1 663)	(N = 265 697)	
	%*	%*		%*	%*	
**Sex**			<0.001			<0.001
Male	67.9	49.1		62.2	48.9	
Female	32.1	50.9		37.8	51.1	
**Educational level**			0.109			0.658
High	31.1	28.6		29.1	29.7	
Low	68.9	71.4		70.9	70.3	
**Country of citizenship**			<0.001			<0.001
Sub-Saharan Africa	22.9	0.7		31.1	0.9	
Other	9.9	5.8		6.5	6.2	
France	67.2	93.5		62.3	92.9	
**Age (years)**			<0.001			<0.001
25–39	52.4	39.6		35.0	36.2	
40–49	29.5	27.2		35.9	26.5	
50–64	18.1	33.1		29.1	37.3	
**Household composition**			<0.001			<0.001
Single with no children	37.7	12.8		37.1	15.6	
Single with children	9.6	6.9		11.7	7.6	
Cohabiting partner with children	16.3	50.8		17.2	47.1	
Cohabiting partner, no children	23.9	25.9		23.2	26.5	
Other cohabiting adults	12.4	3.5		10.9	3.1	

PlwHIV, People living with HIV; General Pop, French general population

* Weighted percentages

**χ^2^ test of comparison across people living with HIV and general population in 2003 and 2011

Among PlwHIV, over the last decade, men who have sex with men accounted for 37.2% and 35.6% of the study population in 2003 and 2011, respectively, while former or current injection drug users accounted for 5.5% and 4.1% respectively. The median age was 39 years in 2003 and 43 years in 2011. In 2003, the median time since diagnosis was 3.8 years, while in 2011, it was 7.4 years. The proportion of the participants being treated was 74.4% in 2003 and 87.3% in 2011.

Among the 2011 PlwHIV, those diagnosed in or after 2008 were younger (54.5% of those diagnosed between 2008 and 2011, 41.1% of those diagnosed between 2004 and 2007, and 22.4% of those diagnosed between 1996 and 2003 were 25 to 39 years old) and more often had sub-Saharan citizenship (respectively, 36.4% versus 36.0% and 25.9%)

### Employment and unemployment rates

**Fig 1:**
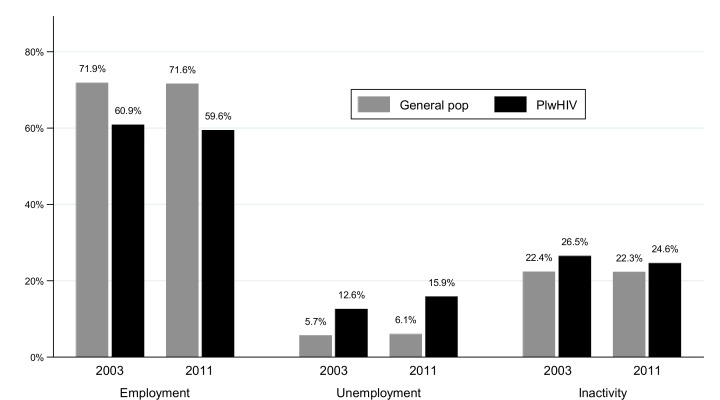
Employment, unemployment and inactivity rates in 2003 and 2011 among people living with HIV and the French general population. %, Weighted percentages; General Pop, French general population; PlwHIV, People living with HIV.

On the subject of employment (Fig 1), in 2003, 60.9% of PlwHIV were employed versus 71.9% of the general population (absolute difference: -11.0%, *p*-value: <0.001). In 2011, 59.5% of PlwHIV were employed versus 71.6% of the general population (absolute difference: -12.1%, *p*-value: <0.001). Details of employment rates according to sociodemographic characteristics are provided as supplementary information (Table A in S1 File).

With regards to unemployment, in 2003, 12.6% of PlwHIV were unemployed versus 5.7% of the general population (absolute difference: +6.9%, *p*-value: <0.001). In 2011, 15.9% of PlwHIV were unemployed versus 6.1% of the general population (absolute difference: +9.7%, *p*-value: <0.001). Among PlwHIV in 2011, those most recently diagnosed had a higher rate of unemployment than those diagnosed earlier (22.7% of those diagnosed in or after 2008, 16.4% of those diagnosed between 2004 and 2007, and 12.6% of those diagnosed between 1996 and 2003; *p* = 0.002). Furthermore unemployment rates were higher in women (20.9% vs. 12.8% in men), in individuals with a low level of education (17.1% vs. 13.0% in those highly educated), and in foreign citizens (26.7% in African nationals, 20.8% in foreign citizens from other regions, vs. 10.1% in French citizens). Unemployment rates also differed according to age, with higher rates among the youngest (22.8% for those aged between 25 and 39 years old; 15.6% among those aged between 40–49 years old and 8.2% among those aged between 50–64 years old). Singles with children were approximately one third (31.7%) unemployed versus 8.3% among those with a cohabiting partner and no children (Table B in S1 File).

Discrimination in job-seeking during the previous two years was reported by 28.7% of the PlwHIV.

### Changes over the 2000’s in difference in unemployment rates between HIV-infected individuals and the general population

After adjustment for sociodemographic determinants of labor market position (sex, age, country of citizenship, educational level and household composition), the employment rate among PlwHIV was lower than in the general population in 2003 and 2011 (2003 aPRR: 0.86, 95% confidence interval [95% CI]: 0.81–0.90; 2011 aPRR: 0.86 [95% CI]: 0.82–0.91). The magnitude of this difference remained stable between 2003 and 2011 (interaction term *p*-value: 0. 705).

With regard to unemployment (Table 2), after adjustment for sociodemographic determinants of labor market position, unemployment was significantly higher in PlwHIV compared to the general population both in 2003 (aPRR: 1.48 [95% CI]: 1.16–1.90) and 2011 (aPRR: 1.62 [95% CI]: 1.34–1.96). This difference tended to increase between 2003 and 2011, although this was not statistically significant (interaction term *p*-value: 0.080).

**Table 2:** Prevalence rate ratios for unemployment among people living with HIV versus the French general population in 2003 and 2011, adjusted for individual sociodemographic determinants of labor market position.

	Unemployment		
	aPRR		[95% CI]
**Survey**			
General population 2003	1		
PlwHIV in 2003	1.48**		[1.16,1.90]
General population 2011	1		
PlwHIV in 2011	1.62***		[1.34,1.96]
***Sociodemographic determinants of labor market position***			
**Sex**			
Male	1		
Female	0.94	[0.82,1.07]	
**Age (years)**			
25–39	1.26**	[1.09,1.46]	
40–49	1		
50–64	0.59***	[0.49,0.72]	
**Country of citizenship**			
France	1		
Sub-Saharan Africa	2.09***	[1.69,2.59]	
Other	1.68***	[1.37,2.06]	
**Educational level**			
High	1		
Low	1.15	[0.98,1.35]	
**Household composition**			
Single with no children	1.38**	[1.11,1.73]	
Single with children	2.09***	[1.65,2.65]	
Cohabiting partner with children	1.17	[0.96,1.43]	
Cohabiting partner, no children	1		
Other cohabiting adults	1.78***	[1.37,2.32]	

aPRR, Adjusted prevalence rate ratio; PlwHIV, People living with HIV; CI, Confidence interval

** p<0.01

*** p<0.001

Results remained unchanged after adjustment for immigrant status rather than citizenship (Table C in S1 File) and when the analysis was restricted to French nationals (Table D in S1 File), suggesting that differences in African nationals proportion and characteristics between PlwHIV and the general population are unlikely to explain our findings.

As for the sociodemographic determinants of unemployment, individuals with a higher rate of unemployment were those under 40 years of age (aPRR: 1.26 [95% CI]: 1.09–1.46), those with foreign citizenship (for sub-Saharan Africa citizenships, aPRR: 2.09 [95% CI]: 1.69–2.59; for other citizenships, aPRR: 1.68 [95% CI]: 1.37–2.06), and those living alone, with or without children (respectively, aPRR: 2.09 [95% CI]: 1.65–2.65, and aPRR: 1.38 [95% CI]: 1.11–1.73), and those living with other cohabiting adults (aPRR: 1.78 [95% CI]: 1.37–2.32).

**Fig 2:**
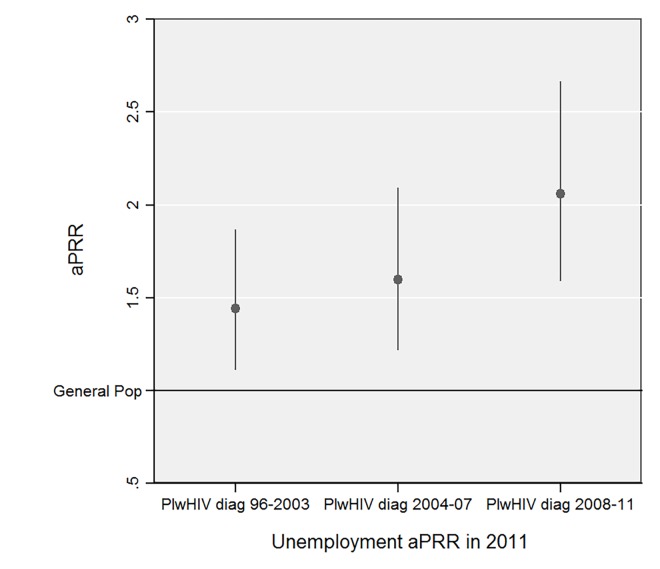
Prevalence rate ratios for unemployment among people living with HIV versus the French general population in 2011 according to the period of diagnosis, adjusted for individual sociodemographic determinants of labor market position. aPRR, prevalence rate ratio adjusted for sex, age, country of citizenship, educational level and household composition; PlwHIV diag 96–2003, People living with HIV diagnosed HIV-infected between 1996 and 2003; PlwHIV diag 2004–07, People living with HIV diagnosed HIV-infected between 2004 and 2007; PlwHIV diag 2008–11, People living with HIV diagnosed HIV-infected between 2008 and 2011; General pop., French general population

As shown in (Fig 2), among PlwHIV surveyed in 2011 although the unemployment rate was higher than that of the general population regardless of the period of diagnosis, the magnitude of this difference was not consistent across periods of diagnosis. Indeed, the aPRR comparing unemployment rates between PlwHIV and the general population was significantly higher for HIV-infected individuals diagnosed in or after 2008 compared to those diagnosed between 1996 and 2003 (aPRR: 2.06 [95% CI]: 1.59–2.67 versus 1.44 [95% CI]: 1.11–1.87, respectively; Wald test: *p = 0*.*02*). In contrast, the aPRR did not differ between HIV-infected individuals diagnosed between 2004 and 2007 and those diagnosed between 1996 and 2003 (aPRR: 1.60 [95% CI]: 1.22–2.09 versus 1.44 [95% CI]: 1.11–1.87, respectively; Wald test: *p = 0*.*52*).

## Discussion

Our results show that, overall, in France, the unemployment rate is still higher in PlwHIV compared to the general population, even after taking into account sociodemographic differences between the two populations. This unemployment gap has tended to increase over the past decade. Furthermore in 2011, this unemployment gap is more marked for those diagnosed in or after 2008, that is, at a time of worsening employment conditions in the labor market.

The Vespa surveys, conducted at two different points in time and nationally representative of HIV-infected hospital outpatients, constitute a unique dataset for studying PlwHIV’s living conditions and health. Furthermore detailed individual information on various indicators such as sociodemographic characteristics and employment situation made it possible to both account for i) changes within the HIV population over the last decade and ii) differences with the French general population. However some limitations should be noted. First, our results do not apply to the small proportion of HIV-infected individuals not followed at hospitals (less than 10% as estimated previously[28]). Nevertheless, HIV care is predominantly provided at hospitals and since 2006, annual medical checkups at hospital have been recommended by experts for PlwHIV [29]. Therefore, our results are likely to be generalizable to the vast majority of HIV-infected individuals being followed at hospitals in France. Secondly, due to the cross-sectional nature of our data, a causal interpretation of our results should be viewed with caution. Notably, we could not assess the extent to which the adverse employment situation of PlwHIV we report is related to HIV-infection itself or to other factors, e.g. discrimination or sector of employment. Third, our study was based on data collected in 2011. Since changes have occurred to the French labor market since then, notably a consistent increase in unemployment, our results may be slightly different in 2016. More specifically, it might be hypothesized that in the current context the unemployment gap between HIV-infected people and the general population may even be more marked than reported in our study. New studies conducted in the current context would be needed to properly answer this question.

### Weaker participation of HIV-individuals on the labor market over the last decade

Since disclosure of HIV-infection is not mandatory in the workplace, nor at job interviews, employment and unemployment rates of PlwHIV, should be similar to that of the French general population until complications due to the disease arise. However, our results show a weaker participation in the labor market of HIV-infected individuals. Our results are consistent with those of another recent study comparing HIV and non-HIV employment rates, in Denmark [30]. Similarly to our findings, they report that the employment rate in the HIV-infected population is still lower than in the general population.

This persistent and rising unemployment gap observed between PlwHIV and the French general population over the past decade is consistent with other studies that report low employment rates and high unemployment rates among individuals with various chronic conditions, including HIV[12,30,31], cancer[32], mental health problems[33], and limiting illnesses[34]. A major strength of our analysis is that it controls for differences in individual sociodemographic characteristics between the two populations. However, we were unable to adjust for prior periods of unemployment because such information was not available in both datasets.

To our knowledge, no study has compared the unemployment rate among PlwHIV with the background population. Most studies only focus on employment and disability or non-employment with no distinction between unemployment and inactivity. Because the rate of unemployment identifies the proportion of individuals willing to re-enter the labor market as they search for a job but are excluded from it, unemployment is a key indicator in the context of chronic disease affecting a working age population. This is especially true in the case of HIV, a disease that is less physically visible and less a health burden, but that continues to have a social impact.

However characterized by a high level of employment protection, French welfare protection may have limited the impact of the economic recession for those who were already employed, explaining this rising but moderate unemployment gap. Studies comparing the unemployment gap across countries have found that those characterized by a more regulated labor market with associated health and social policies, such as Sweden or Norway, were found to protect the employment of chronically ill individuals better in times of economic downturn than those with a deregulated labor market, such as the UK and Canada[35–37].

On the other hand, France is classified 23^rd^ out of 38 according to “the Migration policy Index Score” that measures the labor market mobility of immigrants[38]. This low ranking is related to the fact that 5.3 million jobs are “closed” to non-EU immigrants. Primarily in the public sector, but also in the private sector where foreign qualifications and experiences are rarely fully acknowledged or accepted for professional and licensed occupations. Thus, differences in economic integration of immigrants according to labor market policies arise. It appears, for example, that the UK more successfully integrates immigrants from sub-Saharan Africa than France, Italy or Spain where sub-Saharan immigrants predominately work in low-skilled jobs [39]. In addition, according to a recent study on changes over the last decades in the French labor market, the labor market has become more segmented between a stable and an unstable part [40]. The labor turnover is now concentrated on young adults and on few occupations. While in the past, fixed-term contracts used to be a stepping stone towards more stable contracts, they now appear as a trap for those who work under them. This dual labor market may then affect PlwHIV as the general population. Some individuals, especially immigrants originating from Sub-Saharan Africa and young PlwHIV, seem to be trapped within the unstable part of the labor market.

### Worsening situation for individuals diagnosed during economic recession

Although there is a high level of employment protection in France, we found that the unemployment gap has worsened for individuals diagnosed in or after 2008, that is, during a period of economic recession. This higher gap is in line with previous studies showing that the postdiagnosis years are critical in terms of the person’s employment situation[14,21,30]. Contrary to people with other chronic diseases, where a longer duration of illness is associated with a higher risk of work exits[41], HIV-individuals are at higher risk for being unemployed at the beginning of their illness, even though physical limitations due to HIV infection are likely to still be limited, with the exception of individuals who have already reached an advanced stage of HIV disease at the time of diagnosis.

The increased risk of unemployment among HIV individuals in recent years could therefore be related to various mechanisms. First, those recently diagnosed have faced an unfavorable economic context leading to the exclusion of chronically-ill individuals from the labor market. Studies have assessed the worsening employment gap between ill and non-ill individuals, in recent periods [33,34,42,43]. In time of economic recession, it has been showed that employment protection of disabled and chronically-ill individuals are less applied by companies[44]. This rising unemployment gap suggests that the increase in unemployment among PlwHIV is partly due to the general increase in the unemployment rate in France. Secondly, it can be seen from their work conditions that PlwHIV are more often employed under temporary contracts, which puts them at greater risk for job loss. In 2011, 15.8% of PlwHIV and 18.4% of those diagnosed in or after 2008 were employed under interim or fixed-term contracts versus 9.7% in the general population. Third, our results indicate that the ‘biographical disruption’ due to being diagnosed with a chronic illness theorized in the sociological literature, which describes how the structure of everyday life is disturbed by the diagnosis[45], may be exacerbated by the socioeconomic context. However due to the nature of our data, we were unable to properly disentangle the ‘great recession effect’ from the ‘biographical disruption’ effect. Finally, discrimination and stigmatization are still probably barriers to returning to work and to stay in the workplace. In 2011, in the Vespa-2 study almost one third of unemployed PlwHIV reported discrimination in job-seeking. However, major reasons of discrimination cited by respondents when applying for a job were origin or nationality and skin color [26]. If HIV-infection seems to be less a physical burden in everyday life for infected individuals, social and contextual factors continue to shape living conditions of PlwHIV, among whom a substantial and increasing proportion are individuals already socially disadvantaged on the labor market.

Further studies with a longitudinal design and detailed data both on HIV-infected patients’ health status and living conditions, and comparisons across countries are needed to fully understand the possible mechanisms at the individual and macro levels underlying the situation. Comparison with other chronic conditions would also be interesting to help better disentangle the numerous mechanisms at play in the social consequences of chronic disease.

### Conclusions

In conclusion, our study indicates that, the unemployment rate among PlwHIV is still higher than in the general population, a difference which tended to increase over the past decade. Because HIV-infection affects a large population of adults of prime working age, our findings have public health and social implications and contribute to the discussion of how chronically ill individuals are integrated into the labor market. Unemployment among chronically-ill individuals should be studied further, as this identifies ill individuals who are willing to re-enter the labor market but are excluded from it. Identifying protective factors from the risk of unemployment at the individual and macro level, may contribute to diminishing the social consequences of chronic disease.

## Appendix

**Members of the ANRS-VESPA2 Study Group are:** France Lert (INSERM UMR-S 1018, France.Lert@inserm.fr) and Bruno Spire (INSERM UMR-S 912/ORS PACA), scientific coordinators; Patrizia Carrieri (INSERM UMR-S 912/ORS PACA), Rosemary Dray-Spira (INSERM UMR-S 1136), Christine Hamelin (University Versailles Saint-Quentin-en-Yvelines), Nicolas Lorente (INSERM UMR-S 912/ORS PACA), Marie Préau (INSERM UMR-S 912/ORS PACA), Marie Suzan-Monti (INSERM UMR-S 912/ORS PACA); with the collaboration of Marion Mora (INSERM UMR-S 912/ORS PACA).

**Participating hospitals and investigators:** Aix-en-Provence, CH Pays d’Aix (T. Allègre, P. Mours, J.M. Riou, M. Sordage); Angers, CHU Hôtel-Dieu (J.M. Chennebault, P. Fialaire, V. Rabier); Annemasse, CH Alpes-Léman (M. Froidure, D. Huguet, D. Leduc); Avignon, Hôpital Henri Duffaut (G. P ichancourt, A. Wajsbrot); Besançon, Hôpital Saint-Jacques (C. Bourdeaux, A. Foltzer, B. Hoen, L. Hustache-Mathieu); Bobigny, Hôpital Avicenne (S. Abgrall, R. Barruet, O. Bouchaud, A. Chabrol, S. Mattioni, F. Mechai); Bondy, Hôpital Jean Verdier (V. Jeantils); Bordeaux, Hôpital Saint-André (N. Bernard, F. Bonnet, M. Hessamfar, D. Lacoste, D. Malvy, P. Mercié, P. Morlat, F. Paccalin, M.C. Pertusa, T. Pistone, M.C. Receveur, M.A. Vandenhende); Boulogne-Billancourt, Hôpital Ambroise Paré (C. Dupont, A. Freire Maresca, J. Leporrier, E. Rouveix); Caen, Hôpital Clémenceau (S. Dargere, A. de la Blanchardière, A. Martin, V. Noyon, R. Verdon); CH de Chambéry (O. Rogeaux); Clermont-Ferrand, CHU Gabriel Montpied (J. Beytout, F. Gourdon, H. Laurichesse); Colombes, Hôpital Louis-Mourier (F. Meier, E. Mortier, A.M. Simonpoli); Creil, CH Laennec (F. Cordier); Créteil, CHIC (I. Delacroix, V. Garrait, B. Elharrar), Hôpital Henri Mondor (S. Dominguez, A.S. Lascaux, J.D. Lelièvre, Y. Levy, G. Melica); Dijon, Hôpital du Bocage (M. Buisson, L. Piroth, A. Waldner); Eaubonne, Hôpital Simone Veil (N. Gruat, A. Leprêtre); Garches, Hôpital Raymond-Poincaré (P. de Truchis, D. Le Du, J.Cl. Melchior); CH de Gonesse (R. Sehouane, D. Troisvallets); CHU de Grenoble (M. Blanc, I. Boccon-Gibod, A. Bosseray, J.P. Brion, F. Durand, P. Leclercq, F. Marion, P. Pavese); La Rochelle, Hôpital Saint- Louis (E. Brottier-Mancini, L. Faba, M. Roncato-Saberan); La Roche-sur-Yon, CHD Les Oudairies (O. Bollengier-Stragier, J.L. Esnault, S. Leautez-Nainville, P. P erré); CH de Lagny Marne-la-Vallée (E. Froguel, M. Nguessan, P. Simon); Le Chesnay, CH de Versailles (P. Colardelle, J. Doll, C. Godin-Collet, S. Roussin-Bretagne); Le Kremlin-Bicêtre, Hôpital de Bicêtre (J.F. Delfraissy, M. Duracinsky, C. Goujard, D. Peretti, Y. Quertainmont); CH du Mans (J. Marionneau); Lens, CH Dr. Schaffner (E. Aissi, N. Van Grunderbeeck); Limoges, CHU Dupuytren (E. Denes, S. Ducroix-Roubertou, C. Genet, P. Weinbreck); Lyon, Hôpital de la Croix-Rousse (C. Augustin-Normand, A. Boibieux, L. Cotte, T. Ferry, J. Koffi, P. Miailhes, T. Perpoint, D. Peyramond, I. Schlienger); Hôpital Édouard-Herriot (J.M. Brunel, E. Carbonnel, P. Chiarello, J.M. Livrozet, D. Makhloufi); Marseille, Hôpital de la Conception (C. Dhiver, H. Husson, A. Madrid, I. Ravaux, M.L. de Severac, M. Thierry Mieg, C. Tomei), Hôpital Nord (S. Hakoun, J. Moreau, S. Mokhtari, M.J. Soavi), Hôpital Sainte Marguerite (O. Faucher, A. Ménard, M. Orticoni, I. Poizot-Martin, M.J. Soavi); Montpellier, Hôpital Gui de Chauliac (N. Atoui, V. Baillat, V. Faucherre, C. Favier, J.M. Jacquet, V. Le Moing, A. Makinson, R. Mansouri, C. Merle); Montivilliers, Hôpital Jacques Monod (N. Elforzli); Nantes, Hôtel-Dieu (C. Allavena, O. Aubry, M. Besnier, E. Billaud, B. Bonnet, S. Bouchez, D. Boutoille, C. Brunet, N. Feuillebois, M. Lefebvre, P. Morineau-Le Houssine, O. Mounoury, P. Point, F. Raffi, V. Reliquet, J.P. Talarmin); Nice, Hôpital l’Archet (C. Ceppi, E. Cua, P. Dellamonica, F. De Salvador-Guillouet, J. Durant, S. Ferrando, V. Mondain-Miton, I. Perbost, S. Pillet, B. Prouvost-Keller, C. Pradier, P. Pugliese, V. Rahelinirina, P.M. Roger, E. Rosenthal, F. Sanderson); Orléans, Hôpital de La Source (L. Hocqueloux, M. Niang, T. Prazuck), Hôpital Porte Madeleine (P. Arsac, M.F. Barrault-Anstett); Paris, Hôpital Bichat—Claude-Bernard (M. Ahouanto, E. Bouvet, G. Castanedo, C. Charlois-Ou, A. Dia Kotuba, Z. Eid-Antoun, C. Jestin, K. Jidar, V. Joly, M.A. Khuong-Josses, N. Landgraf, R. Landman, S. Lariven, A. Leprêtre, F. L’hériteau, M. Machado, S. Matheron, F. Michard, G. Morau, G. Pahlavan, B.C. Phung, M.H. Prévot, C. Rioux, P. Yéni), Hôpital Cochin-Tarnier (F. Bani-Sadr, A. Calboreanu, E. Chakvetadze, D. Salmon, B. Silbermann), Hôpital européen Georges-Pompidou (D. Batisse, M. Beumont, M. Buisson, P. Castiel, J. Derouineau, M. Eliaszewicz, G. Gonzalez, D. Jayle, M. Karmochkine, P. Kousignian, J. Pavie, I. Pierre, L. Weiss), Hôpital Lariboisière (E. Badsi, M. Bendenoun, J. Cervoni, M. Diemer, A. Durel, A. Rami, P. Sellier), Hôpital Pitié-Salpêtrière (H. Ait-Mohand, N. Amirat, M. Bonmarchand, F. Bourdillon, G. Breton, F. Caby, J.P. Grivois, C. Katlama, M. Kirstetter, L. Paris, F. Pichon, L. Roudière, L. Schneider, M.C. Samba, S. Seang, A. Simon, H. Stitou, R. Tubiana, M.A. Valantin), Hôpital Saint-Antoine (D. Bollens, J. Bottero, E. Bui, P. Campa, L. Fonquernie, S. Fournier, P.M. Girard, A. Goetschel, H.F. Guyon, K. Lacombe, F. Lallemand, B. Lefebvre, J.L. Maynard, M.C. Meyohas, Z. Ouazene, J. Pacanowski, O. Picard, G. Raguin, P. Roussard, M. Tourneur, J. Tredup, N. Valin); Hôpital Saint-Louis (S. Balkan, F. Clavel, N. Colin de Verdière, N. De Castro, V. de Lastours, S. Ferret, S. Gallien, V. Garrait, L. Gérard, J. Goguel, M. Lafaurie, C. Lascoux-Combe, J.M. Molina, E. Oksenhendler, J. Pavie, C. Pintado, D. Ponscarme, W. Rozenbaum, A. Scemla), Hôpital Tenon (P. Bonnard, L. Lassel, M.G. Lebrette, T. Lyavanc, P. Mariot, R. Missonnier, M. Ohayon, G. Pialoux, M.P. Treilhou, J.P. Vincensini); Hôtel-Dieu (J. Gilquin, B. Hadacek, L. Nait-Ighil, T.H. Nguyen, C. Pintado, A. Sobel, J.P. Viard, O. Zak Dit Zbar); Perpignan, Hôpital Saint-Jean (H. Aumaître, A. Eden, M. Ferreyra, F. Lopez, M. Medus, S. Neuville, M. Saada); Pontoise, CH René Dubos (L. Blum); Quimper, Hôpital Laennec (P. Perfezou); Rennes, Hôpital de Pontchaillou (C. Arvieux, J.M. Chapplain, M. Revest, F. Souala, P. Tattevin); Rouen, Hôpital Charles-Nicolle (S. Bord, F. Borsa-Lebas, F. Caron, C. Chapuzet, Y. Debab, I. Gueit, M. Etienne, C. Fartoukh, K. Feltgen, C. Joly, S. Robaday-Voisin, P. Suel); Saint-Denis, CH Delafontaine (M.A. Khuong, J. Krausse, M. Poupard, G. Tran Van); Saint-Étienne, CHU Nord (C. Cazorla, F. Daoud, P. Fascia, A. Frésard, C. Guglielminotti, F. Lucht); Strasbourg, Nouvel hôpital civil (C. Bernard-Henry, C. Cheneau, J.M. Lang, E. de Mautort, M. P artisani, M. Priester, D. Rey); Suresnes, Hôpital Foch (C. Majerholc, D. Zucman); Toulon, CHI Chalucet (A. Assi, A. Lafeuillade), Hôpital Sainte-Anne (J.P. de Jaureguiberry, O. Gisserot); Toulouse, Hôpital de La Grave (C. Aquilina, F. Prevoteau du Clary), Hôpital Purpan (M. Alvarez, M. Chauveau, L. Cuzin, P. Delobel, D. Garipuy, E. Labau, B. Marchou, P. Massip, M. Mularczyk, M. Obadia); Tourcoing, CH Gustave Dron (F. Ajana, C. Allienne, V. Baclet, X. de la Tribonnière, T. Huleux, H. Melliez, A. Meybeck, B. Riff, M. Valette, N. Viget); Tours, CHRU Bretonneau (F. Bastides, L. Bernard, G. Gras, P. Guadagnin); Vandoeuvre-lès-Nancy, CHU Brabois (T. May, C. Rabaud); Vannes, CH Bretagne Atlantique (A. Dos Santos, Y. P oinsignon); Villejuif, Hôpital Paul-Brousse, (O. Derradji, L. Escaut, E. Teicher, D. Vittecoq); CHI de Villeneuve-Saint-Georges, (J. Bantsima, P. Caraux-Paz, O. Patey).

## Supporting Information

S1 File Supplementary Tables. (DOCX)

S2 File Translation of survey questions used to define the employment situation from the ANRS-Vespa1 and ANRS-Vespa2 questionnaires. (DOCX)

S3 File Full ANRS-Vespa1 questionnaire (in French). (PDF)

S4 File Full ANRS-Vespa2 questionnaire (in French). (PDF)

## References

[1] Berkman LF. Commentary: The hidden and not so hidden benefits of work: identity, income and interaction. Int J Epidemiol. 2014;43: 1517–1519. 10.1093/ije/dyu110 24942143 PMC4190516

[2] Bartley M. Unemployment and ill health: understanding the relationship. J Epidemiol Community Health. 1994;48: 333–337. 10.1136/jech.48.4.333 7964329 PMC1059979

[3] Bartley M, Sacker A, Clarke P. Employment status, employment conditions, and limiting illness: prospective evidence from the British household panel survey 1991–2001. J Epidemiol Community Heal. 2004;58: 501–506. 10.1136/jech.2003.009878 PMC1732781 15143119

[4] Roelfs DJ, Shor E, Davidson KW, Schwartz JE. Losing life and livelihood: a systematic review and meta-analysis of unemployment and all-cause mortality. Soc Sci Med. 2011/2/19 2011;72: 840–854. 10.1016/j.socscimed.2011.01.005 21330027 PMC3070776

[5] Duguet E, le Clainche C. The Effect of Non-Work Related Health Events on Career Outcomes: An Evaluation in the French Labor Market. Rev d’Economie Polit. 2014;124: 437–465.

[6] Virtanen P, Janlert U, Hammarström a. Health status and health behaviour as predictors of the occurrence of unemployment and prolonged unemployment. Public Health. 2013;127: 46–52. 10.1016/j.puhe.2012.10.016 23158056

[7] Schuring M, Robroek SJW, Otten FWJ, Arts CH, Burdorf A. The effect of ill health and socioeconomic status on labor force exit and re-employment: A prospective study with ten years follow-up in the Netherlands. Scand J Work Environ Heal. 2013;39: 134–143. 10.5271/sjweh.3321 22961587

[8] Dray-Spira R, Lert F, Marimoutou C, Bouhnik AD, Obadia Y. Socio-economic conditions, health status and employment among persons living with HIV/AIDS in France in 2001. AIDS Care. 2003;15: 739–748. 10.1080/09540120310001618595 14617496

[9] Goldman DP, Bao Y. Effective HIV treatment and the employment of HIV(+) adults. Heal Serv Res. 2004;39: 1691–1712. 10.1111/j.1475-6773.2004.00313.x 15533182 PMC1361093

[10] Rabkin JG, McElhiney M, Ferrando SJ, Van Gorp W, Lin SH. Predictors of employment of men with HIV/AIDS: a longitudinal study. Psychosom Med. 2004;66: 72–78. 10.1097/01.PSY.0000108083.43147.6D 14747640

[11] Dray-Spira R, Lert F, The ANRS-Vespa study group. Living and working with HIV in France in 2003: results from the ANRS-EN12-VESPA Study. AIDS. 2007;21(Suppl1): S29–36. 10.1097/01.aids.0000255082.31728.52 17159584

[12] Dray-Spira R, Gueguen A, Ravaud JF, Lert F. Socioeconomic differences in the impact of HIV infection on workforce participation in France in the era of highly active antiretroviral therapy. Am J Public Health. 2007;97: 552–558. 10.2105/AJPH.2005.081083 17267720 PMC1805026

[13] Dray-Spira R, Gueguen A, Lert F, The ANRS-Vespa study group. Disease severity, self-reported experience of workplace discrimination and employment loss during the course of chronic HIV disease: differences according to gender and education. Occup Env Med. 2008;65: 112–119. 10.1136/oem.2007.034363 17981911 PMC2259228

[14] Dray-Spira R, Persoz A, Boufassa F, Gueguen A, Lert F, Allegre T, et al Employment loss following HIV infection in the era of highly active antiretroviral therapies. Eur J Public Heal. 2006;16: 89–95. 10.1093/eurpub/cki153 16126745

[15] Dray-Spira R, Legeai C, Le Den M, Boue F, Lascoux-Combe C, Simon A, et al Burden of HIV disease and comorbidities on the chances of maintaining employment in the era of sustained combined antiretroviral therapies use. AIDS. 2012;26: 207–215. 10.1097/QAD.0b013e32834dcf61 22008658 PMC3680952

[16] Morlat P, Roussillon C, Henard S, Salmon D, Bonnet F, Cacoub P, et al Causes of death among HIV-infected patients in France in 2010 (national survey): trends since 2000. AIDS. 2014/6/06 2014;28: 1181–1191. 10.1097/qad.0000000000000222 24901259

[17] Hasse B, Ledergerber B, Furrer H, Battegay M, Hirschel B, Cavassini M, et al Morbidity and aging in HIV-infected persons: the Swiss HIV cohort study. Clin Infect Dis. 2011;53: 1130–1139. 10.1093/cid/cir626 21998280

[18] Deeks SG, Lewin SR, Havlir D V. The end of AIDS: HIV infection as a chronic disease. Lancet. 2013;382: 1525–1533. 10.1016/S0140-6736(13)61809-7 24152939 PMC4058441

[19] Del Amo J, Likatavicius G, Perez-Cachafeiro S, Hernando V, Gonzalez C, Jarrin I, et al The epidemiology of HIV and AIDS reports in migrants in the 27 European Union countries, Norway and Iceland: 1999–2006. Eur J Public Heal. 2011;21: 620–626. 10.1093/eurpub/ckq150 21051469

[20] Hamers FF, Downs AM. The changing face of the HIV epidemic in western Europe: what are the implications for public health policies? Lancet. 2004;364: 83–94. 10.1016/s0140-6736(04)16594-x 15234861

[21] Annequin M, Lert F, Spire B, Dray-Spira R, The ANRS-Vespa2 study group. Has the employment status of people living with HIV changed since the early 2000s? AIDS. 2015;29: 1537–47. 10.1097/QAD.0000000000000722 26244393

[22] Tron L, Lert F, Spire B, Dray-Spira R, The ANRS-Vespa2 study group. Tobacco Smoking in HIV-Infected versus General Population in France: Heterogeneity across the Various Groups of People Living with HIV. PLoS One. 2014;9: e107451 10.1371/journal.pone.0107451 25202968 PMC4159331

[23] Peretti-Watel P, Riandey B, Dray-Spira R, Bouhnik AD, Sitta R, Obadia Y, et al Surveying the HIV-Positive Population inf France. The ANRS-EN12-VESPA 2003 Survey. Population (Paris). 2005;60: 525–550. 10.3917/popu.504.0525

[24] INSEE. Emploi (en continu), version FPR -2011 -(2011) [fichier électronique], INSEE[producteur], Centre Maurice Halbwachs (CMH) [diffuseur]. 2011.

[25] INSEE. Continous labour Force Survey (as of 2003) [Internet]. 2016 [cited 1 Sep 2016]. Available: http://www.insee.fr/en/methodes/sources/pdf/methodologie_eeencontinu_anglais.pdf

[26] Marsicano E, Dray-Spira R, Lert F, Aubriere C, Spire B, Hamelin C, et al Multiple discriminations experienced by people living with HIV in France: results from the ANRS-Vespa2 study. AIDS Care. 2014;26(Suppl1): S97–106. 10.1080/09540121.2014.907385 24738926

[27] Barros A, Hirakata V. Alternatives for logistic regression in cross-sectional studies: an empirical comparison of models that directly estimate the prevalence ratio. BMC Med Res Methodol. 2003;3: 21 10.1186/1471-2288-3-21 14567763 PMC521200

[28] Nadal J-M, Bourdillon F, Haury B, Antoine G. Les principales caractéristiques de la file active hospitalière des personnes atteintes d’infection à VIH en France en 1996. Bull Epidémiologique Hebd. 1997;23: 107–108.

[29] Morlat P. [Medical care of people living with HIV Guidelines of the experts group. 2013 Report] French. Paris: La documentation française; 2013.

[30] Legarth R, Omland LH, Kronborg G, Larsen CS, Pedersen C, Pedersen G, et al Employment status in persons with and without HIV infection in Denmark: 1996–2011. AIDS. 2014;28: 1489–1498. 10.1097/QAD.0000000000000257 24732775

[31] Groß M, Herr A, Hower M, Kuhlmann A, Mahlich J, Stoll M. Unemployment, health, and education of HIV-infected males in Germany. Int J Public Health. 2015;Epub. 10.1007/s00038-015-0750-3 26427862 PMC4947124

[32] de Boer AGEM, Taskila T, Ojajärvi A, van Dijk FJH, Verbeek JH a M. Cancer survivors and unemployment: a meta-analysis and meta-regression. JAMA. American Medical Association; 2009;301: 753–762. 10.1001/jama.2009.187 19224752

[33] Evans-Lacko S, Knapp M, McCrone P, Thornicroft G, Mojtabai R. The mental health consequences of the recession: economic hardship and employment of people with mental health problems in 27 European countries. PLoS One. 2013;8: e69792 10.1371/journal.pone.0069792 23922801 PMC3724888

[34] Minton JW, Pickett KE, Dorling D. Health, employment, and economic change, 1973–2009: repeated cross sectional study. BMJ. 2012;344: e2316 10.1136/bmj.e2316 22573646 PMC3348868

[35] Burström B, Whitehead M, Lindholm C, Diderichsen F. Inequality in the social consequences of illness: how well do people with long-term illness fare in the British and Swedish labor markets? Int J Health Serv. 2000;30: 435–51. 10.2190/6PP1-TDEQ-H44D-4LJQ 11109175

[36] Holland P, Burstrom B, Whitehead M, Diderichsen F, Dahl E, Barr B, et al How do macro-level contexts and policies affect the employment chances of chronically ill and disabled people? Part I: The impact of recession and deindustrialization. Int J Heal Serv. 2011;41: 395–413. 10.2190/HS.41.3.a 21842570

[37] van der Wel KA, Dahl E, Thielen K. Social inequalities in “sickness”: European welfare states and non-employment among the chronically ill. Soc Sci Med. 2011;73: 1608–1617. 10.1016/j.socscimed.2011.09.012 22014419

[38] France |Migrant Integration Policiy Index (MIPEX) 2015 [Internet]. 2015 [cited 2 Sep 2016]. Available: http://www.mipex.eu/france

[39] Castagnone E, Mezger C, Schoumaker B, Nazio T, Rakotonarivo N. Understanding afro-european labour trajectories: integration of migrants into the european labour market, transnational economic participation and economic reintegration into the country of origin. A comparative study. In: MAFE Working Paper n°26. 2013 p. 43. Available: www.mafeproject.com

[40] Le Barbanchon T, Malherbet F. An anatomy of the French labour market: country case study on labour market segmentation. In: Employment working paper ILO n°142. 2013 p. 45. Available: http://www.ilo.org/employment/Whatwedo/Publications/working-papers/WCMS_218969/lang—en/index.htm

[41] Rijken M, Spreeuwenberg P, Schippers J, Groenewegen PP. The importance of illness duration, age at diagnosis and the year of diagnosis for labour participation chances of people with chronic illness: results of a nationwide panel-study in The Netherlands. BMC Public Health. BMC Public Health; 2013;13: 803 10.1186/1471-2458-13-803 24007362 PMC3846917

[42] Kaye HS. The impact of the 2007–09 recession on workers with disabilities. Mon Labor Rev. 2010;133: 19–30.

[43] Heggebø K. Unemployment in Scandinavia during an economic crisis: cross-national differences in health selection. Soc Sci Med. 2015;130: 115–24. 10.1016/j.socscimed.2015.02.010 25689668

[44] Velche D, Ville I, Ravaud J-F. Thematic report on the implementation Report on the employment of disabled people in European countries Academic Network of European Disability Experts (ANED) 2009.

[45] Bury M. Chronic Illness as Biographical Disruption. Sociol Health Illn. 1982;4: 167–82. 10.1111/1467-9566.ep11339939 10260456

