# Supplementary material for: Increase in Unemployment over the 2000’s: Comparison between People Living with HIV and the French General Population
Source: PLoS One. 2016 Nov 4;11(11):e0165634. doi: 10.1371/journal.pone.0165634 (PMC5096670; doi:10.1371/journal.pone.0165634)
Supplement: S1 File — (DOCX) [file pone.0165634.s001.docx]

# Supporting Information

**Supplementary tables**

**Table A. Employment rates according to participants’ sociodemographic determinants of labor market position in 2003 and 2011 across people living with HIV and the French general population.**

|  | **2003** | | | **2011** | | |
| --- | --- | --- | --- | --- | --- | --- |
|  | **PlwHIV** | **General Pop** | ***P*-value**** | **PlwHIV** | **General Pop** | ***P*-value**** |
|  | (N=1010) | (N=175 648) |  | (N=1663) | (N=265 697) |  |
|  | %* | %* |  | %* | %* |  |
| **Sex** |  |  |  |  |  |  |
| Male | 67.0 | 78.6 | 0.00 | 64.6 | 76.4 | 0.00 |
| Female | 47.9 | 65.4 | 0.00 | 51.1 | 67.0 | 0.00 |
| **Educational level** |  |  |  |  |  |  |
| High | 72.5 | 82.3 | 0.00 | 71.6 | 83.8 | 0.00 |
| Low | 55.5 | 67.7 | 0.00 | 54.4 | 66.4 | 0.00 |
| **Country of citizenship** |  |  |  |  |  |  |
| Sub-Saharan Africa | 47.6 | 54.3 | 0.09 | 54.1 | 57.9 | 0.22 |
| Other | 45.7 | 57.6 | 0.03 | 44.0 | 57.0 | 0.03 |
| France | 67.6 | 72.9 | 0.00 | 63.7 | 72.7 | 0.00 |
| **Age (years)** |  |  |  |  |  |  |
| 25-39 | 61.4 | 80.1 | 0.00 | 61.1 | 79.7 | 0.00 |
| 40-49 | 65.3 | 82.5 | 0.00 | 64.4 | 84.2 | 0.00 |
| 50-64 | 51.7 | 53.3 | 0.67 | 51.6 | 54.8 | 0.29 |
| **Household composition** |  |  |  |  |  |  |
| Single with no children | 64.2 | 69.8 | 0.02 | 59.5 | 68.1 | 0.00 |
| Single with children | 42.4 | 67.7 | 0.00 | 45.6 | 68.6 | 0.00 |
| Cohabiting partner with children | 59.4 | 78.9 | 0.00 | 62.7 | 80.0 | 0.00 |
| Cohabiting partner. no children | 71.6 | 61.3 | 0.00 | 66.1 | 60.7 | 0.09 |
| Other cohabiting adults | 46.1 | 64.4 | 0.00 | 55.4 | 62.3 | 0.09 |

PlwHIV. People living with HIV; General Pop. French general population

* Weighted percentages

**χ^2^ test of comparison across people living with HIV and general population in 2003 and 2011

**Table B. Unemployment rates according to participants’ sociodemographic determinants of labor market position in 2003 and 2011 across people living with HIV and the French general population.**

|  | **2003** | | | **2011** | | |
| --- | --- | --- | --- | --- | --- | --- |
|  | **PlwHIV** | **General Pop** | ***P*-value**** | **PlwHIV** | **General Pop** | ***P*-value**** |
|  | (N=1010) | (N=175 648) |  | (N=1663) | (N=265 697) |  |
|  | %* | %* |  | %* | %* |  |
| **Sex** |  |  |  |  |  |  |
| Male | 10.8 | 5.4 | 0.00 | 12.8 | 6.1 | 0.00 |
| Female | 16.4 | 6.0 | 0.00 | 20.9 | 6.1 | 0.00 |
| **Educational level** |  |  |  |  |  |  |
| High | 12.7 | 5.0 | 0.00 | 13.0 | 4.3 | 0.00 |
| Low | 12.6 | 6.0 | 0.00 | 17.1 | 6.8 | 0.00 |
| **Country of citizenship** |  |  |  |  |  |  |
| Sub-Saharan Africa | 22.8 | 17.7 | 0.08 | 26.7 | 19.0 | 0.00 |
| Other | 12.1 | 10.1 | 0.52 | 20.8 | 11.0 | 0.01 |
| France | 9.2 | 5.3 | 0.00 | 10.1 | 5.6 | 0.00 |
| **Age (years)** |  |  |  |  |  |  |
| 25-39 | 14.9 | 7.6 | 0.00 | 22.8 | 8.5 | 0.00 |
| 40-49 | 12.4 | 5.8 | 0.00 | 15.6 | 6.0 | 0.00 |
| 50-64 | 5.9 | 3.4 | 0.05 | 8.2 | 3.8 | 0.00 |
| **Household composition** |  |  |  |  |  |  |
| Single with no children | 9.7 | 7.5 | 0.10 | 14.0 | 7.6 | 0.00 |
| Single with children | 15.6 | 11.0 | 0.21 | 31.7 | 12.0 | 0.00 |
| Cohabiting partner with children | 16.1 | 5.4 | 0.00 | 16.3 | 5.6 | 0.00 |
| Cohabiting partner. no children | 9.1 | 3.9 | 0.00 | 8.3 | 4.0 | 0.00 |
| Other cohabiting adults | 21.6 | 7.4 | 0.00 | 20.6 | 8.8 | 0.00 |

PlwHIV, People living with HIV; General Pop, French general population

* Weighted percentages

**χ^2^ test of comparison across people living with HIV and general population in 2003 and 2011

**Table C. Prevalence rate ratios for unemployment among people living with HIV versus the French general population in 2003 and 2011, adjusted for individual sociodemographic determinants of labor market position.**

|  | Unemployment | | |
| --- | --- | --- | --- |
|  | aPRR | | [95% CI] |
| **Survey** |  | |  |
| General population 2003 | 1 | |  |
| PlwHIV in 2003 | 1.46** | | [1.14,1.87] |
| General population 2011 | 1 | |  |
| PlwHIV in 2011 | 1.52*** | | [1.24,1.86] |
| ***Sociodemographic determinants of labor market position*** |  | |  |
| **Sex** |  |  | |
| Male | 1 |  | |
| Female | 0.91 | [0.79,1.04] | |
| **Age (years)** |  |  | |
| 25-39 | 1.28*** | [1.11,1.49] | |
| 40-49 | 1 |  | |
| 50-64 | 0.59*** | [0.49,0.72] | |
| **Immigrant status** |  |  | |
| Non-immigrants | 1 |  | |
| Sub-Saharan Africa | 2.13*** | [1.71,2.66] | |
| Other | 1.49*** | [1.25,1.79] | |
| **Educational level** |  |  | |
| High | 1 |  | |
| Low | 1.16 | [0.99,1.36] | |
| **Household composition** |  |  | |
| Single with no children | 1.41** | [1.13,1.77] | |
| Single with children | 2.06*** | [1.63,2.62] | |
| Cohabiting partner with children | 1.14 | [0.93,1.39] | |
| Cohabiting partner, no children | 1 |  | |
| Other cohabiting adults | 1.80*** | [1.39,2.35] | |

aPRR, Adjusted prevalence rate ratio; PlwHIV, People living with HIV; CI, Confidence interval

* p<0.05; ** p<0.01; *** p<0.001

P-value interaction term between type of population (general population vs. HIV population) and survey years (2003 vs. 2011) : p=0.150

**Table D. Prevalence rate ratios for unemployment among French citizens living with HIV versus French citizens from the general population in 2003 and 2011, adjusted for individual sociodemographic determinants of labor market position.**

|  | Unemployment | | |
| --- | --- | --- | --- |
|  | aPRR | | [95% CI] |
| **Survey** |  | |  |
| General population 2003 | 1 | |  |
| PlwHIV in 2003 | 1.53** | | [1.17,2.01] |
| General population 2011 | 1 | |  |
| PlwHIV in 2011 | 1.69*** | | [1.36,2.09] |
| ***Sociodemographic determinants of labor market position*** |  | |  |
| **Sex** |  |  | |
| Male | 1 |  | |
| Female | 1.08 | [0.95,1.23] | |
| **Age (years)** |  |  | |
| 25-39 | 1.35*** | [1.15,1.59] | |
| 40-49 | 1 |  | |
| 50-64 | 0.53*** | [0.44,0.65] | |
| **Educational level** |  |  | |
| High | 1 |  | |
| Low | 1.28** | [1.07,1.53] | |
| **Household composition** |  |  | |
| Single with no children | 1.40** | [1.10,1.78] | |
| Single with children | 2.32*** | [1.80,2.99] | |
| Cohabiting partner with children | 1.05 | [0.86,1.28] | |
| Cohabiting partner, no children | 1 |  | |
| Other cohabiting adults | 1.57** | [1.14,2.17] | |

aPRR, Adjusted prevalence rate ratio; PlwHIV, People living with HIV; CI, Confidence interval

* p<0.05; ** p<0.01; *** p<0.001

P-value interaction term between type of population (general population vs. HIV population) and survey years (2003 vs. 2011): p=0.390
