# Supplementary material for: Increase in Unemployment over the 2000’s: Comparison between People Living with HIV and the French General Population
Source: PLoS One. 2016 Nov 4;11(11):e0165634. doi: 10.1371/journal.pone.0165634 (PMC5096670; doi:10.1371/journal.pone.0165634)
Supplement: S2 File — (DOCX) [file pone.0165634.s002.docx]

# Supporting Information

**Translation of survey questions used to define the employment situation from the ANRS-Vespa1 and ANRS-Vespa2 questionnaires.**

Employment status was classified as followed:

*“First, participants were considered employed if they reported having a job (either paid or voluntary), regardless of their occupational status and working hours (full time/part time), or if they had been on sick leave for less than 6 months.*

*Second, the participants who were not employed were categorized as unemployed if they were available to work and reported having actively sought work within the previous 3 months.*

*Otherwise, individuals not at work and not unemployed were categorized as inactive, including students, retirees, people on disability, and those with family responsibilities. “*

A minor difference is to be noted between the two surveys: in ANRS-Vespa1 2003 survey information regarding employment was asked since HIV-diagnosis till survey time, whereas in ANRS-Vespa2-2011 survey only information at study time was reported. However information needed to classify individuals as described above were available in both datasets, only the order of question was slightly different.

**ANRS-Vespa1-2003**

**B.13 Regarding your work situation at the time you learnt your HIV infection**

-*Several answer possible* -

a. You had a declared job 🞏 _1_ 🞏 _2_

b. You had an undeclared job 🞏 _1_ 🞏 _2_

c. You were unemployed 🞏 _1_ 🞏 _2_

d. You were retired 🞏 _1_ 🞏 _2_

e. You had never worked 🞏 _1_ 🞏 _2_

f. Others.

B13 Others. Precize :……………………………

🞏 _1_ 🞏 _2_

**if B.13 a ≠ 1 {included those who hold undeclared job} 🡺 C10**

**if B.13 a = 1 {declared worked } 🡺 C1**

## C.6 Do you still hold the same job /profession /activity?

*-One Answer Only-*

🞏 _1_. Yes

🞏 _2_. No

**If C.6 = 1 🡺 C15**

***For those who do not have the same job as at HI diagnosis***

**C.7 When does this job / activity has he finished?**

/___/___/___/___/ Years /___/___/ month

**C.10 Currently…**

*-One answer only-*

🞏 _1_. You have a declared job

🞏 _2_. You are unemployed

🞏 _3_. You are retired

🞏 _4_. You have no professional activity

🞏 _98_. Do not know

🞏 _99_. Response refusal

**C.15 today, are you in sickness leave?**

*-One answer only-*

🞏 _1_. Yes

🞏 _2_. No

**Si C.15=1**🡺 **C.15bis**

**C.15bis Since when (***Date of your last working day)* **?**

/___/___/___/___/ Years /___/___/ month

- ***For those who do not currently hold a job (declared or not) (C10>1)***

We will talk of your last occupation (declared)

**C.26 When did you left your last declared occupation?**

⎜___⎜___⎜___⎜___⎜ years ⎜___⎜___⎜ month

🞏 _00_. Has never had a declared occupation

**if C.26 = C.7 (if the end date of the last occupation correspond to the end date of job held at the time of diagnosis) OR if C.26 = 00 (never had an occupation) 🡺 C.32**

**if C.26 ≠ C.7 (dates do not match) 🡺 C.27**

**C.32 Now do you plan to work or re-work (declared position)?**

*-One Answer Only-*

🞏 _1_. Yes ***🡺 C36 > C40***

🞏 _2_. No ***🡺 C33 > C35 bis***

🞏 _3_. Not concerned (retirement or early retirement) ***🡺 C42***

**If C.32=1** => **C.37 (for people who are considering going back to work)**

**C.37 During the last 3 months, have you actively sought for a job?**

*-One answer only-*

🞏 _1_. Yes

🞏 _2_. No

🞏 97. Not concerned (retirement or early retirement)

🞏 98. Do not know

🞏 99. Response refusal

**ANRS-Vespa2-2011**

**C.1 Currently, what is your situation?**

*- Several answers possible-*

🞏 1. You have a declared job 🡺**C3**

🞏 2 You have an undeclared job or you are doing unformal job

🞏 3. You are in a paid training program

🞏 4. You are unemployed

🞏 5.. You are retired

🞏 6. You are disabled

🞏 7. You have no professional activity

🞏 8. Other situation: specify………🡪

🞏 97. Not concerned

🞏 99. Response refusal

**If C1=1 (individual has a declared job)** 🡺**C3**

**If C1≠1 ET C1=2 à 20 (Individual has no job declared currently)** 🡺 **C35**

**C.8 Today, are you in sickness leave?**

🞏 1. Yes 🡺 **C8.1**

🞏 2. No 🡺 **C9**

🞏 97. Not concerned 🡺 **C9**

🞏 98. Do not know 🡺 **C9**

🞏 99. Response refusal 🡺 **C9**

**Si C8=1**🡺 **C8.1**

**C8.1 Since when** (*Date of your last working day)* **?**

/___/___/___/___/ Year /___/___/ month /___/___/day

**If C1 ≠ 1 (individuals who do not hold a declared job) AND C1 = 2 à 20 => C35**

We'll talk about your last occupation (declared)

**C.35 when did you left your last declared occupation?**

/___/___/___/___/ year

/___/___/ month

🞏 2. Has never had a declared occupation 🡺 **C46**

**C.46 Today do you plan to work or re-work (declared job) ?**

*-One Answer Only-*

🞏 1. Yes

🞏 2. No

🞏 97. Not concerned (retirement or early retirement) 🡺**C50**

🞏 98. Do not know 🡺**C50**

🞏 99. Response refusal 🡺**C50**

**If C46=1** 🡺 **C 48 (for people who are considering going back to work)**

**C.48 During the last 3 months, have you actively sought for a job?**

*-One Answer Only-*

🞏 1. Yes

🞏 2. No

🞏 97. Not concerned (retirement or early retirement) 🡺**C50**

🞏 98. Do not know 🡺**C50**

🞏 99. Response refusal 🡺**C50**
