# Supplementary material for: Increase in Unemployment over the 2000’s: Comparison between People Living with HIV and the French General Population
Source: PLoS One. 2016 Nov 4;11(11):e0165634. doi: 10.1371/journal.pone.0165634 (PMC5096670; doi:10.1371/journal.pone.0165634)
Supplement: S3 File — (PDF) [file pone.0165634.s003.pdf]

VIH : Enquête Sur les Personnes Atteintes

Une enquête de l'ANRS

Agence nationale
de recherches sur le sida

QUESTIONNAIRE

1 Date

|___|___| (jour) |___|___| (mois) |___|___|___|___| (année)

2 Type de structure

☐ 1. CHU
☐ 2. CH
☐ 3. Privé

3 Numéro de département de la structure :

|___|___|

4 Heure de début de l'entretien |___|___| H |___|___| mm

5 Sexe de la personne interviewée

☐ 1. Homme
☐ 2. Femme

Module A socio-démographique

A0 Quel est votre département de résidence ?

|___|___|

➔ ***Pour l'enquêteur :***
Si pas de département de résidence fixe : Inscrire 99

A1 Quelle est actuellement votre situation matrimoniale, au sens légal ?
- *Une seule réponse possible*-

- ☐ 1. Célibataire
- ☐ 2. Marié(e) ou remarié(e)
- ☐ 3. Pacsé(e)
- ☐ 4. Divorcé(e)
- ☐ 5. Séparé(e)
- ☐ 6. Veuf /ve

A2 Combien de personnes vivent dans votre logement, y compris vous-même ?

|___|___|

A3 Composition du ménage

Inscrire dans ce tableau la personne interviewée et toutes les personnes vivant normalement avec elle (conjoint(e) marié(e) ou de fait, enfants (petits-enfants) et/ou ceux du/de la conjoint(e), parents et/ou ceux de du/de la conjoint(e), autres personnes à charge).

Comptabiliser les personnes qui sont temporairement absentes à la date de l'enquête, y compris les militaires logés en caserne, les élèves en internat, les étudiants en cité universitaire, les jeunes vivant dans un foyer de jeunes travailleurs.

➔ ***Consigne pour l'enquêteur : Pour le pays de naissance, la nationalité, le niveau d'études, le diplôme obtenu, l’occupation actuelle et les principales ressources, <u>ne prendre en compte que les personnes de plus de 16 ans</u>.***

<table>
<tr><th></th><th>A3S</th><th>A3A</th><th>A3L</th><th>A3P</th><th>A3N</th><th>A3E</th><th>A3O</th></tr>
<tr><th>N° d’ordre individuel</th><th>Sexe
1. homme
2. femme</th><th>Année de naissance</th><th>Lien avec la personne de référence (L)</th><th>Pays de naissance
(P)</th><th>Nationalité
(N)</th><th>Niveau d’études
(E)</th><th>Occupation actuelle
(O)</th></tr>
<tr><td>|01|</td><td>|__|</td><td>|__|__|__|__|</td><td>|01|</td><td>|__|</td><td>|__|__|__|</td><td>|__|__|</td><td>|__|</td></tr>
<tr><td>|02|</td><td>|__|</td><td>|__|__|__|__|</td><td>|__|</td><td>|__|</td><td>|__|__|__|</td><td>|__|__|</td><td>|__|</td></tr>
<tr><td>|03|</td><td>|__|</td><td>|__|__|__|__|</td><td>|__|</td><td>|__|</td><td>|__|__|__|</td><td>|__|__|</td><td>|__|</td></tr>
<tr><td>|04|</td><td>|__|</td><td>|__|__|__|__|</td><td>|__|</td><td>|__|</td><td>|__|__|__|</td><td>|__|__|</td><td>|__|</td></tr>
<tr><td>|05|</td><td>|__|</td><td>|__|__|__|__|</td><td>|__|</td><td>|__|</td><td>|__|__|__|</td><td>|__|__|</td><td>|__|</td></tr>
<tr><td>|06|</td><td>|__|</td><td>|__|__|__|__|</td><td>|__|</td><td>|__|</td><td>|__|__|__|</td><td>|__|__|</td><td>|__|</td></tr>
<tr><td>|07|</td><td>|__|</td><td>|__|__|__|__|</td><td>|__|</td><td>|__|</td><td>|__|__|__|</td><td>|__|__|</td><td>|__|</td></tr>
<tr><td>|08|</td><td>|__|</td><td>|__|__|__|__|</td><td>|__|</td><td>|__|</td><td>|__|__|__|</td><td>|__|__|</td><td>|__|</td></tr>
<tr><td>|09|</td><td>|__|</td><td>|__|__|__|__|</td><td>|__|</td><td>|__|</td><td>|__|__|__|</td><td>|__|__|</td><td>|__|</td></tr>
<tr><td>|10|</td><td>|__|</td><td>|__|__|__|__|</td><td>|__|</td><td>|__|</td><td>|__|__|__|</td><td>|__|__|</td><td>|__|</td></tr>
<tr><td>|11|</td><td>|__|</td><td>|__|__|__|__|</td><td>|__|</td><td>|__|</td><td>|__|__|__|</td><td>|__|__|</td><td>|__|</td></tr>
<tr><td>|12|</td><td>|__|</td><td>|__|__|__|__|</td><td>|__|</td><td>|__|</td><td>|__|__|__|</td><td>|__|__|</td><td>|__|</td></tr>
<tr><td>|13|</td><td>|__|</td><td>|__|__|__|__|</td><td>|__|</td><td>|__|</td><td>|__|__|__|</td><td>|__|__|</td><td>|__|</td></tr>
<tr><td>|14|</td><td>|__|</td><td>|__|__|__|__|</td><td>|__|</td><td>|__|</td><td>|__|__|__|</td><td>|__|__|</td><td>|__|</td></tr>
<tr><td>|15|</td><td>|__|</td><td>|__|__|__|__|</td><td>|__|</td><td>|__|</td><td>|__|__|__|</td><td>|__|__|</td><td>|__|</td></tr>
<tr><td>|16|</td><td>|__|</td><td>|__|__|__|__|</td><td>|__|</td><td>|__|</td><td>|__|__|__|</td><td>|__|__|</td><td>|__|</td></tr>
<tr><td>|17|</td><td>|__|</td><td>|__|__|__|__|</td><td>|__|</td><td>|__|</td><td>|__|__|__|</td><td>|__|__|</td><td>|__|</td></tr>
<tr><td>|18|</td><td>|__|</td><td>|__|__|__|__|</td><td>|__|</td><td>|__|</td><td>|__|__|__|</td><td>|__|__|</td><td>|__|</td></tr>
<tr><td>|19|</td><td>|__|</td><td>|__|__|__|__|</td><td>|__|</td><td>|__|</td><td>|__|__|__|</td><td>|__|__|</td><td>|__|</td></tr>
<tr><td>|20|</td><td>|__|</td><td>|__|__|__|__|</td><td>|__|</td><td>|__|</td><td>|__|__|__|</td><td>|__|__|</td><td>|__|</td></tr>
<tr><td>|21|</td><td>|__|</td><td>|__|__|__|__|</td><td>|__|</td><td>|__|</td><td>|__|__|__|</td><td>|__|__|</td><td>|__|</td></tr>
<tr><td>|22|</td><td>|__|</td><td>|__|__|__|__|</td><td>|__|</td><td>|__|</td><td>|__|__|__|</td><td>|__|__|</td><td>|__|</td></tr>
<tr><td>|23|</td><td>|__|</td><td>|__|__|__|__|</td><td>|__|</td><td>|__|</td><td>|__|__|__|</td><td>|__|__|</td><td>|__|</td></tr>
</table>

Code L : Lien avec la personne interviewée

2. Conjoint(e) /compagnon ou compagne de la personne interviewée
3. Enfants de la personne interviewée
4. Autres enfants (de son/sa conjoint(e), y compris enfant adopté, neveux, etc.)
5. Parents ou beaux-parents de la personne interviewée
6. Autre(s) personne(s)

Code P : Pays de naissance

1. France métropolitaine
2. Dom-Tom
3. Pays membres de l'union européenne
4. Autres pays européens
5. Afrique du Nord
6. Afrique sub-saharienne
7. Asie
8. Autre

Code N : Nationalité (page suivante)

Code E : Niveau d'études *(Consigne : présenter la carte 1)*

00. N'a jamais fait d'études
02. **Etudes primaires** du cours préparatoire (CP) au cours moyens 2^{é} année (CM2 ou 7^{é})
15. **1er cycle** d'enseignement général (6^{é} à 3^{é}), études primaires supérieures
17. **2^{é} cycle** d'enseignement général (2nde à terminale) préparation au baccalauréat général ou au brevet supérieur
20. Enseignement spécialisé ou adapté de niveau professionnel court (établissements sociaux-éducatifs, SES, CAT)
21. Classe pré-professionnelle de niveau (CPPN), de préapprentissage (CPA), etc.
22. Apprentissage, préparation à un **CAP**, à un **BEP** ou équivalent
30. Préparation au **bac** général, à un brevet de technicien ou à un bac de technicien, à un bac professionnel, à un brevet d'enseignement agricole, commercial, industriel, etc.
41. **1er cycle** universitaire, IUT, BTS, classes préparatoires aux grandes écoles, cycle de formation des instituteurs, des infirmiers, etc.
46. **2^{é} cycle** universitaire (préparation d'une licence ou d'une maîtrise)
47. **3^{é} cycle** universitaire, doctorat, CAPES, agrégation, formation dans une école d'ingénieur ou grande école

Code O : Occupation actuelle

1. Travaille : exerce une profession à son compte ou comme salarié (même à temps partiel), aide un membre de sa famille dans son travail même sans être rémunéré, est apprenti, stagiaire rémunéré en entreprise, élève-fonctionnaire, intérimaire, CES, etc., y compris en congés de maladie, de maternité, congés annuels, dispenses d'activité, congés de conversion, etc. Exclure les personnes effectuant leur service militaire, en disponibilité, en pré-retraite, en invalidité.
2. Chômeurs (inscrit ou non à l'ANPE)
3. Etudiant, élève, en formation ou en stage non rémunéré, ou hors entreprise
4. Militaires du contingent
5. Retraité (ancien salarié) ou pré-retraité
6. Retiré des affaires (ancien agriculteur, ancien artisan, ancien commerçant…)
7. Femme ou homme au foyer
8. Autre inactif (y compris les personnes ne touchant qu'une pension de reversions, les personnes invalides et les étrangers n'ayant pas le droit de travailler)

Code N : Nationalité

0 **Française**
001 – Française de naissance
002 – Française par acquisition
009 – Non réponse

1 **Union européenne**
101 – Portugaise
102 – Italienne
103 – Espagnole
104 – Allemande
105 – Britannique
106 – Belge
107 – Néerlandaise
108 – Grecque
109 – Suédoise
110 – Danoise
111 – Irlandaise (Eire)
112 – Autrichienne
113 – Luxembourgeoise
114 – Finlandaise

2 **Espace économique européen** (hors UE)
201 – Norvégienne
202 – Islandaise

3 **Autre Europe**
301 – Turque
302 – Polonaise
303 – Suisse
304 – Roumaine
305 – Slovaque
306 – Tchèque
307 – Hongroise
308 – Bulgare
309 – Russe
310 – Ukrainienne
311 – Autre nationalité de l'ex-URSS
312 – Yougoslave : Serbe et Monténégrine
313 – Croate
314 – Bosniaque
315 – Autre nationalité de l'ex-Yougoslavie
316 – Albanaise
317 – Maltaise
318 – Autre nationalité d'Europe

4 **Maghreb**
401 – Algérienne
402 – Marocaine
403 – Tunisienne

5 **Afrique** (hors Maghreb)
501 – Sénégalaise
502 – Malienne
503 – Zaïroise
504 – Camerounaise
505 – Ivoirienne
506 – Mauricienne
507 – Congolaise
508 – Malgache
509 – Mauritanienne
510 – Egyptienne
511 – Togolaise
512 – Guinéenne
513 – Béninoise
514 – Centrafricaine
515 – Gabonaise
516 – Comorienne
517 – Ghanéenne
518 – Burkinabaise
519 – Tchadienne
520 – Nigérienne
521 – Nigériane
522 – Sud-africaine
523 – Autre nationalité d'Afrique (hors Maghreb)

6 **Amérique et Caraïbes**
601 – Amérique (USA)
602 – Canadienne
603 – Haïtienne
604 – Autre nationalité des Caraïbes
605 – Mexicaine
606 – Autre nationalité d'Amérique Centrale
607 – Chilienne
608 – Brésilienne
609 – Colombienne
610 – Argentine
611 – Péruvienne
612 – Vénézuélienne
613 – Autre nationalité d'Amérique du Sud

7 **Asie**
701 – Cambodgienne
702 – Vietnamienne
703 – Laotienne
704 – Libanaise
705 – Iranienne
706 – Syrienne
707 – Israélienne
708 – Irakienne
709 – Chinoise
710 – Japonaise
711 – Sri Lankaise (Ceylanaise)
712 – Pakistanaise
713 – Indienne
714 – Coréenne
715 – Philippine
716 – Thaïlandaise
717 – Indonésienne
718 – Autre nationalité d'Asie

8 **Océanie**
801 – Australienne
802 – Néo-zélandaise
803 – Autre nationalité d'Océanie

9 **Apatride**

Si A3N 1 = 4.. OU 5.. { ≠ nat. du Maghreb ou d'Afrique sud saharienne} ➔ A4 ;
si A3N 1 ≠ 4.. OU 5.. ➔ A5

A4 Avez-vous plusieurs épouses / votre conjoint a-t-il plusieurs épouses ? *-Une seule réponse possible-*
- ☐ 1. Oui
- ☐ 2. Non

A5 Avez-vous (aussi) des enfants qui ne vivent pas dans votre foyer ? *–Une seule réponse possible-*
- ☐ 1. Oui
- ☐ 2. Non ➔ ***A6***

Si A5 = 2 ➔ A6

A5 bis Combien ? |___|

	**A5 ter Quelle est leur année de naissance ?**	**A5 qua Où habitent-ils ? (G)**
1er enfant	|__|__|__|__|	|__|
2ème enfant	|__|__|__|__|	|__|
3ème enfant	|__|__|__|__|	|__|

Code G : lieu de résidence des enfants
1. Chez des proches (père, mère…) en France
2. Dans un foyer ou une famille d'accueil
3. Chez des proches au pays
4. Enfant adulte installé
5. Autre

A6 Vous avez été élevé(e) dans quelle religion ? *-Une seule réponse possible-*
- ☐ 1. Aucune ➔ ***A7***
- ☐ 2. Catholique
- ☐ 3. Israélite
- ☐ 4. Musulmane
- ☐ 5. Protestante
- ☐ 6. Autre

Si A6 = 1 ➔ A7

A6 bis Est-ce que la religion est dans votre vie… *-Une seule réponse possible-*
- ☐ 1. Très importante
- ☐ 2. Importante
- ☐ 3. Pas très importante
- ☐ 4. Pas importante du tout

➔ ***Pour l'enquêteur***
- ☐ 98. Ne sait pas
- ☐ 99. Refus de répondre

A7 Quelle est (ou était) la profession de…

a. Votre mère* ? ____ (en clair)
➔ ***Pour l'enquêteur*** ☐ Non concerné(e) (ne connaît pas sa mère*)*

b. Votre père* ? ____ (en clair)
➔ ***Pour l'enquêteur*** ☐ Non concerné(e) (ne connaît pas son père*)*

**ou des personnes ayant élevé le sujet*

Si A3N 1 = 0.. {nationalité française} ➔ Module B

➔ **POUR LES PERSONNES DE NATIONALITE ETRANGERE**

A8 A quelle date êtes-vous arrivé(e) en France ?

|___|___|___|___| année |___|___| mois (➔ *Insistez pour avoir le mois, si l'année est récente*)

A9 Actuellement avez-vous un titre de séjour ?

- ☐ 1. Oui
- ☐ 2. Non → ***A13***

Si A9 = 1 ➔ A10 > A12 ; si A9 = 2 ➔ A13

A10 La durée de ce titre de séjour est… -*Une seule réponse possible*-

- ☐ 1. Inférieure ou égale à 3 mois
- ☐ 2. Comprise entre plus de 3 mois et moins d'1 an
- ☐ 3. De 1 an
- ☐ 4. De 10 ans

A11 Avez-vous le droit de travailler ? ➔ ***Pour l'enquêteur***

- ☐ 1. Oui
- ☐ 2. Non
- ☐ 98. Ne sait pas
- ☐ 99. Refus de répondre

A12 Avez-vous un permis de séjour <u>pour soins</u> ? ➔ ***Pour l'enquêteur***

- ☐ 1. Oui
- ☐ 2. Non
- ☐ 98. Ne sait pas
- ☐ 99. Refus de répondre

→ ***Module B***

A13 Si non… ➔ ***Pour l'enquêteur***

- ☐ 1. Vous l'attendez
- ☐ 2. Il vient d'expirer
- ☐ 3. Il vous a été refusé
- ☐ 4. Vous ne l'avez jamais demandé
- ☐ 5. Vous ne résidez pas en France (non résident)
- ☐ 6. Autre. **A13 Autre**. Précisez : ______________________________
- ☐ 98. Ne sait pas
- ☐ 99. Refus de répondre

→ ***Module B***

Module B «Histoire de la maladie, état de santé et accès aux soins»

➔ ***A TOUS***

B1 Quand avez-vous eu connaissance de votre infection à VIH ?

/____/___/___/___/ année /___/___/ mois (***Consigne : insister pour avoir le mois quand la date est récente***)

B2 Savez-vous comment vous avez été infecté(e) **?** *-Plusieurs réponses possibles-*

	Oui	Non	➔ *Pour l'enquêteur*
a. Par transfusion	☐ 1	☐ 2.	☐ 99. Refus de répondre
b. Par produits anti-hémophiliques	☐ 1	☐ 2	☐ 98. Ne sait pas
c. Par relations sexuelles avec un homme	☐ 1	☐ 2	
d.. Par relations sexuelles avec une femme	☐ 1	☐ 2	
e. Par usage de drogues en intraveineux	☐ 1	☐ 2	
f. Par accident professionnel	☐ 1	☐ 2	

B3 Où avez-vous fait le dépistage qui a conduit au diagnostic de votre infection à VIH ?
–une seule réponse possible-

➔ ***Pour l'enquêteur*** ☐ 98. Ne sait pas ☐ 99. Refus de répondre

- ☐ 1. Dans un laboratoire d'analyses médicales en ville
- ☐ 2. A l'hôpital
- ☐ 3. Dans un centre de transfusion sanguine
- ☐ 4. Dans un Centre de Dépistage Anonyme et Gratuit (CDAG)
- ☐ 5. En prison (lors d'une incarcération)
- ☐ 6. Dans votre pays d'origine ou à l'étranger
- ☐ 7. Autre. **B4 Autre**. Précisez : ________________________________

B4 Quelles étaient les circonstances de ce dépistage ?
-une seule réponse possible-

➔ ***Pour l'enquêteur*** ☐ 98. Ne sait pas ☐ 99. Refus de répondre

- ☐ 1. A l'occasion d'un bilan de santé (check-up, etc.)
- ☐ 2. Suite à des symptômes (fièvre, amaigrissement...) ou une maladie (MST, etc.)
- ☐ 3 . Suite à une consultation médicale
- ☐ 3 Suite à une hospitalisation pour une autre raison (accident, opération, etc.)
- ☐ 4. A l'occasion d'un examen pré-natal
- ☐ 5. A l'occasion d'un test pré-nuptial
- ☐ 6. A l'occasion d'un don de sang
- ☐ 7. Après un rapport sans préservatif, l'éclatement d'un préservatif, un partage de seringue, etc.
- ☐ 8. A l'occasion de l'annonce de l'infection à VIH de votre conjoint(e) ou d'un/e partenaire sexuel/le
- ☐ 9. Pour savoir où vous en étiez avec le VIH

B5 Lorsque le test a été réalisé, c'était…
-une seule réponse possible-

➔ ***Pour l'enquêteur*** ☐ 98. Ne sait pas ☐ 99. Refus de répondre

- ☐ 1. De votre propre initiative
- ☐ 2. Sur la proposition d'un médecin
- ☐ 3. A la demande de votre partenaire/conjoint
- ☐ 4. C'est le médecin qui a insisté
- ☐ 5. A votre insu

<u>Au moment où vous avez appris votre infection à VIH</u> :

NOUS ALLONS ABORDER MAINTENANT VOTRE MODE DE VIE AU MOMENT OU VOUS AVEZ APPRIS VOTRE INFECTION A VIH.

B6 Vous étiez seul(e) ou en couple -c'est-à-dire avec une personne avec laquelle vous aviez une relation affective et/ou sexuelle régulière, <u>que vous viviez ensemble ou non</u>-
-Une seule réponse possible-

- ☐ 1. Seul(e)
- ☐ 2. En couple avec un homme
- ☐ 3. En couple avec une femme

Si B6 = 1 ET A3S 1 = 2 {✿} ➔ B12 ; Si B6 = 1 ET A3S 1 = 1 {❝} ➔ B13

B7 Vous étiez en couple depuis quand ?
/___/___/___/___/ Année

B8 Connaissiez-vous alors le statut sérologique de votre conjoint(e)/ami(e) ? *-Une seule réponse possible-*

- ☐ 1. Oui, il (elle) était séropositif(ve)
- ☐ 2. Oui, vous avez appris votre séropositivité en même temps
- ☐ 3. Oui, il (elle) était séronégatif(ve)
- ☐ 4. Non, vous ne connaissiez pas son statut

Si (B8 = 1 OU B8 = 2) ET [(A3S 1 = 2 {✿}) OU (A3S 1 = 1 {❝} ET B6 = 3)] ➔ B12 ;
Si (B8 = 1 OU B8 = 2) ET (A3S 1 = 1 {❝} ET B6 = 2) ➔ B13

B9 Quand vous avez appris votre infection à VIH, avez-vous mis au courant votre conjoint(e) ou ami(e) ?
-Une seule réponse possible-

- ☐ 1. Oui, dès que vous l'avez su vous-même
- ☐ 2. Oui, mais vous avez attendu quelques semaines
- ☐ 3. Oui, mais vous avez attendu quelques mois
- ☐ 4. Oui, mais vous avez attendu quelques années
- ☐ 5. Non, vous n'avez rien dit ➔ ***B13 OU B14***

➔ Pour l'enquêteur

- ☐ 98. Ne sait pas
- ☐ 99. Refus de répondre

Si B9 = 5 ET [A3S 1 = 2 {✿} OU (A3S 1 = 1 {❝} ET B6 = 3)] ➔ B12
Si B9 = 5 ET (A3S 1 = 1 {❝} ET B6 = 2) ➔ B13

B10 Suite à cette annonce, a-t-il / a-t-elle fait un test de dépistage du VIH ? *-Une seule réponse possible-*

- ☐ 1. Oui
- ☐ 2. Non ➔ ***B12 OU B13***
- ☐ 3. Non, mais vous avez appris à cette occasion qu'il/qu'elle était séropositif/ive ➔ ***B12 OU B13***

➔ Pour l'enquêteur

- ☐ 99. Refus de répondre
- ☐ 98. Ne sait pas

Si (B10 = 2 OU B10 = 3) ET [A3S 1 = 2 {✿} OU (A3S 1 = 1 {❝} ET B6 = 3)] ➔ B12
Si (B10 = 2 OU B10 = 3) ET (A3S 1 = 1 {❝} ET B6 = 2) ➔ B13

B11 Il/elle était alors : *-Une seule réponse possible-*

- ☐ 1. Séronégatif
- ☐ 2. Séropositif

➔ Pour l'enquêteur

- ☐ 99. Refus de répondre
- ☐ 98. Ne sait pas

Si A3S 1 = 2 {✿} OU (A3S 1 = 1 {❝} ET B6 = 3) ➔ B12 ; Si A3S 1 = 1 {❝} ET B6 = 2 ➔ B13

B12 Au moment où vous avez appris votre infection à VIH, votre conjointe était enceinte OU vous étiez enceinte ? *-Une seule réponse possible-*

- ☐ 1. Oui
- ☐ 2. Non ➔ ***B13***

B12 bis S'agissait-il d'une grossesse…*-Une seule réponse possible-*

- ☐ 1. Désirée

☐ 2. Accidentelle

B12 ter Cette grossesse a-t-elle été… -*Une seule réponse possible***-**

☐ 1. Poursuivie
☐ 2. Interrompue à cause de votre infection à VIH
☐ 3. Interrompue pour d'autres raisons

➔ ***A TOUS***

B13 Concernant le travail au moment où vous avez appris votre infection à VIH…

-*Plusieurs réponses possibles*-	**Oui**	**Non**
a. Vous aviez un emploi déclaré	☐ 1	☐ 2
b. Vous aviez un emploi non déclaré	☐ 1	☐ 2
c. Vous étiez au chômage	☐ 1	☐ 2
d. Vous étiez à la retraite	☐ 1	☐ 2
e. Vous n'aviez jamais travaillé	☐ 1	☐ 2
f. Autre.	☐ 1	☐ 2

B13 Autre. Précisez : ________________________________

**B14 Concernant vos revenus…** -*Plusieurs réponses possibles*-	**Oui**	**Non**
a. Vous perceviez un salaire ou un revenu de votre activité professionnelle	☐ 1	☐ 2
b. Vous touchiez des indemnités de chômage (ASSEDIC ou autres)	☐ 1	☐ 2
c. Vous touchiez le RMI	☐ 1	☐ 2
d. Vous touchiez une Allocation Parent Isolé (API)	☐ 1	☐ 2
e. Vous perceviez des indemnités journalières (arrêt-maladie)	☐ 1	☐ 2
f. Vous perceviez une retraite	☐ 1	☐ 2
g. Vous aviez une allocation pour adulte handicapé (AAH)	☐ 1	☐ 2
h. Vous aviez une pension d'invalidité	☐ 1	☐ 2
i. Vous n'aviez aucune ressource	☐ 1	☐ 2

B15 Résidiez-vous en France au moment où vous avez appris votre infection à VIH ?

☐ 1. Oui
☐ 2. Non

B16 Concernant votre logement… -*Une seule réponse possible***-**

☐ 1. Vous aviez un logement
☐ 2. Vous étiez chez vos parents
☐ 3. Vous viviez chez des amis
☐ 4. Vous viviez en foyer
☐ 5. Vous étiez en prison
☐ 6. Vous étiez sans domicile fixe
☐ 7. Autre situation. **B15 Autre**. Précisez : ________________________________

B17 Toujours au moment où vous avez appris votre infection à VIH, est-ce que vous consommiez des drogues par injection ? -*Une seule réponse possible*-

☐ 1. Oui
☐ 2. Non, mais vous en aviez déjà consommé dans le passé, même une seule fois
☐ 3. Non et vous n'en aviez jamais consommé ➔ ***B18***

B17 = 3 ➔ ***B18***

B17 bis Au moment où vous avez appris votre infection, est-ce que vous suiviez un traitement de substitution (méthadone, Subutex…) ? -*Une seule réponse possible*-

☐ 1. Oui
☐ 2. Non

B18 Après avoir appris que vous étiez infecté(e), combien de temps s'est écoulé avant que vous alliez voir un médecin pour vous faire suivre pour le VIH ? *-Une seule réponse possible-*

☐ 1. Moins d'un mois
☐ 2. Entre un mois et 6 mois
☐ 3. Entre 6 mois et un an
☐ 4. Plus d'un an. ➔ **B18 An. Précisez en quelle année** : /___/___/___/___/

B19 Depuis que vous avez appris votre infection, vous diriez que votre suivi médical a été…
Consigne à l'enquêteur ➔ suivi médical = le fait de consulter un médecin
-une seule réponse possible-

☐ 1. Toujours régulier (depuis que vous connaissez votre infection à VIH) → ***B20***
☐ 2. D'abord irrégulier, puis régulier
☐ 3. Toujours irrégulier
☐ 4. Parfois régulier, parfois irrégulier

Si B19 = 1 ➔ B20 ; si B19 > 1 ➔ B19 bis

B19 bis Quelle a été la plus longue durée sans consulter un médecin pour le VIH?

_________________ (en mois ou en année)

Si B19 = 2 ➔ B19 ter ; Si B19 = 3 OU B19 = 4 ➔ B20

B19 ter Quelles sont les raisons qui vous ont amené à vous faire suivre de nouveau ?
–Plusieurs réponses possibles-

	**Oui**	**Non**
a. L'apparition de symptômes	☐ 1	☐ 2
b. Les nouvelles thérapies	☐ 1	☐ 2
c. Votre entourage	☐ 1	☐ 2
d. Autre	☐ 1	☐ 2

B19 Autre. Précisez : ____

B20 Depuis que vous avez appris votre infection à VIH, avez-vous été hospitalisé(e) au moins une nuit en raison d'un problème de santé lié à votre infection à VIH ? (y compris à cause des effets secondaires)
-Une seule réponse possible-

☐ 1. Oui
☐ 2. Non, jamais ➔ ***B22***

Si B20 = 1 ➔ B21 ; si B20 = 2 ➔ B22

➔ POUR LES PERSONNES QUI ONT DEJA ETE HOSPITALISEES EN RAISON D'UN PROBLEME DE SANTE LIE AU VIH

B21 Combien de fois ?

a. Au cours de la dernière année ? |___|___| en nombre de fois

b. Depuis plus d'un an ? |___|___| en nombre de fois

➔ ***A TOUS***

B22 Depuis que vous avez appris votre infection à VIH, avez-vous pris à un moment donné du Bactrim® ? -*Une seule réponse possible-*

☐ 1. Oui
☐ 2. Non

➔ ***Pour l'enquêteur***
☐ 98. Ne sait pas / ☐ 99. Refus de répondre

B23 Vous êtes suivi(e) principalement pour le VIH ? -*Une seule réponse possible-*
(➔ Pour l'enquêteur : Si c'est le même médecin qui suit le sujet à l'hôpital et dans un cabinet en ville, cocher « hôpital »)

☐ 1. Dans un cabinet de médecine en ville
☐ 2. Dans cet hôpital
☐ 3. Dans un autre hôpital

➔ ***Pour l'enquêteur***
☐ 98. Ne sait pas
☐ 99. Refus de répondre

B24 Tous les combien êtes-vous venu(e) consulter votre médecin qui vous suit pour le VIH dans ce service au cours des 12 derniers mois –hors hospitalisation classique- ? ***(➔ Pour l'enquêteur : on ne prend en compte que les consultations avec le médecin pour le VIH)***
-Une seule réponse possible-

☐ 1. Une fois dans l'année
☐ 2. Tous les 6 mois
☐ 3. Tous les 4 mois
☐ 4. Tous les 3 mois
☐ 5. Tous les 2 mois
☐ 6. Tous les mois
☐ 7. Plus

(2 à 7) → ***Ou, si la personne n'arrive pas à répondre***
B24' Nombre de consultations avec ce médecin dans les 12 derniers mois y compris ce jour : /____/____/

B25 Pour consulter le médecin qui vous suit pour l'infection à VIH à l'hôpital, vous devez vous déplacer (aller simple)… -*Une seule réponse possible-*

☐ 1. A moins de 30 minutes
☐ 2. Entre 30 minutes et 1 heure
☐ 3. Entre 1 heure et 2 heures → B26
☐ 4. Plus de 2 heures → B26

Ou, si la personne n'arrive pas à répondre

☐ 1. A moins de 10 km
☐ 2. Entre 10 et 50 km
☐ 3. Entre 50 et 100 km → B26
☐ 4. Plus de 100 km → B26

Si B25 = 1 OU 2 ➔ B27

B26 Pour quelle raison principale avez-vous choisi cet hôpital ?
-Une seule réponse possible-

☐ 1. Il n'y a pas d'hôpital plus proche
☐ 2. Par discrétion
☐ 3. Parce que vous pensez que vous êtes mieux soigné(e) dans cet hôpital
☐ 4 . Parce que vous avez toujours eu l'habitude de venir dans cet hôpital

B27 Est-ce qu'un traitement antirétroviral vous est prescrit ? ***(➔ pour l'enquêteur, inclure les anti-protéases)*** *-Une seule réponse possible-*

☐ 1. Oui, vous avez un traitement prescrit actuellement ➔ ***B30***
☐ 2. Non, pas actuellement mais vous avez déjà pris un traitement antirétroviral dans le passé ➔ ***B28***
☐ 3. Non, pas actuellement et on ne vous a jamais prescrit un traitement antirétroviral dans le passé ➔ ***B29***

➔ ***POUR CEUX QUI N'ONT PAS ACTUELLEMENT DE TRAITEMENT ANTIRETROVIRAL PRESCRIT MAIS QUI EN ONT DEJA EU DANS LE PASSE***

B28 Quand vous a-t-on prescrit votre premier traitement antirétroviral quel qu'il soit (AZT et/ou inhibiteur de protéase) –ce n'est pas forcément celui que vous prenez actuellement- ?

/___/___/___/___/ année

B28 bis Vous avez interrompu le traitement…-*Une seule réponse possible-*
- ☐ 1. En accord avec le médecin suite à votre initiative
- ☐ 2. En accord avec le médecin suite à l'initiative du médecin ou c'est le médecin qui l'a décidé
- ☐ 3. Sans l'accord du médecin

**B28 ter Vous avez interrompu le traitement à cause…** -*Plusieurs réponses possibles-*	**Oui**	**Non**
a. De la contrainte des prises des médicaments	☐ 1	☐ 2
b. D'effets secondaires	☐ 1	☐ 2
c. De l'insuffisance de résultats	☐ 1	☐ 2
d. Par lassitude	☐ 1	☐ 2
e. Parce que vous êtes entré(e) dans un essai comprenant une interruption thérapeutique	☐ 1	☐ 2
f. D'une autre raison	☐ 1	☐ 2

B28 ter Autre. Précisez : ______________________________________

➔ ***Pour l'enquêteur Allez à B40***

➔ ***POUR CEUX QUI N'ONT JAMAIS EU DE PRESCRIPTION DE TRAITEMENT ANTIRETROVIRAL***

B29 Vous n'avez jamais eu de prescription de traitement antirétroviral parce que…
-*Une seule réponse possible-*
- ☐ 1. Vous n'aviez pas de suivi médical
- ☐ 2. Votre médecin pense qu'un traitement antirétroviral n'est pas nécessaire pour l'instant
- ☐ 3. Votre médecin pense qu'un traitement antirétroviral est nécessaire mais vous ne voulez pas le commencer pour l'instant
- ☐ 4. Autre

B29 Autre. Précisez : ____

➔ ***Pour l'enquêteur Allez à B40***

➔ ***POUR CEUX QUI ONT ACTUELLEMENT UN TRAITEMENT ANTIRETROVIRAL PRESCRIT***

B30 Est-ce que vous prenez actuellement le traitement qu'on vous a prescrit ?
-*Une seule réponse possible-*
- ☐ 1. Oui
- ☐ 2. Non

B31 Quand vous a-t-on prescrit votre premier traitement antirétroviral quel qu'il soit (AZT et/ou inhibiteur de protéase) ***-ce n'est pas forcément celui que vous prenez actuellement-*** **?**

/___/___/___/___/ année

Si B30 = 2 ➔ B40 ; Si B30 = 1 ➔ B32

B32 Pour vous, le traitement que vous prenez actuellement contre le VIH/sida est…
-*Une seule réponse possible-*
- ☐ 1. Pas du tout efficace
- ☐ 2. Peu efficace
- ☐ 3. Assez efficace
- ☐ 4. Très efficace

B33 Pour vous, actuellement, les effets désagréables de votre traitement sont…
- ☐ 1. Inexistants
- ☐ 2. Pas du tout gênants
- ☐ 3. Peu gênants
- ☐ 4. Assez gênants
- ☐ 5. Très gênants

B33 bis Depuis que vous prenez votre traitement, avez-vous noté une modification de votre aspect physique ou de votre silhouette ? *–Une seule réponse possible-*

- ☐ $_1$. Oui
- ☐ $_2$. Peut-être
- ☐ $_3$. Non

➔ ***Pour l'enquêteur***
- ☐ $_{98}$. Ne sait pas
- ☐ $_{99}$. Refus de répondre

B33 ter Au cours des 4 dernières semaines, avez-vous ressenti des difficultés ou des troubles de la sexualité?

- ☐ $_1$. Oui, souvent
- ☐ $_2$. Oui, parfois
- ☐ $_3$. Non, jamais

➔ ***Pour l'enquêteur***
- ☐ $_{98}$. Ne sait pas
- ☐ $_{99}$. Refus de répondre

B34 En ce qui concerne les effets secondaires, estimez-vous que votre médecin s'y intéresse…
-Une seule réponse possible-

- ☐ $_1$. Beaucoup
- ☐ $_2$. Assez
- ☐ $_3$. Peu
- ☐ $_4$. Pas du tout

➔ ***Pour l'enquêteur***
- ☐ $_{98}$. Ne sait pas
- ☐ $_{99}$. Refus de répondre

B35 Selon vous, au cours des <u>7 derniers jours</u>, vous avez :
-Une seule réponse possible-

- ☐ $_0$. Scrupuleusement respecté les prescriptions de votre traitement antirétroviral
- ☐ $_1$. Globalement respecté les prescriptions médicales mais avec quelques écarts
- ☐ $_2$. Souvent modifié les prises (rythmes et quantités)
- ☐ $_3$. Pratiquement jamais respecté les prescriptions de votre traitement antirétroviral
- ☐ $_4$. Arrêté tout traitement

B36 On observe qu'il est souvent plus difficile pour les gens de prendre leur traitement en fin de semaine (samedi-dimanche). En ce qui vous concerne, avez-vous manqué un(e) ou plusieurs comprimé(s) ou gélule(s) de votre traitement antiviral lors du <u>dernier week-end</u> ? *-Une seule réponse possible-*

- ☐ $_1$. Oui
- ☐ $_2$. Non

B37 Au cours des <u>7 derniers JOURS</u>, vous est-il arrivé de prendre la dose journalière d'un (ou de plusieurs) des médicaments de votre traitement antiviral en une seule fois ? *-Une seule réponse possible-*

- ☐ $_1$. Oui, plusieurs fois
- ☐ $_2$. Oui, une fois
- ☐ $_3$. Non, jamais

B38 De nombreuses personnes trouvent qu'il est difficile de respecter l'heure à laquelle elles doivent prendre leur(s) médicament(s), soit qu'elles oublient l'heure, soit qu'elles soient obligées de décaler le moment de la prise de plusieurs heures. Au cours de ces <u>7 derniers JOURS</u>, vous est-il arrivé d'oublier ou de décaler de plusieurs heures l'heure de la prise de votre (ou de vos) médicament(s) antiviral/viraux ? - *Une seule réponse possible-*

- ☐ $_1$. Oui, plusieurs fois
- ☐ $_2$. Oui, une fois
- ☐ $_3$. Non, jamais

B39 Dans les années à venir, pensez-vous personnellement que : -*Une seule réponse possible-*

- ☐ $_1$. Vous n'aurez plus besoin d'avoir un traitement pour le VIH (en restant en bonne santé)
- ☐ $_2$. Vous alternerez des périodes avec traitement et des périodes sans traitement
- ☐ $_3$. Vous aurez un traitement toute votre vie (sans interruption)

➔ ***Pour l'enquêteur*** ☐ $_{98}$. Ne sait pas

➔ **POUR TOUS**

B40 Vous est-il déjà arrivé, de la part de médecins ou de soignants, d'être l'objet de…

	Oui	Non
a. Refus de soins	☐ 1	☐ 2
b. Attitudes de rejet (réticences, jugements…)	☐ 1	☐ 2

Si B40 a = 1 OU B40 b = 1 ➔ B41 ;
si B40 a = 2 ET B40 b = 2 ➔ B42

B41 Etait-ce au cours des 12 derniers mois ?
-Une seule réponse possible-
- ☐ 1. Oui
- ☐ 2. Non

B42 Fumez-vous ? *-Une seule réponse possible-*
- ☐ 1. Régulièrement
- ☐ 2. Une cigarette de temps en temps
- ☐ 3. Non

Si B42 = 3 ➔ B43

B42 bis Combien de cigarettes fumez-vous par jour?
|___|___| ***(si moins de 1 par jour, noter 0) ➔ B43***

B42 ter Avez-vous fumé régulièrement dans le passé ?
-Une seule réponse possible-
- ☐ 1. Oui
- ☐ 2. Non

B43 Au cours des 12 derniers mois, combien de fois vous est-il arrivé de consommer de l'alcool ?
-Une seule réponse possible-
- ☐ 8. Jamais ***➔ B50***
- ☐ 1. Une fois par mois ou moins
- ☐ 2. Deux à quatre fois par mois
- ☐ 3. Deux à trois fois par semaine
- ☐ 4. Quatre à six fois par semaine
- ☐ 5. Tous les jours

➔ Pour l'enquêteur
- ☐ 98. Ne sait pas
- ☐ 99. Refus de répondre

Si B43 ≠ 8 ➔ B44
si B43 = 8 ➔ B50

B44 Les jours où vous buvez de l'alcool, combien de verres consommez-vous ? *-Une seule réponse possible-*
- ☐ 1. Un ou deux
- ☐ 2. Trois ou quatre
- ☐ 3. Cinq ou six
- ☐ 4. Sept à neuf
- ☐ 5. Dix ou plus

➔ Pour l'enquêteur
- ☐ 98. Ne sait pas
- ☐ 99. Refus de répondre

B45 Combien de fois vous arrive-t-il de boire 6 verres ou davantage au cours d'une même occasion ? *-Une seule réponse possible-*
- ☐ 8. Jamais
- ☐ 1. Moins d'une fois par mois
- ☐ 2. Une fois par mois
- ☐ 3. Une fois par semaine
- ☐ 4. Tous les jours ou presque

➔ Pour l'enquêteur
- ☐ 98. Ne sait pas
- ☐ 99. Refus de répondre

B46 Au cours des 12 derniers mois, avez-vous déjà ressenti le besoin de diminuer votre consommation de boissons alcoolisées ? *-Une seule réponse possible-*

☐ 1. Oui
☐ 2. Non

B47 Au cours des 12 derniers mois, votre entourage vous a –t-il déjà fait des remarques au sujet de votre consommation ? *-Une seule réponse possible-*

☐ 1. Oui
☐ 2. Non

B48 Au cours des 12 derniers mois, avez-vous déjà eu l'impression que vous buviez trop ? *-Une seule réponse possible-*

☐ 1. Oui
☐ 2. Non

B49 Au cours des 12 derniers mois, avez-vous déjà eu besoin d'alcool dès le matin pour vous sentir en forme ? *-Une seule réponse possible-*

☐ 1. Oui
☐ 2. Non

B50 Au cours des 12 derniers mois, avez-vous pris des anti-dépresseurs ? (par exemple, Prozac®, Athymil®, Anaframil®, Laroxyl®)

☐ 1. Oui
☐ 2. Non

➔ Pour l'enquêteur

☐ 98. Ne sait pas
☐ 99. Refus de répondre

B51 Au cours des 12 derniers mois, avez-vous pris des tranquillisants ou des somnifères ? (par exemple, Lexomil®, Lysanxia®,Temesta ®, Xanas®, tranxène®, Stilnox®, Imovanne®, Mogadon®)

☐ 1. Oui
☐ 2. Non

➔ Pour l'enquêteur

☐ 98. Ne sait pas
☐ 99. Refus de répondre

Si B51 = 1 ➔ B51 bis ; si B51 = 2 ➔ B52

B51 bis <u>Au cours des 30 derniers jours (du dernier mois)</u>, combien de fois avez-vous pris des tranquillisants ou des somnifères ?

☐ 1. Jamais
☐ 2. Moins d'une fois par semaine
☐ 3. Une fois par semaine
☐ 4. Plusieurs fois par semaine
☐ 5. Tous les jours ou presque

➔ Pour l'enquêteur

☐ 98. Ne sait pas
☐ 99. Refus de répondre

B52 Au cours des 12 derniers mois, avez-vous <u>utilisé</u> l'un des produits suivants ?

	**Jamais**	**Parfois**	**Régulièrement**	**Tous les jours ou presque**
a. Cannabis, haschich	☐ 0	☐ 1	☐ 2	☐ 3
b. Ecstasy, etc.	☐ 0	☐ 1	☐ 2	☐ 3
c. Héroïne	☐ 0	☐ 1	☐ 2	☐ 3
d. Cocaïne ou Crack (caillou)	☐ 0	☐ 1	☐ 2	☐ 3
e. Subutex / Temgesic	☐ 0	☐ 1	☐ 2	☐ 3
f. Méthadone	☐ 0	☐ 1	☐ 2	☐ 3
g. Moscontin / Skénan	☐ 0	☐ 1	☐ 2	☐ 3
h. Néocodion ou autre codéine	☐ 0	☐ 1	☐ 2	☐ 3
i. Rohypnols	☐ 0	☐ 1	☐ 2	☐ 3
j. Amphétamines	☐ 0	☐ 1	☐ 2	☐ 3
k. LSD et autres hallucinogènes (Kétamine)	☐ 0	☐ 1	☐ 2	☐ 3
l. Poppers	☐ 0	☐ 1	☐ 2	☐ 3

Si B52 c ET B52 d ET B52 e ET B52 f ET B52 g ET B52 h ET B52 i ET B52 k = 0 {pas de consommation de produits pouvant s'injecter} ➔ B53
Si B52 c OU B52 d OU B52 e OU B52 f OU B52 g OU B52 h OU B52 i OU B52 k ≠ 0 {consommation de produits pouvant s'injecter} ➔ B52 bis

B52 bis Vous êtes-vous injecté(e) l'un de ces produits au cours des 12 derniers mois ?
-*Une seule réponse possible*-
- ☐ 1. Oui
- ☐ 2. Non

Si B52 e OU B52 f OU B52 g ≠ 0 {consommation de produits de substitution} ➔ B52 ter ;
si B52 e ET B52 f ET B52 g = 0 {pas de consommation de produits de substitution} ➔ B53

B52 ter Etait-ce dans le cadre d'un traitement de substitution prescrit par un médecin ?
- ☐ 1. Oui
- ☐ 2. Non
- ☐ 3. Parfois oui, parfois non

B53 Avez-vous déjà sniffé des produits à l'aide d'une paille ou de tout instrument du même type ?
-*Une seule réponse possible*-
- ☐ 1. Oui, au cours des 12 derniers mois
- ☐ 2. Oui, mais il y a plus longtemps
- ☐ 3. Non

B54 Etes-vous ou avez-vous été positif pour le virus de l'hépatite C ? -*Une seule réponse possible*-
- ☐ 1. Oui
- ☐ 2. Non ➔ ***B58***

➔ Pour l'enquêteur
- ☐ 98. Ne sait pas

Si B54 = 2 ➔ B58

B55 Etes-vous suivi(e) pour l'hépatite C ? -*Une seule réponse possible*-
- ☐ 1. Oui, dans le service où l'on vous suit pour le VIH
- ☐ 2. Oui dans un autre service du même hôpital
- ☐ 3. Oui dans un autre hôpital
- ☐ 4. Oui, en ville par un spécialiste
- ☐ 5. Oui, en ville par un médecin généraliste
- ☐ 6. Non, pas du tout

B56 Avez-vous déjà subi une biopsie du foie ? -*Une seule réponse possible*-
- ☐ 1. Oui
- ☐ 2. Non

➔ Pour l'enquêteur
- ☐ 98. Ne sait pas

B57 Avez-vous actuellement un traitement contre le virus de l'hépatite C ? -*Une seule réponse possible*-
- ☐ 1. Oui
- ☐ 2. Non, votre traitement est terminé
- ☐ 3. Non, votre traitement a été arrêté à cause des effets secondaires
- ☐ 4. Non, votre traitement a été arrêté pour d'autres causes
- ☐ 5. Non, vous n'en avez jamais eu

B58 Comment voyez-vous la gravité de l'hépatite C par rapport au VIH ? -*Une seule réponse possible*-
- ☐ 1. L'hépatite C est plus grave que le VIH
- ☐ 2. L'hépatite C est aussi grave que le VIH
- ☐ 3. L'hépatite C est moins grave que le VIH

➔ Pour l'enquêteur
- ☐ 98. Ne sait pas

B59 Au cours des 12 derniers mois, avez-vous contracté une maladie qui se transmet sexuellement (chaude-pisse, gonorrhée, syphilis, condylome) ? *-Une seule réponse possible-*

☐ 1. Oui
☐ 2. Non

➔ ***Pour l'enquêteur***
☐ 98. Ne sait pas

B60 Au cours de votre vie, avez-vous déjà fait une tentative de suicide ? *-Une seule réponse possible-*

☐ 1. Oui, une fois
☐ 2. Oui, plusieurs fois
☐ 3. Non ⟶ ***Module C***

B60 bis Quand avez-vous fait cette tentative de suicide *(<u>ou la dernière</u> s'il y en a eu plusieurs)?*
-Une seule réponse possible-

☐ 1. Au cours des 12 derniers mois
☐ 2. Il y a entre 1 an et 5 ans
☐ 3. Plus de 5 ans

Module C « Insertion professionnelle »

➔ ***A TOUS***

NOUS ALLONS PARLER MAINTENANT DE VOTRE SITUATION AU REGARD DE L'EMPLOI.

C0 Jusqu'à aujourd'hui, comment s'est déroulée principalement votre vie professionnelle ?
-Une seule réponse possible-

- ☐ 1. Vous n'avez jamais cessé de travailler (éventuellement jusqu'à cessation pour la retraite)
- ☐ 2. Vous avez travaillé régulièrement, pratiquement sans interruption, avant d'être au chômage ou de cesser de travailler
- ☐ 3. Vous avez alterné des périodes où vous avez travaillé et des périodes où vous n'avez pas travaillé
- ☐ 4. Vous avez eu principalement des petits boulots
- ☐ 5. Vous n'avez jamais travaillé
- ☐ 6. Autre. **C0 Autre**. Précisez : ____

Si B13 a ≠ 1 {y compris ceux qui travaillaient de façon non déclarée} ➔ C10
Si B13 a = 1 {travaillait de façon déclarée} ➔ C1

➔ ***POUR CEUX QUI AVAIENT UN EMPLOI DECLARE QUAND ILS ONT APPRIS QU'ILS ETAIENT SEROPOSITIFS (B13 A = 1)***

NOUS ALLONS PARLER DE VOTRE EMPLOI AU MOMENT OU VOUS AVEZ DECOUVERT VOTRE INFECTION A VIH.

C1 Depuis quand occupiez-vous cet emploi ?

/___/___/___/___/ Année /___/___/ mois

C2 Quelle était alors votre profession principale (en clair) **?**
Consigne à l'enquêteur ➔ récolter l'information la plus précise possible

____

C3 Cette profession était exercée en qualité de… *-Une seule réponse possible-*

- ☐ 1. Salarié de l'Etat
- ☐ 2. Salarié d'une collectivité territoriale
- ☐ 3. Salarié du secteur hospitalier public
- ☐ 4. Salarié d'une entreprise publique ou nationalisée (EDF, SNCF)
- ☐ 5. Salarié du secteur privé à but non lucratif
- ☐ 6. Salarié d'une entreprise privée
- ☐ 7. Employé de maison, emploi familial chez un ou plusieurs employeurs
- ☐ 8. Salarié de votre propre entreprise ou salarié de votre conjoint(e)
- ☐ 9. Non salarié, aide familial(e)
- ☐ 10. Commerçant
- ☐ 11. Profession libérale, travailleur indépendant, free-lance, etc. ➔ ***C5*** (items 9 à 98)
- ☐ 12. Intermittent du spectacle
- ☐ 13. Autre
- ☐ 98. Ne sait pas

Si C3 ≥ 9 ➔ C5

C4 Quelle était la nature de votre contrat de travail ? *-Une seule réponse possible-*
- ☐ 1. Contrat à Durée Indéterminée (CDI) OU titulaire de la fonction publique
- ☐ 2. Contrat à Durée Déterminée (CDD)
- ☐ 3. Contrat aidé (CES, emploi jeune, CQ, CEC…)
- ☐ 4. Intérim ou vacations
- ☐ 5. Apprentissage
- ☐ 6. Stage
- ☐ 7. Travail saisonnier
- ☐ 8. Autre. Précisez : ___________________________________

C5 Quel était le montant de votre salaire (ou de votre revenu) mensuel net (➔ *Pour l'enquêteur : avant impôt*) ?

____

➔ Pour l'enquêteur :

Emploi exercé à l'étranger	99996
Smic	99997
Nsp	99998
Refus de réponse	99999

Si C5 = (99998,99999) ➔ C5 bis

C5 bis Mais, environ, quel était votre salaire (revenu) net ? *-Une seule réponse possible-*

☐ 1. Moins de 2500 francs	Moins de 380 euros
☐ 2. De 2500 FF à moins de 4000 FF	De 380 à moins de 610 euros
☐ 3 De 4000 FF à moins de 5000 FF	De 610 à moins de 760 euros
☐ 4. De 5000 FF à moins de 7000 FF	De 760 à moins de 1070 euros
☐ 5. De 7000 FF à moins de 9000 FF	De 1070 à moins de 1370 euros
☐ 6. De 9000 FF moins de 12000 FF	De 1370 à moins de 1830 euros
☐ 7. De 12 000 FF à moins de 15 000 FF	De 1830 à moins de 2290 euros
☐ 8. De 15 000 FF à moins de 20 000 FF	De 2290 à moins de 3050 euros
☐ 9. De 20 000 FF à moins de 30 000 FF	De 3050 à moins de 4570 euros
☐ 10. 30 000 FF ou plus	4570 euros ou plus

➔ Pour l'enquêteur
- ☐ 98. Ne sait pas
- ☐ 99. Refus de réponse

C6 Avez-vous toujours le même emploi / la même activité ? -*Une seule réponse possible-*
- ☐ 1. Oui ➔ ***C15***
- ☐ 2. Non

Si C6 = 1 ➔ C15

C7 A quelle date cet emploi / activité s'est-t-il terminé ?

/___/___/___/___/ Année /___/___/ mois

C8 Pour quelle raison cet emploi / activité s'est-il terminé ? -*Une seule réponse possible-*
- ☐ 1. Vous avez démissionné pour raison de santé*
- ☐ 2. Vous avez démissionné pour une autre raison*
- ☐ 3. C'était la fin de votre contrat
- ☐ 4. Vous avez été licencié(e)
- ☐ 5. Vous avez pris votre retraite / pré-retraite
- ☐ 6. L'entreprise que vous dirigiez a fait faillite

**** Consigne à l'enquêteur : si raison réelle ≠ raison officielle ➔ cocher la raison réelle***

Si C8 ≠ 4 ➔ C9

C8 bis Pour quelle raison avez-vous été licencié(e) ? -*Une seule réponse possible-*
- ☐ 1. Pour raison économique ➔ ***C9***
- ☐ 2. Pour d'autres raisons (mésentente, faute, etc.) ➔ ***C9***

C9 Diriez-vous que votre infection à VIH a joué un rôle dans la rupture de votre contrat de travail ou dans l'arrêt de votre activité ?
-Une seule réponse possible-

- ☐ 1. Beaucoup
- ☐ 2. Assez
- ☐ 3. Un peu
- ☐ 4. Pas du tout

➔ ***Pour l'enquêteur :***
- ☐ 98. Ne sait pas
- ☐ 99. Refus de réponse

➔ <u>A TOUS</u> (SAUF CEUX QUI ONT ACTUELLEMENT LE MEME EMPLOI DECLARE QU'AU MOMENT DE L'ANNONCE DU DISGNOSTIC SEROLOGIQUE)

C10 Actuellement… *-Une seule réponse possible-*

- ☐ 1. Vous avez un emploi / activité déclaré(e)
- ☐ 2. Vous êtes au chômage
- ☐ 3. Vous êtes retraité / pré-retraité
- ☐ 4. Vous n'avez aucune activité professionnelle

➔ ***Pour l'enquêteur :***
- ☐ 98. Ne sait pas
- ☐ 99. Refus de réponse

Si C10 = 1 ➔ C11 ; Si C10 > 1 ➔ C26

C11 Depuis quand occupez-vous cet emploi ?
/___/___/___/___/ Année /___/___/ mois (***Enquêteur ➔ insister pour avoir le mois si la date est récente***)

C12 Quelle est votre profession principale (en clair) **?**
Consigne à l'enquêteur ➔ récolter l'information la plus précise possible

____

C13 Exercez-vous cette profession comme… *-Une seule réponse possible-*

- ☐ 1. Salarié de l'Etat
- ☐ 2. Salarié d'une collectivité territoriale
- ☐ 3. Salarié du secteur hospitalier public
- ☐ 4. Salarié d'une entreprise publique ou nationalisée (EDF, SNCF)
- ☐ 5. Salarié du secteur privé à but non lucratif
- ☐ 6. Salarié d'une entreprise privée
- ☐ 7. Employé de maison, emploi familial chez un ou plusieurs employeurs
- ☐ 8. Salarié de votre propre entreprise ou salarié de votre conjoint(e)
- ☐ 9. Non salarié, aide familial(e)
- ☐ 10. Commerçant
- ☐ 11. Profession libérale, travailleur indépendant, free-lance, etc.
- ☐ 12. Intermittent du spectacle
- ☐ 13. Autre
- ☐ 98. Ne sait pas

(9 à 98) → ***C15***

Si C13 ≥ 9 ➔ C15

C14 Quelle est la nature de votre contrat de travail ? *-Une seule réponse possible-*

- ☐ 1. Contrat à Durée Indéterminée (CDI) OU titulaire de la fonction publique
- ☐ 2. Contrat à Durée Déterminée (CDD)
- ☐ 3. Contrat aidé (CES, emploi jeune, CQ, CEC…)
- ☐ 4. Intérim ou vacations
- ☐ 5. Apprentissage
- ☐ 6. Stage
- ☐ 7. Travail saisonnier
- ☐ 8. Autre. **C14 Autre.** Précisez : ____

C15 Aujourd'hui, êtes-vous en arrêt-maladie ? -*Une seule réponse possible***-**
☐ 1. Oui
☐ 2. Non ⟶

C15 bis Depuis quelle date ? (*Date de votre dernier mois de travail)*

/___/___/___/___/ Année /___/___/ mois (***Insister pour avoir le mois si date récente***)

Si C6 = 1 {même emploi qu'au moment du diagnostic} ET si (C15 = 2 {pas en arrêt de travail} OU C15 bis ≤ 6 mois {en arrêt de travail inférieur ou égal à 6 mois}) ➔ C16

Si [(C10 = 1 ET B13 a ≠ 1) OU (C10 = 1 ET C6 = 2)] ET (C15 = 2 {pas en arrêt de travail} OU C15 bis ≤ 6 mois {en arrêt de travail inférieur ou égal à 6 mois}) ➔ C19 {salaire}

Si C15 bis > 6 mois ➔ C23

C16 Dans votre emploi / activité, par rapport à l'époque où vous avez appris votre infection à VIH, vous avez eu une promotion professionnelle ? -*Une seule réponse possible***-**
☐ 1. Oui
☐ 2. Non

C17 Dans votre emploi / activité, par rapport à l'époque où vous avez appris votre infection à VIH, votre statut est devenu… -*Une seule réponse possible***-**
☐ 1. Plus stable
☐ 2. Plus précaire
☐ 3. N'a pas changé

C18 Compte-tenu du coût de la vie, par rapport à l'époque où vous avez appris votre infection à VIH, votre salaire / revenu… -*Une seule réponse possible***-**
☐ 1. a augmenté
☐ 2. a baissé
☐ 3. est resté stable

Si C18 = 2 ➔ C18 bis

C18 bis Diriez-vous que votre infection à VIH a joué un rôle dans la diminution de vos revenus ?
-*Une seule réponse possible*-
☐ 1. Beaucoup
☐ 2. Assez
☐ 3. Un peu
☐ 4. Pas du tout

➔ Pour l'enquêteur :
☐ 98. Ne sait pas
☐ 99. Refus de réponse

C19 Aujourd'hui quel est le montant de votre salaire (ou de votre revenu) mensuel net (➔ *Pour l'enquêteur : avant impôt*) **?**
➔ *Consigne à l'enquêteur* :
- ***si difficulté (exemple travailleur libéral), rappeler qu'il s'agit d'une moyenne sur l'année :*** **A peu près, sur l'année, vous avez gagné combien par mois** ?
- *si personne en arrêt-maladie de moins de 6 mois : salaire du mois précédent l'arrêt maladie*

/____/____/____/____/____/ euros/francs

➔ Pour l'enquêteur :

Smic	99997
Nsp	99998
Refus de réponse	99999

Si C19 = (99998,99999) ➔ C19 bis ; si C19 ≠ 99998, 99999 ➔ C20

C19 bis Mais, environ, quel est votre salaire net ? -*Une seule réponse possible***-**

☐ 1. Moins de 2500 francs	Moins de 380 euros	➔ *Pour l'enquêteur*
☐ 2. De 2500 FF à moins de 4000 FF	De 380 à moins de 610 euros	☐ 98. Ne sait pas
☐ 3 De 4000 FF à moins de 5000 FF	De 610 à moins de 760 euros	☐ 99 Refus de réponse
☐ 4. De 5000 FF à moins de 7000 FF	De 760 à moins de 1070 euros	
☐ 5. De 7000 FF à moins de 9000 FF	De 1070 à moins de 1370 euros	
☐ 6. De 9000 FF moins de 12000 FF	De 1370 à moins de 1830 euros	
☐ 7. De 12 000 FF à moins de 15 000 FF	De 1830 à moins de 2290 euros	
☐ 8. De 15 000 FF à moins de 20 000 FF	De 2290 à moins de 3050 euros	
☐ 9. De 20 000 FF à moins de 30 000 FF	De 3050 à moins de 4570 euros	
☐ 10. 30 000 FF ou plus	4570 euros ou plus	

C20 Combien d'heures par semaine travaillez-vous habituellement -*hors trajet domicile/travail*- ?
➔ ***Consigne à l'enquêteur*** **:**
- ***si n'arrive pas à répondre, insister :***
  A peu près, sur l'année, vous avez travaillé combien d'heures par semaine ?
- ***si personne en arrêt-maladie de moins de 6 mois, nombre d'heures travaillées avant l'arrêt maladie***

|___|___| heures ➔ ***Pour l'enquêteur :*** ☐ 98 Ne sait pas
☐ 00. Ne peut pas répondre (très variable) ☐ 99 Refus de réponse

C21 En général, combien de temps mettez-vous pour vous déplacer entre votre domicile et votre lieu de travail ? (aller + retour)
➔ ***Consigne à l'enquêteur : si personne en arrêt-maladie, temps de déplacement avant l'arrêt maladie***

|___|___| minutes ou |___|___| en heures
☐ 00. Sans objet : travail à domicile, travail itinérant, VRP, etc.

C22 Actuellement…–*Plusieurs réponses possibles-*
➔ ***Consigne à l'enquêteur : si personne en arrêt-maladie, ressenti du travail avant l'arrêt maladie***

	**Très**	**Un peu**	**Pas du tout**
a. Vous trouvez votre travail pénible physiquement	☐ 1	☐ 2	☐ 3
b. Vous trouvez votre travail pénible psychologiquement	☐ 1	☐ 2	☐ 3
c. Votre infection à VIH rend le travail plus difficile	☐ 1	☐ 2	☐ 3
d. Vos horaires de travail vous mettent en difficulté pour prendre votre traitement	☐ 1	☐ 2	☐ 3
e. Vos horaires de travail vous mettent en difficulté pour vous faire suivre à l'hôpital pour le VIH	☐ 1	☐ 2	☐ 3

C23 Avez-vous vous-même parlé de votre infection à VIH sur votre lieu de travail ou avec des relations de travail ? -*Une seule réponse possible-*
➔ ***Consigne à l'enquêteur : avant l'arrêt de travail pour ceux dans ce cas***
☐ 1. Oui ➔ ***Pour l'enquêteur***
☐ 2. Non ➔ C***23 ter*** ☐ 3. Non concerné

Si C23 = 2 ➔ C23 ter

**C23 bis A quelle(s) personne(s) ?**	**Oui**	**Non**
a. Certains de vos collègues qui sont des amis	☐ 1	☐ 2
b. D'autres collègues	☐ 1	☐ 2
c. Certains de vos supérieurs hiérarchiques	☐ 1	☐ 2
d. Un médecin du travail	☐ 1	☐ 2
e. Un représentant du personnel	☐ 1	☐ 2
f. Autre	☐ 1	☐ 2

C23 bis Autre. Précisez : ____

C23 ter Certaines personnes au travail sont-elles néanmoins au courant sans que vous le leur ayez dit ?
-Une seule réponse possible-

☐ 1. Oui
☐ 2. Non

➔ ***Pour l'enquêteur :***
☐ 98. Ne sait pas

C24 Votre emploi a-t-il été adapté du fait de votre infection à VIH ? *-Une seule réponse possible-*
➔ ***Consigne à l'enquêteur : avant l'arrêt de travail pour ceux dans ce cas***

☐ 1. Oui
☐ 2. Non → ***C25***

Si C24= 2 ➔ C25

C24 bis Vous avez…*–Plusieurs réponses possibles-*

	Oui	Non
a. un poste ou des horaires aménagés	☐ 1	☐ 2
b. un temps partiel thérapeutique	☐ 1	☐ 2
c. une affectation à un poste plus adapté à votre état de santé	☐ 1	☐ 2

C25 Envisagez-vous de changer d'emploi ou de travail dans un avenir proche ?
-Une seule réponse possible-

☐ 1. Oui
☐ 2. Non → ***C41***

Si C25 = 2 ➔ C41

C25 bis Est-ce…*–Plusieurs réponses possibles-*

	Oui	Non
a. parce que vous allez mieux	☐ 1	☐ 2
b. parce que votre santé s'est dégradée	☐ 1	☐ 2
c. pour préserver votre santé	☐ 1	☐ 2
d. en raison de tensions dans votre milieu professionnel liées directement ou indirectement à votre infection à VIH	☐ 1	☐ 2
e. pour avoir un emploi plus compatible avec votre vie familiale	☐ 1	☐ 2
f. pour avoir un emploi plus intéressant	☐ 1	☐ 2
g. pour une autre raison	☐ 1	☐ 2

C25 ter <u>Depuis 3 mois</u> avez-vous effectué des démarches dans ce sens (préparation ou inscription à des formations, examens, concours) ?
-Une seule réponse possible-

☐ 1. Oui
☐ 2. Non

➔ ***Pour l'enquêteur***
☐ 98. Ne sait pas
☐ 99. Refus de répondre
→ ***C41***

➔ *POUR CEUX QUI N'ONT PAS D'EMPLOI (DECLARE) ACTUELLEMENT (C10 > 1)*

NOUS ALLONS PARLER DE VOTRE DERNIERE ACTIVITE PROFESSIONNELLE (DECLAREE).

C26 Quand avez-vous cessé votre <u>dernière</u> activité déclarée ?

|___|___|___|___| année |___|___| mois (***Insister pour avoir le mois si année récente***)

☐ 00. N'a jamais eu d'activité déclarée

Si C26 = C7 (si la date de cessation de la dernière activité correspond à la date de cessation de l'emploi occupé au moment du diagnostic sérologique) OU si C26 = 00 (n'a jamais eu d'activité professionnelle) ➔ C32
Si C26 ≠ C7 (les dates ne correspondent pas) ➔ C27

C27 Quelle était votre profession principale (en clair) **?**
Consigne à l'enquêteur ➔ récolter l'information la plus précise possible

____

C28 Vous exercez cette profession comme… -*Une seule réponse possible*-

- ☐ 1. Salarié de l'Etat
- ☐ 2. Salarié d'une collectivité territoriale
- ☐ 3. Salarié du secteur hospitalier public
- ☐ 4. Salarié d'une entreprise publique ou nationalisée (EDF, SNCF)
- ☐ 5. Salarié du secteur privé à but non lucratif
- ☐ 6. Salarié d'une entreprise privée
- ☐ 7. Employé de maison, emploi familial chez un ou plusieurs employeurs
- ☐ 8. Salarié de votre propre entreprise ou salarié de votre conjoint(e)
- ☐ 9. Non salarié, aide familial(e)
- ☐ 10. Commerçant
- ☐ 11. Profession libérale, travailleur indépendant, free-lance, etc. → ***C29***
- ☐ 12. Intermittent du spectacle
- ☐ 13. Autre
- ☐ 98. Ne sait pas

Si C28 ≥ 9 ➔ C29

C28 bis Quelle était la nature de votre contrat de travail ? -*Une seule réponse possible*-

- ☐ 1. Contrat à Durée Indéterminée (CDI) OU titulaire de la fonction publique
- ☐ 2. Contrat à Durée Déterminée (CDD)
- ☐ 3. Contrat aidé (CES, emploi jeune, CQ, CEC…)
- ☐ 4. Intérim ou vacations
- ☐ 5. Apprentissage
- ☐ 6. Stage
- ☐ 7. Travail saisonnier
- ☐ 8. Autre. Précisez : ____________________________________

C29 Quel était le montant de votre salaire (ou de votre revenu) mensuel net (➔ *Pour l'enquêteur : avant impôt*) ?

/____/____/____/____/____/ euros / francs

➔ Pour l'enquêteur

Emploi exercé à l'étranger	99996
Ne sait pas	99998
Smic	99997
Refus de réponse	99999

Si C29 = (99998,99999) ➔ C29 bis

C29 bis Mais, environ, quel était votre salaire net ? -*Une seule réponse possible*-

☐ 1. Moins de 2500 francs	Moins de 380 euros
☐ 2. De 2500 FF à moins de 4000 FF	De 380 à moins de 610 euros
☐ 3. De 4000 FF à moins de 5000 FF	De 610 à moins de 760 euros
☐ 4. De 5000 FF à moins de 7000 FF	De 760 à moins de 1070 euros
☐ 5. De 7000 FF à moins de 9000 FF	De 1070 à moins de 1370 euros
☐ 6. De 9000 FF moins de 12000 FF	De 1370 à moins de 1830 euros
☐ 7. De 12 000 FF à moins de 15 000 FF	De 1830 à moins de 2290 euros
☐ 8. De 15 000 FF à moins de 20 000 FF	De 2290 à moins de 3050 euros
☐ 9. De 20 000 FF à moins de 30 000 FF	De 3050 à moins de 4570 euros
☐ 10. 30 000 FF ou plus	4570 euros ou plus

➔ Pour l'enquêteur

- ☐ 98. Ne sait pas
- ☐ 99. Refus de réponse

C30 Pour quelle raison cet emploi s'est-il terminé ? -*Une seule réponse possible*-

- ☐ 1. Vous avez démissionné pour raison de santé*
- ☐ 2. Vous avez démissionné pour une autre raison*
- ☐ 3. C'était la fin de votre contrat
- ☐ 4. Vous avez été licencié(e)
- ☐ 5. Vous avez pris votre retraite / pré-retraite
- ☐ 6. L'entreprise que vous dirigiez a fait faillite

**** <u>Consigne à l'enquêteur</u> : si raison réelle ≠ raison officielle ➔ cocher la raison réelle***

Si C30 ≠ 4 ➔ C31

C30 bis Pour quelle raison avez-vous été licencié(e) ? *-Une seule réponse possible-*
- ☐ 1. Pour raison économique
- ☐ 2. Pour d'autres raisons (mésentente, faute, etc.)

C31 Diriez-vous que votre infection à VIH a joué un rôle dans la rupture de votre contrat de travail ou dans l'arrêt de votre activité ? *-Une seule réponse possible-*
- ☐ 1. Beaucoup
- ☐ 2. Assez
- ☐ 3. Un peu
- ☐ 4. Pas du tout

➔ Pour l'enquêteur :
- ☐ 98. Ne sait pas
- ☐ 99. Refus de réponse

C32 Aujourd'hui envisagez-vous de travailler ou de retravailler (de façon déclarée) ? *–Une seule réponse possible-*
- ☐ 1. Oui ***➔ C36 > C40***
- ☐ 2. Non ***➔ C33 > C35 bis***

➔ Pour l'enquêteur :
- ☐ 3. Non concerné(e) (à la retraite ou pré-retraite) ***➔ C42***

Si C32 = 1 ➔ C36 ; si C32 = 3 ➔ C42

➔ POUR CEUX QUI N'ENVISAGENT PAS DE (RE)TRAVAILLER (C32 = 2)

C33 Si vous n'envisagez pas de travailler, est-ce à cause de votre infection à VIH ? *-Une seule réponse possible-*
- ☐ 1. Oui
- ☐ 2. En partie
- ☐ 3. Non ***➔ C35***

**C34 Vous n'envisagez pas de travailler…** *–Plusieurs réponses possibles-*	**Oui**	**Non**
a. A cause des effets secondaires du traitement	☐ 1	☐ 2
b. Parce que vous êtes souvent malade	☐ 1	☐ 2
c. Parce que vous avez peur de ne pas pouvoir tenir un emploi	☐ 1	☐ 2
d. Parce que vous avez peur des risques de discrimination	☐ 1	☐ 2
e. Parce que vous avez un/des enfant(s) en bas âge	☐ 1	☐ 2
f. Parce que vous n'avez pas de papiers	☐ 1	☐ 2
g. Parce que vous avez peur de perdre vos allocations actuelles	☐ 1	☐ 2

C35 Pensez-vous que vos ressources diminueraient si vous repreniez un travail ? *-Une seule réponse possible-*
- ☐ 1. Oui
- ☐ 2. Non

➔ Pour l'enquêteur
- ☐ 98. Ne sait pas
- ☐ 99. Refus de répondre

C35 bis Quel salaire net mensuel serait susceptible de vous faire (re)travailler ?

____

➔ Pour l'enquêteur
- ☐ 98. Ne sait pas
- ☐ 99. Refus de répondre

➔ ***C41***

➔ ***POUR LES PERSONNES QUI ENVISAGENT DE (RE)TRAVAILLER***

C36 Vous envisagez de travailler parce que…
–Plusieurs réponses possibles-

	Oui	Non
a. Votre santé s'est améliorée	☐ 1	☐ 2
b. Vous avez perdu l'AAH	☐ 1	☐ 2
c. Vos revenus sont insuffisants	☐ 1	☐ 2
d. Vous en avez besoin psychologiquement	☐ 1	☐ 2
e. Vous avez trouvé un mode de garde pour votre plus jeune enfant	☐ 1	☐ 2
f. Votre/vos enfant(s) sont assez grands maintenant	☐ 1	☐ 2
g. Vous voulez vous réinsérer dans le vie active	☐ 1	☐ 2

C37 Depuis 3 mois, avez-vous fait des démarches pour trouver un emploi ?
-Une seule réponse possible-
☐ 1. Oui
☐ 2. Non

➔ ***Pour l'enquêteur***
☐ 98. Ne sait pas
☐ 99. Refus de répondre

Si C37 = 2 ➔ C40 ; Si C37 = 1 ➔ C38

C38 Quelles difficultés rencontrez-vous dans la recherche d'un emploi ?
–Plusieurs réponses possibles-

	Oui	Non
a. Les emplois qu'on vous propose sont inadaptés à votre maladie	☐ 1	☐ 2
b. Vous êtes resté(e) trop longtemps sans travailler	☐ 1	☐ 2
c. Vous avez peur des risques de discrimination	☐ 1	☐ 2
d. Vous manquez de qualification	☐ 1	☐ 2
e. Vous avez des problèmes de langue, de papier parce que vous êtes étranger/ère	☐ 1	☐ 2
f. Cela entraîne des dépenses importantes pour vous (coût de la correspondance, téléphone, Minitel, magazines spécialisés, transports, etc.)	☐ 1	☐ 2
g. Un problème de transport (trajets trop longs, pas de voiture, etc.)	☐ 1	☐ 2
h. Vous ne trouver pas de solution pour faire garder votre/vos enfant(s)	☐ 1	☐ 2
i. Autres	☐ 1	☐ 2

C39 Etes-vous aidé(e) dans vos démarches ? *–Plusieurs réponses possibles-*

	Oui	Non
a. Par des relations personnelles (parents, amis)	☐ 1	☐ 2
b. Par l'ANPE, l'APEC, mission locale, etc.	☐ 1	☐ 2
c. Par la COTOREP, l'AGEFIPH, Cap emploi	☐ 1	☐ 2
d. Par une association de lutte contre le sida	☐ 1	☐ 2
e. Par un autre service social	☐ 1	☐ 2

C40 Quel est le plus petit salaire mensuel (*➔ pour l'enquêteur, à plein temps*) que vous seriez prêt(e) à accepter ?
/___/___/___/___/___/___/ FF ou euros

➔***Pour l'enquêteur***
☐ 98. Ne sait pas
☐ 99. Refus de répondre

➔ *A TOUS*

C41 Bénéficiez-vous de la RQTH (Reconnaissance de la Qualité de Travailleur Handicapé) ?
➔ Consigne à l'enquêteur : Ne pas confondre avec l'AAH
-Une seule réponse possible-

☐ 1. Oui
☐ 2. Non

➔ Pour l'enquêteur
☐ 98. Ne sait pas

C41 bis Dans quelle catégorie êtes-vous ? *-Une seule réponse possible-*

☐ 1. A
☐ 2. B
☐ 3. C

➔ Pour l'enquêteur
☐ 98. Ne sait pas

C42 Que vous ayez un emploi déclaré ou que vous n'en ayez pas (chômage, retraite, sans activité) avez-vous un emploi/activité non déclaré ? *-Une seule réponse possible-*

☐ 1. Oui
☐ 2. Non ***➔ Module D***

Si C42 = 2 ➔ Module D ; Si C42 = 1 ➔ C43

➔ ***POUR CEUX QUI ONT ACTUELLEMENT UN EMPLOI NON-DECLARE***

C43 Au cours des 12 derniers mois, vous avez exercé cet emploi pendant : *-une seule réponse possible-*

☐ 1. Moins de 3 mois
☐ 2. De 3 à 6 mois
☐ 3. De 6 à 12 mois
☐ 4. Tout le temps

C44 Durant les périodes où vous avez travaillé, cela a représenté : *-une seule réponse possible-*

☐ 1. Plus qu'un temps plein normal
☐ 2. Comme un temps plein
☐ 3. Comme un mi-temps
☐ 4. Moins qu'un mi-temps

C45 Cet emploi se situe : *-une seule réponse possible*

☐ 1. Dans le commerce
☐ 2. Chez des particuliers comme employé de maison, jardinier, baby-sitter, etc.
☐ 3. Dans la restauration, l'hôtellerie
☐ 4. Sur des chantiers (peinture, bâtiment, etc.)
☐ 5. Dans le domaine artistique ou intellectuel
☐ 6. Dans un autre domaine

Module D Ressources et conditions de vie

➔ *A TOUS*

D1 En considérant l'ensemble des revenus de toutes les personnes qui vivent actuellement dans le même logement que vous (*salaires ou revenus y compris du conjoint, allocations*), quel est le montant mensuel des revenus nets dont votre ménage dispose ?

/___/___/___/___/___/___/___/ FF ou euros ➔ ***D2***

Smic	99997
Nsp	99998
Refus de réponse	99999

Si D1 = 99998, 99999 ➔ D1bis

D1bis Si vous ne pouvez pas donner un montant précis, pouvez-vous au moins en donner une estimation ?

-Une seule réponse possible-

☐ 1. Moins de 2500 francs	Moins de 380 euros	
☐ 2. De 2500 FF à moins de 4000 FF	De 380 à moins de 610 euros	➔ ***Pour l'enquêteur***
☐ 3. De 4000 FF à moins de 5000 FF	De 610 à moins de 760 euros	☐ 98. Ne sait pas
☐ 4. De 5000 FF à moins de 7000 FF	De 760 à moins de 1070 euros	☐ 99. Refus de répondre
☐ 5. De 7000 FF à moins de 9000 FF	De 1070 à moins de 1370 euros	
☐ 6. De 9000 FF moins de 12000 FF	De 1370 à moins de 1830 euros	
☐ 7. De 12 000 FF à moins de 15 000 FF	De 1830 à moins de 2290 euros	
☐ 8. De 15 000 FF à moins de 20 000 FF	De 2290 à moins de 3050 euros	
☐ 9. De 20 000 FF à moins de 30 000 FF	De 3050 à moins de 4570 euros	
☐ 10. 30 000 FF ou plus	4570 euros ou plus	

D2 Actuellement, pour le ménage diriez-vous que financièrement…

- ☐ 1. Vous êtes à l'aise ➔ ***D4***
- ☐ 2. Ça va ➔ ***D4***
- ☐ 3. C'est juste, il faut faire attention
- ☐ 4. Vous y arrivez difficilement
- ☐ 5. Vous ne pouvez pas y arriver sans faire de dettes

➔ ***Pour l'enquêteur***
- ☐ 98 Ne sait pas
- ☐ 99. Refus de répondre

Si D2 = 1 OU si D2 = 2 ➔ D4

D3 Les moyens financiers de votre foyer vous permettent-ils (ou vous permettraient-ils si vous en éprouviez le besoin) de manger de la viande, poulet, ou du poisson au moins une fois tous les 2 jours ?

-Une seule réponse possible-

➔ ***Consigne à l'enquêteur : Lorsque la personne déclare pouvoir manger de la viande ou du poisson au moins une fois tous les deux jours parce qu'elle lui est fournie gratuitement par un tiers (parents, associations type Restaurants du cœur, etc.), cocher « non »***

- ☐ 1. Oui
- ☐ 2. Non

D3 bis <u>Au cours des 4 dernières semaines,</u> vous est-il arrivé (à vous ou à quelqu'un de votre foyer) de passer une journée complète sans prendre au moins un repas complet, par manque d'argent ?

-Une seule réponse possible-

➔ ***Consigne à l'enquêteur : Lorsque la personne n'a mangé qu'un sandwich (ou que des pâtes…) dans la journée par manque d'argent, cocher « oui ». Mais lorsque c'est par manque de temps, ou par choix, cocher « non »***

- ☐ 1. Oui
- ☐ 2. Non

➔ A TOUS

MAINTENANT, NOUS ALLONS PLUS PARTICULIEREMENT PARLER DE VOS RESSOURCES PERSONNELLES.

**D4 Actuellement… OU Vous avez dit que…**–*Plusieurs réponses possibles-*	**Oui**	**Non**
a. Vous percevez un salaire ou un revenu déclaré de votre activité professionnelle	☐ 1	☐ 2
b. Vous bénéficiez d'une allocation pour adulte handicapé (AAH)	☐ 1	☐ 2
c. Vous bénéficiez d'une pension d'invalidité	☐ 1	☐ 2
d. Vous touchez le RMI	☐ 1	☐ 2
e. Vous touchez une Allocation Parent Isolé (API)	☐ 1	☐ 2
f. Vous touchez des indemnités de chômage (ASSEDIC ou autres)	☐ 1	☐ 2
g. Vous percevez des indemnités journalières	☐ 1	☐ 2
h. Vous percevez une retraite	☐ 1	☐ 2
i. Vous percevez des revenus d'un travail non déclaré	☐ 1	☐ 2
j. Vous n'avez aucune ressource	☐ 1	☐ 2

➔ ***Consigne à l'enquêteur :***
Si D4b = 1 ➔ D5 > D9 bis
Sinon si D4b = 2 ET D4c = 1 ➔ D10 > D12
Sinon si D4b = 2 ET D4g = 1 ➔ D13 > D13 bis
Sinon si D4b = 2 ET (D4d = 1 OU D4e = 1) ➔ D14 > D15 bis
Sinon si D4b = 2 ET D4f = 1 ➔ D16 > D16 bis
Sinon D4b = 2 ET D4c = 2 ET D4d = 2 ET D4e = 2 ET D4f = 2 ET D4g = 2 ➔ ***D17 > D17 bis***

➔ POUR LES PERSONNES QUI BENEFICIENT D'UNE ALLOCATION ADULTE HANDICAPE (AAH) –QU'ELLES TRAVAILLENT OU PAS- *D4B = 1*

D5 A quelle date cette allocation vous a t-elle été attribuée pour la première fois ?

/____/____/____/____/ année /____/____/ mois (***Insister pour avoir la date si l'année est récente***)

D6 Depuis, avez-vous déposé une demande de renouvellement ?
☐ 1. Oui ➔ D7 — ➔ ***Pour l'enquêteur*** ☐ 98. Ne sait pas
☐ 2. Non ➔ ***D8*** — ☐ 99. Refus de répondre

Si D6 =2 ➔ D8

D7 Lors de la dernière décision de renouvellement, votre taux d'incapacité a-t-il été révisé ?
-Une seule réponse possible-
☐ 1. Oui à la hausse
☐ 2. Oui à la baisse
☐ 3. Non

D8 Quel est actuellement votre taux d'incapacité? *-Une seule réponse possible-*
☐ 1. Entre 50 et 79%
☐ 2. 80% ou plus

D9 Quel est son montant mensuel actuel ?
/___/___/___/___/ FF ou euros ➔ ***Pour l'enquêteur*** ☐ 98. Ne sait pas ☐ 99. Refus de répondre ➔ ***D9 bis***

Si D9 = 98, 99 ➔ D9 bis

D9 bis Mais environ, de quel montant est cette allocation ? *-Une seule réponse possible-*
☐ 1. Moins de 2500 francs (moins de 380 euros)
☐ 2. De 2500 FF à moins de 4000 FF (de 380 à moins de 610 euros)
☐ 3. De 4000 FF à moins de 5000 FF (de 610 à moins de 760 euros)

➔ ***Pour l'enquêteur***
☐ 98. Ne sait pas
☐ 99. Refus de répondre

Si D4c = 1 (pension d'invalidité) ➔ D10 > D12
Sinon si D4g = 1 (Indemnités journalières) ➔ D13 > D13 bis
Sinon si D4f = 1 (Indemnités ASSEDIC) ➔ D16 > D16 bis
Sinon D4c = 2 ET D4f = 2 ET D4g = 2= 2 ➔ D17 > D17bis

➔ *POUR LES PERSONNES QUI PERÇOIVENT UNE PENSION D'INVALIDITE D4C= 1*

D10 A quelle date cette pension vous a t-elle été attribuée pour la première fois ?

/____/____/____/____/ année /____/____/ mois (***Insister pour avoir le mois si l'année est récente***)

D11 Quel est son montant mensuel actuel ?

/___/___/___/___/___/ FF ou euros ➔ ***Pour l'enquêteur*** ☐ 98. Ne sait pas ☐ 99. Refus de répondre → ***D11 bis***

Si D11 = 98, 99 ➔ D11 bis

D11 bis Mais environ, de quel montant est cette allocation ? -*Une seule réponse possible*-

- ☐ 1. Moins de 2500 francs (moins de 380 euros)
- ☐ 2. De 2500 FF à moins de 4000 FF (de 380 à moins de 610 euros)
- ☐ 3. De 4000 FF à moins de 5000 FF (de 610 à moins de 760 euros)
- ☐ 4. De 5000 FF à moins de 7000 FF (de 760 à moins de 1070 euros)
- ☐ 5. De 7000 FF à moins de 9000 FF (de 1070 à moins de 1370 euros)
- ☐ 6. 9000 FF ou plus (1370 euros ou plus)

➔ ***Pour l'enquêteur*** ☐ 98. Ne sait pas ☐ 99. Refus de répondre

D12 Dans quelle catégorie vous situez-vous ?

- ☐ 1. Catégorie 1
- ☐ 2. Catégorie 2
- ☐ 3. Catégorie 3

➔ ***Pour l'enquêteur*** ☐ 98. Ne sait pas ☐ 99. Refus de répondre

Si D4f = 1 ➔ D16 ;
Sinon si (D4d OU D4e = 1) ➔ D14 > D15 bis
Sinon D4f = 2 ET (D4d ET D4e = 2) ➔ D17 > D17bis

➔ *POUR LES PERSONNES QUI PERÇOIVENT DES INDEMNITES JOURNALIERES D4G = 1*

D13 Quel est le montant mensuel de vos indemnités journalières ?

/___/___/___/___/___/ FF ou euros ➔ ***Pour l'enquêteur*** ☐ 98. Ne sait pas ☐ 99. Refus de répondre

Si ***C15 bis > 6 mois {arrêt maladie supérieur à 6 mois} ➔ D13 bis ;***
Si C15 bis ≤ 6 mois ET si D4d OU D4e = 1 ➔ D14 ; Si C15 bis ≤ 6 mois ET D4d ET D4e = 2 ➔ D17

D13 bis Quel est le montant mensuel net que votre employeur vous verse en complément ?

/___/___/___/___/___/ FF ou euros ➔ ***Pour l'enquêteur*** ☐ 98. Ne sait pas ☐ 99. Refus de répondre

Si D4d OU D4e = 1 ➔ D14
D4d ET D4e = 2 ➔ D17

➔ *POUR LES PERSONNES QUI TOUCHENT LE RMI OU L'ALLOCATION PARENT ISOLE (API) D4D= 1 OU D4E = 1*

D14 A quelle date cette allocation vous a t-elle été attribuée ?

/____/____/____/____/ année /____/____/ mois (***Insister pour avoir le mois si l'année est récente***)

D15 Quel est son montant mensuel actuel ?

/___/___/___/___/___/ FF ou euros ➔ ***Pour l'enquêteur*** ☐ 98. Ne sait pas ☐ 99. Refus de répondre → ***D15 bis***

Si D15 = 98, 99 ➔ D15 bis

D15 bis Mais environ, de quel montant est cette allocation ? – *Une seule réponse possible-*

- ☐ 1. Moins de 1000 francs (moins de 150 euros)
- ☐ 2. De 1000 FF à moins de 2000 FF (de 150 euros à moins de 300 euros)
- ☐ 3. De 2000 FF à moins de 3000 FF (de 300 à moins de 460 euros)
- ☐ 4. De 3000 FF à moins de 4000 FF (de 460 à moins de 610 euros)
- ☐ 5. 4000 FF ou plus (610 euros ou plus)

➔ ***Pour l'enquêteur***
☐ 98. Ne sait pas
☐ 99. Refus de répondre

Si D4f = 1 ➔ D16
Sinon D4f = 2 ➔ D18

➔ *POUR LES PERSONNES QUI TOUCHENT DES INDEMNITES DE CHOMAGE (ASSEDIC OU AUTRES INDEMNITES) D4F = 1*

D16 Quel est son montant mensuel actuel ?

/___/___/___/___/___/ FF ou euros ➔ ***Pour l'enquêteur*** ☐ 98. Ne sait pas ☐ 99. Refus de répondre → ***D16 bis***

Si D16 = 98, 99 ➔ D16 bis

D16 bis Mais environ, de quel montant sont ces indemnités? – *Une seule réponse possible-*

- ☐ 1. Moins de 2500 francs (moins de 380 euros)
- ☐ 2. De 2500 FF à moins de 4000 FF (de 380 à moins de 610 euros)
- ☐ 3. De 4000 FF à moins de 5000 FF (de 610 à moins de 760 euros)
- ☐ 4. De 5000 FF à moins de 7000 FF (de 760 à moins de 1070 euros)
- ☐ 5. 7000 FF et + (1070 euros ou plus)

➔ ***Pour l'enquêteur***
☐ 98. Ne sait pas
☐ 99. Refus de répondre

➔ ***D17 > D19 ter***

➔ *A TOUS (Y COMRRIS LES PERSONNES QUI TRAVAILLENT OU QUI NE TRAVAILLENT PAS ET QUI N'ONT PAS DE REVENUS SOCIAUX) EXCLURE CEUX QUI ONT EXCLUSIVEMENT UN RMI*

D17 Avez-vous déjà bénéficié du RMI (Revenu Minimum d'Insertion) dans le passé ?

- ☐ 1. Oui → D17 bis
- ☐ 2. Non → ***Si D4b = 1 OU si D4c = 1 ➔ D20 ; si autres cas ➔ D18***

D17 bis Etait-ce ? –*Une seule réponse possible-*

- ☐ 1. Exclusivement avant la connaissance de votre infection à VIH
- ☐ 2. Exclusivement après la connaissance de votre infection à VIH
- ☐ 3. Avant ET après la connaissance de votre infection à VIH

→ ***Si D4b = 1 OU si D4c = 1 ➔ D20***
Si autres cas ➔ D18

➔ *Pour l'enquêteur* ☐ 98 Ne sait pas ☐ 99. Refus de répondre

➔ ***A CEUX QUI TOUCHENT UN RMI, DES INDEMNITES DE CHOMAGE, DES INDEMNITES JOURNALIERES OU QUI NE PERCOIVENT PAS DE REVENUS SOCIAUX***
EXCLURE LES PERSONNES PERCEVANT UNE AAH OU UNE PENSION D'INVALIDITE

D18 Avez-vous déjà bénéficié d'une Allocation Adulte Handicapé (AAH) dans le passé ?
-Une seule réponse possible-

- ☐ 1. Oui
- ☐ 2. Non ➔ ***D20***

D19 Pour quelle raison n'en bénéficiez-vous plus? *–Une seule réponse possible-*

- ☐ 1. Vous n'avez pas renouvelé la demande
- ☐ 2. Le taux d'incapacité reconnu a baissé
- ☐ 3. En raison d'un cumul avec d'autres revenus personnels ou du/de la conjoint(e)
- ☐ 4. Autre. Précisez : ______________________________

→ ***D20***

➔ *A TOUS*

D20 Actuellement, par rapport à votre habitation, vous êtes (vous et/ou votre conjoint(e)/ami(e))…
-Une seule réponse possible-

- ☐ 1. Locataire
- ☐ 2 . Sous-locataire
- ☐ 3. Propriétaire de votre logement (ou accédant à la propriété)
- ☐ 4. Vous vivez chez un (ou des) ami(s)
- ☐ 5. Vous vivez chez vos parents ou chez d'autres membres de votre famille
- ☐ 6. Vous êtes sans domicile fixe (hôtel, etc.)
- ☐ 7. Vous logez dans un foyer ou dans un centre d'hébergement
- ☐ 8. Autre. **D20 Autre**. Précisez : ____

(4 à 8) → ***Module E***

Si D20 = 1 (locataire) ➔ D21 ;
si D20 = 2 OU D20 = 3 ➔ D22 ;
si D20 ≥ 4 ➔ Module suivant

D21 Vivez-vous dans un appartement de coordination thérapeutique (ACT) ou dans un appartement relais ? *–Une seule réponse possible-*

- ☐ 1. Oui
- ☐ 2. Non

➔ *POUR LES LOCATAIRES (Y COMPRIS D'ACT), SOUS-LOCATAIRES OU PROPRIETAIRES*
(D20 = 1 OU 2 OU 3 ➔ D22)

D22 Combien payez-vous pour votre logement (loyer, traites) ?
(➔ *Pour l'enquêteur : avant déduction des aides au logement CAF)*

/___/___/___/___/___/ FF ou euros ➔ ***Pour l'enquêteur*** ☐ 98. Ne sait pas

☐ 00. Non concerné
(propriétaire ayant achevé de payer ses traites)

Si D22 = 00 ➔ D24

D23 Percevez-vous de la Caisse d'Allocation Familiale (CAF) une aide au logement ?
–Une seule réponse possible-

- ☐ 1. Oui
- ☐ 2. Non → ***D24***

➔ ***Pour l'enquêteur***
☐ 98. Ne sait pas

Si D23 = 2 ➔ D24

D23 bis Quel en est le montant mensuel ?
/___/___/___/___/___/ FF ou euros ➔ ***Pour l'enquêteur*** ☐ 98. Ne le connaît pas

D24 Estimez-vous que vos conditions actuelles de logement sont…
–Une seule réponse possible-

- ☐ 1. Très satisfaisantes
- ☐ 2. Satisfaisantes
- ☐ 3. Acceptables
- ☐ 4. Insuffisantes
- ☐ 5. Très insuffisantes

D25 Les critiques suivantes s'appliquent-elles à votre logement ? *–Plusieurs réponses possibles-*

	**Oui**	**Non**
a. Il est trop petit ou n'a pas assez de pièces	☐ 1	☐ 2
b. Il y a trop d'escaliers à monter et à descendre	☐ 1	☐ 2
c. Il est difficile ou trop coûteux à bien chauffer	☐ 1	☐ 2
d. Il est trop humide	☐ 1	☐ 2
e. Il est bruyant (bruits extérieurs et intérieurs)	☐ 1	☐ 2

**D26 Vous le trouvez…***–Plusieurs réponses possibles-*	**Oui**	**Non**
a. Loin du centre	☐ 1	☐ 2
b. Mal desservi par les transports en commun	☐ 1	☐ 2
c. Loin des lieux où vous vous faites soigner	☐ 1	☐ 2
d. Loin de vos proches	☐ 1	☐ 2
e. Loin des services publics (mairie, crèche, école, etc.)	☐ 1	☐ 2
f. Loin de votre travail (ou de celui de votre conjoint) ou des possibilités de travail	☐ 1	☐ 2
g. Situé dans un quartier difficile	☐ 1	☐ 2

Si (A3 O = 1 OU A3 O = 2) ET (A3A > 1996) ➔ D27
SI (A3 O ≠ 1 OU ≠ 2) OU (SI A3 A ≤ 1996) ➔ MODULE E

➔ *POUR CEUX QUI ONT UN EMPLOI OU QUI SONT AU CHOMAGE ET QUI ONT AU MOINS UN ENFANT DE MOINS DE 6 ANS*

D27 Par qui le plus jeune enfant est-il principalement gardé pendant la semaine ?
–Plusieurs réponses possibles-

	**Oui**	**Non**
a. Ecole maternelle	☐ 1	☐ 2
b. Assistante maternelle agréée (nourrice)	☐ 1	☐ 2
c. Assistante maternelle non agréée (nourrice)	☐ 1	☐ 2
d. Crèche collective	☐ 1	☐ 2
e. Crèche parentale	☐ 1	☐ 2
f. Crèche familiale	☐ 1	☐ 2
g. Une personne rémunérée à votre domicile	☐ 1	☐ 2
h. Halte garderie	☐ 1	☐ 2
i. Un membre de votre famille	☐ 1	☐ 2
j. Vous-même	☐ 1	☐ 2
k. Votre conjoint(e)	☐ 1	☐ 2
l. Autre	☐ 1	☐ 2

Module E « Secret, entourage et famille »

NOUS ALLONS PARLER MAINTENANT DE VOTRE FAMILLE ET DE VOS AMIS.

E1 Depuis que vous êtes infecté(e) par le VIH, en avez-vous parlé à…

	Oui				Non			
	**Oui, quand vous l'avez appris vous-même****	**Oui, à l'occasion d'une hospitalisation**	**Oui, au début du traitement**	**Oui, à un autre moment**	**Non, et il / elle ne le sait pas**	**Non, mais il / elle l'a appris par quelqu'un d'autre**	**Vous ne savez pas si il / elle le sait**	**Non Concerné *****
a. Votre père ?	☐ 1	☐ 2	☐ 3	☐ 4	☐ 5	☐ 6	☐ 7	☐ 8
b. Votre mère ?	☐ 1	☐ 2	☐ 3	☐ 4	☐ 5	☐ 6	☐ 7	☐ 8
c. Un frère, une sœur* ?	☐ 1	☐ 2	☐ 3	☐ 4	☐ 5	☐ 6	☐ 7	☐ 8
d. Votre/vos enfant(s) ?	☐ 1	☐ 2	☐ 3	☐ 4	☐ 5	☐ 6	☐ 7	☐ 8
e. D'autres membres de votre famille ?	☐ 1	☐ 2	☐ 3	☐ 4	☐ 5	☐ 6	☐ 7	☐ 8
f. Un ou plusieurs amis proches ?	☐ 1	☐ 2	☐ 3	☐ 4	☐ 5	☐ 6	☐ 7	☐ 8

*Dans le cas où il y a plusieurs frères et sœurs, *si au moins un est au courant*, cocher « Oui »
**Consigne à l'enquêteur : à quelques semaines près
***Non Concerné = membre décédé ou inexistant

E2 Vous est-il déjà arrivé d'être l'objet d'attitudes de rejet ou de discrimination liées directement à votre infection à VIH ?

	**Oui**	**Non**	**NC**	***Si oui*** ➔ **E3 Etait-ce dans les 12 derniers mois ?**	
a. Dans votre famille	☐ 1	☐ 2	☐ 3.	Oui ☐ 1	Non ☐ 2
b. De la part d'amis proches	☐ 1	☐ 2	☐ 3.	Oui ☐ 1	Non ☐ 2
c. Au travail	☐ 1	☐ 2	☐ 3.	Oui ☐ 1	Non ☐ 2
d. De la part de partenaires sexuels / elles	☐ 1	☐ 2	☐ 3.	Oui ☐ 1	Non ☐ 2
e. D'autres personnes	☐ 1	☐ 2	☐ 3.	Oui ☐ 1	Non ☐ 2

E3 Avez-vous eu un contact (même par téléphone, lettre ou e-mail) avec les membres de votre famille ou des amis (ne résidant pas avec vous) ?

**Au cours…**	**a. Membres de la famille**	**b. Amis**
1. De la semaine dernière	☐ 1	☐ 1
2. Du mois dernier	☐ 2	☐ 2
3. Des 3 derniers mois	☐ 3	☐ 3
4. Des 12 derniers mois	☐ 4	☐ 4
5. Jamais ou pas depuis longtemps	☐ 5	☐ 5
6. Sans objet	☐ 6	☐ 6

E4 Au cours des 12 derniers mois, avez-vous été…
Consigne à l'enquêteur ➔ penser à lire la liste verticalement d'abord la colonne 'a' en entier, puis la colonne 'b'

	**a. Soutenue(e) psychologiquement par**	**b. Aidé(e) matériellement* par**
1. Un membre de votre famille	Oui ☐ 1 Non ☐ 2 NC ☐ 3	Oui ☐ 1 Non ☐ 2
2. Votre conjoint/e	Oui ☐ 1 Non ☐ 2 NC ☐ 3	Oui ☐ 1 Non ☐ 2 NC ☐ 3
3. Vos amis	Oui ☐ 1 Non ☐ 2	Oui ☐ 1 Non ☐ 2
4. Une association de lutte contre le sida	Oui ☐ 1 Non ☐ 2	Oui ☐ 1 Non ☐ 2
5. D'autres personnes	Oui ☐ 1 Non ☐ 2	Oui ☐ 1 Non ☐ 2

** Aide matérielle : vêtement, argent*

E4 Autres personnes. Précisez : ____

E5 Combien de personnes vivant avec le VIH connaissez-vous actuellement ?
-*Une seule réponse possible*-
☐ 1. Aucune
☐ 2. Une personne
☐ 3. Deux à quatre
☐ 4. Cinq à neuf
☐ 5. Plus de 10 personnes

E6 Combien de personnes décédées du VIH/sida avez-vous connu personnellement ?
-*Une seule réponse possible*-
☐ 1. Aucune
☐ 2. Une personne
☐ 3. Deux à quatre
☐ 4. Cinq à neuf
☐ 5. Plus de 10 personnes

E7 Avez-vous perdu un(e) conjoint(e) / compagnon(compagne) –*Plusieurs réponses possibles*-
ou des partenaires sexuel(le)s du sida ?

	**Oui**	**Non**
a. Oui, un(e) conjoint(e) / compagnon(compagne)	☐ 1	☐ 2
b. Oui, des partenaires sexuel(le)s	☐ 1	☐ 2

E8 Avez-vous déjà été en prison ? -*Une seule réponse possible*-
☐ 1. Oui, dans le passé
☐ 2. Oui, dans les 12 derniers mois
☐ 3. Non, jamais

➔ ***Pour l'enquêteur***
☐ 99. Refus de répondre

Module F « Vie sexuelle, affective et conjugale »

JE VAIS MAINTENANT VOUS POSER QUELQUES QUESTIONS PLUS PERSONNELLES. JE VOUS RAPPELLE QUE CE QUESTIONNAIRE DEMEURE STRICTEMENT ANONYME. IL SE PEUT QUE NOUS SOYONS AMENES A VOUS POSER CERTAINES QUESTIONS QUI NE VOUS CONCERNENT PAS FORCEMENT.

➔ ***A TOUS***

F1 Au cours de votre vie, avec combien de partenaires différents avez-vous eu des relations sexuelles ***(y compris votre conjoint/e actuel/le)*** **?**

	0	1	2-5	6-10	11-20	21-50	Plus de 50
a. Avec des hommes	☐ 0	☐ 1	☐ 2	☐ 3	☐ 4	☐ 5	☐ 6
b. Avec des femmes	☐ 0	☐ 1	☐ 2	☐ 3	☐ 4	☐ 5	☐ 6

Si F1a = 0 ET si F1b = 0 ET A3S 1 = 2 {✿} ➔ Module « Parentalité » en G6

Si F1a = 0 ET si F1b = 0 ET A3S 1 = 1 {❝} ➔ Module « Parentalité » en G21

F2 Vous vous définissez comme : -*Une seule réponse possible*-

- ☐ 1. Hétérosexuel(le)
- ☐ 2. Bisexuel(le)
- ☐ 3. Homosexuel(le)
- ☐ 4. Transsexuel
- ☐ 5. Vous refusez de vous définir

➔ ***Pour l'enquêteur***
☐ 99. Refus de répondre

F3 Au cours de votre vie, combien avez-vous eu de relations de couple (= relations suivies) ayant duré plus de six mois, que vous habitiez ensemble ou non ***(y compris la relation actuelle)*** **?**

a. Avec des hommes ___________

b. Avec des femmes ___________

F4 Au cours de votre vie, vous est–il arrivé d'obtenir de la drogue ou de l'argent en échange de rapports sexuels ? ***-Une seule réponse possible-***

- ☐ 1. Oui
- ☐ 2. Non

➔ ***Pour l'enquêteur***
☐ 99. Refus de répondre

F5 Au cours de votre vie, avez-vous eu des relations sexuelles avec une personne qui est ou a été toxicomane par voie intra-veineuse : ***-Une seule réponse possible-***

- ☐ 1. Oui
- ☐ 2. Non

➔ ***Pour l'enquêteur*** ☐ 99. Refus de répondre
☐ 98. Ne sait pas

Si F5 = 2 ➔ F7

**F6 C'était…** -*Plusieurs réponses possibles*-	**Oui**	**Non**	**Non concerné**
a. Un(e) ex-conjoint(e)	☐ 1	☐ 2	
b. Votre conjoint(e) actuel(le)	☐ 1	☐ 2	☐ 3
c. Un(e) partenaire de rencontre	☐ 1	☐ 2	

NOUS ALLONS PARLER MAINTENANT DE VOTRE VIE AFFECTIVE ET SEXUELLE ACTUELLE.

F7 Actuellement, avez-vous une relation de couple <u>que vous viviez ensemble ou non</u> ?
-*Une seule réponse possible*-

- ☐ 1. Oui, avec votre mari
- ☐ 2. Oui, avec votre femme
- ☐ 3. Oui, avec un ami / un compagnon
- ☐ 4. Oui, avec une amie / une compagne

(1 à 4) ➔ ***F8***

- ☐ 5. Non, mais vous avez déjà été en couple ➔***F7 bis ET F7 ter***
- ☐ 6. Non et vous n'avez jamais été en couple ➔***F8***

Si F7 = 1 OU 2 OU 3 OU 4 OU 6 ➔ F8 ; Si F7 = 5 ➔ F7 bis et F7 ter

F7 bis Depuis combien de temps, la dernière relation a-t-elle cessé ?
/___/___/___/___/ année /___/___/ mois (si rupture récente)

F7 ter Si vous aviez à donner une raison à cette séparation, vous diriez que…
-Une seule réponse possible-
☐ 1. Vous l'avez quitté(e) à cause du VIH
☐ 2. Il/elle vous a quitté(e) à cause du VIH
☐ 3. Vous vous êtes séparés pour d'autres raisons
☐ 4. Votre conjoint(e) est décédé(e)

F8 <u>Au cours des 12 derniers mois</u>, avez-vous eu des rapports sexuels *(y compris votre conjoint(e) ou ami(e)* ?
☐ 1. Oui → F9 ➔ ***Pour l'enquêteur*** ☐ 99. Refus de répondre
☐ 2. Non → ***<u>Si en couple</u> : F7 = 1, 2, 3 OU 4 OU (F7 = 5 ET F7bis < 12 mois ET F7ter ≠ 4)***
➔ ***de F11 > F16***
↘ ***<u>Si seul</u>*** : [***F7 = 5 ET (F7bis ≥ 12 mois OU F7ter = 4)] OU F7 = 6 ➔ F23***

F9 Combien de partenaires sexuels avez-vous eu ? -y compris votre conjoint(e) ou ami(e)-

	0	1	2	3-4	5-10	11-20	Plus de 20
a. des hommes	☐ 0	☐ 1	☐ 2	☐ 3	☐ 4	☐ 5	☐ 6
b. des femmes	☐ 0	☐ 1	☐ 2	☐ 3	☐ 4	☐ 5	☐ 6

F10 Au cours des <u>4 dernières semaines</u>, avez-vous eu des rapports sexuels ?
☐ 1. Oui → F10 bis
☐ 2. Non ➔ ***<u>si en couple</u> : F7 = 1, 2, 3, OU 4 OU (F7 = 5 ET F7bis < 12 mois ET F7ter ≠ 4) ➔ F11***
↘ ***<u>si seul</u> : F7 = 6 OU [F7 = 5 ET (F7bis ≥ 12 mois OU F7ter = 4)] ➔ F18***

F10 bis Combien environ ?
/__/__/ ➔ ***<u>si en couple</u> : F7 = 1, 2, 3, OU 4 OU (F7 = 5 ET F7bis < 12 mois ET F7ter ≠ 4) ➔ F11***
↘ ***<u>si seul</u> : F7 = 6 OU [F7 = 5 ET (F7bis ≥ 12 mois OU F7ter = 4)] ➔ F18***

➔ ***<u>A TOUS CEUX QUI SONT EN COUPLE ACTUELLEMENT QU'ILS VIVENT OU NON ENSEMBLE (OU QUI ONT ETE EN COUPLE DANS L'ANNEE)</u>***

F11 Depuis quand êtes-vous ensemble ?
/___/___/___/___/ Année /___/___/ mois (insister pour avoir le mois si mise en couple récente)

F12 Votre conjoint(e)/ami(e) connaît-il/elle actuellement votre statut sérologique ?
-Une seule réponse possible-
☐ 1. Oui
☐ 2. Non

➔ *Pour l'enquêteur*
☐ 99. Refus de répondre

F12 bis Connaissez-vous actuellement le statut sérologique de votre conjoint(e)?
-Une seule réponse possible-
☐ 1. Oui, il/elle est séronégatif/ve → ***F14***
☐ 2. Non, il/elle n'a pas fait le test → ***F14***
☐ 3. Non, vous n'en avez pas parlé → ***F14***
☐ 4. Oui, il/elle est séropositif/ve → F13

F13 Depuis que vous êtes ensemble, le statut sérologique de votre conjoint(e) a-t-il changé ?
-Une seule réponse possible-
☐ 1. Oui
☐ 2. Non
☐ 3. Vous ne savez pas
☐ 4. Infection à VIH découverte ensemble

→ ***F14***

F14 Au cours des 12 derniers mois, l'un de vous a-t-il refusé d'avoir des rapports sexuels alors que l'autre le désirait ?

	a. Vous-même	b. Votre conjoint
1. Jamais	☐ 1	☐ 1
2. Rarement	☐ 2	☐ 2
3. Quelquefois	☐ 3	☐ 3
4. Souvent	☐ 4	☐ 4
5. Systématiquement	☐ 5	☐ 5

➔ ***Pour l'enquêteur***
☐ 99. Refus de répondre

F15 Au cours des 12 derniers mois, avec votre conjoint(e), vous …

	a. Pénétration	b. Fellation*
1. N'avez jamais utilisé de préservatif	☐ 1	☐ 1
2. N'en avez presque jamais utilisé	☐ 2	☐ 2
3. En avez utilisé parfois	☐ 3	☐ 3
4. En avez presque toujours utilisé	☐ 4	☐ 4
5. En avez toujours utilisé	☐ 5	☐ 5
6. Vous ne pratiquez pas	☐ 6	☐ 6

***** ***Pour l'enquêteur* : Ne pas parler de fellation si le sujet semble difficile à aborder (étrangers, etc.)**

Si F15 < 5 ➔ F15 bis ; si F15 ≥ 5 ➔ F16

F15 bis Quand vous n'utilisez pas de préservatifs, c'est parce que -*Plusieurs réponses possibles***- :**

	Oui	Non
a. Vous oubliez	☐ 1	☐ 2
b. Vous et votre partenaire avez décidé de ne pas vous protéger	☐ 1	☐ 2
c. Vous refusez ou avez des difficultés ou pas envie d'utiliser le préservatif	☐ 1	☐ 2
d. Votre partenaire refuse ou a des difficultés ou n'a pas envie d'utiliser le préservatif	☐ 1	☐ 2
e. Vous ne risquez pas grand chose car vous êtes tous les deux séropositifs	☐ 1	☐ 2
f. Vous ne risquez pas grand chose, votre charge virale est indétectable	☐ 1	☐ 2
g. Vous comptez sur les traitements d'urgence	☐ 1	☐ 2
h. Vous voulez un enfant	☐ 1	☐ 2

F16 Au cours des 12 derniers mois, l'usage du préservatif est-il un sujet de désaccord (ou de tension) avec votre conjoint(e)/ami(e) ? -*Une seule réponse possible***-**

☐ 1. Oui, souvent
☐ 2. Oui, parfois
☐ 3. Oui, rarement
☐ 4. Non, pas du tout

➔ ***Pour l'enquêteur*** ☐ 99. Refus de répondre

Si F7 = 1, 2, 3, OU 4 OU (F7 = 5 ET F7bis < 12 mois ET F7ter ≠ 4) ET (F9a + F9b) ≥ 2 ➔ F18
Sinon si F7 = 6 OU [F7 = 5 ET (F7bis ≥ 12 mois OU F7ter = 4)] ET (F9a + F9b) ≥ 1 ➔ F18
Sinon si F7 = 1, 2, 3, OU 4 OU (F7 = 5 ET F7bis < 12 mois ET F7ter ≠ 4) ET (F9a + F9b) =1 ➔ F24
Sinon (F9a + F9b) = 0 ➔ F23

➔ *A CEUX QUI DECLARENT AVOIR EU AU MOINS 2 PARTENAIRES SEXUELS S'ILS SONT EN COUPLE OU AU MOINS UN S'ILS SONT SEULS AU COURS DES 12 DERNIERS MOIS*

VOUS ETES SEUL(E) OU EN COUPLE ET VOUS AVEZ DECLARE AVOIR EU UN OU PLUSIEURS « PARTENAIRES OCCASIONNELS ». NOUS ALLONS PARLER MAINTENANT DE CES PARTENAIRES OCCASIONNELS.

F18 Au cours des 12 derniers mois, vous avez dit votre infection à VIH à votre partenaire/ vos partenaires occasionnels… -*Une seule réponse possible*-

☐ 1. Systématiquement
☐ 2. Souvent
☐ 3. Parfois
☐ 4. Jamais

F19 Au cours des 12 derniers mois, avec votre partenaire / vos partenaires occasionnels vous …

	a. Pénétration	b. Fellation
1. Avez toujours utilisé un préservatif	☐ 1	☐ 1
2. En avez presque toujours utilisé	☐ 2	☐ 2
3. En avez utilisé parfois	☐ 3	☐ 3
4. N'en avez presque jamais utilisé	☐ 4	☐ 4
5. N'en avez jamais utilisé	☐ 5	☐ 5
6. N'avez pas pratiqué	☐ 6	☐ 6

Si F19a = 2, 3, 4 OU 5 ➔ F20 et F21 ; sinon si F19a = 1 OU F19a = 6 ➔ ***F22***

F20 La/les personnes avec laquelle/lesquelles vous avez eu une pénétration <u>non protégée</u>, était / étaient… *-Plusieurs réponses possibles-*

	Oui	Non
a. Une/des personnes séropositive(s)	☐ 1	☐ 2
b. Une/des personnes séronégative(s)	☐ 1	☐ 2
c. Une/des personnes dont vous ne connaissiez pas le statut sérologique	☐ 1	☐ 2

F21 Quand vous n'utilisez pas de préservatifs c'est parce que… *-Plusieurs réponses possibles-*

	Oui	Non
a. Vous oubliez	☐ 1	☐ 2
b. Vous pensez que c'est aux autres de se protéger	☐ 1	☐ 2
c. Votre / vos partenaire(s) est / sont séropositif(s)	☐ 1	☐ 2
d. Votre charge virale est indétectable	☐ 1	☐ 2
e. Vous ou votre / vos partenaire(s) avez des difficultés ou refusez le préservatif	☐ 1	☐ 2
f. Mettre un préservatif trahit votre séropositivité	☐ 1	☐ 2
g. Vous comptez sur les traitements d'urgence	☐ 1	☐ 2

F22 Au cours des 12 derniers mois, avez-vous utilisé ou fréquenté pour draguer ?

	Régulièrement	Occasionnellement	Rarement	Jamais
a. Petites annonces	☐ 1	☐ 2	☐ 3	☐ 4
b. Dialogues en direct par minitel, réseaux téléphoniques, Internet	☐ 1	☐ 2	☐ 3	☐ 4
c. Saunas, backrooms, sex-club, video club, clubs échangistes	☐ 1	☐ 2	☐ 3	☐ 4
d. Bars ou discothèques	☐ 1	☐ 2	☐ 3	☐ 4
e. Rencontres chez des amis, dans des associations ou au travail	☐ 1	☐ 2	☐ 3	☐ 4
f. Endroits publics ou lieux de drague extérieurs (parcs, parkings, aire d'autoroute, plages, etc.)	☐ 1	☐ 2	☐ 3	☐ 4

F24 ◄

NOUS ALLONS MAINTENANT VOUS POSER QUELQUES QUESTIONS
SUR LA FAÇON DONT VOUS RESSENTEZ ACTUELLEMENT VOTRE VIE SEXUELLE.

➔ ***A CEUX QUI N'ONT PAS D'ACTIVITE SEXUELLE AU COURS DES 12 DERNIERS MOIS (F8 MODALITE 2)***

F23 Vous n'avez pas d'activité sexuelle actuellement parce que…
-Plusieurs réponses possibles-

	Oui	Non
a. Vous n'en avez tout simplement pas envie	☐ 1	☐ 2
b. Vous n'avez pas trouvé quelqu'un qui vous plaise	☐ 1	☐ 2
c. Vous êtes trop fatigué(e), en trop mauvaise santé	☐ 1	☐ 2
d. Vous vous sentez moins séduisant(e)	☐ 1	☐ 2
e. Vous vous sentez isolé(e) et vous ne savez pas comment rencontrer quelqu'un	☐ 1	☐ 2
f. Vous avez peur d'être rejeté(e)	☐ 1	☐ 2
g. Vous avez peur de contaminer votre partenaire	☐ 1	☐ 2
h. Vous préférez ne pas avoir de rapports plutôt que d'avoir à utiliser des préservatifs	☐ 1	☐ 2
i. Vous avez des problèmes sexuels (impuissance, frigidité, troubles de l'érection…)	☐ 1	☐ 2

→ ***F27***

➔ ***A CEUX QUI ONT EU UNE ACTIVITE SEXUELLE AU COURS DES 12 DERNIERS MOIS (F8 MODALITE 1)***

F24 Au cours des 12 derniers mois, vous diriez que votre activité sexuelle a été…
-Une seule réponse possible-

☐ 1. Plus fréquente que l'an dernier
☐ 2. N'a pas changé
☐ 3. Moins fréquente que l'an dernier

F25 Comment qualifieriez-vous votre vie sexuelle actuelle ? *-Une seule réponse possible-*

☐ 1. Satisfaisante
☐ 2. Plutôt satisfaisante
☐ 3. Plutôt pas satisfaisante
☐ 4. Pas satisfaisante

F26 Actuellement… *-Plusieurs réponses possibles-*

	Oui	Non
a. Du fait de votre infection à VIH, vous limitez le nombre de vos partenaires	☐ 1	☐ 2
b. Du fait de votre infection à VIH, vous ne pratiquez plus la pénétration	☐ 1	☐ 2
c. Du fait de votre infection à VIH, vous recherchez des partenaires comme vous séropositifs	☐ 1	☐ 2
d. Du fait de votre infection à VIH, vous payez pour avoir des rapports sexuels	☐ 1	☐ 2
e. Du fait de votre infection à VIH, vous n'avez que des partenaires occasionnels	☐ 1	☐ 2

➔ ***A TOUS***

F27 Avez-vous entendu parler d'un traitement d'urgence qui, pris juste après un rapport non protégé ou une rupture de préservatif peut empêcher la transmission du VIH à une personne séronégative ?

☐ 1. Oui
☐ 2. Non → ***F28***

F27 bis Est-il arrivé que votre partenaire ou un(e) de vos partenaires utilise ce traitement ?
-Une seule réponse possible-

☐ 1. Oui, une fois
☐ 2. Oui, plusieurs fois
☐ 3. Non, jamais → ***F28***

F27 ter Dans ce cas ou ces cas… *-Plusieurs réponses possibles-*

	Oui	Non
a. Votre partenaire est allé(e) aux urgences d'un hôpital	☐ 1	☐ 2
b. Votre partenaire a consulté le médecin qui vous suit pour le VIH	☐ 1	☐ 2
c. Vous lui avez donné votre traitement	☐ 1	☐ 2
d. Autre	☐ 1	☐ 2

F27 ter Autre. Précisez : ____

F28 Vous-même ou votre partenaire, avez-vous déjà utilisé le préservatif féminin ? *(➔ Consigne à l'enquêteur : à poser aussi aux homosexuels*) -*Une seule réponse possible*

☐ 1. Oui
☐ 2. Non

→**F28 bis C'est…** -*Une seule réponse possible*-

☐ 1. Pour essayer (moins de 3 fois)
☐ 2. De temps à autre
☐ 3. Régulièrement

Si A3S 1 = 1 {❝} ➔ G15
Si A3S 1 = 1 {✿} ➔ G0 a

Module G « parentalité »

➔ *POUR TOUTES LES FEMMES*

NOUS ALLONS PARLER MAINTENANT DES GROSSESSES ET DES ENFANTS.
VOUS M'AVEZ DIT QUE VOUS AVEZ EU __________ ENFANTS / N'AVIEZ PAS D'ENFANT.

G0 a Avant de connaître votre infection à VIH, avez-vous eu des grossesses qui ont été interrompues (IVG exclusivement) ? (*➔ pour l'enquêteur : ne pas prendre en compte l'éventuelle grossesse où la séropositivité au VIH a été découverte*) *-Une seule réponse possible-*

☐ 1. Oui ➔ **G0 b Combien ? _________**
☐ 2. Non

G1 Après le dépistage de votre infection à VIH, combien de fois avez-vous été enceinte -y compris IVG et fausses couches mais en dehors d'une éventuelle grossesse actuelle- ?
➔ Pour l'enquêteur : Ne compter QUE les grossesses survenues APRES la connaissance du diagnostic positif.

/____/
☐ 00. Jamais ➔ ***Passez à G6***

G2 Pour chacune de ces grossesses…

	a.		b.			
**S'agissait-il d'une grossesse…**	**Désirée ?**	**Accidentelle ?**	**Fausse couche ?**	**Interrompue à cause de votre infection à VIH ?***	**Interrompue pour d'autres raisons ?***	**Menée à terme ?**
- 1é grossesse	☐ 1	☐ 2	☐ 0	☐ 1	☐ 2	☐ 3
- 2é grossesse	☐ 1	☐ 2	☐ 0	☐ 1	☐ 2	☐ 3
- 3é grossesse	☐ 1	☐ 2	☐ 0	☐ 1	☐ 2	☐ 3
- 4é grossesse	☐ 1	☐ 2	☐ 0	☐ 1	☐ 2	☐ 3

*** Pour l'enquêteur, ne prendre que la raison principale**

Si G2b ≠ 3 *➔ G6 ; si G2b = 3 ➔ G3*

G3 L'enfant / les enfants nés ont-ils été infectés par le VIH ?

______ ***Si aucun, notez 0 et passez à G6*** ***➔ Pour l'enquêteur*** ☐ 98. Ne sait pas
☐ 99. Refus de répondre

Si G3 = 0 OU 98 OU 99 ➔ G6 ; Si G3 ≥ 1 ➔ G4

➔ ***POUR LES FEMMES QUI ONT DES ENFANTS INFECTES PAR LE VIH G3 > 0***

G4 Avez-vous perdu un ou plusieurs enfants ? *-Une seule réponse possible-*
☐ 1. Oui
☐ 2. Non

G5 Les enfants sont-ils…

	**a. Traités pour le VIH ?**		**b. En bonne santé ?**	
- 1é enfant	☐ 1 Oui	☐ 2 Non	☐ 1 Oui	☐ 2 Non
- 2é enfant	☐ 1 Oui	☐ 2 Non	☐ 1 Oui	☐ 2 Non
- 3e enfant	☐ 1 Oui	☐ 2 Non	☐ 1 Oui	☐ 2 Non
- 4é enfant	☐ 1 Oui	☐ 2 Non	☐ 1 Oui	☐ 2 Non

➔ ***POUR TOUTES LES FEMMES***

G6 Selon vous, le risque que l'enfant d'une femme enceinte séropositive soit contaminé à l'heure actuelle en France est…-*Une seule réponse possible***-**

- ☐ 1. Très important
- ☐ 2. Assez important
- ☐ 3. Peu important
- ☐ 4.Très faible

G7 Avez-vous le projet d'avoir un enfant ? -*Une seule réponse possible*-

- ☐ 1. Oui, vous êtes enceinte → ***G10***
- ☐ 2. Oui, vous cherchez actuellement à avoir un enfant → ***G10***
- ☐ 3. Oui, vous avez le désir d'avoir un enfant
- ☐ 3bis. Peut-être, plus tard
- ☐ 4. Non ➔ ***G14***
- ☐ 5. Vous ne pouvez pas / plus avoir d'enfant (ménopause, ligature de trompes, stérilité) ➔ ***G27***

Si G7 = 3 OU 3bis ➔ G8
Si G7 = 1 OU 2 ➔ G10
Si G7 = 4 ➔ G14
Si G7 = 5 ➔ G27

G8 Attendez-vous une occasion particulière pour avoir un (votre prochain) enfant ?
-*Une seule réponse possible*-

- ☐ 1. Oui
- ☐ 2. Non → ***G10***

Si G8 = 1 ➔ G9, si G8 = 2 ➔ G10

G9 Est-ce…-*Plusieurs réponses possibles***-**

	Oui	Non	***Pour l'enquêteur*** NC
a. Une stabilité dans le couple / d'avoir un conjoint	☐ 1	☐ 2	☐ 3
b. Une meilleure situation économique et professionnelle	☐ 1	☐ 2	☐ 3
c. Que votre maladie (ou celle de votre conjoint) soit stabilisée ou que votre état de santé (ou celui de votre conjoint) soit meilleur	☐ 1	☐ 2	☐ 3
d. Que les traitements soient plus efficaces pour prévenir la transmission mère enfant	☐ 1	☐ 2	☐ 3
e. Que les traitements soient moins nocifs pour le bébé	☐ 1	☐ 2	☐ 3
f. Que d'éventuels problèmes de fertilité soient résolus	☐ 1	☐ 2	☐ 3
g. Que vous ne preniez plus de traitement de substitution	☐ 1	☐ 2	☐ 3
h. Que vous (ou votre partenaire) ne preniez plus de traitement pour l'hépatite C	☐ 1	☐ 2	☐ 3
i. Un risque moindre de contaminer votre conjoint	☐ 1	☐ 2	☐ 3

→ **G10 En avez-vous parlé avec un médecin ?**

	Oui	Non
a. Votre médecin traitant pour le VIH	☐ 1	☐ 2
b. Un gynécologue	☐ 1	☐ 2
c. Un autre médecin	☐ 1	☐ 2
	↓ ***G11***	↘ ***G12***

Si G10 a, G10 b OU G10 c = 1 {oui à au moins une modalité} ➔ G11 ;
Sinon G10a, G10b ET G10c = 2 ➔ G12

G11 Vous avez rencontré chez le/les médecin(s) avec qui vous en avez parlé une attitude…

	**Très favorable**	**Assez favorable**	**Pas très favorable**	**Pas du tout favorable**
a. Médecin traitant pour le VIH	☐ 1	☐ 2	☐ 3	☐ 4
b. Gynécologue	☐ 1	☐ 2	☐ 3	☐ 4
c. Autre médecin	☐ 1	☐ 2	☐ 3	☐ 4

G12 Depuis peu, l'assistance médicale à la procréation par insémination ou fécondation in vitro est possible pour les femmes séropositives. Le savez-vous ? *–Une seule réponse possible-*
☐ 1. Oui
☐ 2. Non ⟶ ***G14***

G13 Souhaiteriez-vous en bénéficier ? *–Une seule réponse possible-*
☐ 1. Oui
☐ 2. Vous ne savez pas
☐ 3. Non
☐ 4. Vous en avez déjà bénéficié

G14 Actuellement, faites-vous quelque chose pour ne pas être enceinte ? *–Une seule réponse possible-*
☐ 1. Oui
☐ 2. Non
☐ 3. Non concernée (vous n'avez pas de relations sexuelles avec des hommes) ⟶ ***G27*** (2, 3)

Si G14 = 2 OU = 3 ➔ G27

**G14 bis Que faites-vous ?** *–Plusieurs réponses possibles-*	**Oui**	**Non**	
a. Vous prenez la pilule	☐ 1	☐ 2	⟶ ***G27*** (a–e)
b. Vous avez un stérilet	☐ 1	☐ 2	
c. Vous utilisez des préservatifs masculins/féminins	☐ 1	☐ 2	
d. Vous utilisez des crèmes /spermicides /ovules	☐ 1	☐ 2	
e. Vous pratiquez avec votre conjoint le retrait / la continence périodique	☐ 1	☐ 2	
f. Vous faites autre chose	☐ 1	☐ 2	

G14 bis Autre. Précisez : ____

➔ ***Pour tous les hommes***

NOUS ALLONS PARLER MAINTENANT DES ENFANTS.
VOUS M'AVEZ DIT QUE VOUS AVEZ EU ___________ ENFANTS / N'AVIEZ PAS D'ENFANT.

G15 Depuis que vous connaissez votre infection à VIH, est-il arrivé qu'une femmes soit enceinte de vous ?
➔ Pour l'enquêteur : Ne compter QUE les grossesses survenues APRES la connaissance du diagnostic positif. Comptabiliser les IVG et fausses couches mais en dehors d'une éventuelle grossesse actuelle.
–Une seule réponse possible-

☐ 1. Oui
☐ 2. Non ⟶ *G21*

G16 Combien de fois ?

____

G17 Pour chacune de ces grossesses…

	a.		b.			
**S'agissait-il d'une grossesse…**	**Désirée ?**	**Accidentelle ?**	**Fausse couche ?**	**Interrompue à cause de votre infection à VIH ?***	**Interrompue pour d'autres raisons ?***	**Menée à terme ?**
- 1é grossesse	☐ 1	☐ 2	☐ 0	☐ 1	☐ 2	☐ 3
- 2é grossesse	☐ 1	☐ 2	☐ 0	☐ 1	☐ 2	☐ 3
- 3é grossesse	☐ 1	☐ 2	☐ 0	☐ 1	☐ 2	☐ 3
- 4é grossesse	☐ 1	☐ 2	☐ 0	☐ 1	☐ 2	☐ 3

*** Pour l'enquêteur, ne prendre que la raison principale**

Si G17b ≠ 3 ➔ G21 ; Si G17b = 3 ➔ G18

G18 L'enfant / les enfants nés ont-ils été infectés par le VIH ?
______ ***Si aucun, notez 0 et passez à G21*** ***➔ Pour l'enquêteur*** ☐ 98. Ne sait pas
☐ 99. Refus de répondre

Si G18 = 0 OU 98 OU 99➔ G21 ; Si G18 ≥ 1 ➔ G19

➔ ***Pour les hommes qui ont des enfants contamines par le VIH G18 > 0***

G19 Avez-vous perdu un ou plusieurs enfants ? *–Une seule réponse possible-*

☐ 1. Oui
☐ 2. Non

G20 Les enfants vivants sont-ils…

	**a. Traités pour le VIH ?**		**b. En bonne santé ?**	
- 1é enfant	☐ 1 Oui	☐ 2 Non	☐ 1 Oui	☐ 2 Non
- 2é enfant	☐ 1 Oui	☐ 2 Non	☐ 1 Oui	☐ 2 Non
- 3é enfant	☐ 1 Oui	☐ 2 Non	☐ 1 Oui	☐ 2 Non
- 4é enfant	☐ 1 Oui	☐ 2 Non	☐ 1 Oui	☐ 2 Non

➔ ***A TOUS LES HOMMES***

G21 Actuellement, avez-vous le projet d'avoir un enfant ? -*Une seule réponse possible***-**

- ☐ 1. Oui, votre conjointe est enceinte → ***G23***
- ☐ 2. Oui, vous cherchez actuellement à avoir un enfant → ***G23***
- ☐ 3. Oui, vous (et/ou votre conjointe) avez le désir d'avoir un enfant
- ☐ 3bis. Peut-être plus tard
- ☐ 4. Non ➔ ***G26***
- ☐ 5. Vous ne pouvez pas / plus avoir d'enfant (stérilité, ligature des canaux spermatiques, etc.) ➔ ***G27***

Si G21 = 3 OU 3bis ➔ G22 et G22 bis
Si G21 = 1 OU 2 ➔ G23
Si G21 = 4 ➔ G26
Si G21 = 5 ➔ G27

G22 Attendez-vous une occasion particulière pour avoir un (autre) enfant ? –*Une seule réponse possible*-

- ☐ 1. Oui ou peut-être
- ☐ 2. Non → ***G23***

Si G22 = 1 ➔ G22 bis ; sinon G22 = 2 ➔ G23

G22 bis Est-ce… -*Plusieurs réponses possibles*-

	**Oui**	**Non**	***Pour l'enquêteur*** **NC**
a. Une stabilité dans le couple, d'être en couple	☐ 1	☐ 2	☐ 3
b. Une meilleure situation économique et professionnelle	☐ 1	☐ 2	☐ 3
c. Que votre maladie (ou celle de votre conjointe) soit stabilisée ou que votre état de santé (ou celle de votre conjointe) soit meilleur	☐ 1	☐ 2	☐ 3
d. Que les traitements soient plus efficaces pour prévenir la transmission mère enfant	☐ 1	☐ 2	☐ 3
e. Que les traitements soient moins nocifs pour le bébé	☐ 1	☐ 2	☐ 3
f. Que d'éventuels problèmes de fertilité soient résolus	☐ 1	☐ 2	☐ 3
g. Que vous ne preniez plus de traitement de substitution	☐ 1	☐ 2	☐ 3
h. Que vous (ou votre conjointe) ne preniez plus de traitement pour l'hépatite C	☐ 1	☐ 2	☐ 3
i. Un risque moindre de contaminer votre conjointe	☐ 1	☐ 2	☐ 3

G23 En avez-vous parlé (vous ou votre conjointe) avec un médecin ?

	**Oui**	**Non**
a. Votre médecin traitant pour le VIH	☐ 1	☐ 2
b. Gynécologue de votre conjointe / amie	☐ 1	☐ 2
c. Un autre médecin	☐ 1	☐ 2

Si G23a, G23b OU G23c = 1 { oui à au moins 1 modalité} ➔ G24 ; si G23a, G23b ET G23c = 2 ➔ G25

G24 Avez-vous rencontré chez le/les médecin(s) avec qui vous (ou votre conjointe) en avez parlé une attitude…

	**Très favorable**	**Assez favorable**	**Pas très favorable**	**Pas du tout favorable**
a. Médecin traitant pour le VIH	☐ 1	☐ 2	☐ 3	☐ 4
b. Gynécologue	☐ 1	☐ 2	☐ 3	☐ 4
c. Autre médecin	☐ 1	☐ 2	☐ 3	☐ 4

G25 Depuis quelques années, l'assistance médicale à la procréation par inséminations ou fécondation in vitro est possible pour aider les couples dont l'homme est infecté par le VIH à avoir des enfants.
Le savez-vous ? *–Une seule réponse possible-*

- ☐ 1. Oui
- ☐ 2. Non → ***G26***

G25 bis Souhaiteriez-vous en bénéficier ? *–Une seule réponse possible-*

- ☐ 1. Oui
- ☐ 2. Ne sait pas
- ☐ 3. Non
- ☐ 4. Vous en avez déjà bénéficié

G26 Actuellement, (dans votre couple) faites-vous quelque chose pour ne pas avoir d'enfant ?
–Une seule réponse possible-

- ☐ 1. Oui
- ☐ 2. Non
- ☐ 3. Non concerné (vous n'avez pas de relations sexuelles avec des femmes)

Si G26 = 2 OU = 3 ➔ G27

**G26 bis Que faites-vous ?** *–Plusieurs réponses possibles-*	**Oui**	**Non**
a. Vous prenez la pilule	☐ 1	☐ 2
b. Vous avez un stérilet	☐ 1	☐ 2
c. Vous utilisez des préservatifs masculins/féminins	☐ 1	☐ 2
d. Vous utilisez des crèmes /spermicides /ovules	☐ 1	☐ 2
e. Vous pratiquez avec votre conjoint le retrait / la continence périodique	☐ 1	☐ 2
f. Vous faites autre chose	☐ 1	☐ 2

G26 bis Autre. Précisez : ____

➔ ***A TOUS***

G27 Au cours des douze derniers mois, avez-vous…

	**Jamais**	**1 à 2 fois / an**	**Une fois / mois**	**Une fois / semaine**	**Plus souvent**
a. Pratiqué une activité artistique et culturelle (musique, cinéma, peinture, etc.).	☐ 1	☐ 2	☐ 3	☐ 4	☐ 5
b. Pratiqué un sport	☐ 1	☐ 2	☐ 3	☐ 4	☐ 5
c. Participé à titre bénévole aux activités d'une association d'entraide ou caritative	☐ 1	☐ 2	☐ 3	☐ 4	☐ 5
d. Participé à titre bénévole aux activités d'une autre association (politique, culturelle, locataire, syndicale, etc.)	☐ 1	☐ 2	☐ 3	☐ 4	☐ 5
e. Participé à une activité religieuse	☐ 1	☐ 2	☐ 3	☐ 4	☐ 5

**G28 Au cours des 12 derniers mois, avez-vous…**	**Oui**	**Non**
a. donné de l'argent pour la lutte contre le sida ?	☐ 1	☐ 2
b. été volontaire ou militant(e) d'associations de lutte contre le sida ?	☐ 1	☐ 2
c. été un(e) permanent(e) ou un(e) salarié(e) d'associations de lutte contre le sida ?	☐ 1	☐ 2
d. participé à une activité de l'association (groupe de paroles, soirées, ateliers…) ?	☐ 1	☐ 2

JE VOUS REMERCIE VIVEMENT DE VOTRE COOPERATION.
MEME SI CERTAINES QUESTIONS ONT PU VOUS SEMBLER TRES INDISCRETES, SACHEZ QUE VOS REPONSES SONT IMPORTANTES POUR MIEUX ANALYSER LA SITUATION DES PERSONNES ATTEINTES D'INFECTION A VIH EN FRANCE ET DEFINIR DES ACTIONS A L'ECHELON NATIONAL AFIN D'AMELIORER CETTE SITUATION.
BONNE JOURNEE, MERCI ET AU REVOIR.

Observations de l'enquêteur :

(*à remplir par l'enquêteur après l'interview*)

QUESTIONNAIRE COMPLET : TOTAL EN MN |__|__|

1 Accueil de l'enquête

☐ 1. La personne a accepté de répondre : Immédiatement

☐ 2. La personne a accepté de répondre avec réticence

→ **En cas de réticence, principal motif invoqué :**

- ☐ 1. Durée
- ☐ 2. Par principe (ne répond à aucun sondage)
- ☐ 3. Trop sollicité par d'autres enquêtes et essais thérapeutiques sur le VIH
- ☐ 4. Sujet, enfants ou autres accompagnateurs
- ☐ 5. Mauvaise condition physique
- ☐ 6. Autre Précisez : _________________

2 L'entretien s'est déroulé…

- ☐ 1. Sans interruption
- ☐ 2. La personne a signifié à une ou plusieurs reprises son envie d'arrêter
- ☐ 3. A été perturbé par ses enfants, le service ou la personne qui l'accompagnait

3 Attitude de l'enquêté(e). La personne que vous avez interrogé était…

- ☐ 1. Détendue
- ☐ 2. Pressée par le temps
- ☐ 3. Maîtrisait mal la langue française
- ☐ 4. Fatiguée

4 Appréciation des réponses : Les réponses de cette personne ont été…

- ☐ 1. Très sincères
- ☐ 2. Sincères
- ☐ 3. Pas sincères
- ☐ 4. Pas du tout sincères
- ☐ 5. NSP

5 Si abandon en cours de questionnaire, raison de l'abandon :

- ☐ 1. Questionnaire trop long
- ☐ 2. Questions trop intimes
- ☐ 3. Interruption par le service
- ☐ 4. Interruption par l'entourage du répondant
- ☐ 5. Fatigue physique
