# Supplementary material for: Increase in Unemployment over the 2000’s: Comparison between People Living with HIV and the French General Population
Source: PLoS One. 2016 Nov 4;11(11):e0165634. doi: 10.1371/journal.pone.0165634 (PMC5096670; doi:10.1371/journal.pone.0165634)
Supplement: S4 File — (PDF) [file pone.0165634.s004.pdf]

Vespa2

VIH : Enquête Sur les Personnes Atteintes

QUESTIONNAIRE FACE-À-FACE

SOMMAIRE

MODULE A : CARACTERISTIQUES SOCIO-DEMOGRAPHIQUES 4

MODULE B : L'HISTOIRE DE LA MALADIE, L'ACCES AUX SOINS, LA PRISE EN CHARGE MEDICALE, CONNAISSANCE DE LA MALADIE ET DE LA CONSOMMATION DE PRODUITS… . 6

MODULE B1 : DEPISTAGE 6

MODULE B2 : PRISE EN CHARGE MEDICALE... 9

MODULE B3 : CONNAISSANCE SUR LA MALADIE 16

MODULE B4 : CONSOMMATION DE PRODUITS 18

MODULE C : INSERTION PROFESSIONNELLE 23

MODULE D : RESSOURCES ET CONDITIONS DE VIE.. 29

MODULE E : LIENS SOCIAUX, DISCRIMINATION, STIGMATISATION 32

MODULE F : VIE AFFECTIVE, CONJUGALE ET SEXUELLE 38

A - MODULE HSH... 41

B - MODULE FSF 53

C - MODULE HETEROSEXUEL (REPONDANT FEMME OU MALE TO FEMALE [MTOF])........ 54

D - MODULE HETEROSEXUEL (REPONDANT HOMME OU FEMALE TO MALE [FTOM]) 59

E – MODULE COMMUN.. 64

MODULE G : SANTE MENTALE 66

EPISODE DEPRESSIF CARACTERISE 66

ANXIETE GENERALISEE 66

NEVEROSE POST TRAUMATIQUE 67

EPISODE DEPRESSIF CARACTERISE – ANHEDONIE 69

NEVROSE POST TRAUMATIQUE 71

MODULE H : RELATIONS AVEC LES ASSOCIATIONS DE LUTTE CONTRE LE SIDA 77

QUESTIONNAIRE CAPI
VESPA 2

Date !

Numéro Identifiant :

Accord pour l'accès aux données de sécurité sociale

- ☐ 1 oui
- ☐ 2 non

Type de structure

- ☐ 1 CHU
- ☐ 2 CH
- ☐ 3 Privé

Numéro de département de la structure :

QUESTIONNAIRE CAPI
VESPA 2

MODULE A : CARACTERISTIQUES SOCIO-DEMOGRAPHIQUES

A.1 Vous êtes de sexe :
☐ 1 Masculin
☐ 2 Féminin
☐ 3 **vous êtes** transgenre

A.2 Quels sont votre mois et votre année de naissance ?
/__/__/ Mois et /_1_/_9_/__/__/ ans

A.3 Combien d'enfants avez-vous eu ? /____//____/
(*Tous les enfants avec lesquels il y a un lien de filiation : enfants biologiques et adoptés*)

A.4 Combien de personnes vivent dans votre logement, y compris vous-même ?

/____/ **/____/**

Si A4 =1 passer directement à A8 / Sinon continuer en A5

A.5 Actuellement, dans ce logement, vivez-vous avec votre conjoint, marié ou pas ?
☐ 1 Oui
☐ 2 Non
☐ 97. Non concerné (ne pas suggérer)
☐ 98. Ne sait pas (ne pas suggérer)
☐ 99. Refus de réponse (ne pas suggérer)

A.6 Actuellement, dans ce logement, vivez-vous avec d'autres personnes de plus de 18 ans ?
☐ 1 Oui
☐ 2 Non
☐ 97. Non concerné
☐ 98. Ne sait pas
☐ 99. Refus de réponse

A.7 Actuellement, dans ce logement, vivez-vous avec des enfants de moins de 18 ans ?
☐ 1 Oui➔ **A7.1**
☐ 2 Non
☐ 97. Non concerné
☐ 98. Ne sait pas
☐ 99. Refus de réponse

A7.1 Parmi eux, combien ont moins de 14 ans ?

/____//____/

A.8 Dans quel pays êtes-vous né(e) ?

A.9 Dans quel pays est né votre père ?

A.10 Dans quel pays est née votre mère ?

A.11 Quelle est votre nationalité ?
☐ 1 Français de naissance
☐ 2 Français par acquisition
☐ 3 Etranger, quelle est votre nationalité ? Précisez

A.11 bis. Avez-vous travaillé au cours du dernier mois (au moins un jour) ?
☐ 1 Oui
☐ 2 Non
☐ 97. Non concerné
☐ 98. Ne sait pas
☐ 99. Refus de réponse

A.12 Quel est votre diplôme le plus élevé ?
☐ 1 Aucun diplôme
☐ 2 Un diplôme de fin d'études primaires ou diplôme étranger de même niveau
☐ 3 Brevet des collèges, BEPC, brevet élémentaire ou diplôme étranger de même niveau
☐ 4 CAP, BEP ou diplôme de même niveau
☐ 5 Baccalauréat technologique ou professionnel ou diplôme de même niveau
☐ 6 Baccalauréat général, brevet supérieur, capacité en droit ou diplôme étranger de même niveau
☐ 7 Diplôme de niveau Bac + 2
☐ 8 Diplôme de niveau Bac + 3 (licence, diplôme professionnel etc.)
☐ 9 Diplôme de niveau Bac + 4 (master, maitrise)
☐ 10 Diplôme de niveau Bac + 5 (DESS, DEA, diplôme d'ingénieur, doctorat)
☐ 97. Non concerné (ne pas suggérer)
☐ 98. Ne sait pas (ne pas suggérer)
☐ 99. Refus de réponse (ne pas suggérer)

A.13 Est-ce que la religion est dans votre vie…
☐ 1 Très importante
☐ 2 Importante
☐ 3 Pas très importante
☐ 4 Pas importante du tout
☐ 97. Non concerné (ne pas suggérer)
☐ 98. Ne sait pas (ne pas suggérer)
☐ 99. Refus de réponse (ne pas suggérer)

Si A11=1 ➔ Fin du module A

Si A11 = 2 ou 3 (*personnes étrangères ou françaises par acquisition)* ➔A14

A.14 En dehors de la France et de votre pays d'origine, dans quels pays, avez-vous vécu au moins un an depuis vos 16 ans ?
PLUSIEURS REPONSES POSSIBLES

A.15 Quand êtes-vous arrivé(e) en France ? *(Insister pour avoir le mois si l'arrivée est récente*
Faire préciser le mois pour une arrivée depuis 2005 inclus

/__/__/__/__/ année /__/__/ mois 98 = NSP 99 = refus

Si A11 = 2 (Français par acquisition) ➔ ne pas poser A16 et passer directement à A 17
Si A11 = 3 (Etrangers) ➔continuer en A16

A.16 Actuellement, quelle est votre situation administrative ?
- ☐ 1 Vous avez une carte de résident
- ☐ 2 Vous avez une carte de séjour temporaire (CST)
- ☐ 3 Vous avez un visa touristique
- ☐ 4 Vous avez une autorisation provisoire de séjour (APS)
- ☐ 5 Vous avez un récépissé de demande de titre de séjour
- ☐ 6 Vous êtes en attente de régularisation
- ☐ 7 Vous êtes Citoyen de l'Union Européenne
- ☐ 97. Non concerné (ne pas suggérer)
- ☐ 98. Ne sait pas (ne pas suggérer)
- ☐ 99. Refus de réponse (ne pas suggérer)

A.17 Quand vous êtes arrivé en France, c'était
PLUSIEURS REPONSES POSSIBLES
- ☐ 1 pour travailler
- ☐ 2 pour faire des études
- ☐ 3 pour fuir des conflits ou des violences politiques
- ☐ 4 pour rejoindre votre famille
- ☐ 5 pour des vacances
- ☐ 6 pour affaires
- ☐ 7 pour changer de vie
- ☐ 8 pour vous soigner
- ☐ 9 pour une autre raison : préciser
- ☐ 97. Non concerné (ne pas suggérer)
- ☐ 98. Ne sait pas (ne pas suggérer)
- ☐ 99. Refus de réponse (ne pas suggérer)

A.18 Concernant vos proches, êtes vous arrivé(e) en France … ?
UNE SEULE REPONSE POSSIBLE

A18.1 En même temps que votre conjoint, marié ou pas, ou pour le/la rejoindre
A18.2 En même temps que votre/ vos enfants ou pour le/les rejoindre ➔ ne pas poser si A3=0
A18.3 En même temps que votre père et/ou votre mère ou pour les rejoindre
A18.4 En même temps que d'autres parents proches ou pour les rejoindre
- ☐ 1 Oui
- ☐ 2 Non
- ☐ 97. Non concerné (ne pas suggérer)
- ☐ 98. Ne sait pas (ne pas suggérer)
- ☐ 99. Refus de réponse (ne pas suggérer)

A.19 Depuis que vous êtes arrivé(e) en France, avez-vous fait des voyages ou des séjours dans votre pays d'origine ?
- ☐ 1 non, pas encore ➔ **Fin du module A, aller directement au module B**
- ☐ 2 oui, une fois
- ☐ 3 oui, plusieurs fois
- ☐ 97. Non concerné (ne pas suggérer)
- ☐ 98. Ne sait pas (ne pas suggérer)
- ☐ 99. Refus de réponse (ne pas suggérer)

Si A 19= 2 ou 3 ➔poser les questions A 19.1 et A 19.2

A19.1 En quelle année avez-vous fait votre dernier séjour ? /__/__/__/__/

A19.2 Combien de temps a duré ce séjour ?
- ☐ 1 moins d'1 mois
- ☐ 2 Entre 1 et 2 mois
- ☐ 3 entre 2 mois et 5 mois
- ☐ 4 entre 5 mois et 12 mois
- ☐ 5 plus de 12 mois
- ☐ 97. Non concerné (ne pas suggérer)
- ☐ 98. Ne sait pas (ne pas suggérer)
- ☐ 99. Refus de réponse (ne pas suggérer)

MODULE B : L'HISTOIRE DE LA MALADIE, L'ACCES AUX SOINS, LA PRISE EN CHARGE MEDICALE, CONNAISSANCE DE LA MALADIE ET LA CONSOMMATION DE PRODUITS

MODULE B1 : DEPISTAGE

B.1 A quelle date avez-vous eu votre premier test VIH positif ?

Mois : /__/__/
Année : /__/__/__/__/

B.2 Comment avez-vous été infecté(e) par le VIH?
☐1 Par transmission maternelle (à la naissance ou par allaitement) (n'afficher que si année du premier test positif – année de naissance < 15)
☐2 Par transfusion
☐3 Par des produits anti-hémophiliques
☐4 Par relations sexuelles avec un homme
☐5 Par relations sexuelles avec une femme
☐6 Par injection de drogues
☐7 Autrement (préciser)
☐ 97. Non concerné (ne pas suggérer)
☐ 98. Ne sait pas (ne pas suggérer)
☐ 99. Refus de réponse (ne pas suggérer)

Si infection par relations sexuelles ou Autrement B2=4 ou 5 ou 7 :
B.2_bis. A cette époque, vous est-il arrivé d'avoir des rapports sexuels forcés avec ce partenaire ?
☐1Oui
☐2Non
☐ 97. Non concerné (ne pas suggérer)
☐ 98. Ne sait pas (ne pas suggérer)
☐ 99. Refus de réponse (ne pas suggérer)

B.3 A l'époque de votre diagnostic d'infection à VIH, consommiez-vous des drogues par injection ? Exclu
UNE SEULE REPONSE POSSIBLE

B.4
☐ 1 Oui
☐ 2 Non, mais vous l'avez fait avant, même une seule fois
☐ 3 Non et vous n'en aviez jamais consommé
☐ 97. Non concerné (ne pas suggérer)
☐ 98. Ne sait pas (ne pas suggérer)
☐ 99. Refus de réponse (ne pas suggérer)

B.5 Quelle est la personne à laquelle vous avez annoncé <u>en premier</u> votre séropositivité ?
UNE SEULE REPONSE POSSIBLE

☐ 1 Votre conjoint/partenaire principal (vous viviez ensemble à ce moment là)
☐ 2 Votre partenaire (si vous ne viviez pas ensemble à ce moment là)
☐ 3 Votre mère
☐ 4 Votre père
☐ 5 Vos deux parents ensemble
☐ 6 Votre/un de vos enfants
☐ 7 Votre/un de vos frères
☐ 8 Votre/une de vos sœurs
☐ 9 Un(e) ami(e) proche (y compris collègue de travail)
☐ 10 Un membre d'une association
☐ 11 Une autre personne : Qui ? **(à préciser)**
☐ 12 Ne l'a annoncé à personne
☐ 97. Non concerné (ne pas suggérer)
☐ 98. Ne sait pas (ne pas suggérer)
☐ 99. Refus de réponse (ne pas suggérer)

B.6 Où pensez-vous avoir été contaminé par le virus du sida ?
☐ 1 En France
☐ 2 A l'occasion d'un séjour ou d'un voyage dans un autre pays
☐ 3 Dans le pays où vous viviez avant de venir en France
☐ 98. Vous ne savez pas **(citer)**
☐ 97. Non concerné (ne pas suggérer)
☐ 99. Refus de réponse (ne pas suggérer)

<u>Pour les personnes dépistées depuis moins de 36 mois. Filtrer sur B1 selon la date de l'enquête</u>

B.7 Où avez-vous fait le dépistage de votre infection à VIH ?
UNE SEULE REPONSE POSSIBLE
☐ 1 En France
☐ 2 Dans un autre pays
☐ 97. Non concerné (ne pas suggérer)
☐ 98. Ne sait pas (ne pas suggérer)
☐ 99. Refus de réponse (ne pas suggérer)

B.8 Lorsque le test a été réalisé, c'était…
☐ 1 De votre propre initiative
☐ 2 Le médecin vous l'a proposé
☐ 3 A la demande de votre partenaire/conjoint
☐ 4 A votre insu, sans avoir été averti avant
☐ 5 A l' occasion d'un bilan chirurgical
☐ 97. Non concerné (ne pas suggérer)
☐ 98. Ne sait pas (ne pas suggérer)
☐ 99. Refus de réponse (ne pas suggérer)

B.9 Après le diagnostic, combien de temps s'est écoulé avant que vous alliez voir un médecin pour vous faire suivre pour le VIH ?
- ☐ 1 Moins d'un mois
- ☐ 2 Entre un mois et 6 mois
- ☐ 3 Entre 6 mois et un an
- ☐ 4 Plus d'un an
- ☐ 97. Non concerné (ne pas suggérer)
- ☐ 98. Ne sait pas (ne pas suggérer)
- ☐ 99. Refus de réponse (ne pas suggérer)

B.12 Faisiez-vous des études ?
- ☐ 1 Oui
- ☐ 2 Non
- ☐ 97. Non concerné (ne pas suggérer)
- ☐ 98. Ne sait pas (ne pas suggérer)
- ☐ 99. Refus de réponse (ne pas suggérer)

B.13 Concernant votre situation professionnelle au moment où vous avez appris votre infection à VIH
- ☐ 1 Vous aviez un emploi/activité déclaré
- ☐ 2 Vous aviez une activité non déclarée ou vous faisiez des petits boulots
- ☐ 3 Vous étiez en formation rémunérée
- ☐ 4 Vous aviez déjà travaillé dans le passé mais vous n'aviez pas d'emploi à ce moment là
- ☐ 5. Vous étiez au chômage
- ☐ 6. Vous étiez retraité / préretraité
- ☐ 7 Vous étiez en invalidité
- ☐ 8 Vous n'aviez jamais travaillé
- ☐ 9 Autre situation (à préciser)
- ☐ 97. Non concerné (ne pas suggérer) *item exclusif*
- ☐ 98. Ne sait pas (ne pas suggérer) *item exclusif*
- ☐ 99. Refus de réponse (ne pas suggérer) *item exclusif*

Poser B 14 Si B1<36 mois et si B13=4, 5, 6, 7 ou 8
B.14 Au moment de votre diagnostic, aviez-vous un revenu personnel, *c'est-à-dire de l'argent que vous obteniez de votre travail ou d'allocations (RMI, allocation chômage, etc.)* et que vous pouviez utiliser comme vous l'entendiez ?
- ☐ 1 Oui
- ☐ 2 Non, vous n'aviez aucune ressource personnelle
- ☐ 3 Non, mais vous bénéficiez des revenus de votre conjoint
- ☐ 97. Non concerné (ne pas suggérer)
- ☐ 98. Ne sait pas (ne pas suggérer)
- ☐ 99. Refus de réponse (ne pas suggérer)

Nous allons maintenant parler de votre vie depuis le diagnostic de votre infection par le virus du sida.
Depuis le diagnostic de votre infection par le virus du sida…

Si A3 ≠ 0 ➔B15
Si A3=0 et si A1 = 2 ➔aller directement à B16
Si A3=0 et si A1= 1 ➔aller directement à B17

Si A3 ≠ 0 ➔ B15
B.15 Avez-vous vécu séparé de votre/vos enfants ?
- ☐ 1 Oui
- ☐ 2 Non
- ☐ 97. Non concerné (ne pas suggérer)
- ☐ 98. Ne sait pas (ne pas suggérer)
- ☐ 99. Refus de réponse (ne pas suggérer)

Si A1 = 2 (femme) ➔ B16
B.16 Avez-vous fait une IVG « (Interruption Volontaire de Grossesse) »?
- ☐ 1 Oui
- ☐ 2 Non
- ☐ 97. Non concerné (ne pas suggérer)
- ☐ 98. Ne sait pas (ne pas suggérer)
- ☐ 99. Refus de réponse (ne pas suggérer)

A toutes les personnes dépistées depuis moins de 36 mois
B.18 Avez-vous eu une période d'au moins 6 mois sans avoir des relations sexuelles ?
- ☐ 1 Oui
- ☐ 2 Non
- ☐ 97. Non concerné (ne pas suggérer)
- ☐ 98. Ne sait pas (ne pas suggérer)
- ☐ 99. Refus de réponse (ne pas suggérer)

Si les personnes avaient un emploi au moment du diagnostic, B13 = 1 ou 2 ➔ B19 et B 20
B.19 Avez-vous perdu votre emploi ?
- ☐ 1 Oui
- ☐ 2 Non
- ☐ 97. Non concerné (ne pas suggérer)
- ☐ 98. Ne sait pas (ne pas suggérer)
- ☐ 99. Refus de réponse (ne pas suggérer)

B.20 Avez-vous eu un/des congés de maladie de plus d'un mois ?
- ☐ 1 Oui
- ☐ 2 Non
- ☐ 97. Non concerné (ne pas suggérer)
- ☐ 98. Ne sait pas (ne pas suggérer)
- ☐ 99. Refus de réponse (ne pas suggérer)

Si B12 = 1 ➔ B21

B.21 Avez-vous arrêté vos études ?

☐ 1 Oui
☐ 2 Non
☐ 97. Non concerné (ne pas suggérer)
☐ 98. Ne sait pas (ne pas suggérer)
☐ 99. Refus de réponse (ne pas suggérer)

MODULE B2 : PRISE EN CHARGE MEDICALE

POUR TOUT LE MONDE

B22b. Au cours des 3 dernières années, vous est-il arrivé de passer plus d'1 an sans voir un médecin pour le suivi de votre VIH ?
☐ 1 Oui
☐ 2 Non
☐ 97. Non concerné (ne pas suggérer)
☐ 98. Ne sait pas (ne pas suggérer)
☐ 99. Refus de réponse (ne pas suggérer)

B.23 Au cours des 12 derniers mois, avez-vous été hospitalisé(e) au moins une nuit, quelle que soit la raison ?
UNE SEULE REPONSE POSSIBLE
☐ 1 Oui ➔ **B24**
☐ 2 Non ➔ **B25**
☐ 97. Non concerné (ne pas suggérer)
☐ 98. Ne sait pas (ne pas suggérer)
☐ 99. Refus de réponse (ne pas suggérer)

Si B23=1➔ B24
B.24 Combien de fois au cours des 12 derniers mois ? |___|___| en nombre de fois

B.24.1 Quelle a été la durée de cette hospitalisation / de la 1ère de ces hospitalisations ? |___|___| jours

B.24.2 Quelle a été la durée de la 2nde de ces hospitalisations ? |___|___| jours

B.24.3 Quelle a été la durée de la 3ème de ces hospitalisations ? |___|___| jours

B.24.4 Quelle a été la durée de la 4ème de ces hospitalisations ? |___|___| jours

B.24.5 Quelle a été la durée de la 5ème de ces hospitalisations ? |___|___| jours

B.24.6 Quelle a été la durée de la 6ème de ces hospitalisations ? |___|___| jours

B.24.7 Quelle a été la durée de la 7ème de ces hospitalisations ? |___|___| jours

B.24.8 Quelle a été la durée de la 8ème de ces hospitalisations ? |___|___| jours

B.24.9 Quelle a été la durée de la 9ème de ces hospitalisations ? |___|___| jours

Etc. pour chacune des hospitalisations (en fonction du nombre de fois en B24)

Si B24 est supérieur à 5 fois poser B24Bis
B24Bis. Au total, combien de jours avez-vous été hospitalisé(e) dans l'année ?

|___|___| **Jours**

B.25 Vous êtes suivi(e) pour le VIH ?
UNE SEULE REPONSE POSSIBLE. SI C'EST LE MEME MEDECIN QUI SUIT LE SUJET A L'HOPITAL ET DANS UN CABINET EN VILLE, COCHER « HOPITAL »
Inverser l'ordre des deux premières modalités
☐ 1 Principalement, dans cet hôpital
☐ 2 Principalement, dans un cabinet de médecine en ville
☐ 3 Principalement, dans un autre hôpital
☐ 4 Autant en ville qu'à l'hôpital
☐ 97. Non concerné (ne pas suggérer)
☐ 98. Ne sait pas (ne pas suggérer)
☐ 99. Refus de réponse (ne pas suggérer)

B.25 bis Durant les deux dernières années, avez-vous changé d'hôpital pour votre suivi VIH ?
☐ 1 Oui
☐ 2 Non
☐ 97. Non concerné (ne pas suggérer)
☐ 98. Ne sait pas (ne pas suggérer)
☐ 99. Refus de réponse (ne pas suggérer)

Si B25 bis =1
B.25ter Etait-ce parce que ?
☐ 1 Vous avez déménagé
☐ 2 Votre médecin a changé d'hôpital
☐ 3 Le service dans lequel vous étiez suivi a fermé
☐ 4 Pour une autre raison
☐ 97. Non concerné (ne pas suggérer)
☐ 98. Ne sait pas (ne pas suggérer)
☐ 99. Refus de réponse (ne pas suggérer)

B.26 Tous les combien êtes-vous venu(e) consulter le médecin qui vous suit pour le VIH dans ce service <u>au cours des 12 derniers mois</u> –hors hospitalisation classique- ?
UNE SEULE REPONSE POSSIBLE
☐ 1. Une fois dans l'année
☐ 2. Tous les 6 mois
☐ 3. Tous les 4 mois
☐ 4. Tous les 3 mois
☐ 5. Tous les 2 mois
☐ 6. Tous les mois
☐ 7. Plus souvent
☐ 97. Non concerné (ne pas suggérer)

☐ 98. Ne sait pas (ne pas suggérer)
☐ 99. Refus de réponse (ne pas suggérer)

Si B26= 97 ou 98 ou 99 poser B26.1
B26.1 Combien de consultations avez-vous eu avec ce médecin au cours des 12 derniers mois y compris ce jour :

/__/__/__/ jours

B.28 Est-ce que vous prenez actuellement un traitement antirétroviral ?
UNE SEULE REPONSE POSSIBLE

☐ 1. Oui
☐ 2. Non, pas actuellement mais vous en avez déjà reçu un traitement dans le passé ➔ **B37**
☐ 3. Non, pas actuellement et vous n'avez jamais reçu de traitement ➔ **B37**
☐ 97. Non concerné (ne pas suggérer)
☐ 98. Ne sait pas (ne pas suggérer)
☐ 99. Refus de réponse (ne pas suggérer)
Si B28= 1 (la personne prend actuellement un traitement antirétroviral

Nous allons maintenant parler de ce qui s'est passé le dernier mois
B.30 Au cours du dernier mois, vous est-il arrivé d'interrompre volontairement votre traitement pendant plusieurs jours ?
UNE SEULE REPONSE POSSIBLE
☐ 1 Jamais
☐ 2 Oui, une fois
☐ 3 Oui, plusieurs fois
☐ 97. Non concerné (ne pas suggérer)
☐ 98. Ne sait pas (ne pas suggérer)
☐ 99. Refus de réponse (ne pas suggérer)

B.31 Au cours du dernier mois, vous est-il arrivé d'oublier ou de sauter une prise ?
UNE SEULE REPONSE POSSIBLE
☐ 1 Jamais
☐ 2 Rarement
☐ 3 Souvent
☐ 97. Non concerné (ne pas suggérer)
☐ 98. Ne sait pas (ne pas suggérer)
☐ 99. Refus de réponse (ne pas suggérer)

B.32 Au cours du dernier mois, vous est-il arrivé de décaler une (ou plusieurs) prise(s) de plus de deux heures ?
UNE SEULE REPONSE POSSIBLE
☐ 1Jamais
☐ 2 Rarement
☐ 3 Souvent
☐ 97. Non concerné (ne pas suggérer)
☐ 98. Ne sait pas (ne pas suggérer)
☐ 99. Refus de réponse (ne pas suggérer)

B.33 Globalement, durant le dernier mois, vous diriez que vous avez suivi votre traitement antirétroviral ?
UNE SEULE REPONSE POSSIBLE
☐ 1 Très mal
☐ 2 Mal
☐ 3 Assez mal
☐ 4 Assez bien
☐ 5 Presque parfaitement
☐ 6 Parfaitement
☐ 97. Non concerné (ne pas suggérer)
☐ 98. Ne sait pas (ne pas suggérer)
☐ 99. Refus de réponse (ne pas suggérer)

B.34 Pour vous, actuellement, les effets désagréables de votre traitement sont…
UNE SEULE REPONSE POSSIBLE
☐ 1. Inexistants
☐ 2. Pas du tout gênants
☐ 3. Peu gênants
☐ 4. Assez gênants
☐ 5. Très gênants
☐ 97. Non concerné (ne pas suggérer)
☐ 98. Ne sait pas (ne pas suggérer)
☐ 99. Refus de réponse (ne pas suggérer)

POUR TOUT LE MONDE

B.37 Actuellement quelle sécurité sociale avez-vous ?
☐ 1 Le régime général, un régime spécial ou assimilé
☐ 2 La CMU
☐ 3 L'AME
☐ 4 Autres
☐ 97. Non concerné (ne pas suggérer)
☐ 98. Ne sait pas (ne pas suggérer)
☐ 99. Refus de réponse (ne pas suggérer)

B.37.1 Avez-vous une mutuelle ou une assurance complémentaire ?
☐ 1 Oui
☐ 2 Non
☐ 97. Non concerné (ne pas suggérer)
☐ 98. Ne sait pas (ne pas suggérer)
☐ 99. Refus de réponse (ne pas suggérer)

B.37.2 Avez-vous désigné un médecin traitant/référent à la sécurité sociale ?
☐ 1 Oui ➔ **B37.3**
☐ 2 Non➔ **B38**
☐ 97. Non concerné (ne pas suggérer)
☐ 98. Ne sait pas (ne pas suggérer)
☐ 99. Refus de réponse (ne pas suggérer)

Si B37.2=1
B.37.3 Ce médecin est-il ?
UNE SEULE REPONSE POSSIBLE
☐1 un médecin généraliste en ville
☐2 le médecin qui vous suit pour le VIH à l'hôpital
☐3 Un médecin qui vous suit à la fois en ville et à l'hôpital
☐4 un autre médecin
☐ 97. Non concerné (ne pas suggérer)
☐ 98. Ne sait pas (ne pas suggérer)
☐ 99. Refus de réponse (ne pas suggérer)

B.38 Au cours des 12 derniers mois, combien de fois avez-vous consulté un médecin généraliste quelle que soit la raison pour laquelle vous avez consulté ?

/__/__/ fois

Si B38=97,98 ou 99 poser B38 Bis
B38bis Avez-vous consulté un médecin généraliste quelle que soit la raison pour laquelle vous avez consulté :
☐ 1. Jamais➔ **B41**
☐ 2. Une fois dans l'année
☐ 3. Tous les 6 mois
☐ 4. Tous les 4 mois
☐ 5. Tous les 3 mois
☐ 6. Tous les mois
☐ 7. Plus souvent
☐ 97. Non concerné (ne pas suggérer)
☐ 98. Ne sait pas (ne pas suggérer)
☐ 99. Refus de réponse (ne pas suggérer)

Si (B38 ≠ 0 et B38< 97) ou si B38bis > 1 ➔ poser B39

B.39 Est-il au courant de votre séropositivité ?
☐ 1 Oui ➔ **B40**
☐ 2 Non ➔ **B41**
☐ 97. Non concerné (ne pas suggérer)
☐ 98. Ne sait pas (ne pas suggérer)
☐ 99. Refus de réponse (ne pas suggérer)

Si B39=1➔ poser B40
B.40 Au cours des 12 derniers mois, vous a-t-il fait ou renouvelé une ordonnance pour votre traitement VIH ?
☐ 1 Oui
☐ 2 Non
☐ 97. Non concerné (ne pas suggérer)
☐ 98. Ne sait pas (ne pas suggérer)
☐ 99. Refus de réponse (ne pas suggérer)

POUR TOUT LE MONDE
B.41 <u>Au cours des 12 derniers mois,</u> êtes vous allé chez le dentiste ?
☐ 1 Oui ➔ **B42**
☐ 2 Non ➔ **B43**
☐ 97. Non concerné (ne pas suggérer)
☐ 98. Ne sait pas (ne pas suggérer)
☐ 99. Refus de réponse (ne pas suggérer)

Si B41=1➔ B42
B.42 Ce dentiste est-il au courant de votre séropositivité ?
☐ 1 Oui
☐ 2 Non
☐ 97. Non concerné (ne pas suggérer)
☐ 98. Ne sait pas (ne pas suggérer)
☐ 99. Refus de réponse (ne pas suggérer)

POUR TOUT LE MONDE
B.43 Est-il déjà arrivé qu'un dentiste refuse de vous soigner à cause de votre séropositivité?
☐ 1. Oui ➔ **B44**
☐ 2. Non➔ **B45**
☐ 97. Non concerné (ne pas suggérer)
☐ 98. Ne sait pas (ne pas suggérer)
☐ 99. Refus de réponse (ne pas suggérer)

Si B43=1
B.44 Est- ce arrivé au cours des 2 dernières années ?
☐ 1 Oui
☐ 2 Non
☐ 97. Non concerné (ne pas suggérer)
☐ 98. Ne sait pas (ne pas suggérer)
☐ 99. Refus de réponse (ne pas suggérer)

POUR TOUT LE MONDE

B.45 Avez-vous ou avez-vous eu un ou des problèmes de santé chroniques autres que le VIH ?
☐ $_{1}$ Oui
☐ $_{2}$ Non
☐ $_{97}$. Non concerné (ne pas suggérer)
☐ $_{98}$. Ne sait pas (ne pas suggérer)
☐ $_{99}$. Refus de réponse (ne pas suggérer)

Actuellement, êtes-vous régulièrement suivi par
B.46 un (e) gastro-entérologue ou un proctologue?
☐ $_{1}$ Oui
☐ $_{2}$ Non
☐ $_{97}$. Non concerné (ne pas suggérer)
☐ $_{98}$. Ne sait pas (ne pas suggérer)
☐ $_{99}$. Refus de réponse (ne pas suggérer)

B.47 un(e) hépatologue ?
☐ $_{1}$ Oui
☐ $_{2}$ Non
☐ $_{97}$. Non concerné (ne pas suggérer)
☐ $_{98}$. Ne sait pas (ne pas suggérer)
☐ $_{99}$. Refus de réponse (ne pas suggérer)

B.48 un(e) endocrinologue ou un diabétologue ?
☐ $_{1}$ Oui
☐ $_{2}$ Non
☐ $_{97}$. Non concerné (ne pas suggérer)
☐ $_{98}$. Ne sait pas (ne pas suggérer)
☐ $_{99}$. Refus de réponse (ne pas suggérer)

B.49 un(e) cardiologue ?
☐ $_{1}$ Oui
☐ $_{2}$ Non
☐ $_{97}$. Non concerné (ne pas suggérer)
☐ $_{98}$. Ne sait pas (ne pas suggérer)
☐ $_{99}$. Refus de réponse (ne pas suggérer)

B.50 un(e) psychiatre ?
☐ $_{1}$ Oui
☐ $_{2}$ Non
☐ $_{97}$. Non concerné (ne pas suggérer)
☐ $_{98}$. Ne sait pas (ne pas suggérer)
☐ $_{99}$. Refus de réponse (ne pas suggérer)

B.51 un(e) neurologue ?
☐ $_{1}$ Oui
☐ $_{2}$ Non
☐ $_{97}$. Non concerné (ne pas suggérer)
☐ $_{98}$. Ne sait pas (ne pas suggérer)
☐ $_{99}$. Refus de réponse (ne pas suggérer)

B.52 un(e) rhumatologue ?
☐ $_{1}$ Oui
☐ $_{2}$ Non
☐ $_{97}$. Non concerné (ne pas suggérer)
☐ $_{98}$. Ne sait pas (ne pas suggérer)
☐ $_{99}$. Refus de réponse (ne pas suggérer)

B.53 un(e) cancérologue (oncologue) ?
☐ $_{1}$ Oui
☐ $_{2}$ Non
☐ $_{97}$. Non concerné (ne pas suggérer)
☐ $_{98}$. Ne sait pas (ne pas suggérer)
☐ $_{99}$. Refus de réponse (ne pas suggérer)

Si A1=2 ou A1=3 (la répondante est une femme ou transgenre) ➔ B54
B.54 un (e) gynécologue ?
☐ $_{1}$ Oui
☐ $_{2}$ Non
☐ $_{97}$. Non concerné (ne pas suggérer)
☐ $_{98}$. Ne sait pas (ne pas suggérer)
☐ $_{99}$. Refus de réponse (ne pas suggérer)

POUR TOUT LE MONDE
Ne pas poser si femme A1=2 et A2 femme née avant 1961
B55 bis : Actuellement, est ce que vous ou votre partenaire utilisez un moyen pour éviter une grossesse ?
PLUSIEURS REPONSES POSSIBLES
☐ $_{1}$ Oui
☐ $_{2}$ Non
☐ $_{3}$. Vous n'êtes pas concerné (Homosexuel, femme ménopausée, homme se déclarant trop âgé)
☐ $_{98}$. Ne sait pas (ne pas suggérer)
☐ $_{99}$. Refus de réponse (ne pas suggérer)

Ne pas poser si femme A1=2 et A2 femme née avant 1961
Si B55bis = 1 poser la question B55 Ter
B55Ter : Si oui, lequel ? "Merci de citer toutes les méthodes y compris les méthodes naturelles"

- ☐1 Pilule
- ☐2 Stérilet
- ☐3 Implant
- ☐4 Préservatif masculin
- ☐5 Retrait du partenaire avant l'éjaculation
- ☐6 Eviter les rapports sexuels les jours les plus à risque de grossesse (Ogino, températures, glaire, Billings, abstinence périodique...)
- ☐7 Diaphragme, cape cervical
- ☐8 Crèmes spermicides, ovules, éponge
- ☐9 Patch contraceptif
- ☐10 Anneau vaginal
- ☐11 Depoprovera/injection hormonale
- ☐12 Pilule du lendemain ou contraception d'urgence
- ☐13 Préservatif féminin
- ☐14 Abstinence
- ☐15 Ligature des trompes – stérilisation
- ☐16 Vasectomie
- ☐17 Aucune méthode ➔ **Item exclusif**
- ☐98 Ne sais pas ➔ **Item exclusif**
- ☐99 Ne veux pas répondre ➔ **Item exclusif**

Ne pas poser si femme A1=2 et A2 femme née avant 1965
Ne pas poser si B55= 3
Poser Si B55Bis = Non ou Ne sait pas ou Refus ou Refus OU si B55Ter=17 ou 98 ou 99 ➔poser la question B55 quater

B55quater : Pouvez-vous me dire si les phrases suivantes vous concernent vous ou votre partenaire actuellement ?

- ☐ 1 Oui
- ☐ 2 Non
- ☐ 97. Non concerné (ne pas suggérer)
- ☐ 98. Ne sait pas (ne pas suggérer)
- ☐ 99. Refus de réponse (ne pas suggérer)

B56.1 Vous n'avez pas de rapports sexuels en ce moment
B56.2 Vous souhaitez avoir un enfant
B56.3 vous ou votre partenaire vient d'accoucher ou allaite
B56.4 Vous évitez les rapports sexuels les jours à risque ou vous utilisez le retrait ou les préservatifs
B56.5 Aucune méthode contraceptive ne vous convient à vous ou votre partenaire
B56.6 Autres situations (à préciser)

Pour toutes les personnes de plus de 50 ans (A2> 50) ➔ B57
B.57 Avez-vous déjà passé un test recherchant du sang dans les selles (par exemple HémocultII®, Hémocheck, Hemo-Fec…)?

- ☐ 1. Oui ➔ **B58**
- ☐ 2. Non
- ☐ 97. Non concerné (ne pas suggérer)
- ☐ 98. Ne sait pas (ne pas suggérer)
- ☐ 99. Refus de réponse (ne pas suggérer)

Si B57=1➔ B58
B.58 La dernière fois, c'était il y a combien de temps (ou en quelle année) ?

B58.1 /__/__/ (préciser le nombre d'années) OU **B58.2** /__/__/__/__/ (préciser l'année)

Si B58=98 (ne sais pas) ➔poser la question suivante :
Était-ce il y a… ?
UNE SEULE REPONSE POSSIBLE

- ☐ 1 Moins de 2 ans
- ☐ 2 Entre 2 et 3 ans
- ☐ 3 Entre 3 ans et 5 ans
- ☐ 4 Plus de 5 ans
- ☐ 97. Non concerné (ne pas suggérer)
- ☐ 98. Ne sait pas (ne pas suggérer)
- ☐ 99. Refus de réponse (ne pas suggérer)

Si A1=2
B58.4 Etes- vous ménopausée?

- ☐ 1Oui,
- ☐ 2Non,
- ☐ 97. Non concerné (ne pas suggérer)
- ☐ 98. Ne sait pas (ne pas suggérer)
- ☐ 99. Refus de réponse (ne pas suggérer)

Si A1=2 et Si B58.4 =1
B58.5 Votre ménopause a-t-elle été confirmée par des dosages hormonaux:

- ☐ 1Oui,
- ☐ 2Non,
- ☐ 97. Non concerné (ne pas suggérer)
- ☐ 98. Ne sait pas (ne pas suggérer)
- ☐ 99. Refus de réponse (ne pas suggérer)

Poser B58.6 est Si A1=2 et Si B58.4 =1
B58.6 A quel âge avez-vous été ménopausée ?

|___|___| **ans**

Si B58.6 = Non concerné/NSP/Refus possible
B58.7 c'était il y a... ?
UNE SEULE REPONSE POSSIBLE
☐ 1 1 an ou 2,
☐ 2 Entre 2 ans et 5 ans,
☐ 3 Entre de 5 ans et 10 ans,
☐ 4 Plus de 10 ans
☐ 97. Non concerné (ne pas suggérer)
☐ 98. Ne sait pas (ne pas suggérer)
☐ 99. Refus de réponse (ne pas suggérer)

Si A1=2 (la répondante est une femme) ➔ B59
B.59 Avez-vous déjà passé une mammographie (radiographie des seins)?
☐ 1. Oui ➔ B60
☐ 2. Non ➔ B62
☐ 97. Non concerné (ne pas suggérer) ➔ B62
☐ 98. Ne sait pas (ne pas suggérer) ➔ B62
☐ 99. Refus de réponse (ne pas suggérer) ➔ B62

Si B59=1➔ B60
B.60 La dernière fois, c'était il y a combien de temps (ou en quelle année)?
☐ 1 /__/__/ (préciser le nombre d'années) OU B62b /__/__/__/__/ (préciser l'année) ➔ B62
☐ 2. Ne sais pas mais la personne interrogée dit suivre les recommandations de son praticien ou être à jour dans ses examens➔ B62
☐ 97. Non concerné (ne pas suggérer)
☐ 98. Ne sait pas (ne pas suggérer) ➔ B61
☐ 99. Refus de réponse (ne pas suggérer) ➔ B61

Si B60=98, 99 (ne sais pas) ➔ B61
B.61 Si NSP, Était-ce il y a… ?
☐ 1 Moins de 2 ans
☐ 2 Entre 2 et 3 ans
☐ 3 Entre 3 ans et 5 ans
☐ 4 Plus de 5 ans
☐ 97. Non concerné (ne pas suggérer)
☐ 98. Ne sait pas (ne pas suggérer)
☐ 99. Refus de réponse (ne pas suggérer)

Si A1=2
B.62 Avez-vous déjà fait un frottis du col de l'utérus (vaginal) ?
☐ 1. Oui ➔ B63
☐ 2. Non ➔ B65
☐ 97. Non concerné (ne pas suggérer) ➔ B65
☐ 98. Ne sait pas (ne pas suggérer) ➔ B65
☐ 99. Refus de réponse (ne pas suggérer) ➔ B65

Si B62=1➔ B63
B.63 La dernière fois, c'était il y a combien de temps (ou en quelle année)?
☐ 1 /__/__/ (nombre d'années) OU B65b /__/__/__/__/ (préciser l'année) ➔ B65
☐ 2. Ne sais pas mais la personne interrogée dit suivre les recommandations de son praticien ou être à jour dans ses examens➔ B65
☐ 97. Non concerné (ne pas suggérer)
☐ 98. Ne sait pas (ne pas suggérer) ➔ B64
☐ 99. Refus de réponse (ne pas suggérer) ➔ B64

Si B63=98 ou 99 (ne sais pas)➔ B64
B.64 Si NSP, Était-ce il y a… ?
☐ 1 Moins de 2 ans
☐ 2 Entre 2 et 5 ans
☐ 3 Plus de 5 ans
☐ 97. Non concerné (ne pas suggérer)
☐ 98. Ne sait pas (ne pas suggérer)
☐ 99. Refus de réponse (ne pas suggérer)

POUR TOUT LE MONDE
Il arrive de rencontrer des difficultés pour se faire soigner

B.65 <u>Dans les 12 derniers mois</u>, vous est-il arrivé de renoncer à certains soins de santé pour des raisons financières ?
☐ 1. Oui
☐ 2. Non
☐ 97. Non concerné (ne pas suggérer)
☐ 98. Ne sait pas (ne pas suggérer)
☐ 99. Refus de réponse (ne pas suggérer)

Au <u>cours des deux dernières années</u>…

B.67 Est-il arrivé qu'un médecin de ville refuse de vous soigner ?
☐ 1. Oui
☐ 2. Non
☐ 97. Non concerné (ne pas suggérer)
☐ 98. Ne sait pas (ne pas suggérer)
☐ 99. Refus de réponse (ne pas suggérer)

B.68 Au cours des deux dernières années, est-il arrivé qu'on refuse de vous soigner dans un hôpital ?
☐ 1. Oui
☐ 2. Non
☐ 97. Non concerné (ne pas suggérer)
☐ 98. Ne sait pas (ne pas suggérer)
☐ 99. Refus de réponse (ne pas suggérer)

B.69 Au cours des deux dernières années, est-il arrivé qu'un médecin ou du personnel médical vous traite moins bien ou vous reçoive plus mal que les autres patients ?

☐ 1. Oui
☐ 2. Non
☐ 97. Non concerné (ne pas suggérer)
☐ 98. Ne sait pas (ne pas suggérer)
☐ 99. Refus de réponse (ne pas suggérer)

Si B67=1 ou B68=1 ou B69=1 ➔ B70

B.70 Pensez-vous que c'était à cause de …

PLUSIEURS REPONSES POSSIBLES

☐1 Vos origines ou votre nationalité
☐2 Votre couleur de peau
☐3 Votre sexe (le fait d'être un homme ou une femme)
☐4 Votre orientation sexuelle
☐5 Votre séropositivité
☐6 Votre usage d'alcool
☐7 Votre usage de drogue actuel ou passé
☐8 Votre façon de vous habiller
☐9 Le lieu ou vous vivez, la réputation de votre quartier
☐10 Parce que vous êtes à la CMU ou à l'AME (pour soins)
☐11 Autre (à préciser)
☐ 97. Non concerné (ne pas suggérer)
☐ 98. Ne sait pas (ne pas suggérer)
☐ 99. Refus de réponse (ne pas suggérer)

MODULE B3 : CONNAISSANCE SUR LA MALADIE

POUR TOUT LE MONDE

B.71 Concernant votre maladie, vous vous sentez ?
UNE SEULE REPONSE POSSIBLE

- ☐ 1. Bien informé
- ☐ 2. Assez informé
- ☐ 3. Mal informé
- ☐ 4. Pas informé du tout
- ☐ 97. Non concerné (ne pas suggérer)
- ☐ 98. Ne sait pas (ne pas suggérer)
- ☐ 99. Refus de réponse (ne pas suggérer)

Quand on est suivi pour une maladie, on a souvent besoin de lire des documents, des ordonnances ou d'utiliser des données chiffrées. Je vais maintenant vous poser des questions sur vos aptitudes à lire, à écrire et à calculer.

B.73 Dans votre vie quotidienne, comment évalueriez-vous vos aptitudes à lire ? Diriez-vous qu'elles sont…
UNE SEULE REPONSE POSSIBLE
- ☐ 1 Excellentes
- ☐ 2 Très bonnes
- ☐ 3 Bonnes
- ☐ 4 Moyennes
- ☐ 5 Insuffisantes
- ☐ 97. Non concerné (ne pas suggérer)
- ☐ 98. Ne sait pas (ne pas suggérer)
- ☐ 99. Refus de réponse (ne pas suggérer)

B.74 Dans votre vie quotidienne, comment évalueriez-vous vos aptitudes à écrire ? Diriez-vous qu'elles sont…
UNE SEULE REPONSE POSSIBLE
- ☐ 1 Excellentes
- ☐ 2 Très bonnes
- ☐ 3 Bonnes
- ☐ 4 Moyennes
- ☐ 5 Insuffisantes
- ☐ 97. Non concerné (ne pas suggérer)
- ☐ 98. Ne sait pas (ne pas suggérer)
- ☐ 99. Refus de réponse (ne pas suggérer)

Maintenant quelques questions sur les chiffres et les calculs

B.75 Si le risque de contracter une maladie est de 10 pour cent, sur 1 000 personnes, combien risquent d'attraper cette maladie ?
- ☐ 1 100 ➔ B77
- ☐ 2 10 ➔ B76
- ☐ 3 90 ➔ B76
- ☐ 4 900 ➔ B76
- ☐ 5 Autre réponse ➔ B76
- ☐ 97. Non concerné (ne pas suggérer) ➔ B76
- ☐ 98. Ne sait pas (ne pas suggérer) ➔ B76
- ☐ 99. Refus de réponse (ne pas suggérer) ➔ B76

B.76 En période de soldes, un magasin vend tous ses articles à moitié prix. Avant les soldes, un canapé coûte 300 Euros. Combien vaudra-t-il en soldes ?
- ☐ 1 150 euros
- ☐ 2 600 euros
- ☐ 3 Autre réponse
- ☐ 97. Non concerné (ne pas suggérer)
- ☐ 98. Ne sait pas (ne pas suggérer)
- ☐ 99. Refus de réponse (ne pas suggérer)

Si B75= 1 ➔ B 77 et B78
B.77 Un vendeur de voitures d'occasion vend une voiture à 6 000 euros. Ce prix représente les deux tiers du prix de la voiture neuve. Combien valait la voiture neuve ?
- ☐ 1 9 000 euros
- ☐ 2 4 000 euros
- ☐ 3 8 000 euros
- ☐ 4 12 000 euros
- ☐ 5 18 000 euros
- ☐ 6 Autre réponse
- ☐ 97. Non concerné (ne pas suggérer)
- ☐ 98. Ne sait pas (ne pas suggérer)
- ☐ 99. Refus de réponse (ne pas suggérer)

B.78 Supposons que vous ayez 2000 euros sur un compte d'épargne. Ce compte vous rapporte un intérêt de 10 pour cent par an. Combien aurez-vous sur votre compte au bout de 2 ans ?
- ☐ 1 2 420 euros
- ☐ 2 2 020 euros
- ☐ 3 2 040 euros
- ☐ 4 2 100 euros
- ☐ 5 2 200 euros
- ☐ 6 2 400 euros
- ☐ 7 Autre réponse
- ☐ 97. Non concerné (ne pas suggérer)
- ☐ 98. Ne sait pas (ne pas suggérer)
- ☐ 99. Refus de réponse (ne pas suggérer)

POUR TOUT LE MONDE

Les examens de sang qui vous sont faits régulièrement donnent votre taux de CD4 et votre charge virale.

B.80 Le but du traitement est-il de faire monter ou descendre le taux de CD4 ?

- ☐ $_1$ Monter
- ☐ $_2$ Descendre
- ☐ $_{97}$. Non concerné (ne pas suggérer)
- ☐ $_{98}$. Ne sait pas (ne pas suggérer)
- ☐ $_{99}$. Refus de réponse (ne pas suggérer)

B.82 Le but du traitement est-il de faire monter ou descendre la charge virale ?

- ☐ $_1$ Monter
- ☐ $_2$ Descendre
- ☐ $_{97}$. Non concerné (ne pas suggérer)
- ☐ $_{98}$. Ne sait pas (ne pas suggérer)
- ☐ $_{99}$. Refus de réponse (ne pas suggérer)

MODULE B4 : CONSOMMATION DE PRODUITS

POUR TOUT LE MONDE

Nous allons maintenant aborder votre consommation de produits, en commençant par le tabac.

B.83 Est-ce que vous fumez, ne serait-ce que de temps en temps ?
☐ 1 Oui ➔ **B84**
☐ 2 Non ➔ **B86**
☐ 97. Non concerné (ne pas suggérer)
☐ 98. Ne sait pas (ne pas suggérer)
☐ 99. Refus de réponse (ne pas suggérer)

Si B83 = 1 ➔ B84
B.84 Vous fumez…
UNE SEULE REPONSE POSSIBLE
☐ 1 Tous les jours➔ **B85**
☐ 2 Moins souvent➔ **B90**
☐ 97. Non concerné (ne pas suggérer)
☐ 98. Ne sait pas (ne pas suggérer)
☐ 99. Refus de réponse (ne pas suggérer)

Si B84 = 1 ➔ B85
B.85 En moyenne, combien de cigarettes fumez-vous par jour ?
UNE SEULE REPONSE POSSIBLE
☐ 1 1 à 5 cigarettes
☐ 2 6 à 10 cigarettes
☐ 3 11 à 20 cigarettes
☐ 4 Plus de 20 cigarettes par jour (plus d'un paquet)
☐ 5 Autre (à préciser) :
☐ 97. Non concerné (ne pas suggérer)
☐ 98. Ne sait pas (ne pas suggérer)
☐ 99. Refus de réponse (ne pas suggérer)

Si B83=2 ➔ B86
B.86 Au cours de votre vie, avez-vous déjà fumé ?
UNE SEULE REPONSE POSSIBLE
☐ 1 Non, jamais ➔ **B90**
☐ 2 Oui, juste pour essayer➔ **B90**
☐ 3 Oui, occasionnellement, ou quotidiennement MAIS pendant MOINS de 6 mois➔ **B88**
☐ 4 Oui, quotidiennement pendant AU MOINS 6 mois ➔ **B87 puis B88**
☐ 97. Non concerné (ne pas suggérer)
☐ 98. Ne sait pas (ne pas suggérer)
☐ 99. Refus de réponse (ne pas suggérer)

Si B84=1 ou B86=4➔ B87
B.87 A quel âge avez-vous commencé à fumer quotidiennement ? /______/ ans

Si B86=3 ou 4➔ B88
B.88 A quel âge avez-vous arrêté de fumer ? /_______/ ans

Si B84=1➔ B89
B.89 Le matin, combien de temps après votre réveil fumez-vous votre première cigarette ?
UNE SEULE REPONSE POSSIBLE
☐ 1 Dans les 5 premières minutes
☐ 2 Entre 6 et 30 minutes
☐ 3 Après 30 minutes
☐ 97. Non concerné (ne pas suggérer)
☐ 98. Ne sait pas (ne pas suggérer)
☐ 99. Refus de réponse (ne pas suggérer)

Nous allons maintenant parler de votre consommation d'alcool

B.90 Quelle est la fréquence de votre consommation d'alcool ?
UNE SEULE REPONSE POSSIBLE
☐ 1 vous ne buvez jamais d'alcool ➔ **B100**
☐ 2 1 fois par mois ou moins
☐ 3 2 à 4 fois par mois
☐ 4 2 à 3 fois par semaine
☐ 5 Au moins 4 fois par semaine
☐ 97. Non concerné (ne pas suggérer)
☐ 98. Ne sait pas (ne pas suggérer)
☐ 99. Refus de réponse (ne pas suggérer)

B.91 Combien de verres contenant de l'alcool consommez-vous un jour typique où vous buvez ?
UNE SEULE REPONSE POSSIBLE
☐ 1 1 ou 2
☐ 2 3 ou 4
☐ 3 5 ou 6
☐ 4 7, 8 ou 9
☐ 5 10 ou plus
☐ 97. Non concerné (ne pas suggérer)
☐ 98. Ne sait pas (ne pas suggérer)
☐ 99. Refus de réponse (ne pas suggérer)

B.92 Avec quelle fréquence buvez-vous six verres ou davantage lors d'une occasion particulière ?
UNE SEULE REPONSE POSSIBLE
☐ 1 Jamais
☐ 2 Moins d'une fois par mois
☐ 3 Une fois par mois
☐ 4 Une fois par semaine
☐ 5 Tous les jours ou presque
☐ 97. Non concerné (ne pas suggérer)
☐ 98. Ne sait pas (ne pas suggérer)
☐ 99. Refus de réponse (ne pas suggérer)

Si B90 >2 ET si B91 >1 poser les questions de B93 à B99
Si B92 >1, poser les questions de B93 à B99
Dans les autres cas passer à B100

B.93 Au cours des douze derniers mois, combien de fois avez-vous constaté que vous n'étiez plus capable de vous arrêter de boire une fois que vous aviez commencé ?
UNE SEULE REPONSE POSSIBLE
☐ 1 Jamais
☐ 2 Moins d'une fois par mois
☐ 3 Une fois par mois
☐ 4 Une fois par semaine
☐ 5 Tous les jours ou presque
☐ 97. Non concerné (ne pas suggérer)
☐ 98. Ne sait pas (ne pas suggérer)
☐ 99. Refus de réponse (ne pas suggérer)

B.94 Au cours des douze derniers mois, combien de fois votre consommation d'alcool vous a-t-elle empêché de vous comporter normalement ?
UNE SEULE REPONSE POSSIBLE
☐ 1 Jamais
☐ 2 Moins d'une fois par mois
☐ 3 Une fois par mois
☐ 4 Une fois par semaine
☐ 5 Tous les jours ou presque
☐ 97. Non concerné (ne pas suggérer)
☐ 98. Ne sait pas (ne pas suggérer)
☐ 99. Refus de réponse (ne pas suggérer)

B.95 Au cours des douze derniers mois, combien de fois avez-vous eu besoin d'un premier verre pour pouvoir démarrer après avoir beaucoup bu la veille ?
UNE SEULE REPONSE POSSIBLE
☐ 1 Jamais
☐ 2 Moins d'une fois par mois
☐ 3 Une fois par mois
☐ 4 Une fois par semaine
☐ 5 Tous les jours ou presque
☐ 97. Non concerné (ne pas suggérer)
☐ 98. Ne sait pas (ne pas suggérer)
☐ 99. Refus de réponse (ne pas suggérer)

B.96 Au cours des douze derniers mois, combien de fois avez-vous eu un sentiment de culpabilité ou des remords après avoir bu ?
UNE SEULE REPONSE POSSIBLE
☐ 1 Jamais
☐ 2 Moins d'une fois par mois
☐ 3 Une fois par mois
☐ 4 Une fois par semaine
☐ 5 Tous les jours ou presque
☐ 97. Non concerné (ne pas suggérer)
☐ 98. Ne sait pas (ne pas suggérer)
☐ 99. Refus de réponse (ne pas suggérer)

B.97 Au cours des douze derniers mois, combien de fois avez-vous été incapable de vous rappeler ce qui s'était passé la soirée précédente parce que vous aviez bu ?
UNE SEULE REPONSE POSSIBLE
☐ 1 Jamais
☐ 2 Moins d'une fois par mois
☐ 3 Une fois par mois
☐ 4 Une fois par semaine
☐ 5 Tous les jours ou presque
☐ 97. Non concerné (ne pas suggérer)
☐ 98. Ne sait pas (ne pas suggérer)
☐ 99. Refus de réponse (ne pas suggérer)

B.98 Avez-vous déjà été blessé ou quelqu'un d'autre a-t-il été blessé parce que vous aviez bu ?
UNE SEULE REPONSE POSSIBLE
☐ 1 Non
☐ 2 Oui mais pas au cours des douze derniers mois
☐ 3 Oui, au cours de l'année
☐ 97. Non concerné (ne pas suggérer)
☐ 98. Ne sait pas (ne pas suggérer)
☐ 99. Refus de réponse (ne pas suggérer)

B.99 Un parent, un ami, un médecin ou un autre soignant s'est-il déjà inquiété de votre consommation d'alcool ou a-t-il suggéré que vous la réduisiez ?
UNE SEULE REPONSE POSSIBLE
☐ 1 Non
☐ 2 Oui mais pas au cours des douze derniers mois
☐ 3 Oui, au cours de l'année
☐ 97. Non concerné (ne pas suggérer)
☐ 98. Ne sait pas (ne pas suggérer)
☐ 99. Refus de réponse (ne pas suggérer)

Nous allons maintenant parler des drogues

B.100 <u>Au cours de votre vie</u> avez-vous déjà consommé du cannabis (joint, haschich, marijuana) ?
- ☐ 1 Oui
- ☐ 2 Non ➔ **103**
- ☐ 97. Non concerné (ne pas suggérer) ➔ **103**
- ☐ 98. Ne sait pas (ne pas suggérer) ➔ **103**
- ☐ 99. Refus de réponse (ne pas suggérer) ➔ **103**

Si B100=1
B.102 <u>Depuis votre diagnostic VIH</u>, vous est-il arrivé de consommer du cannabis pour soulager certains symptômes (douleurs, nausées) ?
UNE SEULE REPONSE POSSIBLE

- ☐ 1 Jamais
- ☐ 2 Parfois
- ☐ 3 Souvent
- ☐ 4 Toujours
- ☐ 97. Non concerné (ne pas suggérer)
- ☐ 98. Ne sait pas (ne pas suggérer)
- ☐ 99. Refus de réponse (ne pas suggérer)

Si B100=1
B.102b Au cours **<u>des 4 dernières semaines,</u> avez-vous consommé du cannabis ?**
UNE SEULE REPONSE POSSIBLE
- ☐ 1 Pas du tout
- ☐ 2 Moins d'une fois par semaine
- ☐ 3 1 à 6 fois par semaine
- ☐ 4 1 fois par jour
- ☐ 5 Plus d'une fois par jour
- ☐ 97. Non concerné (ne pas suggérer)
- ☐ 98. Ne sait pas (ne pas suggérer)
- ☐ 99. Refus de réponse (ne pas suggérer)

B.103 En dehors du cannabis, <u>au cours de votre vie,</u> avez-vous déjà consommé d'autres drogues illicites ou des médicaments détournés ?
- ☐ 1 Oui ➔ **B104**
- ☐ 2 Non ➔ **Module C = C1**
- ☐ 97. Non concerné (ne pas suggérer) ➔ **Module C = C1**
- ☐ 98. Ne sait pas (ne pas suggérer) ➔ **B110**
- ☐ 99. Refus de réponse (ne pas suggérer) ➔ **B110**

Si B103= 1
B.104 Depuis votre diagnostic VIH, vous est-il arrivé de consommer des stimulants (cocaïne, amphétamines) pour soulager certains symptômes (fatigue, douleurs, etc.) ?
UNE SEULE REPONSE POSSIBLE
- ☐ 1 Jamais
- ☐ 2 Parfois
- ☐ 3 Souvent
- ☐ 4 Toujours
- ☐ 97. Non concerné (ne pas suggérer)
- ☐ 98. Ne sait pas (ne pas suggérer)
- ☐ 99. Refus de réponse (ne pas suggérer)

Si B103= 1
B.104.b Au cours des 4 dernières semaines, en dehors du cannabis, avez-vous consommé d'autres drogues illicites ou des médicaments détournés ?
- ☐ 1 Oui
- ☐ 2 Non
- ☐ 97. Non concerné (ne pas suggérer)
- ☐ 98. Ne sait pas (ne pas suggérer)
- ☐ 99. Refus de réponse (ne pas suggérer)

B104c doivent être posées si B104b=1
B104cQuels produits avez-vous utilisé dans les 4 dernières semaines ?

B. 104c_1 héroïne
- ☐ 1 Pas du tout
- ☐ 2 Moins d'1fois par semaine
- ☐ 3 1 à 6 fois/semaine
- ☐ 4 1 fois/jour
- ☐ 5 Plusieurs fois par jour
- ☐ 97. Non concerné (ne pas suggérer)
- ☐ 98. Ne sait pas (ne pas suggérer)
- ☐ 99. Refus de réponse (ne pas suggérer)

B.104c.2 Méthadone, skénan, moscontin, néocodion
- ☐ 1 Pas du tout
- ☐ 2 Moins d'1fois par semaine
- ☐ 3 1 à 6 fois/semaine
- ☐ 4 1 fois/jour
- ☐ 5 Plusieurs fois par jour
- ☐ 97. Non concerné (ne pas suggérer)
- ☐ 98. Ne sait pas (ne pas suggérer)
- ☐ 99. Refus de réponse (ne pas suggérer)

B.104c.3 Subutex ou autre produit à base de buprénorphine
- ☐ 1 Pas du tout
- ☐ 2 Moins d'1fois par semaine
- ☐ 3 1 à 6 fois/semaine
- ☐ 4 1 fois/jour
- ☐ 5 Plusieurs fois par jour

☐ 97. Non concerné (ne pas suggérer)
☐ 98. Ne sait pas (ne pas suggérer)
☐ 99. Refus de réponse (ne pas suggérer)

B.104c.4 Benzodiazépines NON PRESCRITES (comme par exemple du rivotril, du rohypnol, du seresta, du valium….)
☐ 1 Pas du tout
☐ 2 Moins d'1fois par semaine
☐ 3 1 à 6 fois/semaine
☐ 4 1 fois/jour
☐ 5 Plusieurs fois par jour
☐ 97. Non concerné (ne pas suggérer)
☐ 98. Ne sait pas (ne pas suggérer)
☐ 99. Refus de réponse (ne pas suggérer)

B.104c.5 Cocaïne
☐ 1 Pas du tout
☐ 2 Moins d'1fois par semaine
☐ 3 1 à 6 fois/semaine
☐ 4 1 fois/jour
☐ 5 Plusieurs fois par jour
☐ 97. Non concerné (ne pas suggérer)
☐ 98. Ne sait pas (ne pas suggérer)
☐ 99. Refus de réponse (ne pas suggérer)

B.104c.6 Crack
☐ 1 Pas du tout
☐ 2 Moins d'1fois par semaine
☐ 3 1 à 6 fois/semaine
☐ 4 1 fois/jour
☐ 5 Plusieurs fois par jour
☐ 97. Non concerné (ne pas suggérer)
☐ 98. Ne sait pas (ne pas suggérer)
☐ 99. Refus de réponse (ne pas suggérer)

B.104c.7 Amphétamines
☐ 1 Pas du tout
☐ 2 Moins d'1fois par semaine
☐ 3 1 à 6 fois/semaine
☐ 4 1 fois/jour
☐ 5 Plusieurs fois par jour
☐ 97. Non concerné (ne pas suggérer)
☐ 98. Ne sait pas (ne pas suggérer)
☐ 99. Refus de réponse (ne pas suggérer)

B.104c.8 Autres : (précisez)
☐ 1 Pas du tout
☐ 2 Moins d'1fois par semaine
☐ 3 1 à 6 fois/semaine
☐ 4 1 fois/jour
☐ 5 Plusieurs fois par jour
☐ 97. Non concerné (ne pas suggérer)
☐ 98. Ne sait pas (ne pas suggérer)
☐ 99. Refus de réponse (ne pas suggérer)

B.105 Vous êtes-vous injecté des produits ne serait-ce qu'une seule fois dans votre vie, même il y a longtemps ?
☐ 1 Oui ➔ **B106**
☐ 2 Non ➔ **B110**
☐ 97. Non concerné (ne pas suggérer) ➔ **B110**
☐ 98. Ne sait pas (ne pas suggérer) ➔ **B110**
☐ 99. Refus de réponse (ne pas suggérer) ➔ **B110**

Si B105=1
B.106 Quel âge aviez-vous cette toute première fois ?

/___/___/ 1 **ans**
☐ 97. Non concerné (ne pas suggérer)
☐ 98. Ne sait pas (ne pas suggérer)
☐ 99. Refus de réponse (ne pas suggérer)

Si B105=1
B.107 et au cours des quatre dernières semaines, à quelle fréquence vous êtes-vous injecté des drogues ?
UNE SEULE REPONSE POSSIBLE

☐ 1 Jamais
☐ 2 Moins d'une fois par semaine
☐ 3 Plus d'une fois par semaine (mais moins d'une fois par jour)
☐ 4 Une fois par jour
☐ 5 Plus d'une fois par jour

☐ 97. Non concerné (ne pas suggérer)
☐ 98. Ne sait pas (ne pas suggérer)
☐ 99. Refus de réponse (ne pas suggérer)

Si B107 = 2 à 5 ou 97 ou 98 ou 99
B.108 Au cours des quatre dernières semaines, est-il arrivé que quelqu'un réutilise le matériel d'injection que vous aviez déjà utilisé ? (cuillère, fond alu, seringue, coton, citron vinaigre, eau, filtre etc.)
UNE SEULE REPONSE POSSIBLE

- ☐ 1 Jamais
- ☐ 2 Une fois
- ☐ 3 Plus d'une fois
- ☐ 97. Non concerné (ne pas suggérer)
- ☐ 98. Ne sait pas (ne pas suggérer)
- ☐ 99. Refus de réponse (ne pas suggérer)

Si B107 = 2 à 5 ou 97 ou 98 ou 99
B.109 Au cours des quatre dernières semaines, est-il arrivé que quelqu'un réutilise une seringue que vous aviez déjà utilisée ?
UNE SEULE REPONSE POSSIBLE

- ☐ 1 Jamais
- ☐ 2 Une fois
- ☐ 3 Plus d'une fois
- ☐ 97. Non concerné (ne pas suggérer)
- ☐ 98. Ne sait pas (ne pas suggérer)
- ☐ 99. Refus de réponse (ne pas suggérer)

B.110 Au cours de votre vie, un médecin vous a-t-il déjà prescrit un traitement de substitution (méthadone, subutex ou skenan) ?
SI LA REPONSE EST OUI, PLUSIEURS REPONSES POSSIBLES
- ☐ 1 Non ➔ **items exclusif**
- ☐ 2 Oui, du **Subutex**
- ☐ 3 Oui, de la **Méthadone**
- ☐ 4 Oui, autres (précisez le traitement)
- ☐ 97. Non concerné (ne pas suggérer) ➔ **items exclusif**
- ☐ 98. Ne sait pas (ne pas suggérer) ➔ **items exclusif**
- ☐ 99. Refus de réponse (ne pas suggérer) ➔ **items exclusif**

Poser B110.2 si B110 = 2 ou 3
B110.2 Et la première fois que vous avez pris du « … », c'était en quelle année
/___/___/ 1 **ans**
- ☐ 97. Non concerné (ne pas suggérer)
- ☐ 98. Ne sait pas (ne pas suggérer)
- ☐ 99. Refus de réponse (ne pas suggérer)
- ☐ 1 Subutex
- ☐ 2 Méthadone

Si B110 > 1 ➔ B111
B.111 Au cours des quatre dernières semaines, avez-vous pris un traitement de substitution prescrit ?
UNE SEULE REPONSE POSSIBLE

- ☐ 1 Non
- ☐ 2 Oui, Subutex
- ☐ 3 Oui, Méthadone
- ☐ 4 Oui, autre (à préciser)
- ☐ 97. Non concerné (ne pas suggérer)
- ☐ 98. Ne sait pas (ne pas suggérer)
- ☐ 99. Refus de réponse (ne pas suggérer)

MODULE C : INSERTION PROFESSIONNELLE

Nous allons maintenant nous intéresser à votre situation par rapport à l'emploi.

A tous

C.1 Actuellement, qu'elle est votre situation ?

PLUSIEURS REPONSES POSSIBLES

- ☐ 1. Vous avez un emploi déclaré/activité ➔C3
- ☐ 2 Vous avez une activité non déclarée ou vous faites des petits boulots
- ☐ Vous êtes en formation rémunérée
- ☐ 3. Vous êtes au chômage
- ☐ 4. Vous êtes retraité / préretraité
- ☐ 5. Vous êtes en invalidité
- ☐ 6. Vous n'avez aucune activité professionnelle
- ☐ 7. Autre situation : préciser………→ *prévoir INSER*
- ☐ 97. Non concerné (ne pas suggérer)
- ☐ 99. Refus de réponse (ne pas suggérer)

A tous

C.2 Actuellement, faites vous des études ?

- ☐ 1 Oui
- ☐ 2 Non
- ☐ 97. Non concerné (ne pas suggérer)
- ☐ 98. Ne sait pas (ne pas suggérer)
- ☐ 99. Refus de réponse (ne pas suggérer)

Si C1=1 (la personne a un emploi déclaré actuellement) ➔C3 et la suite

Si C1≠1 ET C1=2 à 20 (la personne n'a pas d'emploi déclaré actuellement) ➔ C35

C.3 Depuis quand occupez-vous votre emploi actuel?

/___/___/___/___/ Année

/___/___/ mois

C.4 Avez-vous accédé à cet emploi…

UNE SEULE REPONSE POSSIBLE

- ☐ 1. avant que vous ayez appris votre infection à VIH
- ☐ 2. après que vous ayez appris votre infection à VIH
- ☐ 97. Non concerné (ne pas suggérer)
- ☐ 98. Ne sait pas (ne pas suggérer)
- ☐ 99. Refus de réponse (ne pas suggérer)

C.5 Quelle profession exercez-vous ?

C.6 Exercez-vous cette profession comme… -

UNE SEULE REPONSE POSSIBLE

- ☐ 1. Salarié ➔ **C6.1 puis C7**
- ☐ 2. A votre compte, en libéral, travailleur indépendant, free-lance ➔ **C8**
- ☐ 3. Intermittent du spectacle
- ☐ 4. Autre situation, Précisez :……→ *prévoir INSER*
- ☐ 97. Non concerné (ne pas suggérer)
- ☐ 98. Ne sait pas (ne pas suggérer)
- ☐ 99. Refus de réponse (ne pas suggérer)

Si C6=1➔ C6.1

C.6.1 Plus précisément, vous êtes ?

UNE SEULE REPONSE POSSIBLE

- ☐ 1. Salarié du public (Etat, collectivité territoriale, AP, EDF, SNCF etc.)
- ☐ 2. Salarié du secteur privé
- ☐ 3. Salarié d'un particulier
- ☐ 4. Autre
- ☐ 97. Non concerné (ne pas suggérer)
- ☐ 98. Ne sait pas (ne pas suggérer)
- ☐ 99. Refus de réponse (ne pas suggérer)

Si C6=1➔C7

C.7 Quelle est la nature de votre contrat de travail ?

UNE SEULE REPONSE POSSIBLE

- ☐ 1. Contrat à Durée Indéterminée (CDI) OU titulaire de la fonction publique
- ☐ 2. Contrat à Durée Déterminée (CDD)
- ☐ 3. Contrat aidé (Contrat Unique d'Insertion/CUI -Contrat Initiative Emploi/ CIE -Contrat d'Accompagnement dans l'Emploi/CAE, etc.)
- ☐ 4. Intérim ou vacations
- ☐ 5. Apprentissage, stage
- ☐ 7. Travail saisonnier
- ☐ 8. Autre. Précisez : …………→ *prévoir INSER*
- ☐ 97. Non concerné (ne pas suggérer)
- ☐ 98. Ne sait pas (ne pas suggérer)
- ☐ 99. Refus de réponse (ne pas suggérer)

Si C1=1 (POUR TOUTES LES PERSONNES AYANT UN EMPLOI DECLARE ACTUELLEMENT) et C6 =2 poser C8

C.8 Aujourd'hui, êtes-vous en arrêt-maladie ?

- ☐ 1. Oui ➔ **C8.1**
- ☐ 2. Non ➔ **C9**
- ☐ 97. Non concerné (ne pas suggérer) ➔ **C9**
- ☐ 98. Ne sait pas (ne pas suggérer) ➔ **C9**
- ☐ 99. Refus de réponse (ne pas suggérer) ➔ **C9**

Si C8=1➔ C8.1

C8.1 Depuis quelle date (*Date de votre dernier mois de travail)* **?**

/___/___/___/___/ Année /___/___/ mois /___/___/Jour

Si C8=2➔ C9

C.9 Et au cours des 12 derniers mois, avez-vous été en arrêt maladie ?

- ☐ 1. Oui ➔ **C9.1**
- ☐ 2. Non ➔ **C10**
- ☐ 97. Non concerné (ne pas suggérer)
- ☐ 98. Ne sait pas (ne pas suggérer)
- ☐ 99. Refus de réponse (ne pas suggérer)

Si C9=1➔ C9.1

C.9.1 Combien de temps au total ?

- ☐ 1. Une semaine ou moins
- ☐ 2. Entre 1 semaine et 1 mois (>1 semaine et ≤1 mois)
- ☐ 3. Entre 1 et 3 mois (>1 mois et ≤3 mois)
- ☐ 4. Plus de 3 mois (>3 mois)

Si C1=1 (POUR TOUTES LES PERSONNES AYANT UN EMPLOI DECLARE ACTUELLEMENT)

C.10 Avez-vous vous-même parlé de votre infection à VIH sur votre lieu de travail ou avec des relations de travail ?

- ☐ 1. Oui ➔ **C11**
- ☐ 2. Non ➔**C12**
- ☐ 97. Non concerné (ne pas suggérer) ➔ **C12**
- ☐ 98. Ne sait pas (ne pas suggérer)
- ☐ 99. Refus de réponse (ne pas suggérer)

Si C10=1➔ C11

C.11 A quelle(s) personne(s) ?

PLUSIEURS REPONSES POSSIBLES

C.12

- ☐ 1. Oui
- ☐ 2. Non
- ☐ 97. Non concerné (ne pas suggérer)
- ☐ 98. Ne sait pas (ne pas suggérer)
- ☐ 99. Refus de réponse (ne pas suggérer)

**C11.1 A un ou des collègues**	☐ 1	☐ 2
**C11.2 Au médecin du travail**	☐ 1	☐ 2
**C11.3 A votre employeur ou votre supérieur hiérarchique**	☐ 1	☐ 2
**C11.4 A d'autres personnes (à préciser)**	☐ 1	☐ 2

Il est possible d'être traité de façon injuste ou déplacée au travail. Nous allons parler de ce qui a pu arriver au cours des deux dernières années.

Vous personnellement, AU COURS DES DEUX DERNIERES ANNEES, dans votre travail, est-il arrivé :

C12.1qu'on vous refuse injustement une promotion ?

UNE SEULE REPONSE POSSIBLE

- ☐ 1. Oui
- ☐ 2. Non
- ☐ 97. Non concerné (ne pas suggérer)
- ☐ 98. Ne sait pas (ne pas suggérer)
- ☐ 99. Refus de réponse (ne pas suggérer)

C12.2 qu'on vous impose systématiquement des horaires dont personne ne voulait ?

- ☐ 1. Oui
- ☐ 2. Non
- ☐ 97. Non concerné (ne pas suggérer)
- ☐ 98. Ne sait pas (ne pas suggérer)
- ☐ 99. Refus de réponse (ne pas suggérer)

C12.3 qu'on vous charge systématiquement de tâches inutiles ou dégradantes, ou dont personne ne voulait ?

- ☐ 1. Oui
- ☐ 2. Non
- ☐ 97. Non concerné (ne pas suggérer)
- ☐ 98. Ne sait pas (ne pas suggérer)
- ☐ 99. Refus de réponse (ne pas suggérer)

Si C12=1 ou C13=1 ou C14=1 ➔ C15

C.15 Pensez vous que c'était à cause de…

PLUSIEURS REPONSES POSSIBLES

- ☐1 Vos origines ou votre nationalité
- ☐2 Votre couleur de peau
- ☐3 Au fait d'être enceinte, d'avoir des enfants en bas âge **(filtrer pour les femmes)**
- ☐4 Votre orientation sexuelle
- ☐5 Votre séropositivité
- ☐6 Votre usage d'alcool
- ☐7 Votre usage de drogue actuel ou passé **(seulement pour les Usagers de Drogues : B100=1 ou B103=1 ou B105=1)**
- ☐8Votre façon de vous habiller

- ☐9 Le lieu ou vous vivez, la réputation de votre quartier
- ☐10 Parce que vous êtes à la CMU ou à l'AME
- ☐11 autres **(à préciser)**
- ☐ 97. Non concerné (ne pas suggérer)
- ☐98. Ne sait pas (ne pas suggérer)
- ☐ 99. Refus de réponse (ne pas suggérer)

C.16 Depuis que vous avez appris votre infection à VIH, avez-vous bénéficié… :
- ☐ 1. Oui
- ☐ 2. Non
- ☐ 97. Non concerné (ne pas suggérer)
- ☐ 98. Ne sait pas (ne pas suggérer)
- ☐ 99. Refus de réponse (ne pas suggérer)

C16.1. d'aménagements du temps de travail tels que mi-temps thérapeutique, temps partiel, etc…
C16.2. d'aménagements des horaires de travail
C16.3. d'aménagements du poste de travail tels que reclassements, réattributions des tâches

Si C16.1=1 ou C16.2=1 ou C16.3=1 ➔ C17
C.17 Désiriez-vous ces aménagements ?
- ☐ 1. Oui
- ☐ 2. Non
- ☐ 97. Non concerné (ne pas suggérer)
- ☐98. Ne sait pas (ne pas suggérer)
- ☐ 99. Refus de réponse (ne pas suggérer)

Nous allons parler maintenant de vos conditions de travail

C.18 Quelle est votre durée de transport domicile-travail, aller-retour ?
UNE SEULE REPONSE POSSIBLE
- ☐1 moins de 1 heure
- ☐2 1 à 2 heures
- ☐3 Plus de 2 heures
- ☐4 Déplacements trop fréquents pour donner une réponse (ex VRP)
- ☐ 97. Non concerné (ne pas suggérer)
- ☐ 98. Ne sait pas (ne pas suggérer)
- ☐99. Refus de réponse (ne pas suggérer)

C.19 Effectuez-vous un travail de nuit, c'est-à-dire entre minuit et 5 heures ?
UNE SEULE REPONSE POSSIBLE
- ☐1 Oui, au moins 50 nuits par an
- ☐2 Oui, moins de 50 nuits par an
- ☐3 Non
- ☐ 97. Non concerné (ne pas suggérer)
- ☐ 98. Ne sait pas (ne pas suggérer)
- ☐ 99. Refus de réponse (ne pas suggérer)

C.20 Disposez-vous d'au moins 48 heures consécutives (deux jours de suite) de repos au cours d'une semaine ?
UNE SEULE REPONSE POSSIBLE
- ☐1 Oui
- ☐3 Pas toujours
- ☐4 Non, jamais
- ☐ 97. Non concerné (ne pas suggérer)
- ☐ 98. Ne sait pas (ne pas suggérer)
- ☐ 99. Refus de réponse (ne pas suggérer)

C.22 Diriez-vous qu'il vous est souvent difficile de respecter les objectifs ou les délais imposés dans votre travail ?
UNE SEULE REPONSE POSSIBLE
- ☐1 Oui
- ☐2 Non
- ☐3 Travaille seul (ne pas suggérer)
- ☐ 97. Non concerné *(ne pas suggérer)*
- ☐ 98. Ne sait pas (ne pas suggérer)
- ☐ 99. Refus de réponse (ne pas suggérer)

C.23 Diriez-vous que votre travail vous permet souvent de prendre des décisions par vous-même ?
UNE SEULE REPONSE POSSIBLE
- ☐1 Oui
- ☐2 Non
- ☐3 Travaille seul (ne pas suggérer)
- ☐ 97. Non concerné *(ne pas suggérer)*
- ☐ 98. Ne sait pas (ne pas suggérer)
- ☐ 99. Refus de réponse (ne pas suggérer)

C.25 Diriez-vous que les collègues avec qui vous travaillez vous manifestent de l'intérêt ?
UNE SEULE REPONSE POSSIBLE
- ☐1 Oui
- ☐2 Non
- ☐3 Travaille seul (ne pas suggérer)
- ☐ 97. Non concerné *(ne pas suggérer)*
- ☐ 98. Ne sait pas (ne pas suggérer)
- ☐ 99. Refus de réponse (ne pas suggérer)

C.27 Diriez-vous que dans votre travail, vous disposez des moyens de faire un travail de qualité ?
UNE SEULE REPONSE POSSIBLE
- ☐1 Oui
- ☐2 Non
- ☐3 Travaille seul (ne pas suggérer)
- ☐ 97. Non concerné *(ne pas suggérer)*
- ☐ 98. Ne sait pas (ne pas suggérer)
- ☐ 99. Refus de réponse (ne pas suggérer)

C.28 Diriez-vous de votre travail qu'il est fatiguant nerveusement ?
UNE SEULE REPONSE POSSIBLE
☐1 Oui
☐2 Non
☐ 97. Non concerné (ne pas suggérer)
☐ 98. Ne sait pas (ne pas suggérer)
☐ 99. Refus de réponse (ne pas suggérer)

C.29 Diriez-vous de votre travail qu'il est fatiguant physiquement ?
UNE SEULE REPONSE POSSIBLE
☐1 Oui
☐2 Non
☐ 97. Non concerné (ne pas suggérer)
☐ 98. Ne sait pas (ne pas suggérer)
☐ 99. Refus de réponse (ne pas suggérer)

C.33 Pensez-vous que, dans les deux prochaines années, il y a un risque pour que vous perdiez votre emploi ?
UNE SEULE REPONSE POSSIBLE
☐1 Oui sans doute
☐2 Oui peut-être
☐3 Probablement non
☐ 97. Non concerné (ne pas suggérer)
☐ 98. Ne sait pas (ne pas suggérer)
☐ 99. Refus de réponse (ne pas suggérer)

C.34 Et si vous perdiez votre emploi, pensez-vous que vous retrouveriez un emploi similaire avec le même niveau de salaire ?
UNE SEULE REPONSE POSSIBLE
☐1 Oui sans doute
☐2 Oui peut-être
☐3 Probablement non
☐ 97. Non concerné (ne pas suggérer)
☐ 98. Ne sait pas (ne pas suggérer)
☐ 99. Refus de réponse (ne pas suggérer)

Si C1≠ 1 (personnes qui n'ont pas d'emploi déclaré actuellement) ET C1=2 à 20➔ C35 et la suite
Nous allons parler de votre dernière activité professionnelle (déclarée)

C.35 Quand avez-vous cessé votre dernière activité déclarée ?

/___/___/___/___/ Année

/___/___/ mois

☐ 2. N'a jamais eu d'activité déclarée ➔ C46

C.36 Etait-ce avant ou après le diagnostic de votre infection à VIH ?
UNE SEULE REPONSE POSSIBLE
☐ 1 Avant
☐ 2 Après
☐ 97. Non concerné (ne pas suggérer)
☐ 98. Ne sait pas (ne pas suggérer)
☐ 99. Refus de réponse (ne pas suggérer)

C.37 Quelle était votre profession ?
Précisez : ………………………………………………………

C.38 Exerciez-vous cette profession comme…
UNE SEULE REPONSE POSSIBLE
☐ 1. Salarié ➔ C38.1 puis C39
☐ 2. A votre compte, en libéral, travailleur indépendant, free-lance... ➔ C40
☐ 3. Intermittent du spectacle
☐ 4. Autre situation (**Préciser)**
☐ 97. Non concerné (ne pas suggérer)
☐ 98. Ne sait pas (ne pas suggérer)
☐ 99. Refus de réponse (ne pas suggérer)

Si C38=1➔ C38.1
C.38.1 Plus précisément, vous étiez ?
UNE SEULE REPONSE POSSIBLE
☐ 1. Salarié du public (Etat, collectivité territoriale, AP, EDF, SNCF etc.)
☐ 2. Salarié du secteur privé
☐ 3. Salarié d'un particulier
☐ 4. Autre
☐ 97. Non concerné (ne pas suggérer)
☐ 98. Ne sait pas (ne pas suggérer)
☐ 99. Refus de réponse (ne pas suggérer)

Si C38=1➔C39
C.39 Quelle était la nature de votre contrat de travail ?
UNE SEULE REPONSE POSSIBLE
☐ 1. Contrat à Durée Indéterminée (CDI) OU titulaire de la fonction publique
☐ 2. Contrat à Durée Déterminée (CDD)
☐ 3. Contrat aidé (Contrat Unique d'Insertion/CUI -, Contrat Initiative Emploi/ CIE -Contrat d'Accompagnement dans l'Emploi/ CAE, etc.)
☐ 4. Intérim ou vacations
☐ 5. Apprentissage, stage
☐ 7. Travail saisonnier
☐ 8. Autre **(Préciser)**
☐ 97. Non concerné (ne pas suggérer)

☐ 98. Ne sait pas (ne pas suggérer)
☐ 99. Refus de réponse (ne pas suggérer)

Si C1≠ 1 (personnes qui n'ont pas d'emploi déclaré actuellement) ET C1=2 à 20

C.40 Pour quelle raison cet emploi s'est-il terminé ?

UNE SEULE REPONSE POSSIBLE

☐ 1. Vous avez démissionné pour raison de santé
☐ 2. Vous avez démissionné pour une autre raison
☐ 3. C'était la fin de votre contrat
☐ 4. Vous avez été licencié(e) ➔ **C41**
☐ 5. Vous avez pris votre retraite / préretraité➔ **C42**
☐ 6. L'entreprise que vous dirigiez a fait faillite
☐ 97. Non concerné (ne pas suggérer)
☐ 98. Ne sait pas (ne pas suggérer)
☐ 99. Refus de réponse (ne pas suggérer)

Si C40 ≠ 4 ou ≠ 5 ➔ C43

Si C40 = 4 ➔ C41

C.41 Pour quelle raison avez-vous été licencié(e) ?

UNE SEULE REPONSE POSSIBLE

☐ 1. Pour raison économique
☐ 2. Pour d'autres raisons (mésentente, faute, etc.)
☐ 97. Non concerné (ne pas suggérer)
☐ 98. Ne sait pas (ne pas suggérer)
☐ 99. Refus de réponse (ne pas suggérer)

Si C40 = 5 ➔ C42

C.42 S'agissait-il d'un départ à la retraite / pré-retraite anticipé ?

UNE SEULE REPONSE POSSIBLE

☐1 Oui
☐2 Non
☐ 97. Non concerné (ne pas suggérer)
☐ 98. Ne sait pas (ne pas suggérer)
☐ 99. Refus de réponse (ne pas suggérer)

Poser C.43 => Si C1≠ 1 (personnes qui n'ont pas d'emploi déclaré actuellement) ET C1=2 à 20 et C35 ≠ code 2

C.43 Diriez-vous que votre état de santé a joué un rôle dans la rupture de votre contrat de travail ou dans l'arrêt de votre activité ?

UNE SEULE REPONSE POSSIBLE

☐ 1. Beaucoup ➔ **C44**
☐ 2. Assez ➔ **C44**
☐ 3. Un peu ➔ **C44**
☐ 4. Pas du tout
☐ 97. Non concerné (ne pas suggérer)
☐ 98. Ne sait pas (ne pas suggérer)
☐ 99. Refus de réponse (ne pas suggérer)

Si C43 = 1,2 ou 3 ➔ C44

C.44 Etait-ce en rapport avec …?

UNE SEULE REPONSE POSSIBLE

☐ 1 Votre infection VIH
☐ 2 Un autre problème de santé
☐ 97. Non concerné (ne pas suggérer)
☐ 98. Ne sait pas (ne pas suggérer)
☐ 99. Refus de réponse (ne pas suggérer)

C.45 Juste après avoir cessé cette activité, est ce que vous pouviez compter sur les revenus de votre conjoint ?

UNE SEULE REPONSE POSSIBLE

☐ 1. oui
☐ 2. non
☐ 97. Non concerné (ne pas suggérer)
☐ 98. Ne sait pas (ne pas suggérer)
☐ 99. Refus de réponse (ne pas suggérer)

C.46 Aujourd'hui envisagez-vous de travailler ou de retravailler (de façon déclarée) ?

UNE SEULE REPONSE POSSIBLE

☐ 1. Oui
☐ 2. Non
☐ 97. Non concerné (ne pas suggérer) (à la retraite ou préretraite) ➔**C50**
☐ 98. Ne sait pas (ne pas suggérer) ➔**C50**
☐ 99. Refus de réponse (ne pas suggérer) ➔**C50**

Poser C47 si C46=1 ou 2

C.47 Si vous repreniez un emploi, pensez-vous que vos ressources …

UNE SEULE REPONSE POSSIBLE

☐ 1 Diminueraient
☐ 2 Augmenteraient
☐ 3 Resteraient les mêmes
☐ 97. Non concerné (ne pas suggérer)
☐ 98. Ne sait pas (ne pas suggérer)
☐ 99. Refus de réponse (ne pas suggérer)

Si C46=1 ➔ C 48 (pour les personnes qui envisagent de (re)travailler)

C.48 Depuis 3 mois, avez-vous fait des démarches pour trouver un emploi ?

UNE SEULE REPONSE POSSIBLE

☐ 1. Oui
☐ 2. Non ➔ **C50**
☐ 97. Non concerné (ne pas suggérer) ➔ **C50**
☐ 98. Ne sait pas (ne pas suggérer) ➔ **C50**
☐ 99. Refus de réponse (ne pas suggérer) ➔ **C50**

A TOUS

Poser C50 Si C1≠ 1 et si C48=2 ou NSP ou Non Concerné ou Refus de répondre

C.50 Au cours des deux dernières années, avez-vous fait des démarches pour chercher un emploi ou changer d'emploi ?

☐1 Oui ➔ **C51**
☐2 Non➔**Module D = D1**

Durant ces deux dernières années, au cours de vos recherches d'emploi, avez-vous trouvé :
UNE SEULE REPONSE POSSIBLE

C51.1 qu'on ne vous faisait pas confiance ?

☐ 1. Oui
☐ 2. Non
☐ 97. Non concerné (ne pas suggérer)
☐ 98. Ne sait pas (ne pas suggérer)
☐ 99. Refus de réponse (ne pas suggérer)

C51.2 qu'on vous posait des questions déplacées ?

☐ 1. Oui
☐ 2. Non
☐ 97. Non concerné (ne pas suggérer)
☐ 98. Ne sait pas (ne pas suggérer)
☐ 99. Refus de réponse (ne pas suggérer)

C51.3 qu'on vous refuse injustement un emploi?

☐ 1. Oui
☐ 2. Non
☐ 97. Non concerné (ne pas suggérer)
☐ 98. Ne sait pas (ne pas suggérer)
☐ 99. Refus de réponse (ne pas suggérer)

Si C51=1 ou C52=1 ou C53 = 1 ➔ C54

C.54 Pensez vous que c'était à cause de… …

PLUSIEURS REPONSES POSSIBLES

☐1 Vos origines ou votre nationalité
☐2 Votre couleur de peau
☐3 Votre sexe (le fait d'être un homme ou une femme)
☐4 Votre orientation sexuelle
☐5 Votre séropositivité
☐6 Votre usage d'alcool
☐7 Votre usage de drogue actuel ou passé
☐8 Votre façon de vous habiller
☐9 Le lieu ou vous vivez, la réputation de votre quartier
☐10 Parce que vous êtes à la CMU ou à l'AME
☐11 Autres **(à préciser)**
☐ 97. Non concerné (ne pas suggérer)
☐ 98. Ne sait pas (ne pas suggérer)
☐ 99. Refus de réponse (ne pas suggérer)

MODULE D : RESSOURCES ET CONDITIONS DE VIE

Nous allons maintenant parler de vos ressources financières

D.1 Avez-vous un revenu personnel, c'est-à-dire de l'argent que vous obtenez de votre travail ou d'allocations (RSA, allocation chômage, API, etc.) et que pouvez utiliser comme vous l'entendez ?
UNE SEULE REPONSE POSSIBLE
☐ 1. Oui
☐ 2. Non
☐ 98. *Ne sait pas*
☐ 99. *Refus de réponse*

D.2 Est-ce que vous touchez personnellement…
UNE SEULE REPONSE POSSIBLE
☐ 1. Le RSA (revenu de solidarité active)/ l'API (allocation parent isolé)
☐ 2. L'AAH (allocation aux adultes handicapés)
☐ 3 Une pension d'invalidité de la sécurité sociale, d'une mutuelle, d'une assurance privée
☐ 4 Aucune de ces prestations
☐ 97. *Non concerné*
☐ 98. *Ne sait pas*
☐ 99. *Refus de réponse*

D.3 Si l'on additionne l'ensemble des revenus de toutes les personnes qui vivent actuellement dans le même logement que vous (*salaires ou revenus y compris du conjoint, allocations*), au total, cela représente quel montant par mois?

/___/___/___/___/___/___/ euros

Si D3= Si NON CONCERNE(99997)/ Si NSP(99998) / SI REFUS (99999)
D.4 Si vous ne pouvez pas donner un montant précis, pouvez-vous en donner une estimation ?
UNE SEULE REPONSE POSSIBLE

☐ 1. Moins de 500 €
☐ 2. de 500 à 700 €
☐ 3. De701 € à 1000 €
☐ 4. de 1001 à 1500 €
☐ 5. de 1501 à 2000 €
☐ 6. de 2001 à 3000 €
☐ 7. de 3001 à 5000 €
☐ 8. de 5001 à 7500 €
☐ 9. plus de 7500 €
☐ 97. *Non concerné*
☐ 98. *Ne sait pas*
☐ 99. *Refus de réponse*

D.5 Actuellement, diriez-vous plutôt que financièrement…
UNE SEULE REPONSE POSSIBLE
☐ 1. Vous ne pouvez pas y arriver sans faire de dettes
☐ 2. Vous y arrivez difficilement
☐ 3. C'est juste, il faut faire attention
☐ 4. Ca va
☐ 5. Vous êtes plutôt à l'aise
☐ 6. Vous êtes vraiment à l'aise
☐ 97. Non concerné
☐ 98. Ne sait pas
☐ 99. *Refus de réponse*

D.6 Les moyens financiers dont vous disposez vous permettent-ils (ou vous permettraient-ils si vous en éprouviez le besoin)…

D.6_1 De manger de la viande, ou du poisson au moins une fois tous les 2 jours ?
UNE SEULE REPONSE POSSIBLE
☐ 1 Oui
☐ 2 Non
☐ 97. Non concerné
☐ 98. Ne sait pas
☐ 99. Refus de réponse

D.6_2 D'acheter des vêtements neufs ?
UNE SEULE REPONSE POSSIBLE
☐ 1 Oui
☐ 2 Non
☐ 97. Non concerné
☐ 98. Ne sait pas
☐ 99. Refus de réponse

D.6_3 De posséder deux paires de bonnes chaussures (pour chaque adulte du ménage) ?
UNE SEULE REPONSE POSSIBLE
☐ 1 Oui
☐ 2 Non
☐ 97. Non concerné
☐ 98. Ne sait pas
☐ 99. Refus de réponse

D.6_4 De remplacer des meubles hors d'usage ?
UNE SEULE REPONSE POSSIBLE
☐ 1 Oui
☐ 2 Non
☐ 97. Non concerné
☐ 98. Ne sait pas
☐ 99. Refus de réponse

D.6_5 De maintenir votre logement à la bonne température ?
UNE SEULE REPONSE POSSIBLE
☐ 1 Oui
☐ 2 Non
☐ 97. Non concerné
☐ 98. Ne sait pas
☐ 99. Refus de réponse

D.6_6 De recevoir des parents ou des amis pour boire un verre ou pour un repas au moins une fois par mois ?
UNE SEULE REPONSE POSSIBLE
☐ 1 Oui
☐ 2 Non
☐ 97. Non concerné
☐ 98. Ne sait pas
☐ 99. Refus de réponse

D.6_7 D'offrir des cadeaux à la famille ou aux amis une fois par an au moins ?
UNE SEULE REPONSE POSSIBLE
☐ 1 Oui
☐ 2 Non
☐ 97. Non concerné
☐ 98. Ne sait pas
☐ 99. Refus de réponse

D.6_8 De vous payer une semaine de vacances en dehors de chez vous une fois par an ?
UNE SEULE REPONSE POSSIBLE
☐ 1 Oui
☐ 2 Non
☐ 97. Non concerné
☐ 98. Ne sait pas
☐ 99. Refus de réponse

D.12 Au cours des 4 dernières semaines, vous est-il arrivé à vous ou à quelqu'un avec qui vous habitez de passer une journée complète sans prendre au moins un repas complet, par manque d'argent ?
UNE SEULE REPONSE POSSIBLE
☐ 1 Oui
☐ 2 Non
☐ 97. Non concerné
☐ 98. Ne sait pas
☐ 99. Refus de réponse

D.13 Au cours des 4 dernières semaines, vous est-il arrivé d'avoir recours à des repas gratuits dans une structure d'aide alimentaire (Restaurants du Coeur, Secours populaire,…)
UNE SEULE REPONSE POSSIBLE
☐ 1 Oui
☐ 2 Non
☐ 97. Non concerné
☐ 98. Ne sait pas
☐ 99. Refus de réponse

D.14 Au cours des 4 dernières semaines, vous est-il arrivé d'avoir recours à une épicerie sociale ou solidaire, c'est à dire une épicerie où les aliments sont vendus de 10 à 30% du prix du marché ?
UNE SEULE REPONSE POSSIBLE
☐ 1 Oui
☐ 2 Non
☐ 97. Non concerné
☐ 98. Ne sait pas
☐ 99. Refus de réponse

Nous allons maintenant parler du logement que vous avez actuellement
D.15 Actuellement, par rapport à votre habitation, vous êtes (vous et/ou votre conjoint(e)/compagne) ?
UNE SEULE REPONSE POSSIBLE
☐ 1. Locataire
☐ 2. Locataire d'un appartement médicalisé comme un ACT, un appartement relais
☐ 3. Propriétaire de votre logement (ou accédant à la propriété)
☐ 4. Vous vivez chez un (ou des) ami(s)
☐ 5. Vous vivez chez vos parents ou chez d'autres membres de votre famille
☐ 6. Vous logez dans un foyer ou dans un centre d'hébergement
☐ 7. Vous êtes sans domicile fixe (hôtel, etc.)
☐ 97. Non concerné
☐ 98. Ne sait pas
☐ 99. Refus de réponse

D.16 Estimez-vous que vos conditions actuelles de logement sont …
UNE SEULE REPONSE POSSIBLE
☐ 1. Très satisfaisantes
☐ 2. Satisfaisantes
☐ 3. Acceptables
☐ 4. Insuffisantes
☐ 5. Très insuffisantes
☐ 97. Non concerné
☐ 98. Ne sait pas
☐ 99. Refus de réponse

Depuis le diagnostic de votre infection VIH/séropositivité, avez-vous été dans une des situations suivantes au moins une nuit

D.17 Vous avez été obligé(e) de loger dans la famille ou chez des amis parce que vos revenus ne vous permettaient pas d'avoir votre propre logement ?
- ☐ 1 Oui
- ☐ 2 Non
- ☐ 97. Non concerné
- ☐ 98. Ne sait pas
- ☐ 99. Refus de réponse

D.18 Vous avez utilisé un hébergement d'urgence (où on ne va que pour dormir) ?
- ☐ 1 Oui
- ☐ 2 Non
- ☐ 97. Non concerné
- ☐ 98. Ne sait pas
- ☐ 99. Refus de réponse

D.19 Vous avez habité dans un foyer, parce que vous n'aviez pas le choix ?
- ☐ 1 Oui
- ☐ 2 Non
- ☐ 97. Non concerné
- ☐ 98. Ne sait pas
- ☐ 99. Refus de réponse

D.20 Vous avez habité dans un hôtel, parce que vous n'aviez pas le choix ?
- ☐ 1 Oui
- ☐ 2 Non
- ☐ 97. Non concerné
- ☐ 98. Ne sait pas
- ☐ 99. Refus de réponse

D.21 Vous avez habité dans un squat, parce que vous n'aviez pas le choix ?
- ☐ 1 Oui
- ☐ 2 Non
- ☐ 97. Non concerné
- ☐ 98. Ne sait pas
- ☐ 99. Refus de réponse

D.22 Vous avez été obligé(e) de dormir au moins une nuit dans la rue, un parking ou un autre lieu public parce que vous n'aviez rien d'autre
- ☐ 1 Oui
- ☐ 2 Non
- ☐ 97. Non concerné
- ☐ 98. Ne sait pas
- ☐ 99. Refus de réponse

Si la personne est de nationalité étrangère (A8 ≠ « France métropolitaine ou France outre-mer » et A11=2 ou 3) ➔ D23 à D27

Si Autres cas ➔Module E

Si A8 ≠ « France métropolitaine ou France outre-mer » et A11=2 ou 3
PLUSIEURS REPONSES POSSIBLES
D.23 Avez-vous un(e) ou des conjoint(e) qui vit ?
- ☐ 1. Dans votre pays d'origine
- ☐ 2. Dans un autre pays hors de France
- ☐ 3. Non, vous n'avez pas de conjoint(e) hors de France
- ☐ 97. Non concerné
- ☐ 98. Ne sait pas
- ☐ 99. Refus de réponse

Si A8 ≠ « France métropolitaine ou France outre-mer » et A11=2 ou 3 et A3 >0 poser D.24
PLUSIEURS REPONSES POSSIBLES
D.24 Avez-vous un enfant ou des enfants qui vivent ?
- ☐ 1. Dans votre pays d'origine
- ☐ 2. Dans un autre, hors de France
- ☐ 3. Non
- ☐ 97. Non concerné
- ☐ 98. Ne sait pas
- ☐ 99. Refus de réponse

Si D.23 =1 ou 2
D.25 Avez-vous fait des démarches pour faire venir votre conjoint en France ?
- ☐ 1 Oui
- ☐ 2 Non
- ☐ 97. Non concerné
- ☐ 98. Ne sait pas
- ☐ 99. Refus de réponse

Si D.24 =1 ou 2
D.26 Avez-vous fait des démarches pour faire venir votre/vos enfant(s) en France ?
- ☐ 1 Oui
- ☐ 2 Non
- ☐ 97. Non concerné
- ☐ 98. Ne sait pas
- ☐ 99. Refus de réponse

MODULE E : LIENS SOCIAUX, DISCRIMINATION, STIGMATISATION

Nous allons maintenant parler des personnes avec qui vous êtes en relation

E.1 Combien avez-vous d'amis proches c'est-à-dire des personnes avec qui vous êtes bien et à qui vous pouvez parler de choses personnelles ?
/_____/ → *sur 2 positions*
Si E1=0 ne pas poser E.2 ni E2.1 et passer directement à E3

E2 est posée si E1 >0
E.2 Et parmi ces amis, combien en avez-vous vus ou avec combien avez-vous parlé au téléphone ou échangé des mails dans les deux dernières semaines ?
/_____/ → *sur 2 positions*

E2.1 est posée si E1 >0
E.2.1 Et parmi vos amis proches, combien connaissent votre séropositivité ?
/_____/ → *sur 2 positions*

E.3 Au cours des deux dernières semaines avez-vous vu, parlé au téléphone ou échangé des mails avec …..
PLUSIEURS REPONSES POSSIBLES

E.3.1. Votre père
☐1 Oui, vu
☐2 Oui, parlé au téléphone
☐3 Oui, échangé des mails
☐4 Non
☐ 97. Non concerné (ne pas suggérer)
☐ 98. Ne sait pas (ne pas suggérer)
☐ 99. Refus de réponse (ne pas suggérer)

E3.1.2 doit être posée si E 3.1 =1, 2,3, ou 4
E3.1.2 Votre père connaît-il votre séropositivité ?
☐1 Oui
☐2 Non, il ne le sait pas
☐3 Vous ne savez pas s'il le sait
☐ 97. Non concerné (ne pas suggérer)
☐ 98. Ne sait pas (ne pas suggérer)
☐ 99. Refus de réponse (ne pas suggérer)

E.3.2. Votre mère
☐1 Oui, vu
☐2 Oui, parlé au téléphone
☐3 Oui, échangé des mails
☐4 Non
☐ 97. Non concerné (ne pas suggérer)
☐ 98. Ne sait pas (ne pas suggérer)
☐ 99. Refus de réponse (ne pas suggérer)

E3.2.2 doit être posée si E 3.2 =1, 2,3, ou 4
E3.2.2 Votre mère connaît-il votre séropositivité ?
☐1 Oui
☐2 Non, elle ne le sait pas
☐3 Vous ne savez pas s'elle le sait
☐ 97. Non concerné (ne pas suggérer)
☐ 98. Ne sait pas (ne pas suggérer)
☐ 99. Refus de réponse (ne pas suggérer)

E.3.3. Les parents de votre conjoint
☐1 Oui, vu
☐2 Oui, parlé au téléphone
☐3 Oui, échangé des mails
☐4 Non
☐ 97. Non concerné (ne pas suggérer)
☐ 98. Ne sait pas (ne pas suggérer)
☐ 99. Refus de réponse (ne pas suggérer)

E3.3.2 doit être posée si E 3.3 =1, 2,3, ou 4
E3.3.2 Les parents de votre conjoint connaissent-ils votre séropositivité ?
☐1 Oui
☐2 Non, ils ne le savent pas
☐3 Vous ne savez pas s'ils savent
☐ 97. Non concerné (ne pas suggérer)
☐ 98. Ne sait pas (ne pas suggérer)
☐ 99. Refus de réponse (ne pas suggérer)

Si A3=0 ou 97, 98, 99 ne pas poser la question E3.4
E.3.4. Vos enfants
☐1 Oui, vu
☐2 Oui, parlé au téléphone
☐3 Oui, échangé des mails
☐4 Non
☐ 97. Non concerné (ne pas suggérer)
☐ 98. Ne sait pas (ne pas suggérer)
☐ 99. Refus de réponse (ne pas suggérer)

E3.4.2 doit être posée si E 3.4 =1, 2,3, ou 4
E3.4.2 Vos enfants ou un de vos enfants connaissent-ils votre séropositivité ?
☐$_1$ Oui
☐$_2$ Non, ils ne le savent pas
☐$_3$ Vous ne savez pas s'ils savent
☐ $_{97}$. Non concerné (ne pas suggérer)
☐ $_{98}$. Ne sait pas (ne pas suggérer)
☐ $_{99}$. Refus de réponse (ne pas suggérer)

E.3.5. Votre frère/un de vos frères ou votre sœur/une de vos soeurs
☐$_1$ Oui, vu
☐$_2$ Oui, parlé au téléphone
☐$_3$ Oui, échangé des mails
☐$_4$ Non
☐ $_{97}$. Non concerné (ne pas suggérer)
☐ $_{98}$. Ne sait pas (ne pas suggérer)
☐ $_{99}$. Refus de réponse (ne pas suggérer)

E3.5.2 doit être posée si E 3.5 =1, 2,3, ou 4
E3.5.2 Vos frères ou sœurs ou l'un d'entre eux connaissent-ils votre séropositivité ?
☐$_1$ Oui
☐$_2$ Non, ils ne le savent pas
☐$_3$ Vous ne savez pas s'ils savent
☐ $_{97}$. Non concerné (ne pas suggérer)
☐ $_{98}$. Ne sait pas (ne pas suggérer)
☐ $_{99}$. Refus de réponse (ne pas suggérer)

E.3.7. D'autres parents qui vous sont proches
☐$_1$ Oui, vu
☐$_2$ Oui, parlé au téléphone
☐$_3$ Oui, échangé des mails
☐$_4$ Non
☐ $_{97}$. Non concerné (ne pas suggérer)
☐ $_{98}$. Ne sait pas (ne pas suggérer)
☐ $_{99}$. Refus de réponse (ne pas suggérer)

E3.7.2 doit être posée si E 3.7 =1, 2,3, ou 4
E3.7.2 Ces parents proches ou l'un d'entre eux connaissent-ils votre séropositivité ?
☐$_1$ Oui
☐$_2$ Non, il ne le sait pas
☐$_3$ Vous ne savez pas s'il le sait
☐ $_{97}$. Non concerné (ne pas suggérer)
☐ $_{98}$. Ne sait pas (ne pas suggérer)
☐ $_{99}$. Refus de réponse (ne pas suggérer)

E.4 Est-ce que vous vous sentez- seul(e)
☐$_1$ Oui
☐$_2$ Non
☐ $_{97}$. Non concerné (ne pas suggérer)
☐ $_{98}$. Ne sait pas (ne pas suggérer)
☐ $_{99}$. Refus de réponse (ne pas suggérer)

Nous allons maintenant parler plus particulièrement de vos expériences avec votre entourage à propos de votre infection VIH

E.5 Vous êtes-vous déjà senti rejeté à cause de votre séropositivité ?
☐$_1$ Oui
☐$_2$ Non
☐ $_{97}$. Non concerné (ne pas suggérer)
☐ $_{98}$. Ne sait pas (ne pas suggérer)
☐ $_{99}$. Refus de réponse (ne pas suggérer)

E.6 Est-il déjà arrivé que des amis ou des membres de votre famille hésitent à vous embrasser ou à vous serrer la main, ou à vous laisser embrasser leurs enfants, à cause de votre séropositivité ?

☐$_1$ Oui
☐$_2$ Non
☐ $_{97}$. Non concerné (ne pas suggérer)
☐ $_{98}$. Ne sait pas (ne pas suggérer)
☐ $_{99}$. Refus de réponse (ne pas suggérer)

Toujours à propos de votre entourage

E.8 <u>Actuellement</u>, y a-t-il quelqu'un sur qui vous pouvez compter pour discuter de choses personnelles ou prendre une décision difficile ?
☐$_1$ Oui
☐$_2$ Non
☐ $_{97}$. Non concerné (ne pas suggérer)
☐ $_{98}$. Ne sait pas (ne pas suggérer)
☐ $_{99}$. Refus de réponse (ne pas suggérer)
☐ $_{99}$. Refus de réponse (ne pas suggérer)

E.9 Au cours des 12 derniers mois, avez-vous reçu le soutien dont vous aviez besoin pour affronter les situations difficiles ou vous remonter le moral ?
UNE SEULE REPONSE POSSIBLE

☐ 1 Oui
☐ 2 Oui mais pas assez
☐ 3 Non
☐ 4 Pas besoin de soutien
☐ 97. Non concerné (ne pas suggérer)
☐ 98. Ne sait pas (ne pas suggérer)
☐ 99. Refus de réponse (ne pas suggérer)

E.10 En dehors de votre conjoint, Est-ce qu'il y a quelqu'un sur qui vous pouvez compter pour vous aider matériellement (argent, tâches quotidiennes, garde des enfants, etc.) ?
☐ 1 Oui
☐ 2 Non
☐ 97. Non concerné (ne pas suggérer)
☐ 98. Ne sait pas (ne pas suggérer)
☐ 99. Refus de réponse (ne pas suggérer)

E.11 Au cours des 12 derniers mois, avez-vous reçu l'aide matérielle (argent, tâches quotidiennes, garde des enfants, etc.) dont vous aviez besoin ?
ENQ:ENUMERER
☐ 1 Oui
☐ 2 Oui mais pas assez
☐ 3 Non
☐ 4 Pas besoin d'aide matérielle
☐ 97. Non concerné (ne pas suggérer)
☐ 98. Ne sait pas (ne pas suggérer)
☐ 99. Refus de réponse (ne pas suggérer)

E.12 Avez-vous dans votre entourage une (ou des) personne(s) âgée(s) de plus de 65 ans qui a (ont) besoin d'aide pour les activités courantes de la vie quotidienne?
☐ 1 Oui
☐ 2 Non
☐ 97. Non concerné (ne pas suggérer)
☐ 98. Ne sait pas (ne pas suggérer)
☐ 99. Refus de réponse (ne pas suggérer)

Si oui E12 poser la question E.13
E.13 Apportez-vous régulièrement de l'aide à cette (ces) personne(s)?
☐ 1 Oui
☐ 2 Non
☐ 97. Non concerné (ne pas suggérer)
☐ 98. Ne sait pas (ne pas suggérer)
☐ 99. Refus de réponse (ne pas suggérer)

Nous allons maintenant parler des circonstances dans lesquelles il a pu arriver que quelqu'un vous manque d'égards, vous parle mal ou vous traite de façon injuste ou déplacée.

E.14 Au cours des 2 dernières années, est-il arrivé qu'on vous traite mal dans votre famille ?
☐ 1 Oui
☐ 2 Non
☐ 97. Non concerné (ne pas suggérer)
☐ 98. Ne sait pas (ne pas suggérer)
☐ 99. Refus de réponse (ne pas suggérer)

E.15 Au cours des 2 dernières années, est-il arrivé qu'on vous traite mal à l'occasion d'une fête, une soirée ou un événement familial ?
☐ 1 Oui
☐ 2 Non
☐ 97. Non concerné (ne pas suggérer)
☐ 98. Ne sait pas (ne pas suggérer)
☐ 99. Refus de réponse (ne pas suggérer)

Si E14=1 ou E15=1 → poser la question E16
E.16 Pensez vous que c'était lié à :
PLUSIEURS REPONSES POSSIBLES
☐1 Vos origines ou votre nationalité
☐2 Votre couleur de peau
☐3 Votre sexe (le fait d'être un homme ou une femme)
☐4 Votre orientation sexuelle
☐5 Votre séropositivité
☐6 Votre usage d'alcool
☐7 Votre usage de drogue actuel ou passé
☐8votre façon de vous habiller
☐9 Le lieu ou vous vivez, la réputation de votre quartier
☐10 Parce que vous êtes à la CMU ou à l'AME
☐11 Autre **(à préciser)**
☐ 97. Non concerné (ne pas suggérer)
☐ 98. Ne sait pas (ne pas suggérer)
☐ 99. Refus de réponse (ne pas suggérer)

E.17 Au cours des 2 dernières années, êtes vous allé à la poste ?
☐ 1 Oui
☐ 2 Non
☐ 97. Non concerné (ne pas suggérer)
☐ 98. Ne sait pas (ne pas suggérer)
☐ 99. Refus de réponse (ne pas suggérer)

Si E17=1→ E.18

E.18 Vous est-il arrivé d'être mal reçu ou mal traité à la poste ?

- ☐ 1 Oui
- ☐ 2 Non
- ☐ 97. Non concerné (ne pas suggérer)
- ☐ 98. Ne sait pas (ne pas suggérer)
- ☐ 99. Refus de réponse (ne pas suggérer)

E.19 Au cours des 2 dernières années, êtes vous allé à la préfecture?

- ☐ 1 Oui
- ☐ 2 Non
- ☐ 97. Non concerné (ne pas suggérer)
- ☐ 98. Ne sait pas (ne pas suggérer)
- ☐ 99. Refus de réponse (ne pas suggérer)

Si E19=1→ E.20

E.20 Vous est-il arrivé d'être mal reçu ou mal traité à la préfecture ?

- ☐ 1 Oui
- ☐ 2 Non
- ☐ 97. Non concerné (ne pas suggérer)
- ☐ 98. Ne sait pas (ne pas suggérer)
- ☐ 99. Refus de réponse (ne pas suggérer)

Si E18=1 ou E20=1 → poser la question E.21

E.21 Pensez vous que c'était lié à:

MONTRER CARTE DISCRIMINANTE, PLUSIEURS REPONSES POSSIBLES. COCHEZLES REPONSES CORRESPONDANT EVENTUELLEMENT A PLUSIEURS EVENEMENTS.

- ☐1 Vos origines ou votre nationalité
- ☐2 Votre couleur de peau
- ☐3 Votre sexe (le fait d'être un homme ou une femme)
- ☐4 Votre orientation sexuelle
- ☐5 Votre séropositivité
- ☐6 Votre usage d'alcool
- ☐7 Votre usage de drogue actuel ou passé
- ☐8 Votre façon de vous habiller
- ☐9 Le lieu ou vous vivez, la réputation de votre quartier
- ☐10 Parce que vous êtes à la CMU ou à l'AME
- ☐11 Autre **(à préciser)**
- ☐ 97. Non concerné (ne pas suggérer)
- ☐ 98. Ne sait pas (ne pas suggérer)
- ☐ 99. Refus de réponse (ne pas suggérer)

E.22 Depuis 2 ans, avez-vous fréquenté des lieux de loisirs comme une discothèque, un bar, un restaurant, un hôtel ?

- ☐1. Oui
- ☐2. Non
- ☐ 97. Non concerné (ne pas suggérer)
- ☐ 98. Ne sait pas (ne pas suggérer)
- ☐ 99. Refus de réponse (ne pas suggérer)

Si E22=1 → poser la question E.23

E.23 Vous est-il arrivé qu'on vous refuse l'entrée d'un de ces lieux ?

- ☐1. Oui
- ☐2. Non
- ☐ 97. Non concerné (ne pas suggérer)
- ☐ 98. Ne sait pas (ne pas suggérer)
- ☐ 99. Refus de réponse (ne pas suggérer)

Si E23=1 → poser la question E.24

E.24 Pensez vous que c'était lié à :

MONTRER CARTE DISCRIMINANTE, PLUSIEURS REPONSES POSSIBLES. COCHEZLES REPONSES CORRESPONDANT EVENTUELLEMENT A PLUSIEURS EVENEMENTS.

- ☐1 Vos origines ou votre nationalité
- ☐2 Votre couleur de peau
- ☐3 Votre sexe (le fait d'être un homme ou une femme)
- ☐4 Votre orientation sexuelle
- ☐5 Votre séropositivité
- ☐6 Votre usage d'alcool
- ☐7 Votre usage de drogue actuel ou passé
- ☐8 Votre façon de vous habiller
- ☐9 Le lieu ou vous vivez, la réputation de votre quartier
- ☐10 Parce que vous êtes à la CMU ou à l'AME
- ☐11 Autre **(à préciser)**
- ☐ 97. Non concerné (ne pas suggérer)
- ☐ 98. Ne sait pas (ne pas suggérer)
- ☐ 99. Refus de réponse (ne pas suggérer)

En France, quelques personnes ont été condamnées à des peines de prison pour avoir caché leur séropositivité à leur partenaire et l'avoir contaminé.

E.25 Le saviez-vous ?

- ☐1 Oui
- ☐2 Non
- ☐ 97. Non concerné (ne pas suggérer)
- ☐ 98. Ne sait pas (ne pas suggérer)
- ☐ 99. Refus de réponse (ne pas suggérer)

E.26 Est-ce que cela vous inquiète ?
☐1 Oui
☐2 Non
☐ 97. Non concerné (ne pas suggérer)
☐ 98. Ne sait pas (ne pas suggérer)
☐ 99. Refus de réponse (ne pas suggérer)

E.27 Est-ce que vous-même, vous avez été tenté de porter plainte contre la personne qui vous a contaminé ?
UNE SEULE REPONSE POSSIBLE
☐1 Oui, vous avez porté plainte
☐2 Oui, vous avez été tenté mais vous ne l'avez pas fait
☐3 Non
☐ 97. Non concerné (ne pas suggérer)
☐ 98. Ne sait pas (ne pas suggérer)
☐ 99. Refus de réponse (ne pas suggérer)

E.28 Est-ce que quelqu'un a déjà menacé de porter plainte contre vous parce qu'il pensait que vous lui aviez transmis le VIH ?
☐1 Oui
☐2 Non
☐ 97. Non concerné (ne pas suggérer)
☐ 98. Ne sait pas (ne pas suggérer)
☐ 99. Refus de réponse (ne pas suggérer)

E.29 Combien de personnes vivant avec le VIH connaissez-vous personnellement ?
/__/__/__/__/ → *sur 4 positions*

Uniquement si la personne ne réussit pas à donner un chiffre à E29 poser E29.1- si E29=9998 ou 9999
E.29.1 Combien environ de personnes vivant avec le VIH connaissez-vous personnellement environ ?
UNE SEULE REPONSE POSSIBLE
☐1 Aucune
☐2 Une seule
☐3 2 à 5
☐4 6 à 10
☐5 Plus de 10
☐ 97. Non concerné (ne pas suggérer)
☐ 98. Ne sait pas (ne pas suggérer)
☐ 99. Refus de réponse (ne pas suggérer)

Nous allons maintenant parler plus généralement de la façon dont vous vivez votre maladie

Je vais vous lire plusieurs propositions, et vous me direz à chaque fois dans quelle mesure elles s'appliquent à vous.

E.30.1 Il m'arrive d'éviter les gens qui savent que je suis séropositif parce que je pense qu'ils ont une mauvaise opinion de moi.
UNE SEULE REPONSE POSSIBLE
☐ 1 s'applique tout à fait à vous
☐ 2 un peu
☐ 3 pas vraiment
☐ 4 pas du tout à vous
☐ 97. Non concerné (ne pas suggérer)
☐ 98. Ne sait pas (ne pas suggérer)
☐ 99. Refus de réponse (ne pas suggérer)

E.30.2 Je dois faire mes preuves davantage que les autres, parce que je suis séropositif.
UNE SEULE REPONSE POSSIBLE
☐ 1 s'applique tout à fait à vous
☐ 2 un peu
☐ 3 pas vraiment
☐ 4 pas du tout à vous
☐ 97. Non concerné (ne pas suggérer)
☐ 98. Ne sait pas (ne pas suggérer)
☐ 99. Refus de réponse (ne pas suggérer)

E.30.3 J'ai parfois honte d'être séropositif
UNE SEULE REPONSE POSSIBLE
☐ 1 s'applique tout à fait à vous
☐ 2 un peu
☐ 3 pas vraiment
☐ 4 pas du tout à vous
☐ 97. Non concerné (ne pas suggérer)
☐ 98. Ne sait pas (ne pas suggérer)
☐ 99. Refus de réponse (ne pas suggérer)

E.30.4 Si je devais donner un conseil à un ami séropositif, je lui dirais de cacher sa séropositivité.
UNE SEULE REPONSE POSSIBLE
☐ 1 s'applique tout à fait à vous
☐ 2 un peu
☐ 3 pas vraiment
☐ 4 pas du tout à vous
☐ 97. Non concerné (ne pas suggérer)
☐ 98. Ne sait pas (ne pas suggérer)
☐ 99. Refus de réponse (ne pas suggérer)

E.30.5 Si je devais révéler ma séropositivité pour postuler à un emploi, je préférerais ne pas aller à l'entretien d'embauche
UNE SEULE REPONSE POSSIBLE
☐ 1 s'applique tout à fait à vous
☐ 2 un peu
☐ 3 pas vraiment
☐ 4 pas du tout à vous
☐ 97. Non concerné (ne pas suggérer)
☐ 98. Ne sait pas (ne pas suggérer)
☐ 99. Refus de réponse (ne pas suggérer)

D’une façon plus générale, pouvez-vous me dire si vous êtes tout à fait d’accord, plutôt d’accord, plutôt pas d’accord ou pas du tout d’accord, avec les affirmations suivantes :

E35.1 Beaucoup de gens se méfient des personnes séropositives.
UNE SEULE REPONSE POSSIBLE

- ☐$_1$ tout à fait d’accord
- ☐$_2$ plutôt d’accord
- ☐$_3$ plutôt pas d’accord
- ☐$_4$ pas du tout d’accord
- ☐ $_{97}$. Non concerné (ne pas suggérer)
- ☐ $_{98}$. Ne sait pas (ne pas suggérer)
- ☐ $_{99}$. Refus de réponse (ne pas suggérer)

E35.2Beaucoup de gens n’engageraient pas une personne séropositive pour s’occuper de leurs enfants.
UNE SEULE REPONSE POSSIBLE

- ☐$_1$ tout à fait d’accord
- ☐$_2$ plutôt d’accord
- ☐$_3$ plutôt pas d’accord
- ☐$_4$ pas du tout d’accord
- ☐ $_{97}$. Non concerné (ne pas suggérer)
- ☐ $_{98}$. Ne sait pas (ne pas suggérer)
- ☐ $_{99}$. Refus de réponse (ne pas suggérer)

E35.3 Beaucoup de gens pensent que la séropositivité est une sorte de punition pour des fautes qu’on a commises.
UNE SEULE REPONSE POSSIBLE

- ☐$_1$ tout à fait d’accord
- ☐$_2$ plutôt d’accord
- ☐$_3$ plutôt pas d’accord
- ☐$_4$ pas du tout d’accord
- ☐ $_{97}$. Non concerné (ne pas suggérer)
- ☐ $_{98}$. Ne sait pas (ne pas suggérer)
- ☐ $_{99}$. Refus de réponse (ne pas suggérer)

E35.4 Beaucoup de gens refuseraient de flirter avec / draguer une personne séropositive.
UNE SEULE REPONSE POSSIBLE

- ☐$_1$ tout à fait d’accord
- ☐$_2$ plutôt d’accord
- ☐$_3$ plutôt pas d’accord
- ☐$_4$ pas du tout d’accord
- ☐ $_{97}$. Non concerné (ne pas suggérer)
- ☐ $_{98}$. Ne sait pas (ne pas suggérer)
- ☐ $_{99}$. Refus de réponse (ne pas suggérer)

MODULE F : VIE AFFECTIVE, CONJUGALE ET SEXUELLE

Je vais maintenant vous poser des questions plus personnelles. Certaines questions vont vous paraître très intimes, mais comme je vous l'ai dit, cette enquête est strictement confidentielle et anonyme. Il se peut que certaines questions ne vous concernent pas directement, dites le moi simplement.

F1. Vous vous définissez comme :
UNE SEULE REPONSE POSSIBLE
☐ 1 Hétérosexuel(le)
☐ 2 Bisexuel(le)
☐ 3 Gay/Homosexuel
☐ 4 Lesbienne/homosexuelle
☐ 5 Transsexuel/transgenre ➔ F1a
☐ 6 Vous refusez de vous définir par votre orientation sexuelle
☐ 97 Non concerné (ne pas suggérer)
☐ 98 Ne sait pas (ne pas suggérer)
☐ 99 Refus de réponse (ne pas suggérer)

Si A1=3 ou F1=5
F1a) Vous êtes devenus :
☐ 1 Femme vers homme
☐ 2 Homme vers femme
☐ 97 Non concerné (ne pas suggérer)
☐ 98 Ne sait pas (ne pas suggérer)
☐ 99 Refus de réponse (ne pas suggérer)

F2. Au cours de votre vie, avec combien de partenaires différent(e)s avez-vous eu des relations sexuelles (y compris votre conjoint/e actuel/le)?
UNE SEULE REPONSE POSSIBLE

F2.a) Combien d'hommes :
☐ 1 0
☐ 2 1
☐ 3 2-5
☐ 4 6-10
☐ 5 11-20
☐ 6 21-50
☐ 7 50-100
☐ 8 100-500
☐ 9 Plus de 500
☐ 97 Non concerné (ne pas suggérer)
☐ 98 Ne sait pas (ne pas suggérer)
☐ 99 Refus de réponse (ne pas suggérer)

F2.b) Combien de femmes :
☐ 1 0
☐ 2 1
☐ 3 2-5
☐ 4 6-10
☐ 5 11-20
☐ 6 21-50
☐ 7 50-100
☐ 8 100-500
☐ 9 Plus de 500
☐ 97 Non concerné (ne pas suggérer)
☐ 98 Ne sait pas (ne pas suggérer)
☐ 99 Refus de réponse (ne pas suggérer)

Si F2.b=1 : (i.e. 0 femmes) ne pas passer les parties D1, D2 et D3 (répondants hommes hétéros)Pour tous : Si F2.a=F2.b={0, refus,NSP}, il faut passer les questionsF3, F4, F5 et F7, puis aller en E.1 puis en E.3 si F5=1, et en E.2 puis E.3 sinon.

F3. A quel âge avez-vous eu votre premier rapport sexuel (ce que vous considérez comme votre premier rapport sexuel) ?

Si F2.a ≠ 1 ou 10 ou 11
F3.a Avec un homme
/_____/ → sur 2 positions et sans seuil minimum ➔ **SI F3.a) bis**

SI F3.a ≠ 97
F3.a) bis. Aviez-vous utilisé un préservatif ?
☐ 1 Oui
☐ 2 Non
☐ 97. Non concerné (ne pas suggérer)
☐ 98. Ne sait pas (ne pas suggérer)
☐ 99. Refus de réponse (ne pas suggérer)

Si F2.b ≠ 1 ou 10 ou 11
F3.b) Avec une femme :
/_____/ → sur 2 positions et sans seuil minimum ➔ **F3.b)bis**

SI F3.b ≠ 97
F3.b) bis Aviez-vous utilisé un préservatif ?
☐ 1 Oui
☐ 2 Non
☐ 97. Non concerné (ne pas suggérer)
☐ 98. Ne sait pas (ne pas suggérer)
☐ 99. Refus de réponse (ne pas suggérer)

Si A.1=1
F4. Etes-vous circoncis ?
- ☐ 1 Oui
- ☐ 2 Non
- ☐ 97. Non concerné (ne pas suggérer)
- ☐ 98. Ne sait pas (ne pas suggérer)
- ☐ 99. Refus de réponse (ne pas suggérer)

Si F2a≠1 ou F2b≠1 alors poser F5
F5. Au cours des 12 derniers mois, avez-vous eu des rapports sexuels ?
- ☐ 1 Oui ➔ F7
- ☐ 2 Non ➔ questions F8 et F9
- ☐ 97 Non concerné (ne pas suggérer) ➔ questions F8 et F9
- ☐ 98 Ne sait pas (ne pas suggérer) ➔ questions F8 et F9
- ☐ 99 Refus de réponse (ne pas suggérer) ➔ F109

Si F5 ≠ 1 : (i.e. pas de rapports sexuels) ne pas poser F8.e ; F11 et F16 à F22, ainsi que F9.e ; F82 et F87 à F95 (passer à la partie E après avoir posé F13 à F15 et/ou F83 à F86 selon les cas)
F5 : Si F5= « non concerné » il faut quand même passer F8 et F9 (comme pour NSP)

A tous ceux qui ont eu des rapports sexuels au cours des 12 derniers mois (F5=1)
F7. Au cours des 12 derniers mois, avez-vous eu des rapports sexuels en payant et/ou en étant payé :
PLUSIEURS REPONSES POSSIBLES
- ☐ 1 En payant
- ☐ 2 En étant payé
- ☐ 3 Non
- ☐ 98 Ne sait pas (ne pas suggérer)
- ☐ 99 Refus de réponse (ne pas suggérer)

Si F5=1 ou 2 ou 98 et F2.a ≠ 1 ou 10 ou 11 (au moins un partenaire sexuel masculin au cours de sa vie)
F8. Actuellement, avez-vous un partenaire masculin que vous considérez comme votre partenaire principal, que vous ayez eu des rapports sexuels ou non ?
- ☐ 1 Oui ➔ F8.a)
- ☐ 2 Non ➔ F9
- ☐ 97 Non concerné (ne pas suggérer) ➔ F9
- ☐ 98 Ne sait pas (ne pas suggérer) ➔ F9
- ☐ 99 Refus de réponse (ne pas suggérer) ➔ F9

F8.a) Depuis quand êtes-vous ensemble ?

/____/Année(s)
/___/ Mois

La date recueillie en F8.a (partenaire principal masculin) doit donc être antérieure à 12 mois pour passer les questions ou énoncés suivants : F16, F16.c, F16.d, F16.e, F17, F20, F21, F22, phrase avant F62 et avant F72 (partie C.3.a), F62, F62.b, F62.c, F63, F67, F68, F69 et F70.

F8.b) Quel âge a-t-il ?

/__/ __/ ans

F8.c) De quel pays est-il originaire ?
Non concerné (ne pas suggérer)
Ne sait pas (ne pas suggérer)
Refus de réponse (ne pas suggérer)

F8.d) Vivez-vous sous le même toit ?
- ☐ 1 Oui
- ☐ 2 Non
- ☐ 97. Non concerné (ne pas suggérer)
- ☐ 98. Ne sait pas (ne pas suggérer)
- ☐ 99. Refus de réponse (ne pas suggérer)

si F5=1 et F8=1 et F2a=2, 3, 4, 5, 6, 7,8 ou 9
F8. e) Avez-vous eu des rapports sexuels avec lui au cours des 12 derniers mois
- ☐ 1 Oui
- ☐ 2 Non
- ☐ 97. Non concerné (ne pas suggérer)
- ☐ 98. Ne sait pas (ne pas suggérer)
- ☐ 99. Refus de réponse (ne pas suggérer)

Si F8.e ≠ 1 : (i.e. pas de rapports sexuels avec partenaire principal homme) ne pas poser F11 et F16 à F22 ainsi que F56 et F62 à F70

Si F5=1 ou 2 ou 98 et F2.b ≠ 1 ou 10 ou 11
F9. Actuellement, avez-vous une partenaire féminine que vous considérez comme votre partenaire principal, que vous ayez eu des rapports sexuels ou non ?
- ☐ 1 Oui ➔ F9.a)
- ☐ 2 Non ➔ F10
- ☐ 97. Non concerné (ne pas suggérer) ➔ F10
- ☐ 98. Ne sait pas (ne pas suggérer) ➔ F10
- ☐ 99. Refus de réponse (ne pas suggérer) ➔ F10

Si F9=1
F9.a) Depuis quand êtes-vous ensemble ?

/____/Année(s) /___/ Mois

La date recueillie en F9.a (partenaire principale féminine) doit être antérieure à 12 mois pour passer les questions ou énoncés suivants : phrase avant F87 et avant F97 (partie D.3.a), F87, F87.b, F87.c, F88, F92, F93, F94, F95

Si F9=1et F2.b ≠ 1 ou 10 ou 11
F9.b) Quel âge a-t-elle ?

/__/ __/ ans

Si F9=1et F2.b ≠ 1 ou 10 ou 11
F9.c) De quel pays est-elle originaire ?
- ☐ 97. Non concerné (ne pas suggérer)
- ☐ 98. Ne sait pas (ne pas suggérer)
- ☐ 99. Refus de réponse (ne pas suggérer)

Si F9=1et F2.b ≠ 1 ou 10 ou 11
F9.d) Vivez-vous sous le même toit ?
- ☐ 1 Oui
- ☐ 2 Non
- ☐ 97. Non concerné (ne pas suggérer)
- ☐ 98. Ne sait pas (ne pas suggérer)
- ☐ 99. Refus de réponse (ne pas suggérer)

Si F5=1 et F9=1 et F2b=2,3, 4, 5, 6, 7, 8 ou 9
F9.e) Avez-vous eu des rapports sexuels avec elle au cours des 12 derniers mois ?
- ☐ 1 Oui
- ☐ 2 Non
- ☐ 97. Non concerné (ne pas suggérer)
- ☐ 98. Ne sait pas (ne pas suggérer)
- ☐ 99. Refus de réponse (ne pas suggérer)

Si F9.e ≠ 1 : (i.e. pas de rapports sexuels avec partenaire principale femme) ne pas poser F82 et F87 à F95
Si F8 ou F9 = non concerné ou refus ou NSP : ne pas empêcher le passage des autres parties, partenaires occasionnels etc.

Seulement si A.1=2 et A.2>=1961 et F8=1
F10. Actuellement, avez-vous le projet d'avoir un enfant ?
UNE SEULE REPONSE POSSIBLE

- ☐ 1Oui, votre conjointe est (vous êtes) enceinte → **F10.ab)**
- ☐ 2Oui, vous cherchez actuellement à avoir un enfant
- ☐ 3Oui, vous (et/ou votre conjointe) avez le désir d'avoir un enfant
- ☐ 4 Peut-être plus tard
- ☐ 5 Non
- ☐ 6 Vous ne pouvez pas/plus avoir d'enfant (stérilité, ligature des canaux spermatiques (des trompes) etc.)
- ☐ 97. Non concerné (ne pas suggérer)
- ☐ 98. Ne sait pas (ne pas suggérer)
- ☐ 99. Refus de réponse (ne pas suggérer)

Si F10= 2, 3 ou 4
F10.a) envisagez vous :
PLUSIEURS REPONSES POSSIBLES
- ☐ 1 De faire cet enfant naturellement
- ☐ 2 De pratiquer une auto-insémination
- ☐ 3 D'avoir recours à l'AMP (Assistance médicale à la procréation)
- ☐ 97. Non concerné (ne pas suggérer)
- ☐ 98. Ne sait pas (ne pas suggérer)
- ☐ 99. Refus de réponse (ne pas suggérer)

Si F10=1 poser F10.ab)
F10.ab) Avez-vous :
PLUSIEURS REPONSES POSSIBLES
- ☐ 1 Fait cet enfant naturellement
- ☐ 2 Pratiqué une auto-insémination
- ☐ 3 Eu recours à l'AMP (Assistance médicale à la procréation)
- ☐ 97. Non concerné (ne pas suggérer)
- ☐ 98. Ne sait pas (ne pas suggérer)
- ☐ 99. Refus de réponse (ne pas suggérer)

Attention : ICI, si F5 est différent de 2,3 ou 4 le répondant est dirigé :
- **vers la sous-partie A (si répondant A1=1 ou (F1a=1) et F2a<>1, 10, 11)**
- **vers la sous-partie B (si répondant A1=2 ou (F1a=2) et F2b<>1, 10, 11)**
- **vers la sous-partie C (si répondant A1=2 ou (F1a=2) et F2a<>1, 10, 11)**
- **vers la sous-partie D (si répondant A1=1 ou (F1a=1) et F2b<>1, 10, 11)**
- **109 sinon**

Attention, ces filtres ne sont pas exclusifs (cas des hommes et femmes bisexuels, qui doivent passer pour les hommes bi en A puis D et pour les femmes bi en B puis C), dans cet ordre là.

A - MODULE HSH
(Hommes ou Female to Male [FtoM] ayant eu au moins un partenaire masculin dans l'année)

A.1 - HSH : Partenaire principal

Si **[(A.1=1) ou F1a=1)]** et F8=1 **=> si est un homme ou un homme devenu femme et a un partenaire principal masculin**

Pour les autres, aller au filtre A3

Nous allons maintenant parler de votre partenaire principal masculin.

Si F5=1 ou si F8.e=1
F11. Au cours des 4 dernières semaines, combien avez-vous eu de rapports sexuels avec votre partenaire principal ?

|____| mettre d'abord un champ à remplir par l'enquêteur

Si F11 = 98 ou 99
F11a- Combien environ ?
☐ $_1$Aucun
☐ $_2$1 à 4 rapports sexuels (1 fois par semaine ou moins)
☐ $_3$5 à 8 rapports sexuels (entre 1 et 2 fois par semaine)
☐ $_4$9 à 12 rapports sexuels (entre 2 et 3 fois par semaine)
☐ $_5$13 à 16 rapports sexuels (entre 3 et 4 fois par semaine)
☐ $_6$Plus de 16 rapports sexuels (plus de 4 fois par semaine)
☐ $_{97}$. Non concerné (ne pas suggérer)
☐ $_{98}$. Ne sait pas (ne pas suggérer)
☐ $_{99}$. Refus de réponse (ne pas suggérer)

F12. Votre partenaire est-il circoncis ?
☐ $_1$ Oui
☐ $_2$ Non
☐ $_{97}$. Non concerné (ne pas suggérer)
☐ $_{98}$. Ne sait pas (ne pas suggérer)
☐ $_{99}$. Refus de réponse (ne pas suggérer)

Nous allons maintenant remonter au début de cette relation avec votre partenaire principal masculin

Poser F13 si Année F8.a > 1985
F13. Au début de votre relation, vous étiez :
UNE SEULE REPONSE POSSIBLE
☐ $_1$Séronégatif
☐ $_2$Séropositif
☐ $_3$Vous ne connaissiez pas votre statut sérologique
☐ $_{97}$. Non concerné (ne pas suggérer)
☐ $_{98}$. Ne sait pas (ne pas suggérer)
☐ $_{99}$. Refus de réponse (ne pas suggérer)

F14. Avez-vous utilisé un préservatif lors de votre premier rapport anal avec ce partenaire ?
☐ $_1$ Oui
☐ $_2$ Non
☐ $_{97}$. Non concerné (ne pas suggérer)
☐ $_{98}$. Ne sait pas (ne pas suggérer)
☐ $_{99}$. Refus de réponse (ne pas suggérer)

Si F13=2
F14.a) Comment avez-vous annoncé votre séropositivité à votre partenaire ?
UNE SEULE REPONSE POSSIBLE
☐ $_1$ Vous lui avez dit de vous-même
☐ $_2$ Vous lui avez dit car il vous l'avait demandé
☐ $_3$ Il savait que vous étiez séropositif sans avoir à en parler (ex : vous êtes rencontrés dans un espace réservé aux personnes séropositives (internet, soirées privées etc.))
☐ $_4$ Vous ne lui avez jamais dit
☐ $_{97}$. Non concerné (ne pas suggérer)
☐ $_{98}$. Ne sait pas (ne pas suggérer)
☐ $_{99}$. Refus de réponse (ne pas suggérer)

Si (F13≠2 ou Année(F8.a) < 1985**)**
F14.a) bis Comment avez-vous annoncé votre séropositivité à votre partenaire ?
UNE SEULE REPONSE POSSIBLE
☐ $_1$ Vous lui avez dit de vous-même
☐ $_1$ Vous lui avez dit car il vous l'avait demandé
☐ $_1$ Il l'a su autrement
☐ $_1$ Vous ne lui avez jamais dit
☐ $_{97}$. Non concerné (ne pas suggérer)
☐ $_{98}$. Ne sait pas (ne pas suggérer)
☐ $_{99}$. Refus de réponse (ne pas suggérer)

Si (F13≠2 ou Année(F8.a) < 1985**) Et** F14.a) bis=1 o 2 o 3
F14.b) A quel moment ?
UNE SEULE REPONSE POSSIBLE
☐ $_1$Immédiatement après avoir découvert votre séropositivité
☐ $_2$Dans les trois mois qui ont suivi la découverte de votre séropositivité
☐ $_3$Plus tard dans votre relation
☐ $_{97}$. Non concerné (ne pas suggérer)
☐ $_{98}$. Ne sait pas (ne pas suggérer)
☐ $_{99}$. Refus de réponse (ne pas suggérer)

Si F13=2 et F14.a=1 ou 2

F14.c) A quel moment ?
UNE SEULE REPONSE POSSIBLE
- ☐ 1Avant votre premier rapport sexuel
- ☐ 2Dans les trois premiers mois de votre relation
- ☐ 3Plus tard dans votre relation
- ☐ 97. Non concerné (ne pas suggérer)
- ☐ 98. Ne sait pas (ne pas suggérer)
- ☐ 99. Refus de réponse (ne pas suggérer)

Poser F15 si Année F8.a > 1985
F15. Toujours au début de votre relation, quel était le statut sérologique de votre partenaire ?
UNE SEULE REPONSE POSSIBLE
- ☐ 1Séronégatif
- ☐ 2Séropositif
- ☐ 3Vous ne saviez pas
- ☐ 4Il ne connaissait pas son statut
- ☐ 97. Non concerné (ne pas suggérer)
- ☐ 98. Ne sait pas (ne pas suggérer)
- ☐ 99. Refus de réponse (ne pas suggérer)

Si F15≠2
F15.a) Votre partenaire est-il séropositif aujourd'hui ?
- ☐ 1 Oui
- ☐ 2 Non
- ☐ 97. Non concerné (ne pas suggérer)
- ☐ 98. Ne sait pas (ne pas suggérer)
- ☐ 99. Refus de réponse (ne pas suggérer)

Maintenant nous allons aborder vos rapports sexuels avec ce partenaire principal dans les douze derniers mois

F5=1 et F8.e=1 et date (F8a) - date (système/aujourd'hui) >12 mois sinon aller au filtre au début de la partie A.3) => sont interrogées, les personnes qui ont un partenaire masculin principal depuis plus de 12 mois (couples formés depuis plus d'un an aux questions portant sur la dernière année

F16. Au cours des 12 derniers mois, avez-vous pratiqué avec votre partenaire principal masculin :

F16.a) La masturbation réciproque
UNE SEULE REPONSE POSSIBLE
- ☐ 1Jamais
- ☐ 2Rarement
- ☐ 3Parfois
- ☐ 4Souvent
- ☐ 5Toujours
- ☐ 97. Non concerné (ne pas suggérer)
- ☐ 98. Ne sait pas (ne pas suggérer)
- ☐ 99. Refus de réponse (ne pas suggérer)

F16.b) La fellation
UNE SEULE REPONSE POSSIBLE
- ☐ 1Jamais ➔ F16.c)
- ☐ 2Rarement ➔ **F16.b) bis**
- ☐ 3Parfois ➔ **F16.b) bis**
- ☐ 4Souvent ➔ **F16.b) bis**
- ☐ 5Toujours ➔ **F16.b) bis**
- ☐ 97Non concerné (ne pas suggérer) ➔ F16.c)
- ☐ 98Ne sait pas (ne pas suggérer) ➔ F16.c)
- ☐ 99Refus de réponse (ne pas suggérer) ➔ F16.c)

Si F16b=2, 3,4 ou 5
F16.b)bis Met-il un préservatif quand vous le sucez ?
UNE SEULE REPONSE POSSIBLE
- ☐ 1Jamais ➔ **F16.b) ter**
- ☐ 2Presque jamais ➔ **F16.b) ter**
- ☐ 3Parfois ➔ **F16.b) ter**
- ☐ 4Presque toujours ➔ **F16.b) ter**
- ☐ 5Toujours ➔ F16.b) qua
- ☐ 97Non concerné (ne pas suggérer) ➔ F16.b) qua
- ☐ 98Ne sait pas (ne pas suggérer) ➔ F16.b) qua
- ☐ 99Refus de réponse (ne pas suggérer) ➔ F16.b) qua

Si F16b)bis=1,2, 3 ou 4
F16.b)ter Lui arrive-t-il d'éjaculer dans votre bouche [sans préservatif] **?**
UNE SEULE REPONSE POSSIBLE
- ☐ 1Jamais
- ☐ 2Presque jamais
- ☐ 3Parfois
- ☐ 4Presque toujours
- ☐ 5Toujours
- ☐ 97. Non concerné (ne pas suggérer)
- ☐ 98. Ne sait pas (ne pas suggérer)
- ☐ 99. Refus de réponse (ne pas suggérer)

Si F16b)bis>=1
F16.b)qua Mettez-vous un préservatif pour vous faire sucer ?
UNE SEULE REPONSE POSSIBLE
- ☐ 1Jamais ➔ **F16.b)Quin**
- ☐ 2Presque jamais ➔ **F16.b)Quin**
- ☐ 3Parfois ➔ **F16.b)Quin**
- ☐ 4Presque toujours ➔ **F16.b)Quin**
- ☐ 5Toujours ➔ F16.c)
- ☐ 97Non concerné (ne pas suggérer) ➔ F16.c)

☐ 98 Ne sait pas (ne pas suggérer) ➔ F16.c)
☐ 99Refus de réponse (ne pas suggérer) ➔ F16.c)

Si F16b)qua=1,2, 3 ou 4
F16.b)Quin Vous arrive-t-il d'éjaculer dans sa bouche [sans préservatif] **?**
UNE SEULE REPONSE POSSIBLE
☐ 1Jamais
☐ 2Presque jamais
☐ 3Parfois
☐ 4Presque toujours
☐ 5Toujours
☐ 97Non concerné (ne pas suggérer)
☐ 98 Ne sait pas (ne pas suggérer)
☐ 99 Refus de réponse (ne pas suggérer)

F16.c) <u>Au cours des douze derniers mois</u> avez-vous pratiqué la sodomie active [vous l'avez pénétré dans l'anus] ?
UNE SEULE REPONSE POSSIBLE
☐ 1Jamais ➔ F16.d)
☐ 2Rarement ➔ **F16.c) bis**
☐ 3Parfois ➔ **F16.c) bis**
☐ 4Souvent ➔ **F16.c) bis**
☐ 5Toujours ➔ **F16.c) bis**
☐ 97 Non concerné (ne pas suggérer) ➔ F16.d)
☐ 98 Ne sait pas (ne pas suggérer) ➔ F16.d)
☐ 99 Refus de réponse (ne pas suggérer) ➔ F16.d)

Si F16c) = 2, 3,4 ou 5
F16.c) bis Mettez-vous un préservatif pour le sodomiser [pénétrer dans l'anus]**?**
UNE SEULE REPONSE POSSIBLE
☐ 1 Jamais ➔ **F16.d) ter**
☐ 2 Presque jamais ➔ **F16.d) ter**
☐ 3 Parfois ➔ **F16.d) ter**
☐ 4 Presque toujours ➔ **F16.d) ter**
☐ 5 Toujours ➔ F16.e)
☐ 97 Non concerné (ne pas suggérer) ➔ F16.e)
☐ 98 Ne sait pas (ne pas suggérer) ➔ F16.e)
☐ 99 Refus de réponse (ne pas suggérer) ➔ F16.e)

Si F16c)bis = 1 ,2, 3ou 4
F16.c) ter Lorsque vous le sodomisez, pratiquez-vous le retrait avant d'éjaculer?
UNE SEULE REPONSE POSSIBLE
☐ 1 Jamais
☐ 2Presque jamais
☐ 3Parfois
☐ 4Presque toujours
☐ 5Toujours
☐ 97Non concerné (ne pas suggérer)
☐ 98 Ne sait pas (ne pas suggérer)
☐ 99 Refus de réponse (ne pas suggérer)

F16.c) qua Et la dernière fois que vous l'avez sodomisé [pénétré dans l'anus]**, avez-vous utilisé un préservatif ?**
☐ 1 Oui
☐ 2 Non
☐ 97. Non concerné (ne pas suggérer)
☐ 98. Ne sait pas (ne pas suggérer)
☐ 99. Refus de réponse (ne pas suggérer)

F16.d) Toujours <u>au cours des douze derniers mois</u>, avez-vous pratiqué la sodomie passive [votre partenaire vous a pénétré dans l'anus]**?**
UNE SEULE REPONSE POSSIBLE
☐ 1 Jamais ➔ F16.e)
☐ 2 Rarement ➔ **F16.d) bis**
☐ 3 Parfois ➔ **F16.d) bis**
☐ 4 Souvent ➔ **F16.d) bis**
☐ 5 Toujours ➔ **F16.d) bis**
☐ 97 Non concerné (ne pas suggérer) ➔ F16.e)
☐ 98 Ne sait pas (ne pas suggérer) ➔ F16.e)
☐ 99Refus de réponse (ne pas suggérer) ➔ F16.e)

Si F16d=2, 3, 4 ou 5
F16.d)bis Votre partenaire met-il un préservatif pour vous sodomiser?
UNE SEULE REPONSE POSSIBLE
☐ 1 Jamais ➔ F16.d) ter
☐ 2 Presque jamais ➔ F16.d) ter
☐ 3 Parfois ➔ F16.d) ter
☐ 4 Presque toujours ➔ F16.d) ter
☐ 5 Toujours ➔ F16.e)
☐ 97 Non concerné (ne pas suggérer) ➔ F16.e)
☐ 98 Ne sait pas (ne pas suggérer) ➔ F16.e)
☐ 99 Refus de réponse (ne pas suggérer) ➔ F16.e)

Si F16dbis=1, 2, 3 ou 4
F16.d) ter Votre partenaire se retire-t-il avant d'éjaculer [dans votre anus]**?**
UNE SEULE REPONSE POSSIBLE
☐ 1 Jamais
☐ 2 Presque jamais
☐ 3 Parfois
☐ 4 Presque toujours
☐ 5 Toujours
☐ 97 Non concerné (ne pas suggérer)
☐ 98 Ne sait pas (ne pas suggérer)
☐ 99 Refus de réponse (ne pas suggérer)

Si F16dter>=1
F16.d) qua Et la dernière fois qu'il vous a sodomisé [pénétré dans l'anus] **a-t-il utilisé un préservatif ?**
☐ 1 Oui
☐ 2 Non
☐ 97. Non concerné (ne pas suggérer)
☐ 98. Ne sait pas (ne pas suggérer)
☐ 99. Refus de réponse (ne pas suggérer)

F16.e) Au cours des douze derniers mois, avez-vous pratiqué le « fist-fucking »?
UNE SEULE REPONSE POSSIBLE
☐ 1 Jamais
☐ 2 Rarement
☐ 3 Souvent
☐ 4 Parfois
☐ 5 Toujours
☐ 97 Non concerné (ne pas suggérer)
☐ 98 Ne sait pas (ne pas suggérer)
☐ 99 Refus de réponse (ne pas suggérer)

F17. Au cours des 12 derniers mois, étiez-vous sous l'effet du ou des produit(s) suivant(s) pendant vos rapports sexuels avec votre partenaire principal :

F17.a) Cannabis ?
☐ 1 Oui ➔ **F17.a)bis**
☐ 2 Non ➔ F17.b)
☐ 97. Non concerné (ne pas suggérer) ➔ F17.b)
☐ 98. Ne sait pas (ne pas suggérer) ➔ F17.b)
☐ 99. Refus de réponse (ne pas suggérer) ➔ F17.b)

F17.a) bis A quelle fréquence ?
UNE SEULE REPONSE POSSIBLE
☐ 1 Rarement
☐ 2 Souvent
☐ 3 Toujours
☐ 97 Non concerné (ne pas suggérer)
☐ 98 Ne sait pas (ne pas suggérer)
☐ 99 Refus de réponse (ne pas suggérer)

F17.b) Ecstasy
☐ 1 Oui ➔ **F17.b) bis**
☐ 2 Non ➔ F17.c)
☐ 97 Non concerné (ne pas suggérer) ➔ F17.c)
☐ 98 Ne sait pas (ne pas suggérer) ➔ F17.c)
☐ 99 Refus de réponse (ne pas suggérer) ➔ F17.c)

F17.b)bis A quelle fréquence ?
UNE SEULE REPONSE POSSIBLE

☐ 1 Rarement
☐ 2 Souvent
☐ 3 Toujours
☐ 97 Non concerné (ne pas suggérer)
☐ 98 Ne sait pas (ne pas suggérer)
☐ 99 Refus de réponse (ne pas suggérer)

F17.c) Cocaïne
☐ 1 Oui ➔ **F17.c) bis**
☐ 2 Non ➔ F17.d)
☐ 97 Non concerné (ne pas suggérer) ➔ F17.d)
☐ 98 Ne sait pas (ne pas suggérer) ➔ F17.d)
☐ 99 Refus de réponse (ne pas suggérer) ➔ F17.d)

F17.c)bis A quelle fréquence ?
UNE SEULE REPONSE POSSIBLE
☐ 1 Rarement
☐ 2 Souvent
☐ 3 Toujours
☐ 97 Non concerné (ne pas suggérer)
☐ 98 Ne sait pas (ne pas suggérer)
☐ 99 Refus de réponse (ne pas suggérer)

F17.d) Poppers
☐ 1 Oui ➔ **F17.d) bis**
☐ 2 Non ➔ F17.e)
☐ 97 Non concerné (ne pas suggérer) ➔ F17.e)
☐ 98 Ne sait pas (ne pas suggérer) ➔ F17.e)
☐ 99 Refus de réponse (ne pas suggérer) ➔ F17.e)

F17.d)bis A quelle fréquence ?
UNE SEULE REPONSE POSSIBLE
☐ 1 Rarement
☐ 2 Souvent
☐ 3 Toujours
☐ 97 Non concerné (ne pas suggérer)
☐ 98 Ne sait pas (ne pas suggérer)
☐ 99 Refus de réponse (ne pas suggérer)

F17.e) Alcool
☐ 1 Oui ➔ **F17.e) bis**
☐ 2 Non ➔ F17.f)
☐ 97 Non concerné (ne pas suggérer) ➔ F17.f)
☐ 98 Ne sait pas (ne pas suggérer) ➔ F17.f)
☐ 99 Refus de réponse (ne pas suggérer) ➔ F17.f)

F17.e)bis A quelle fréquence ?
UNE SEULE REPONSE POSSIBLE
☐ 1 Rarement
☐ 2 Souvent
☐ 3 Toujours
☐ 97 Non concerné (ne pas suggérer)
☐ 98 Ne sait pas (ne pas suggérer)
☐ 99 Refus de réponse (ne pas suggérer)

F17.f) Viagra®, et autres stimulants
☐ 1 Oui ➔ **F17.f) bis**
☐ 2 Non ➔ F17.g)
☐ 97 Non concerné (ne pas suggérer) ➔ F17.g)
☐ 98 Ne sait pas (ne pas suggérer) ➔ F17.g)
☐ 99 Refus de réponse (ne pas suggérer) ➔ F17.g)

F17.f)bis A quelle fréquence ?
UNE SEULE REPONSE POSSIBLE
☐ 1 Rarement
☐ 2 Souvent
☐ 3 Toujours
☐ 97 Non concerné (ne pas suggérer)
☐ 98 Ne sait pas (ne pas suggérer)
☐ 99 Refus de réponse (ne pas suggérer)

F17.g) Autres. Précisez : ________________
☐ 1 Oui ➔ **F17.g) bis**
☐ 2 Non ➔ F18
☐ 97 Non concerné (ne pas suggérer) ➔ F18
☐ 98 Ne sait pas (ne pas suggérer) ➔ F18
☐ 99 Refus de réponse (ne pas suggérer) ➔ F18

F17.g) bis A quelle fréquence ?
UNE SEULE REPONSE POSSIBLE
☐ 1 Rarement
☐ 2 Souvent
☐ 3 Toujours
☐ 97 Non concerné (ne pas suggérer)
☐ 98 Ne sait pas (ne pas suggérer)
☐ 99 Refus de réponse (ne pas suggérer)

F18. Pour vous, utiliser un préservatif lors de la pénétration anale avec votre partenaire principal est : [De manière générale, sans distinction actif/passif]
UNE SEULE REPONSE POSSIBLE
☐ 1 Très difficile
☐ 2 Plutôt difficile
☐ 3 Plutôt facile
☐ 4 Très facile
☐ 97 Non concerné (ne pas suggérer)
☐ 98 Ne sait pas (ne pas suggérer)
☐ 99 Refus de réponse (ne pas suggérer)

F20. Au cours des 12 derniers mois, avez-vous eu d'autres partenaires sexuels masculins que votre partenaire principal ?
☐ 1 Oui ➔ **F20.a)**
☐ 2 Non ➔ F21
☐ 97 Non concerné (ne pas suggérer) ➔ F21
☐ 98 Ne sait pas (ne pas suggérer) ➔ F21
☐ 99 Refus de réponse (ne pas suggérer) ➔ F21

Si F20=1
F20.a) Etait-ce dans le cadre de « sex parties », ou de plan à plusieurs dans le ou lesquels il était présent ?
UNE SEULE REPONSE POSSIBLE
☐ 1 Oui, souvent,
☐ 2 Oui, parfois
☐ 3 Non, jamais
☐ 97 Non concerné (ne pas suggérer)
☐ 98 Ne sait pas (ne pas suggérer)
☐ 99 Refus de réponse (ne pas suggérer)

Si F20=1 Et F20.a = 1 ou 2
F20.b) Lui avez-vous dit que vous aviez eu d'autres partenaires que lui en dehors des « sex parties » où il était présent ?
UNE SEULE REPONSE POSSIBLE
☐ 1 Oui
☐ 2 Non
☐ 3 Pas d'autres partenaires sexuels que ceux rencontrés dans les « sex parties » où il était présent
☐ 97 Non concerné (ne pas suggérer)
☐ 98 Ne sait pas (ne pas suggérer)
☐ 99 Refus de réponse (ne pas suggérer)

Si F20=1 ET F20.a ≠ 1 et 2
F20.c) Lui avez-vous dit que vous aviez eu d'autres partenaires que lui ?
☐ 1 Oui
☐ 2 Non
☐ 97. Non concerné (ne pas suggérer)
☐ 98. Ne sait pas (ne pas suggérer)
☐ 99. Refus de réponse (ne pas suggérer)

F21. Depuis que vous le connaissez, est-il déjà arrivé que votre partenaire principal vous menace ou exerce une pression psychologique sur vous, pour avoir des relations sexuelles ? [Il a, au travers de mots blessant, dénigrant, médisant, rabaissant etc., ou encore au travers de sous-entendus, porté atteinte à votre moral]
UNE SEULE REPONSE POSSIBLE
☐ 1 Oui c'est arrivé dans les 12 derniers mois
☐ 2 Oui mais c'est arrivé il y a plus de 12 derniers mois
☐ 3 Non
☐ 97 Non concerné (ne pas suggérer)
☐ 98 Ne sait pas (ne pas suggérer)
☐ 99 Refus de réponse (ne pas suggérer)

F22. Depuis que vous le connaissez, est-il déjà arrivé que votre partenaire principal utilise la force physique pour avoir des relations sexuelles avec vous, ou qu'il vous impose des gestes sexuels que vous refusiez ?
UNE SEULE REPONSE POSSIBLE
☐ 1 Oui c'est arrivé dans les 12 derniers mois
☐ 2 Oui mais c'est arrivé il y a plus de 12 derniers mois
☐ 3 Non
☐ 97 Non concerné (ne pas suggérer)
☐ 98 Ne sait pas (ne pas suggérer)
☐ 99 Refus de réponse (ne pas suggérer)

A.2 - HSH : Pas de partenaire principal au moment de la passation

(Si [(A.1=1) ou F1a=1)] et F8 ≠ 1)
A vérifier : on doit poser même si F8=3 ou 4 (refus ou NSP)

(Pour les autres : aller au filtre au début de la partie A.3 « HSH : Autres partenaires réguliers et occasionnels »)

Vous m'avez dit que vous n'avez actuellement pas de partenaire principal masculin (ou vous n'avez pas répondu à cette question)

F23. Avez-vous déjà eu un partenaire masculin que vous considériez comme votre partenaire principal ?
☐ 1 Oui ➔ **F23.a)**
☐ 2 Non ➔ Filtre au début de la partie A.3
☐ 97 Non concerné (ne pas suggérer) ➔ Filtre au début de la partie A.3
☐ 98 Ne sait pas (ne pas suggérer) ➔ Filtre au début de la partie A.3
☐ 99 Refus de réponse (ne pas suggérer) ➔ Filtre au début de la partie A.3

Nous allons maintenant parler de cette relation ou de la dernière de ces relations avec un partenaire principal

F23.a) En quelle année cette relation a-t-elle commencé ?
_ _ _ _

F23.b) En quelle année cette relation s'est-elle terminée ?
_ _ _ _

Si F23=1
F23.c) A ce moment là, connaissait-il votre séropositivité ?
☐ 1 Oui ➔ F23.d)
☐ 2 Non ➔ **F23.c) bis**
☐ 3 Vous ne savez pas ➔ **F23.c) bis**
☐ 4 Vous n'étiez pas encore séropositif ➔ F23.d)
☐ 97 Non concerné (ne pas suggérer) ➔ F23.d)
☐ 99 Refus de réponse (ne pas suggérer) ➔ F23.d)

Si F23c= 2 ou 3
F23.c) bis Aviez-vous peur d'être rejeté en le lui disant ?
☐ 1 Oui
☐ 2 Non
☐ 97. Non concerné (ne pas suggérer)
☐ 98. Ne sait pas (ne pas suggérer)
☐ 99. Refus de réponse (ne pas suggérer)

Si F23=1
F 23.d) Connaissiez vous son statut sérologique ?
☐ 1 Oui, il vous a dit qu'il était séropositif
☐ 2 Oui, vous pensez qu'il était séropositif
☐ 3 Oui, vous pensez qu'il était séronégatif
☐ 4 Oui, il vous a dit qu'il était séronégatif
☐ 5 Non, vous ne le saviez pas
☐ 97. Non concerné (ne pas suggérer)
☐ 98. Ne sait pas (ne pas suggérer)
☐ 99. Refus de réponse (ne pas suggérer)

A.3 - HSH : Autres Partenaires réguliers et occasionnels

Si (A.1=1) ou et F1a=1) et

- **(Si F8=1 ET [(F5=1 et F20=1) ou (F5=1 et F8.e ≠ 1)]) ou (F5=1 et date(F8.a)-date (aujourd'hui) < 12 mois))**

Nous allons maintenant parler de vos partenaires sexuels masculins, qui sont différents de votre partenaire principal masculin

Ou

- **(Si F8≠1) et F2.a ≠ {1, 10,11} et F5=1)**

Nous allons maintenant parler de vos partenaires sexuels masculins de ces 12 derniers mois

(Pour les autres : aller au filtre au début de la partie « D - Hommes hétéros)

F6.a) Au cours des 12 derniers mois, combien de partenaires sexuels masculins avez-vous eu en dehors de votre partenaire principal (si vous en avez un)?
/_____/ (→ sur 4 positions)
➔ Si =0, refus ou NSP : aller au filtre au début de la partie « D - Hommes hétéros »

F24. Au cours des 12 derniers mois, comment avez-vous rencontré ces partenaires sexuels masculins ?

☐ 1 Par Internet
☐ 2 Par petites annonces dans la presse
☐ 3 Par réseaux téléphoniques
☐ 4 via une application pour faire des rencontres sur smartphone/iPhone
☐ 5 Dans des saunas,
☐ 6 Dans des backrooms,
☐ 7 Dans des vidéoclubs, des cinémas
☐ 8 Bars ou discothèques
☐ 9 Chez des amis
☐ 10 Dans des associations
☐ 11 Au travail
☐ 12 Lieux extérieurs de drague homo (parc, parking etc.)
☐ 13 Dans la rue et les endroits publics
☐ 14 Sex parties, soirées privées
☐ 97. Non concerné (ne pas suggérer)
☐ 98. Ne sait pas (ne pas suggérer)
☐ 99. Refus de réponse (ne pas suggérer)

F25. Au cours des quatre dernières semaines, avez-vous cherché des partenaires sexuels masculins sur internet?
☐ 1 Jamais
☐ 2 Moins d'une fois par semaine
☐ 3 Une fois par semaine
☐ 4 Plus d'une fois par semaine
☐ 5 Tous les jours ou presque
☐ 97. Non concerné (ne pas suggérer)
☐ 98. Ne sait pas (ne pas suggérer)
☐ 99. Refus de réponse (ne pas suggérer)

F26. Au cours des 12 derniers mois, pour rencontrer des partenaires avez-vous utilisé des sites web, des petites annonces où le statut sérologique est systématiquement affiché ou annoncé ?
UNE SEULE REPONSE POSSIBLE
☐ 1 Jamais
☐ 2 Presque jamais
☐ 3 Parfois
☐ 4 Presque toujours
☐ 5 Toujours
☐ 97 Non concerné (ne pas suggérer)
☐ 98 Ne sait pas (ne pas suggérer)
☐ 99 Refus de réponse (ne pas suggérer)

Parmi ces partenaires sexuels masculins, nous allons en distinguer deux types :
- **a) Les partenaires sexuels réguliers : (amants, copains de baise, fuck buddy, plan cul régulier)**
- **b) Les partenaires occasionnels : (rapports anonymes, coups d'un soir) avec qui vous n'avez eu qu'un seul rapport sexuel**

A.3.a) HSH : Partenaires sexuels réguliers

F27. Au cours des 12 derniers mois, combien avez-vous eu de partenaires sexuels réguliers [des copains de baise, fuck buddy, plan cul régulier etc.] **?**
/__ //__ //__ /__/ [si vous en avez eu beaucoup sans les avoir comptés, donnez un nombre approximatif]
➔ Si =0 → F28 ; Sinon → F27.b)
Refus de répondre (ne pas suggérer) **➔F28**
NSP (ne pas suggérer) **➔F28**

SI F27>0 ET F27<997 poser F27b)
F27.b) Pour vous, utiliser un préservatif pour la pénétration anale avec un partenaire sexuel régulier est : [De manière générale]
☐ 1 Très difficile
☐ 2 Plutôt difficile
☐ 3 Plutôt facile
☐ 4 Très facile
☐ 97 Non concerné (ne pas suggérer)
☐ 98 Ne sait pas (ne pas suggérer)
☐ 99 Refus de réponse (ne pas suggérer)

A.3.b) HSH : Partenaires occasionnels

F28. <u>Au cours des 12 derniers mois</u>, combien avez-vous eu de <u>partenaires sexuels occasionnels</u> [rapport anonyme, coup d'un soir**]** ?
/__ //__//__//__/ **[**si vous en avez eu beaucoup sans les avoir comptés, donnez un nombre approximatif**]**
➔ **Si =0 → F29 ; Sinon → F28.b)**
Refus de répondre (ne pas suggérer) ➔ F29
NSP (ne pas suggérer) ➔ F29

SI F28>0 ET F27<997 poser F28b)
F28.b) Pour vous, utiliser un préservatif pour la pénétration anale avec un <u>partenaire sexuel occasionnel</u> est : **[**De manière générale**]**
- ☐ 1 Très difficile
- ☐ 2 Plutôt difficile
- ☐ 3 Plutôt facile
- ☐ 4 Très facile
- ☐ 97 Non concerné (ne pas suggérer)
- ☐ 98 Ne sait pas (ne pas suggérer)
- ☐ 99 Refus de réponse (ne pas suggérer)

Si F27= {0, non concerné, refus, NSP} <u>et</u> F28={0, non concerné ,refus, NSP} ➔ Filtre début de la partie « D »

F29. <u>Au cours des 4 dernières semaines</u>, combien de relations sexuelles avez-vous eu avec ce/ces partenaire(s) [sexuel régulier et/ou occasionnel**]?**
/__ //__ /__/ /__/➔ **F30**

F29Bis. Si NSP en F29 poser les items
- ☐ 1 Aucun ➔ **F30**
- ☐ 2 1 à 4 rapports sexuels (1 fois par semaine ou moins) ➔ **F30**
- ☐ 3 5 à 8 rapports sexuels (entre 1 et 2 fois par semaine) ➔ **F30**
- ☐ 4 9 à 12 rapports sexuels (entre 2 et 3 fois par semaine) ➔ **F30**
- ☐ 5 13 à 16 rapports sexuels (entre 3 et 4 fois par semaine) ➔ **F30**
- ☐ 6 Plus de 16 rapports sexuels (plus de 4 fois par semaine) ➔ **F30**
- ☐ 97 Non concerné (ne pas suggérer) ➔ **F30**
- ☐ 98 Ne sait pas (ne pas suggérer) ➔ **F30**
- ☐ 99 Refus de réponse (ne pas suggérer) ➔ **F30**

Nous allons maintenant parler du <u>dernier rapport sexuel que vous avez eu</u> avec l'un de ces partenaires.

F30. Ce dernier rapport était avec :
UNE SEULE REPONSE POSSIBLE
- ☐ 1 Un partenaire sexuel régulier ➔ **Uniquement la partie** (i) **F31 à F37.d)bis**
- ☐ 2 Un partenaire occasionnel ➔ **Uniquement la partie** (ii) **F38 à F44.d)bis**
- ☐ 3 Plusieurs partenaires en même temps (plan à trois, sex parties etc.) ➔ **Uniquement la partie** (iii) **F45 à F49.d)bis**
- ☐ 97. Non concerné (ne pas suggérer) ➔ **Filtre début « D »**
- ☐ 98. Ne sait pas (ne pas suggérer) ➔ **Filtre début « D »**
- ☐ 99. Refus de réponse (ne pas suggérer) ➔ **Filtre début « D »**

A.3 (i) - HSH : Dernier rapport avec un partenaire sexuel régulier F30=1

F31. Ce partenaire sexuel est-il circoncis ?
- ☐ 1 Oui
- ☐ 2 Non
- ☐ 97. Non concerné (ne pas suggérer)
- ☐ 98. Ne sait pas (ne pas suggérer)
- ☐ 99. Refus de réponse (ne pas suggérer)

F33.a) Est-il séropositif?
- ☐ 1 Oui
- ☐ 2 Non
- ☐ 97. Non concerné (ne pas suggérer)
- ☐ 98. Ne sait pas (ne pas suggérer)
- ☐ 99. Refus de réponse (ne pas suggérer)

F33.b) Et lui, sait-il que vous êtes séropositif ?
- ☐ 1 Oui, vous lui avez dit de vous-même
- ☐ 2 Oui car il vous l'avait demandé
- ☐ 3 Non
- ☐ 4 Vous n'avez pas eu à le faire, il connaissait déjà votre statut
- ☐ 97. Non concerné (ne pas suggérer)
- ☐ 98. Ne sait pas (ne pas suggérer)
- ☐ 99. Refus de réponse (ne pas suggérer)

Nous allons maintenant parler plus précisément du <u>dernier rapport sexuel</u> avec ce partenaire :

F35. Où s'est déroulé ce dernier rapport sexuel avec ce partenaire sexuel régulier ?
- ☐ 1 Chez vous
- ☐ 2 Chez votre partenaire
- ☐ 3 Dans un autre lieu privé (chez quelqu'un, à l'hôtel par exemple)
- ☐ 4 Dans un sauna, une backroom, un vidéoclub
- ☐ 5 Dans un lieu extérieur de drague homo (parc, parking etc.)
- ☐ 6 Dans un lieu public
- ☐ 7 Autre, Précisez (ne pas suggérer)
- ☐ 97. Non concerné (ne pas suggérer)
- ☐ 98. Ne sait pas (ne pas suggérer)
- ☐ 99. Refus de réponse (ne pas suggérer)

F36. Etiez-vous sous l'effet d'un (ou des) produit(s) suivant(s) lors ce dernier rapport sexuel ?
☐ 1 Oui
☐ 2 Non
☐ 97. Non concerné (ne pas suggérer)
☐ 98. Ne sait pas (ne pas suggérer)
☐ 99. Refus de réponse (ne pas suggérer)

a) Cannabis,
b) Ecstasy
c) Cocaïne
d) Poppers
e) Alcool
f) Viagra®, et autres stimulants
g) Autres (Précisez)

F37. Lors de ce dernier rapport sexuel avec ce partenaire sexuel régulier, avez-vous utilisé le préservatif :

F37.a) En vous faisant sucer
☐ 1 Oui
☐ 2 Non
☐ 3 Il ne vous a pas sucé lors de ce dernier rapport
☐ 98. Ne sait pas (ne pas suggérer)
☐ 99. Refus de réponse (ne pas suggérer)

F37.a) bis (Si F37.a)=2) Avez-vous éjaculé dans sa bouche ?
☐ 1 Oui
☐ 2 Non
☐ 97. Non concerné (ne pas suggérer)
☐ 98. Ne sait pas (ne pas suggérer)
☐ 99. Refus de réponse (ne pas suggérer)

F37.b) En le suçant ?
☐ 1 Oui
☐ 2 Non
☐ 3 Il ne vous a pas sucé lors de ce dernier rapport
☐ 98. Ne sait pas (ne pas suggérer)
☐ 99. Refus de réponse (ne pas suggérer)

F37.b) bis (Si F37.b)=2) A-t-il éjaculé dans votre bouche ?
☐ 1 Oui
☐ 2 Non
☐ 97. Non concerné (ne pas suggérer)
☐ 98. Ne sait pas (ne pas suggérer)
☐ 99. Refus de réponse (ne pas suggérer)

F37.c) Quand vous l'avez sodomisé ?
☐ 1 Oui
☐ 2 Non
☐ 3 Il ne vous a pas sodomisé lors de ce dernier rapport
☐ 98. Ne sait pas (ne pas suggérer)
☐ 99. Refus de réponse (ne pas suggérer)

F37.c) bis (Si F37.c)=2) Vous êtes-vous retiré avant d'éjaculer ?
☐ 1 Oui
☐ 2 Non
☐ 97. Non concerné (ne pas suggérer)
☐ 98. Ne sait pas (ne pas suggérer)
☐ 99. Refus de réponse (ne pas suggérer)

F37.d) Avez-vous utilisé le préservatif **Quand il vous a sodomisé?**
☐ 1 Oui ➔ **Filtre début « D »**
☐ 2 Non ➔ **F37.d) bis**
☐ 3 Il ne vous a pas sodomisé lors de ce dernier rapport ➔ **Filtre début « D »**
☐ 98. Ne sait pas (ne pas suggérer) ➔ **Filtre début « D »**
☐ 99. Refus de réponse (ne pas suggérer) ➔ **Filtre début « D »**

F37.d)bis (Si F37.d)=2)Votre partenaire s'est-il retiré avant d'éjaculer [dans votre anus] **?**
☐ 1 Oui ➔ **Filtre début « D »**
☐ 2 Non ➔ **Filtre début « D »**
☐ 97. Non concerné (ne pas suggérer) ➔ **Filtre début « D »**
☐ 98. Ne sait pas (ne pas suggérer) ➔ **Filtre début « D »**
☐ 99. Refus de réponse (ne pas suggérer) ➔ **Filtre début « D »**

F37bis Toujours lors de ce dernier rapport avec ce partenaire sexuel régulier, avez-vous pratiqué le « fist-fucking »?
UNE SEULE REPONSE POSSIBLE
☐ 1 Oui
☐ 2 Non
☐ 97. Non concerné (ne pas suggérer)
☐ 98. Ne sait pas (ne pas suggérer)
☐ 99. Refus de réponse (ne pas suggérer)

A.3 (ii) - HSH : dernier rapport avec partenaire sexuel occasionnel F30=2

F38. Ce partenaire sexuel était-il circoncis ?
☐ 1 Oui
☐ 2 Non
☐ 97. Non concerné (ne pas suggérer)
☐ 98. Ne sait pas (ne pas suggérer)
☐ 99. Refus de réponse (ne pas suggérer)

F39. Avant d'avoir ce rapport sexuel avec ce partenaire occasionnel, avez-vous discuté de l'utilisation du préservatif avec lui ?
☐ 1 Oui
☐ 2 Non
☐ 97. Non concerné (ne pas suggérer)
☐ 98. Ne sait pas (ne pas suggérer)
☐ 99. Refus de réponse (ne pas suggérer)

F40. Toujours avant d'avoir un rapport avec ce partenaire occasionnel, connaissiez-vous son statut sérologique ?
☐ 1 Oui
☐ 2 Non
☐ 97. Non concerné (ne pas suggérer)
☐ 98. Ne sait pas (ne pas suggérer)
☐ 99. Refus de réponse (ne pas suggérer)

F40.a) (Si F40=1) Il était :
☐ 1Séropositif
☐ 2Séronégatif
☐ 3Il ne connaissait pas son statut
☐ 97. Non concerné (ne pas suggérer)
☐ 98. Ne sait pas (ne pas suggérer)
☐ 99. Refus de réponse (ne pas suggérer)

F41. Toujours avant d'avoir un rapport avec ce partenaire occasionnel, lui avez-vous révélé votre séropositivité ?
☐ 1Oui, vous lui avez dit de vous-même
☐ 2Oui car il vous l'avait demandé
☐ 3Non
☐ 4Vous n'avez pas eu à le faire, il connaissait déjà votre statut
☐ 97. Non concerné (ne pas suggérer)
☐ 98. Ne sait pas (ne pas suggérer)
☐ 99. Refus de réponse (ne pas suggérer)

Nous allons maintenant parler plus précisément du dernier rapport sexuel avec ce partenaire :

F42. Où s'est déroulé ce dernier rapport avec ce partenaire occasionnel ?
☐ 1 Chez vous
☐ 2 Chez votre partenaire
☐ 3 Dans un autre lieu privé (chez quelqu'un, à l'hôtel par exemple)
☐ 4 Dans un sauna, une backroom, un vidéoclub
☐ 5 Dans un lieu extérieur de drague homo (parc, parking etc.)
☐ 6 Dans un lieu public
☐ 7 Autre, Précisez (ne pas suggérer)
☐ 97. Non concerné (ne pas suggérer)
☐ 98. Ne sait pas (ne pas suggérer)
☐ 99. Refus de réponse (ne pas suggérer)

F43. Etiez-vous sous l'effet d'un (ou des) produit(s) suivant(s) lors ce dernier rapport sexuel ?
à monter sous forme de sous-questions
☐ 1 Oui
☐ 2 Non
☐ 97. Non concerné (ne pas suggérer)
☐ 98. Ne sait pas (ne pas suggérer)
☐ 99. Refus de réponse (ne pas suggérer)

a) Cannabis,
b) Ecstasy
c) Cocaïne
d) Poppers
e) Alcool
f) Viagra®, et autres stimulants
g) Autres (Précisez)

F44. Lors de ce dernier rapport sexuel avec ce partenaire occasionnel, avez-vous utilisé le préservatif :

F44.a) En vous faisant sucer
☐ 1 Oui
☐ 2 Non
☐ 3 Il ne vous a pas sucé lors de ce dernier rapport
☐ 98. Ne sait pas (ne pas suggérer)
☐ 99. Refus de réponse (ne pas suggérer)

F44.a) bis (Si F44.a)=2) Avez-vous éjaculé dans sa bouche ?
☐ 1 Oui
☐ 2 Non
☐ 97. Non concerné (ne pas suggérer)
☐ 98. Ne sait pas (ne pas suggérer)
☐ 99. Refus de réponse (ne pas suggérer)

F44.b) avez-vous utilisé le préservatif **en le suçant ?**
☐ 1Oui
☐ 2Non
☐ 3Vous ne l'avez pas sucé lors de ce dernier rapport
☐ 98. Ne sait pas (ne pas suggérer)
☐ 99. Refus de réponse (ne pas suggérer)

F44.b) bis (Si F44.b)=2) A-t-il éjaculé dans votre bouche ?
☐ 1 Oui
☐ 2 Non
☐ 97. Non concerné (ne pas suggérer)
☐ 98. Ne sait pas (ne pas suggérer)
☐ 99. Refus de réponse (ne pas suggérer)

F44.c) Avez-vous utilisé le préservatif **quand vous l'avez sodomisé?**
☐ 1 Oui
☐ 2 Non
☐ 3 Vous ne l'avez pas sodomisé lors de ce dernier rapport
☐ 98. Ne sait pas (ne pas suggérer)
☐ 99. Refus de réponse (ne pas suggérer)

F44.c) bis (Si F44.c)=2) Vous êtes-vous retiré avant d'éjaculer [dans son anus**] ?**
☐ 1 Oui
☐ 2 Non
☐ 97. Non concerné (ne pas suggérer)
☐ 98. Ne sait pas (ne pas suggérer)
☐ 99. Refus de réponse (ne pas suggérer)

F44.d) Avez-vous utilisé le préservatif **Quand il vous a sodomisé?**
☐ 1 Oui **➔ Filtre début « D »**
☐ 2 Non **➔ F44.d) bis**
☐ 3 Vous ne l'avez pas sodomisé lors de ce dernier rapport **➔ Filtre début « D »**
☐ 98. Ne sait pas (ne pas suggérer) **➔ Filtre début « D »**
☐ 99. Refus de réponse (ne pas suggérer) **➔ Filtre début « D »**

F44.d) bis Votre partenaire s'est-il retiré avant d'éjaculer [dans votre anus**] ?**
☐ 1 Oui **➔ Filtre début « D »**
☐ 2 Non **➔ Filtre début « D »**
☐ 97. Non concerné (ne pas suggérer) **➔ Filtre début « D »**
☐ 98. Ne sait pas (ne pas suggérer) **➔ Filtre début « D »**
☐ 99. Refus de réponse (ne pas suggérer) **➔ Filtre début « D »**

F44 bis) Toujours lors de ce dernier rapport avec ce partenaire occasionnel, avez-vous pratiqué le « fist-fucking » ?
UNE SEULE REPONSE POSSIBLE
☐ 1 Oui
☐ 2 Non
☐ 97. Non concerné (ne pas suggérer)
☐ 98. Ne sait pas (ne pas suggérer)
☐ 99. Refus de réponse (ne pas suggérer)

A.3 (iii) - HSH : plan à plusieurs F30=3

F45. Combien étiez-vous de partenaires sexuels au total (vous compris)?
/___//___//___/

F46. Où s'est déroulée cette sex party ou plan à plusieurs?
☐ 1 Chez vous
☐ 2 Chez votre partenaire
☐ 3 Dans un autre lieu privé (chez quelqu'un, à l'hôtel par exemple)
☐ 4 Dans un sauna, une backroom, un vidéoclub
☐ 5 Dans un lieu extérieur de drague homo (parc, parking etc.)
☐ 6 Dans un lieu public
☐ 7 Autre, Précisez (ne pas suggérer)
☐ 97. Non concerné (ne pas suggérer)
☐ 98. Ne sait pas (ne pas suggérer)
☐ 99. Refus de réponse (ne pas suggérer)

F47. Etiez-vous sous l'effet d'un (ou des) produits suivants lors de cette sex party ou plan à plusieurs ?
☐ 1 Oui
☐ 2 Non
☐ 97. Non concerné (ne pas suggérer)
☐ 98. Ne sait pas (ne pas suggérer)
☐ 99. Refus de réponse (ne pas suggérer)

a) Cannabis,
b) Ecstasy
c) Cocaïne
d) Poppers
e) Alcool
f) Viagra®, et autres stimulants
g) Autres (Précisez)

F48. Identifiez chacun de vos partenaires par une lettre :
…
…
… } Chaque lettre devra être mise à la place des « … »
…
Attention : bien limiter les itérations à 4

Nous allons maintenant nous intéresser au partenaire « … » : reprise et affichage de la 1ère lettre

F49. Avec « … », avez-vous utilisé le préservatif :

F49.a) En vous faisant sucer
☐ 1 Oui
☐ 2 Non
☐ 3 Vous ne l'avez pas sucé lors de ce dernier rapport
☐ 98. Ne sait pas (ne pas suggérer)
☐ 99. Refus de réponse (ne pas suggérer)

F49.a) bis (Si F49.a)=2) Avez-vous éjaculé dans sa bouche ?
☐ 1 Oui
☐ 2 Non
☐ 97. Non concerné (ne pas suggérer)
☐ 98. Ne sait pas (ne pas suggérer)
☐ 99. Refus de réponse (ne pas suggérer)

F49.b) [Toujours avec « … », avez-vous utilisé le préservatif**] En le suçant ?**
☐ 1 Oui
☐ 2 Non
☐ 97. Non concerné (ne pas suggérer)
☐ 98. Ne sait pas (ne pas suggérer)
☐ 99. Refus de réponse (ne pas suggérer)

F49.b)bis (Si F49.b)=2) A-t-il éjaculé dans votre bouche ?
☐ 1 Oui
☐ 2 Non
☐ 3 Vous ne l'avez pas sucé lors de ce dernier rapport
☐ 98. Ne sait pas (ne pas suggérer)
☐ 99. Refus de réponse (ne pas suggérer)

F49.c) [Toujours avec « … »], avez-vous utilisé le préservatif **quand vous l'avez sodomisé** [pénétré dans l'anus] **?**
☐ 1 Oui
☐ 2 Non
☐ 3Vous ne l'avez pas sodomisé lors de ce dernier rapport
☐ 98. Ne sait pas (ne pas suggérer)
☐ 99. Refus de réponse (ne pas suggérer)

F49.c) bis (Si F49.c)=2) Vous êtes-vous retiré avant d'éjaculer [dans son anus] **?**
☐ 1 Oui
☐ 2 Non
☐ 97. Non concerné (ne pas suggérer)
☐ 98. Ne sait pas (ne pas suggérer)
☐ 99. Refus de réponse (ne pas suggérer)

F49.d) Quand il vous a sodomisé ?
☐ 1 Oui
☐ 2 Non
☐ 3 Il ne vous a pas sodomisé lors de ce dernier rapport
☐ 97. Non concerné (ne pas suggérer)
☐ 98. Ne sait pas (ne pas suggérer)
☐ 99. Refus de réponse (ne pas suggérer)

F49.d) bis (Si F49.d)=2) Votre partenaire s'est-il retiré avant d'éjaculer [dans votre anus] **?**
☐ 1 Oui
☐ 2 Non
☐ 97. Non concerné (ne pas suggérer)
☐ 98. Ne sait pas (ne pas suggérer)
☐ 99. Refus de réponse (ne pas suggérer)

F49 bis) Toujours lors de ce dernier rapport, et avec « … », avez-vous pratiqué le « fist-fucking »
UNE SEULE REPONSE POSSIBLE
☐ 1 Oui
☐ 2 Non
☐ 97. Non concerné (ne pas suggérer)
☐ 98. Ne sait pas (ne pas suggérer)
☐ 99. Refus de réponse (ne pas suggérer)

➔ Itération des questions F49 jusqu'à avoir passé tous les partenaires – 4 MAX -puis➔ Filtre début « D – module FSF »

B - MODULE FSF
Femmes ou Male to Female [MtoF] ayant eu au moins une partenaire féminine dans l'année

Si [A.1=2 Ou F1a=2)] Et F2.b ≠ {1, 10, 11}
SINON : filtre début « C »

Nous allons maintenant parler de vos relations sexuelles avec une ou des femmes.

F50. Au cours des 12 derniers mois, vous avez eu des relations sexuelles
PLUSIEURS REPONSES POSSIBLES
- ☐ 1Avec une femme qui est votre partenaire principale
- ☐ 2Avec une femme qui est une partenaire régulière
- ☐ 3Avec une/plusieurs partenaires occasionnelles
- ☐ 97. Non concerné (ne pas suggérer)
- ☐ 98. Ne sait pas (ne pas suggérer)
- ☐ 99. Refus de réponse (ne pas suggérer)

F51. Au cours des 12 derniers mois, avez-vous caressé le sexe d'une femme avec la bouche (ou cunnilingus) ?
- ☐ 1Souvent
- ☐ 2Parfois
- ☐ 3Rarement
- ☐ 4Jamais
- ☐ 97. Non concerné (ne pas suggérer)
- ☐ 98. Ne sait pas (ne pas suggérer)
- ☐ 99. Refus de réponse (ne pas suggérer)

F51.a) (Si F51={1,2,3}) Avez-vous utilisé une digue dentaire ou un carré de latex lors de cette pratique ?
- ☐ 1Souvent
- ☐ 2Parfois
- ☐ 3Rarement
- ☐ 4Jamais
- ☐ 97. Non concerné (ne pas suggérer)
- ☐ 98. Ne sait pas (ne pas suggérer)
- ☐ 99. Refus de réponse (ne pas suggérer)

F52. Au cours des douze derniers mois, une femme vous a-t-elle caressé le sexe avec la bouche (ou cunnilingus) ?
- ☐ 1Souvent
- ☐ 2Parfois
- ☐ 3Rarement
- ☐ 4Jamais
- ☐ 97. Non concerné (ne pas suggérer)
- ☐ 98. Ne sait pas (ne pas suggérer)
- ☐ 99. Refus de réponse (ne pas suggérer)

F52.a) (Si F52={1,2,3}) Avez-vous utilisé une digue dentaire ou un carré de latex lors de cette pratique ?
- ☐ 1Souvent
- ☐ 2Parfois
- ☐ 3Rarement
- ☐ 4Jamais
- ☐ 97. Non concerné (ne pas suggérer)
- ☐ 98. Ne sait pas (ne pas suggérer)
- ☐ 99. Refus de réponse (ne pas suggérer)

F53. Au cours des douze derniers mois, avez-vous pénétré le vagin ou l'anus d'une femme avec un objet, un godemiché ?
- ☐ 1Souvent
- ☐ 2Parfois
- ☐ 3Rarement
- ☐ 4Jamais
- ☐ 97. Non concerné (ne pas suggérer)
- ☐ 98. Ne sait pas (ne pas suggérer)
- ☐ 99. Refus de réponse (ne pas suggérer)

F54. Au cours des douze derniers mois, une femme vous a-t-elle pénétré le vagin ou l'anus avec un objet, un godemiché?
- ☐ 1Souvent
- ☐ 2Parfois
- ☐ 3Rarement
- ☐ 4Jamais
- ☐ 97. Non concerné (ne pas suggérer)
- ☐ 98. Ne sait pas (ne pas suggérer)
- ☐ 99. Refus de réponse (ne pas suggérer)

F55. (Sauf si : F53={4,5,6} Et F54={4,5,6}) Au cours des douze derniers mois, avez-vous pris des précautions particulières au cours de ces rapports (utilisation de préservatifs, désinfection des objets échangés etc.) lors des rapports avec pénétration ?
- ☐ 1Souvent ➔ filtre début « C – module hétéro femme »
- ☐ 2Parfois ➔ filtre début « C – module hétéro femme »
- ☐ 3Rarement ➔ filtre début « C – module hétéro femme »
- ☐ 4Jamais ➔ filtre début « C – module hétéro femme »
- ☐ 97. Non concerné (ne pas suggérer) ➔ filtre début « C – module hétéro femme »
- ☐ 98. Ne sait pas (ne pas suggérer) ➔ filtre début « C – module hétéro femme »
- ☐ 99. Refus de réponse (ne pas suggérer) ➔ filtre début « C – module hétéro femme »

C - MODULE HETEROSEXUEL (REPONDANT FEMME OU MALE TO FEMALE [MTOF]) Au moins un partenaire de sexe opposé dans l'année

C.1 - Hétéro (Femme/MtoF) : Partenaire principal

(Si (A.1=2 ou F1a=2)) et F8=1)
Sinon, aller au filtre début C2 (femme, hétéro, pas de partenaire principal)

Nous allons maintenant parler de façon un peu plus précise de vos relations avec votre partenaire principal masculin:

F56. (sauf si F5≠1 ou si F8.e≠1) Au cours des 4 dernières semaines Combien avez-vous eu des relations sexuelles avec votre partenaire principal masculin ?

|____| mettre d'abord un champ à remplir par l'enquêteur

- ☐ 1 Aucun
- ☐ 2 1 à 4 rapports sexuels (1 fois par semaine ou moins)
- ☐ 3 5 à 8 rapports sexuels (entre 1 et 2 fois par semaine)
- ☐ 4 9 à 12 rapports sexuels (entre 2 et 3 fois par semaine)
- ☐ 5 13 à 16 rapports sexuels (entre 3 et 4 fois par semaine)
- ☐ 6 Plus de 16 rapports sexuels (plus de 4 fois par semaine)
- ☐ 97 Non concerné (ne pas suggérer)
- ☐ 98 Ne sait pas (ne pas suggérer)
- ☐ 99 Refus de réponse (ne pas suggérer)

F57. Votre partenaire est-il circoncis ?

- ☐ 1 Oui
- ☐ 2 Non
- ☐ 97. Non concerné (ne pas suggérer)
- ☐ 98. Ne sait pas (ne pas suggérer)
- ☐ 99. Refus de réponse (ne pas suggérer)

Nous allons maintenant remonter au début de cette relation avec votre partenaire principal masculin :

Poser F58 si Année F8.a > 1985

F58. Au début de votre relation, vous étiez :

- ☐ 1 Séronégative
- ☐ 2 Séropositive
- ☐ 3 Vous ne connaissiez pas votre statut sérologique
- ☐ 97. Non concerné (ne pas suggérer)
- ☐ 98. Ne sait pas (ne pas suggérer)
- ☐ 99. Refus de réponse (ne pas suggérer)

F59. Lors de votre premier rapport sexuel avec ce partenaire, avez-vous utilisé un préservatif ?

- ☐ 1 Oui
- ☐ 2 Non
- ☐ 97. Non concerné (ne pas suggérer)
- ☐ 98. Ne sait pas (ne pas suggérer)
- ☐ 99. Refus de réponse (ne pas suggérer)

Si F58=2

F60. Comment avez-vous annoncé votre séropositivité à votre partenaire ?

- ☐ 1 Vous lui avez dit de vous-même
- ☐ 2 Vous lui avez dit car il vous l'avait demandé
- ☐ 3 Il savait que vous étiez séropositif sans avoir à en parler ex : vous êtes rencontrés dans un espace réservé aux personnes séropositives (internet, soirées privées etc.)
- ☐ 4 Vous ne lui avez jamais dit
- ☐ 97. Non concerné (ne pas suggérer)
- ☐ 98. Ne sait pas (ne pas suggérer)
- ☐ 99. Refus de réponse (ne pas suggérer

Si (F58≠2 ou Année(F8.a) < 1985)

F60.a). Comment avez-vous annoncé votre séropositivité à votre partenaire ?

- ☐ 1 Vous lui avez dit de vous-même
- ☐ 2 Vous lui avez dit car il vous l'avait demandé
- ☐ 3 Il l'a su autrement
- ☐ 4 Vous ne lui avez jamais dit
- ☐ 97. Non concerné (ne pas suggérer)
- ☐ 98. Ne sait pas (ne pas suggérer)
- ☐ 99. Refus de réponse (ne pas suggérer)

F60.b) Si (F58≠2 ou Année(F8.a)<1985) ET F60.a=1 o 2 o 3 A quel moment ?

- ☐ 1 Immédiatement après avoir découvert votre séropositivité
- ☐ 2 Dans les trois mois qui ont suivi la découverte de votre séropositivité
- ☐ 3 Plus tard dans votre relation
- ☐ 97. Non concerné (ne pas suggérer)
- ☐ 98. Ne sait pas (ne pas suggérer)
- ☐ 99. Refus de réponse (ne pas suggérer

F60. c) Si (F58=2 et F60={1,2}) A quel moment ?

- ☐ 1 Avant votre premier rapport sexuel
- ☐ 2 Dans les trois premiers mois de votre relation
- ☐ 3 Plus tard dans votre relation
- ☐ 97. Non concerné (ne pas suggérer)
- ☐ 98. Ne sait pas (ne pas suggérer)
- ☐ 99. Refus de réponse (ne pas suggérer

Poser F61 si Année F8.a > 1985

F61. Toujours au début de votre relation, quel était le statut sérologique de votre partenaire ?

- ☐ 1 Séronégatif
- ☐ 2 Séropositif
- ☐ 3 Vous ne saviez pas
- ☐ 4 Il ne connaissait pas son statut
- ☐ 97. Non concerné (ne pas suggérer)
- ☐ 98. Ne sait pas (ne pas suggérer)
- ☐ 99. Refus de réponse (ne pas suggérer

F61.a) (Si F61≠2) Votre partenaire est-il séropositif aujourd'hui ?

- ☐ 1 Oui
- ☐ 2 Non
- ☐ 97. Non concerné (ne pas suggérer)
- ☐ 98. Ne sait pas (ne pas suggérer)
- ☐ 99. Refus de réponse (ne pas suggérer)

(SI F8.e=1 et date (F8a) - date(système/aujourd'hui) >12 mois, sinon aller au filtre au début de la partie C.3) Nous allons maintenant parler de vos rapports sexuels avec ce partenaire au cours des 12 derniers mois :

F62. Au cours des 12 derniers mois, vous avez pratiqué avec votre partenaire principal:

F62.a) La fellation

☐ 1 Jamais	➔ F62.b)
☐ 2 Rarement	➔ **F62.a) bis**
☐ 3 Parfois	➔ **F62.a) bis**
☐ 4 Souvent	➔ **F62.a) bis**
☐ 5 Toujours	➔ **F62.a) bis**
☐ 97 Non concerné (ne pas suggérer)	➔ F62.b)
☐ 98 Ne sait pas (ne pas suggérer)	➔ F62.b)
☐ 99 Refus de réponse (ne pas suggérer)	➔ F62.b)

F62.a) bis Pour celle-ci, utilisez-vous des préservatifs?

- ☐ 1 Jamais
- ☐ 2 Presque jamais
- ☐ 3 Parfois
- ☐ 4 Presque toujours
- ☐ 5 Toujours
- ☐ 97 Non concerné (ne pas suggérer)
- ☐ 98 Ne sait pas (ne pas suggérer)
- ☐ 99 Refus de réponse (ne pas suggérer)

F62.b) La pénétration vaginale ?

☐ 1 Jamais	➔ F62.c)
☐ 2 Rarement	➔ **F62.b) bis**
☐ 3 Parfois	➔ **F62.b) bis**
☐ 4 Souvent	➔ **F62.b) bis**
☐ 5 Toujours	➔ **F62.b) bis**
☐ 97 Non concerné (ne pas suggérer)	➔ F62.c)
☐ 98 Ne sait pas (ne pas suggérer)	➔ F62.c)
☐ 99 Refus de réponse (ne pas suggérer)	➔ F62.c)

F62.b) bis Et pour la pénétration vaginale, utilisez-vous des préservatifs ?

☐ 1 Jamais	➔ **F62.b) ter**
☐ 2 Presque jamais	➔ **F62.b) ter**
☐ 3 Parfois	➔ **F62.b) ter**
☐ 4 Presque toujours	➔ **F62.b) ter**
☐ 5 Toujours	➔ F62.c)
☐ 97 Non concerné (ne pas suggérer)	➔ F62.c)
☐ 98 Ne sait pas (ne pas suggérer)	➔ F62.c)
☐ 99 Refus de réponse (ne pas suggérer)	➔ F62.c)

F62.b) ter Votre partenaire se retire-t-il avant d'éjaculer ?

- ☐ 1 Jamais
- ☐ 2 Presque jamais
- ☐ 3 Parfois
- ☐ 4 Presque toujours
- ☐ 5 Toujours
- ☐ 97 Non concerné (ne pas suggérer)
- ☐ 98 Ne sait pas (ne pas suggérer)
- ☐ 99 Refus de réponse (ne pas suggérer)

F62.c) La pénétration anale ?

☐ 1 Jamais	➔ F63
☐ 2 Rarement	➔ **F62.c) bis**
☐ 3 Parfois	➔ **F62.c) bis**
☐ 4 Souvent	➔ **F62.c) bis**
☐ 5 Toujours	➔ **F62.c) bis**
☐ 97 Non concerné (ne pas suggérer)	➔ F63
☐ 98 Ne sait pas (ne pas suggérer)	➔ F63
☐ 99 Refus de réponse (ne pas suggérer)	➔ F63

F62.c) bis Et pour la pénétration anale, utilisez-vous des préservatifs ?

- ☐ 1 Jamais
- ☐ 2 Presque jamais
- ☐ 3 Parfois
- ☐ 4 Presque toujours
- ☐ 5 Toujours
- ☐ 97 Non concerné (ne pas suggérer)
- ☐ 98 Ne sait pas (ne pas suggérer)
- ☐ 99 Refus de réponse (ne pas suggérer)

F63. <u>Au cours des 12 derniers mois</u>, avez-vous utilisé des préservatifs féminins avec votre partenaire principal ?

- ☐ 1 Jamais
- ☐ 2 Presque jamais
- ☐ 3 Parfois
- ☐ 4 Presque toujours
- ☐ 5 Toujours
- ☐ 97 Non concerné (ne pas suggérer)
- ☐ 98 Ne sait pas (ne pas suggérer)
- ☐ 99 Refus de réponse (ne pas suggérer)

F64. Et lors de <u>votre dernier rapport sexuel</u> avec ce partenaire, avez-vous utilisé un préservatif lors de la pénétration ?

- ☐ 1 Oui
- ☐ 2 Non
- ☐ 97. Non concerné (ne pas suggérer)
- ☐ 98. Ne sait pas (ne pas suggérer)
- ☐ 99. Refus de réponse (ne pas suggérer)

F65. Pour vous, utiliser un préservatif pour la pénétration avec votre partenaire principal est : [De manière générale, lors de la pénétration anale et/ou vaginale**]**

- ☐ 1 Très difficile
- ☐ 2 Plutôt difficile
- ☐ 3 Plutôt facile
- ☐ 4 Très facile
- ☐ 97. Non concerné (ne pas suggérer)
- ☐ 98. Ne sait pas (ne pas suggérer)
- ☐ 99. Refus de réponse (ne pas suggérer)

F68. <u>au cours des 12 derniers mois</u>, avez-vous eu d'autres partenaires sexuels masculins que votre partenaire principal ?

- ☐ 1 Oui
- ☐ 2 Non
- ☐ 97. Non concerné (ne pas suggérer)
- ☐ 98. Ne sait pas (ne pas suggérer)
- ☐ 99. Refus de réponse (ne pas suggérer)

F68.a) (Si F68=1) Lui avez-vous dit ?

- ☐ 1 Oui
- ☐ 2 Non
- ☐ 97. Non concerné (ne pas suggérer)
- ☐ 98. Ne sait pas (ne pas suggérer)
- ☐ 99. Refus de réponse (ne pas suggérer)

F69. Depuis que vous le connaissez, est-il déjà arrivé que votre partenaire principal vous menace ou exerce une pression psychologique sur vous, pour avoir des relations sexuelles ?
[Il a, au travers de mots blessant, dénigrant, médisant, rabaissant etc., ou encore au travers de sous-entendus, porté atteinte à votre moral]

- ☐ 1 Oui c'est arrivé dans les 12 derniers mois
- ☐ 2 Oui mais c'est arrivé il y a plus de 12 derniers mois
- ☐ 3 Non
- ☐ 97. Non concerné (ne pas suggérer)
- ☐ 98. Ne sait pas (ne pas suggérer)
- ☐ 99. Refus de réponse (ne pas suggérer)

F70. Depuis que vous le connaissez, est-il déjà arrivé que votre partenaire principal utilise la force physique pour avoir des relations sexuelles avec vous, ou qu'il vous impose des gestes sexuels que vous refusiez ?

- ☐ 1 Oui c'est arrivé dans les 12 derniers mois
- ☐ 2 Oui mais c'est arrivé il y a plus de 12 derniers mois
- ☐ 3 Non
- ☐ 97. Non concerné (ne pas suggérer)
- ☐ 98. Ne sait pas (ne pas suggérer)
- ☐ 99. Refus de réponse (ne pas suggérer)

C.2 - Hétéro (Femme/MtoF) : Pas de partenaire principal au moment de la passation

Si A.1=2 ou F1a=2 et F8< >1

A vérifier on doit poser même si F8=3o4 (refus ou NSP)

<u>SINON</u> : Filtres avant Début partie C.3)

Vous m'avez dit que vous n'avez pas de partenaire principal masculin actuellement (ou vous n'avez pas répondu à cette question)

F71. Avez-vous déjà eu un partenaire stable, un conjoint ou un compagnon ?

- ☐ 1 Oui ➔ **F71.a)**
- ☐ 2 Non ➔ Filtres avant F72
- ☐ 97. Non concerné (ne pas suggérer) ➔ Filtres avant F72
- ☐ 98. Ne sait pas (ne pas suggérer) ➔ Filtres avant F72
- ☐ 99. Refus de réponse (ne pas suggérer) ➔ Filtres avant F72

Nous allons maintenant parler de cette relation ou de la dernière de ces relations avec un- partenaire stable

F71.a) En quelle année cette relation avait-elle commencé ?
_ _ _ _

F71.b) En quelle année cette relation s'est-elle terminée ?
_ _ _ _

F71.c) A ce moment là, connaissait-il votre séropositivité ?
- ☐ 1Oui ➔ **F71.d)**
- ☐ 2Non ➔ **F71.c) bis**
- ☐ 3Vous ne savez pas ➔ **F71.c) bis**
- ☐ 4Vous n'étiez pas encore séropositive ➔ **F71.d)**
- ☐ 97Non concerné (ne pas suggérer) ➔ **F71.d)**
- ☐ 99 Refus de réponse (ne pas suggérer) ➔ **F71.d)**

F71.c) bis Aviez-vous peur d'être rejetée en le lui disant ?
- ☐ 1 Oui
- ☐ 2 Non
- ☐ 97. Non concerné (ne pas suggérer)
- ☐ 98. Ne sait pas (ne pas suggérer)
- ☐ 99. Refus de réponse (ne pas suggérer)

F 71.d) Connaissiez vous son statut sérologique ?
- ☐ 1 Oui, il vous a dit qu'il était séropositif ➔ Filtres début C.3
- ☐ 2 Oui, vous pensez qu'il était séropositif ➔ Filtres début C.3
- ☐ 3 Oui, vous pensez qu'il était séronégatif ➔ Filtres début C.3
- ☐ 4 Oui, il vous a dit qu'il était séronégatif ➔ Filtres début C.3
- ☐ 5 Non, vous ne le saviez pas ➔ Filtres début C.3
- ☐ 97 Non concerné (ne pas suggérer) ➔ Filtres début C.3
- ☐ 98 Ne sait pas (ne pas suggérer) ➔ Filtres début C.3
- ☐ 99 Refus de réponse (ne pas suggérer) ➔ Filtres début C.3

C.3 - Hétéro (Femme/MtoF) : Autres partenaires sexuels

Si (A.1=2) ou (F1a=2) et

(Si F8=1 et [(F5=1 et F68=1) ou (F5=1 et F8.e ≠ 1)] ou (F5=1 et date(F8.a)-date (aujourd'hui) < 12 mois))
Nous allons maintenant parler de vos partenaires sexuels masculins en dehors de votre partenaire principal au cours des 12 derniers mois

Ou

(Si F8<>1 et F2.a ≠ {1, 10,11} et F5=1)
Nous allons maintenant parler de vos partenaires sexuels masculins de ces 12 derniers mois

➔ **Si aucun de ces filtres → Filtre début partie E**

F6abis Au cours des 12 derniers mois, combien de partenaires sexuels masculins avez-vous eu, en dehors de votre partenaire principal (si vous en avez un)?
/_____/ (→ sur 4 positions) ➔ Si =0, refus ou NSP : aller au filtre au début de la partie « E »

F72. Au cours des 4 dernières semaines, combien de rapports sexuels avez-vous eu avec ce ou ces partenaires occasionnels masculin ?

/____/ NE PAS OUBLIER DE METTRE LE CHAMP➔ Si =0, refus ou NSP ➔ **F77b**

- ☐ 1 Aucun ➔ **F77b**
- ☐ 21 à 4 rapports sexuels (1 fois par semaine ou moins) ➔ **F77b**
- ☐ 35 à 8 rapports sexuels (entre 1 et 2 fois par semaine) ➔ **F77b**
- ☐ 49 à 12 rapports sexuels (entre 2 et 3 fois par semaine) ➔ **F77b**
- ☐ 513 à 16 rapports sexuels (entre 3 et 4 fois par semaine) ➔ **F77b**
- ☐ 6Plus de 16 rapports sexuels (plus de 4 fois par semaine) ➔ **F77b**
- ☐ 97 Non concerné (ne pas suggérer) ➔ **F77b**
- ☐ 98 Ne sait pas (ne pas suggérer) ➔ **F77b**
- ☐ 99 Refus de réponse (ne pas suggérer) ➔ **F77b**

F77.b) Pour vous, utiliser un préservatif avec ce type de partenaire sexuel est :
- ☐ 1 Très difficile
- ☐ 2 Plutôt difficile
- ☐ 3 Plutôt facile
- ☐ 4 Très facile
- ☐ 97 Non concerné (ne pas suggérer)
- ☐ 98 Ne sait pas (ne pas suggérer)
- ☐ 99 Refus de réponse (ne pas suggérer)

(Si F6.a) bis=1) Nous allons maintenant nous intéresser à votre dernier rapport sexuel avec ce partenaire masculin

(Si F6.a) bis > 1) Nous allons maintenant nous intéresser à votre dernier rapport sexuel avec l'un de ces partenaires masculins

F73. Etait-ce :
- ☐ 1 Une relation d'un soir
- ☐ 2 Une relation régulière ou suivie
- ☐ 97 Non concerné (ne pas suggérer)
- ☐ 98 Ne sait pas (ne pas suggérer)
- ☐ 99 Refus de réponse (ne pas suggérer)

F74. Ce partenaire sexuel est-il circoncis ?
☐ 1 Oui
☐ 2 Non
☐ 97. Non concerné (ne pas suggérer)
☐ 98. Ne sait pas (ne pas suggérer)
☐ 99. Refus de réponse (ne pas suggérer)

F77. Quel était le statut sérologique de ce partenaire au moment de ce dernier rapport ?
☐ 1 Séronégatif
☐ 2 Séropositif
☐ 3 vous ne saviez pas
☐ 97. Non concerné (ne pas suggérer)
☐ 98. Ne sait pas (ne pas suggérer)
☐ 99. Refus de réponse (ne pas suggérer)

F77.b)bis Et lui, savait-il à ce moment là que vous étiez séropositive ?
☐ 1 Oui, vous lui avez dit de vous-même
☐ 2 Oui car il vous l'avait demandé
☐ 3 Non
☐ 4 Vous n'avez pas eu à le faire, il connaissait déjà votre statut
☐ 97. Non concerné (ne pas suggérer)
☐ 98. Ne sait pas (ne pas suggérer)
☐ 99. Refus de réponse (ne pas suggérer)

Nous allons maintenant parler plus précisément du dernier rapport sexuel avec ce partenaire :

F78. Lors de ce dernier rapport sexuel avec ce partenaire, avez-vous utilisé un préservatif :

F78.a) Pour la fellation ?
☐ 1Oui
☐ 2Non
☐ 3Vous n'avez pas pratiqué la fellation lors de ce dernier rapport
☐ 98. Ne sait pas (ne pas suggérer)
☐ 99. Refus de réponse (ne pas suggérer)

F78.b) Pour la pénétration vaginale ?
☐ 1Oui
☐ 2Non
☐ 3Vous n'avez pas pratiqué la pénétration vaginale lors de ce dernier rapport
☐ 98. Ne sait pas (ne pas suggérer)
☐ 99. Refus de réponse (ne pas suggérer)

F78.c) Pour la pénétration anale ?
☐ 1Oui
☐ 2Non
☐ 3Vous n'avez pas pratiqué la pénétration anale lors de ce dernier rapport
☐ 98. Ne sait pas (ne pas suggérer)
☐ 99. Refus de réponse (ne pas suggérer)

F78.d) (Si F78.b =2 ET/OU F78.c=2) Votre partenaire s'est-il retiré avant d'éjaculer [lors de la pénétration anale et/ou vaginale**] ?**
☐ 1 Oui
☐ 2 Non
☐ 97. Non concerné (ne pas suggérer)
☐ 98. Ne sait pas (ne pas suggérer)
☐ 99. Refus de réponse (ne pas suggérer)

F79. Est-ce que cette relation dure encore ?
☐ 1Oui
☐ 2Non elle a duré quelques mois
☐ 3Non elle a duré quelques semaines
☐ 4Non elle n'a duré que quelques jours
☐ 5Non c'était un seul rapport sexuel
☐ 6Vous ne savez pas comment elle va se passer
☐ 97. Non concerné (ne pas suggérer)
☐ 98. Ne sait pas (ne pas suggérer)
☐ 99. Refus de réponse (ne pas suggérer)

F80. Au cours de ces 12 derniers mois, avez-vous cherché des partenaires sexuels masculins sur internet?
☐ 1 Jamais
☐ 2moins d'une fois par semaine
☐ 3une fois par semaine
☐ 4plus d'une fois par semaine
☐ 5tous les jours ou presque
☐ 6Vous n'utilisez pas internet
☐ 97. Non concerné (ne pas suggérer)
☐ 98. Ne sait pas (ne pas suggérer)
☐ 99. Refus de réponse (ne pas suggérer)

F81. Et pour rencontrer des partenaires masculins avez-vous utilisé des sites web, des petites annonces où le statut sérologique est systématiquement affiché au cours des 12 derniers mois?
☐ 1Jamais
☐ 2Presque jamais
☐ 3Parfois
☐ 4Presque toujours
☐ 5Toujours
☐ 97. Non concerné (ne pas suggérer)
☐ 98. Ne sait pas (ne pas suggérer)
☐ 99. Refus de réponse (ne pas suggérer)

D - MODULE HETEROSEXUEL (REPONDANT HOMME OU FEMALE TO MALE [FTOM])
Au moins un partenaire de sexe opposé dans l'année

(Si (A.1=1 ou F1a=1)) et (F2.b≠ 1 ou 10 ou 11) et (F9=1))
Sinon : filtre avant la question F96 (homme, hétéro, pas de partenaire principal) Début partie D.2

D.1 - Hétéro (Homme/FtoM) : Partenaire principale

Nous allons maintenant parler de façon un peu plus précise de vos relations avec votre partenaire principale féminine :

F82. (Sauf si F9.e≠1 et F5≠1) Au cours des 4 dernières semaines, Combien avez-vous eu de relations sexuelles avec votre partenaire principale féminine ?

|____| mettre d'abord un champ à remplir par l'enquêteur

☐ 1 Aucun
☐ 2 1 à 4 rapports sexuels (1 fois par semaine ou moins)
☐ 3 5 à 8 rapports sexuels (entre 1 et 2 fois par semaine)
☐ 4 9 à 12 rapports sexuels (entre 2 et 3 fois par semaine)
☐ 5 13 à 16 rapports sexuels (entre 3 et 4 fois par semaine)
☐ 6 Plus de 16 rapports sexuels (plus de 4 fois par semaine)
☐ 97 Non concerné (ne pas suggérer)
☐ 98 Ne sait pas (ne pas suggérer)
☐ 99 Refus de réponse (ne pas suggérer)

Nous allons maintenant remonter au début de cette relation avec votre partenaire principale féminine :

Poser F83 si Année(F9.a) > 1985
F83. Au début de votre relation, vous étiez :
☐ 1 Séronégatif
☐ 2 Séropositif
☐ 3 Vous ne connaissiez pas votre statut sérologique
☐ 97 Non concerné (ne pas suggérer)
☐ 98 Ne sait pas (ne pas suggérer)
☐ 99 Refus de réponse (ne pas suggérer)

F84. Lors de votre premier rapport sexuel avec cette partenaire, avez-vous utilisé un préservatif ?
☐ 1 Oui
☐ 2 Non
☐ 97. Non concerné (ne pas suggérer)
☐ 98. Ne sait pas (ne pas suggérer)
☐ 99. Refus de réponse (ne pas suggérer)

Si F83=2
F85. Comment avez-vous annoncé votre séropositivité à votre partenaire principale féminine ?
☐ 1 Vous lui avez dit de vous-même
☐ 2 Vous lui avez dit car elle vous l'avait demandé
☐ 3 Elle savait que vous étiez séropositif sans avoir à en parler (ex : vous êtes rencontrés dans un espace réservé aux personnes séropositives (internet, soirées privées etc.))
☐ 4 Vous ne lui avez jamais dit
☐ 97. Non concerné (ne pas suggérer)
☐ 98. Ne sait pas (ne pas suggérer)
☐ 99. Refus de réponse (ne pas suggérer)

Si (F83≠2 ou Année(F9.a) < 1985)
F85.a). Comment avez-vous annoncé votre séropositivité à votre partenaire principale féminine ?
☐ 1 Vous lui avez dit de vous-même
☐ 2 Vous lui avez dit car elle vous l'avait demandé
☐ 3 Elle l'a su autrement
☐ 4 Vous ne lui avez jamais dit
☐ 97. Non concerné (ne pas suggérer)
☐ 98. Ne sait pas (ne pas suggérer)
☐ 99. Refus de réponse (ne pas suggérer)

F85.b) Si (F83≠2 ou Année(F9.a) < 1985) Et F85.a=1o2o3 A quel moment ?
☐ 1 Immédiatement après avoir découvert votre séropositivité
☐ 2 Dans les trois mois qui ont suivi la découverte de votre séropositivité
☐ 3 Plus tard dans votre relation
☐ 97. Non concerné (ne pas suggérer)
☐ 98. Ne sait pas (ne pas suggérer)
☐ 99. Refus de réponse (ne pas suggérer)

F85. c) Si (F83=2 et F85 = {1,2}) A quel moment ?
☐ 1 Avant votre premier rapport sexuel
☐ 2 Dans les trois premiers mois de votre relation
☐ 3 Plus tard dans votre relation
☐ 97. Non concerné (ne pas suggérer)
☐ 98. Ne sait pas (ne pas suggérer)
☐ 99. Refus de réponse (ne pas suggérer)

Poser F86 si Année(F9.a) > 1985
F86. Toujours au début de votre relation, quel était le statut sérologique de votre partenaire principale féminine ?
☐ 1 Séronégative
☐ 2 Séropositive
☐ 3 Vous ne saviez pas
☐ 4 Elle ne connaissait pas son statut
☐ 97. Non concerné (ne pas suggérer)
☐ 98. Ne sait pas (ne pas suggérer)
☐ 99. Refus de réponse (ne pas suggérer)

F86.a) (Si F86≠2) Votre partenaire est-elle séropositive aujourd'hui ?
☐ 1Oui
☐ 2Non
☐ 97. Non concerné (ne pas suggérer)
☐ 98. Ne sait pas (ne pas suggérer)
☐ 99. Refus de réponse (ne pas suggérer)

(Si F9.e=1 et date (F8a) - date(système/aujourd'hui) >12 mois, sinon aller au filtre au début de la partie D.3) Nous allons maintenant parler de vos relations sexuelles avec cette partenaire au cours des 12 derniers mois :

F87. Au cours des 12 derniers mois, vous avez pratiqué avec votre partenaire principale féminine :

F87.a) La fellation ?
☐ 1Jamais → F87.b)
☐ 2Rarement → **F87.a) bis**
☐ 3Parfois → **F87.a) bis**
☐ 4Souvent → **F87.a) bis**
☐ 5Toujours → **F87.a) bis**
☐ 97Non concerné (ne pas suggérer) → F87.b)
☐ 98 Ne sait pas (ne pas suggérer) → F87.b)
☐ 99 Refus de réponse (ne pas suggérer) → F87.b)

F87.a)bis Lors de la fellation, utilisez-vous des préservatifs?
☐ 1Jamais
☐ 2Presque jamais
☐ 3Parfois
☐ 4Presque toujours
☐ 5Toujours
☐ 97 Non concerné (ne pas suggérer)
☐ 98 Ne sait pas (ne pas suggérer)
☐ 99 Refus de réponse (ne pas suggérer)

F87.b) La pénétration vaginale ?
☐ 1Jamais → F87.c)
☐ 2 Presque jamais → **F87.b) bis**
☐ 3 Parfois → **F87.b) bis**
☐ 4 Presque toujours → **F87.b) bis**
☐ 5Toujours → **F87.b) bis**
☐ 97 Non concerné (ne pas suggérer) → F87.c)
☐ 98 Ne sait pas (ne pas suggérer) → F87.c)
☐ 99 Refus de réponse (ne pas suggérer) → F87.c)

F87.b)bis Et pour la pénétration vaginale, utilisez-vous des préservatifs ?
☐ 1Jamais
☐ 2Presque jamais
☐ 3Parfois
☐ 4Presque toujours
☐ 5Toujours
☐ 97 Non concerné (ne pas suggérer)
☐ 98 Ne sait pas (ne pas suggérer)
☐ 99 Refus de réponse (ne pas suggérer)

F87b) ter (F87.b) bis < 5) Vous retirez-vous avant d'éjaculer
☐ 1Jamais
☐ 2Presque jamais
☐ 3Parfois
☐ 4Presque toujours
☐ 5Toujours
☐ 97 Non concerné (ne pas suggérer)
☐ 98 Ne sait pas (ne pas suggérer)
☐ 99 Refus de réponse (ne pas suggérer)

F87.c) La pénétration anale ?
☐ 1Jamais → F88
☐ 2Rarement → **F87.c) bis**
☐ 3Parfois → **F87.c) bis**
☐ 4Souvent → **F87.c) bis**
☐ 4Toujours → **F87.c) bis**
☐ 97 Non concerné (ne pas suggérer) → F88
☐ 98 Ne sait pas (ne pas suggérer) → F88
☐ 99 Refus de réponse (ne pas suggérer) → F88

F87.c) bis Et pour la pénétration anale, utilisez-vous des préservatifs ?
☐ 1Jamais
☐ 2Presque jamais
☐ 3Parfois
☐ 4Presque toujours
☐ 5Toujours
☐ 97 Non concerné (ne pas suggérer)
☐ 98 Ne sait pas (ne pas suggérer)
☐ 99 Refus de réponse (ne pas suggérer)

F88. Au cours des 12 derniers mois, avez-vous utilisé des préservatifs féminins avec votre partenaire principale ?
☐ 1Jamais
☐ 2Presque jamais
☐ 3Parfois
☐ 4Presque toujours
☐ 5Toujours
☐ 97 Non concerné (ne pas suggérer)
☐ 98 Ne sait pas (ne pas suggérer)
☐ 99 Refus de réponse (ne pas suggérer))

F89. Et si l'on parle maintenant de votre dernier rapport sexuel avec cette partenaire, avez-vous utilisé un préservatif lors de la pénétration?
☐ 1 Oui
☐ 2 Non
☐ 97. Non concerné (ne pas suggérer)
☐ 98. Ne sait pas (ne pas suggérer)
☐ 99. Refus de réponse (ne pas suggérer)

F90. Pour vous, utiliser un préservatif pour la pénétration avec votre partenaire principale est : [De manière générale, lors de la pénétration anale et/ou vaginale**]**
☐ 1Très difficile
☐ 2Plutôt difficile
☐ 3Plutôt facile
☐ 4Très facile
☐ 97. Non concerné (ne pas suggérer)
☐ 98. Ne sait pas (ne pas suggérer)
☐ 99. Refus de réponse (ne pas suggérer)

F93. Au cours des 12 derniers mois, avez-vous eu d'autres partenaires sexuelles féminines que votre partenaire principale ?
☐ 1 Oui
☐ 2 Non
☐ 97. Non concerné (ne pas suggérer)
☐ 98. Ne sait pas (ne pas suggérer)
☐ 99. Refus de réponse (ne pas suggérer)

F93.a) (Si F93=1) Lui avez-vous dit ?
☐ 1 Oui
☐ 2 Non
☐ 97. Non concerné (ne pas suggérer)
☐ 98. Ne sait pas (ne pas suggérer)
☐ 99. Refus de réponse (ne pas suggérer)

F94. Depuis que vous la connaissez, est-il déjà arrivé que votre partenaire principale vous menace ou exerce une pression psychologique sur vous, pour avoir des relations sexuelles ?
(Elle a, au travers de mots blessant, dénigrant, médisant, rabaissant etc., ou encore au travers de sous- entendus, porté atteinte à votre moral)
☐ 1 Oui c'est arrivé dans les 12 derniers mois
☐ 2 Oui mais c'est arrivé il y a plus de 12 derniers mois
☐ 3 Non
☐ 97. Non concerné (ne pas suggérer)
☐ 98. Ne sait pas (ne pas suggérer)
☐ 99. Refus de réponse (ne pas suggérer)

F95. Depuis que vous la connaissez, est-il déjà arrivé que votre partenaire principale utilise la force physique pour avoir des relations sexuelles avec vous, ou qu'elle vous impose des gestes sexuels que vous refusiez ?
☐ 1 Oui c'est arrivé dans les 12 derniers mois
☐ 2 Oui mais c'est arrivé il y a plus de 12 derniers mois
☐ 3 Non
☐ 97. Non concerné (ne pas suggérer)
☐ 98. Ne sait pas (ne pas suggérer)
☐ 99. Refus de réponse (ne pas suggérer)

D.2 - Hétéro (Homme/FtoM) : Pas de partenaire principale au moment de la passation

Si A.1=1 ou F1a=1 ET F9 <>1

Attention : on doit poser même si F9=3o4 (refus ou NSP)

SINON : Filtres avant la question F97 (Début partie D.3)

Vous m'avez dit que vous n'avez pas de partenaire principale féminine actuellement (ou vous n'avez pas répondu à cette question)

F96. Avez-vous déjà eu une partenaire stable, une conjointe ou une compagne ?
☐ 1Oui ➔ **F96.a)**
☐ 2Non ➔ **Début partie D.3**
☐ 97 Non concerné (ne pas suggérer) ➔ **Début partie D.3**
☐ 98 Ne sait pas (ne pas suggérer) ➔ **Début partie D.3**
☐ 99 Refus de réponse (ne pas suggérer) ➔ **Début partie D.3**

Nous allons maintenant parler de cette relation ou de la dernière de ces relations avec une partenaire stable

F96.a) En quelle année cette relation a-t-elle commencé ?
_ _ _ _

F96.b) En quelle année cette relation s'est-elle terminée ?
_ _ _ _

F96.c) A ce moment là, connaissait-elle votre séropositivité ?
☐ 1Oui ➔ **F96.d)**
☐ 2Non ➔ **F96.c) bis**
☐ 3Vous ne savez pas ➔ **F96.c) bis**
☐ 4Vous n'étiez pas encore séropositif ➔ **F96.d)**
☐ 97 Non concerné (ne pas suggérer) ➔ **F96.d)**
☐ 99Refus de réponse (ne pas suggérer) ➔ **F96.d)**

F96.c) bis Aviez-vous peur d'être rejeté en le lui disant ?

- ☐ 1 Oui
- ☐ 2 Non
- ☐ 97. Non concerné (ne pas suggérer)
- ☐ 98. Ne sait pas (ne pas suggérer)
- ☐ 99. Refus de réponse (ne pas suggérer)

F96.d) Connaissiez vous son statut sérologique ?

- ☐ 1Oui, elle vous a dit qu'elle était séropositive ➔ **Début partie D.3**
- ☐ 2Oui, vous pensez qu'elle était séropositive ➔ **Début partie D.3**
- ☐ 3Oui, vous pensez qu'elle était séronégative ➔ **Début partie D.3**
- ☐ 4Oui, elle vous a dit qu'elle était séronégative ➔ **Début partie D.3**
- ☐ 5Non, vous ne le saviez pas ➔ **Début partie D.3**
- ☐ 97 Non concerné (ne pas suggérer) ➔ **Début partie D.3**
- ☐ 98 Ne sait pas (ne pas suggérer) ➔ **Début partie D.3**
- ☐ 99 Refus de réponse (ne pas suggérer) ➔ **Début partie D.3**

D.3 - Hétéro (Homme/FtoM) : Autres partenaires sexuels

Si (A.1=1) ou F1a=1) et

(Si F9=1 Et [(F5=1 et F93=1) ou (F5=1 et F9.e ≠ 1)] ou (F5=1 et date(F9.a)-date (aujourd'hui) < 12 mois])
Nous allons maintenant parler de vos partenaires sexuelles féminines en dehors de votre partenaire principale au cours des 12 derniers mois

Ou

(Si* F9≠1 et *F2.b* ≠ {1, 10,11} et F5=1*)
Nous allons maintenant parler de vos partenaires sexuelles féminines de ces 12 derniers mois

➔ Si aucun de ces filtres ➔ F106 (module commun : Début partie E1)

F97) Au cours des 12 derniers mois, combien de partenaires sexuelles féminines avez-vous eu, en dehors de votre partenaire principale (si vous en avez une)?
/_____/ **(➔ sur 4 positions) ➔ Si =0, refus ou NSP : aller au filtre début de la partie « E1 »**

F97a) Au cours des 4 dernières semaines, combien de relations sexuelles avez-vous eu avec ce ou ces partenaires?
/____/ ➔ Si =0,refus ou NSP : ➔ **F101b**

- ☐ 1 Aucun ➔ **F101b**
- ☐ 21 à 4 rapports sexuels (1 fois par semaine ou moins) ➔ **F101b**
- ☐ 35 à 8 rapports sexuels (entre 1 et 2 fois par semaine) ➔ **F101b**
- ☐ 49 à 12 rapports sexuels (entre 2 et 3 fois par semaine) ➔ **F101b**
- ☐ 513 à 16 rapports sexuels (entre 3 et 4 fois par semaine) ➔ **F101b**
- ☐ 6Plus de 16 rapports sexuels (plus de 4 fois par semaine) ➔ **F101b**
- ☐ 97 Non concerné (ne pas suggérer) ➔ **F101b**
- ☐ 98 Ne sait pas (ne pas suggérer) ➔ **F101b**
- ☐ 99 Refus de réponse (ne pas suggérer) ➔ **F101b**

F101.b) Pour vous, utiliser un préservatif avec ce type de partenaire sexuelle est :

- ☐ 1Très difficile
- ☐ 2Plutôt difficile
- ☐ 3Plutôt facile
- ☐ 4Très facile
- ☐ 97 Non concerné (ne pas suggérer)
- ☐ 98 Ne sait pas (ne pas suggérer)
- ☐ 99 Refus de réponse (ne pas suggérer)

(F6.b=1 partenaire) Nous allons maintenant nous intéresser à votre dernier rapport sexuel avec cette partenaire féminine

(F6.b>1 partenaire) Nous allons maintenant nous intéresser à votre dernier rapport sexuel avec l'une de ces partenaires féminines

F97. Etait-ce :

- ☐ 1Une relation d'un soir
- ☐ 2Une relation régulière ou suivie
- ☐ 97 Non concerné (ne pas suggérer)
- ☐ 98 Ne sait pas (ne pas suggérer)
- ☐ 99 Refus de réponse (ne pas suggérer)

F101. Quel était le statut sérologique de cette partenaire au moment de ce dernier rapport ?

- ☐ 1Séronégative
- ☐ 2Séropositive
- ☐ 3Vous ne saviez pas
- ☐ 97 Non concerné (ne pas suggérer)
- ☐ 98 Ne sait pas (ne pas suggérer)
- ☐ 99 Refus de réponse (ne pas suggérer)

F101.b) bis Et elle, savait-elle à ce moment là que vous étiez séropositif ?

- ☐ 1Oui, vous lui avez dit de vous-même
- ☐ 2Oui car elle vous l'avait demandé
- ☐ 3Non
- ☐ 4Vous n'avez pas eu à le faire, elle connaissait déjà votre statut
- ☐ 97 Non concerné (ne pas suggérer)
- ☐ 98 Ne sait pas (ne pas suggérer)
- ☐ 99 Refus de réponse (ne pas suggérer)

Nous allons maintenant parler plus précisément du <u>dernier rapport sexuel</u> avec cette partenaire :

F102. Lors de ce dernier rapport sexuel avec cette partenaire, avez-vous <u>utilisé un préservatif</u> :

F102.a) Pour la fellation
☐ $_1$Oui
☐ $_2$Non
☐ $_3$Vous n'avez pas pratiqué la fellation lors de ce dernier rapport
☐ $_{98}$ Ne sait pas (ne pas suggérer)
☐ $_{99}$ Refus de réponse (ne pas suggérer)

F102.b) Pour la pénétration vaginale
☐ $_1$Oui
☐ $_2$Non
☐ $_3$Vous n'avez pas pratiqué la pénétration vaginale lors de ce dernier rapport
☐ $_{98}$. Ne sait pas (ne pas suggérer)
☐ $_{99}$. Refus de réponse (ne pas suggérer)

F102.c) Pour la pénétration anale
☐ $_1$Oui
☐ $_2$Non
☐ $_3$Vous n'avez pas pratiqué la pénétration anale lors de ce dernier rapport
☐ $_{98}$. Ne sait pas (ne pas suggérer)
☐ $_{99}$. Refus de réponse (ne pas suggérer)

F102.d) (Si F102.b =2 ET/OU F102.c=2) Vous êtes-vous retiré avant d'éjaculer ?
☐ $_1$ Oui
☐ $_2$ Non
☐ $_{97}$. Non concerné (ne pas suggérer)
☐ $_{98}$. Ne sait pas (ne pas suggérer)
☐ $_{99}$. Refus de réponse (ne pas suggérer)

F103. <u>Est-ce que cette relation dure encore ?</u>
☐ $_1$Oui
☐ $_2$Non elle a duré quelques mois
☐ $_3$Non elle a duré quelques semaines
☐ $_4$Non elle n'a duré que quelques jours
☐ $_5$Non c'était un seul rapport sexuel
☐ $_6$Vous ne savez pas comment elle va se passer
☐ $_{97}$. Non concerné (ne pas suggérer)
☐ $_{98}$. Ne sait pas (ne pas suggérer)
☐ $_{99}$. Refus de réponse (ne pas suggérer)

F104. <u>Au cours de ces 12 derniers mois</u>, avez-vous cherché des partenaires sexuels féminines sur internet?
☐ $_1$ Jamais
☐ $_2$moins d'une fois par semaine
☐ $_3$une fois par semaine
☐ $_4$plus d'une fois par semaine
☐ $_5$tous les jours ou presque
☐ $_6$Vous n'utilisez pas internet
☐ $_{97}$. Non concerné (ne pas suggérer)
☐ $_{98}$. Ne sait pas (ne pas suggérer)
☐ $_{99}$. Refus de réponse (ne pas suggérer)

F105. Et pour rencontrer des partenaires féminines avez-vous utilisé des sites web, des petites annonces où le statut sérologique est systématiquement affiché au cours des 12 derniers mois?
☐ $_1$Jamais
☐ $_2$Presque jamais
☐ $_3$Parfois
☐ $_4$Presque toujours
☐ $_5$Toujours
☐ $_{97}$. Non concerné (ne pas suggérer)
☐ $_{98}$. Ne sait pas (ne pas suggérer)
☐ $_{99}$. Refus de réponse (ne pas suggérer)

E – MODULE COMMUN

E.1 - Rapports sexuels dans les douze derniers mois

Si F5=1
(Quels que soient le sexe et l'orientation sexuelle. Tous ceux qui ont eu des rapports dans les 12 derniers mois doivent passer E.1)

Nous allons maintenant vous poser quelques questions sur la façon dont vous ressentez actuellement votre vie sexuelle.

F106. Dans les 12 derniers mois :

F106.a) Avez-vous eu des douleurs lors des rapports ?
☐ 1 Oui
☐ 2 Non
☐ 97. Non concerné (ne pas suggérer)
☐ 98. Ne sait pas (ne pas suggérer)
☐ 99. Refus de réponse (ne pas suggérer)

F106.b) (Si A1=1) Avez-vous eu des troubles de l'érection ?
☐ 1 Oui
☐ 2 Non
☐ 97. Non concerné (ne pas suggérer)
☐ 98. Ne sait pas (ne pas suggérer)
☐ 99. Refus de réponse (ne pas suggérer)

F106.c) Diriez vous que vous n'avez pas eu assez de rapports sexuels ?
☐ 1 Oui
☐ 2 Non
☐ 97. Non concerné (ne pas suggérer)
☐ 98. Ne sait pas (ne pas suggérer)
☐ 99. Refus de réponse (ne pas suggérer)

F106.d) Avez-vous ressenti une absence de désir sexuel, la baisse ou perte de la libido ?
☐ 1 Oui
☐ 2 Non
☐ 97. Non concerné (ne pas suggérer)
☐ 98. Ne sait pas (ne pas suggérer)
☐ 99. Refus de réponse (ne pas suggérer)

F106.e) Est-il arrivé qu'un préservatif se déchire ou glisse au cours d'un rapport ?
☐ 1 Oui
☐ 2 Non
☐ 97. Non concerné (ne pas suggérer)
☐ 98. Ne sait pas (ne pas suggérer)
☐ 99. Refus de réponse (ne pas suggérer)

F106.e)bis (si F106.e)=1)Combien de fois ?
/__/__/

F106.f) Avez-vous privilégié les relations sexuelles avec des partenaires séropositifs ?
☐ 1 Oui
☐ 2 Non
☐ 97. Non concerné (ne pas suggérer)
☐ 98. Ne sait pas (ne pas suggérer)
☐ 99. Refus de réponse (ne pas suggérer)

F107. Au cours des 12 derniers mois, avez-vous :
☐ 1 Oui
☐ 2 Non
☐ 97. Non concerné (ne pas suggérer)
☐ 98. Ne sait pas (ne pas suggérer)
☐ 99. Refus de réponse (ne pas suggérer)

a) Eté infecté par le virus de l'hépatite C ?
b) Eu une Syphilis ?
c) Eu une Lymphogranulomatose Vénérienne (LGV) ?
d) Eu une ou plusieurs gonorrhées ?
e) Eu des condylomes ?
f) Eu une autre infection sexuellement transmissible ?

F108. Comment qualifieriez-vous votre vie sexuelle actuelle ?
☐ 1 Très Satisfaisante
☐ 2 Plutôt satisfaisante
☐ 3 Plutôt pas satisfaisante
☐ 4 Pas satisfaisante
☐ 97 Non concerné (ne pas suggérer)
☐ 98 Ne sait pas (ne pas suggérer)
☐ 99 Refus de réponse (ne pas suggérer)

E.2 - Pas de rapports sexuels dans les douze derniers mois

Filtre : Si F5≠1
(Quel que soit le sexe et l'orientation sexuelle. Tous ceux qui n'ont pas eu de rapports dans les 12 derniers mois doivent passer E.1)

Vous m'avez dit que vous n'aviez pas eu de rapports sexuels dans les 12 derniers mois :

F109. Quelle est la date de votre dernier rapport sexuel ?
_ _/ _ _ _ _ (MM/AAAA)

F110. Si vous n'avez pas d'activité sexuelle actuellement, c'est parce que…
- ☐ 1 Oui
- ☐ 2 Non
- ☐ 97. Non concerné (ne pas suggérer)
- ☐ 98. Ne sait pas (ne pas suggérer)
- ☐ 99. Refus de réponse (ne pas suggérer)

a) Vous n'en avez tout simplement pas envie
b) Vous n'avez pas trouvé quelqu'un qui vous plaise
c) Vous êtes trop fatigué(e), en trop mauvaise santé
d) Vous vous sentez moins séduisant(e)
e) Vous vous sentez isolé(e) et vous ne savez pas comment rencontrer quelqu'un
f) Vous avez peur d'être rejeté(e)
g) Vous avez peur de contaminer votre partenaire
h) Vous préférez ne pas avoir de rapports plutôt que d'avoir à utiliser des préservatifs
i) Vous avez des problèmes sexuels (impuissance, frigidité, troubles de l'érection…)

E.3 - Pour tous :

F111. Avez-vous entendu parler d'un traitement d'urgence qui, pris juste après un rapport non protégé ou une rupture de préservatif peut empêcher la transmission du VIH à une personne séronégative ?

- ☐ 1 Oui
- ☐ 2 Non
- ☐ 97. Non concerné (ne pas suggérer)
- ☐ 98. Ne sait pas (ne pas suggérer)
- ☐ 99. Refus de réponse (ne pas suggérer)

F112. Au cours des deux dernières années, est-il arrivé :

F112.a) …qu'une personne refuse d'avoir un rapport sexuel avec vous parce que vous êtes séropositif/ve ?
- ☐ 1 Oui
- ☐ 2 Non
- ☐ 97. Non concerné (ne pas suggérer)
- ☐ 98. Ne sait pas (ne pas suggérer)
- ☐ 99. Refus de réponse (ne pas suggérer)

F112.b) …qu'un partenaire sexuel rompe avec vous parce que vous êtes séropositif/ve.
- ☐ 1 Oui
- ☐ 2 Non
- ☐ 97. Non concerné (ne pas suggérer)
- ☐ 98. Ne sait pas (ne pas suggérer)
- ☐ 99. Refus de réponse (ne pas suggérer)

MODULE G : SANTE MENTALE

Nous allons maintenant vous poser des questions sur votre moral

EPISODE DEPRESSIF CARACTERISE

G.1 **G1. Au cours des 12 derniers mois, vous est-il arrivé de vivre une période d'au moins deux semaines d'affilée pendant lesquelles vous vous sentiez constamment triste, déprimé(e), sans espoir ?**
- ☐ 1 Oui ➔ G2
- ☐ 2 Non ➔ G4
- ☐ 97. Non concerné (ne pas suggérer) ➔ G4
- ☐ 98. Ne sait pas (ne pas suggérer) ➔ G4
- ☐ 99. Refus de réponse (ne pas suggérer) ➔ G4

G.2 **G2. Pendant cette période, ces sentiments de tristesse duraient-ils ?**
- ☐ 1 Toute la journée ➔ G3
- ☐ 2 Une bonne partie de la journée ➔ G3
- ☐ 3 Environ la moitié de la journée ➔ G3
- ☐ 4 Moins de la moitié ➔ G4
- ☐ 97. Non concerné (ne pas suggérer) ➔ G4
- ☐ 98. Ne sait pas (ne pas suggérer) ➔ G4
- ☐ 99. Refus de réponse (ne pas suggérer) ➔ G4

G.3 **G3. Pendant cette période, ces sentiments de tristesse arrivaient-ils ?**
- ☐ 1 Chaque jour ➔ G7
- ☐ 2 Presque chaque jour ➔ G7
- ☐ 3 Moins souvent ➔ G4
- ☐ 97. Non concerné (ne pas suggérer) ➔ G4
- ☐ 98. Ne sait pas (ne pas suggérer) ➔ G4
- ☐ 99. Refus de réponse (ne pas suggérer) ➔ G4

G.4 **G4. Au cours des 12 derniers mois, vous est-il arrivé de vivre une période d'au moins deux semaines d'affilée, pendant lesquelles vous avez perdu de l'intérêt pour la plupart des choses comme les loisirs, le travail ou les activités qui vous donnent habituellement du plaisir ?**
- ☐ 1 Oui ➔ G5
- ☐ 2 Non ➔ G7
- ☐ 97. Non concerné (ne pas suggérer) ➔ G7
- ☐ 98. Ne sait pas (ne pas suggérer) ➔ G7
- ☐ 99. Refus de réponse (ne pas suggérer) ➔ G7

G.5 **G5. Pendant cette période, ressentiez-vous cette perte d'intérêt ?**
- ☐ 1 Toute la journée ➔ G6
- ☐ 2 Une bonne partie de la journée ➔ G6
- ☐ 3 Environ la moitié de la journée ➔ G6
- ☐ 4 Moins de la moitié ➔ G7
- ☐ 97. Non concerné (ne pas suggérer) ➔ G7
- ☐ 98. Ne sait pas (ne pas suggérer) ➔ G7
- ☐ 99. Refus de réponse (ne pas suggérer) ➔ G7

G.6 **G6. Pendant cette période, cette perte d'intérêt arrivait-elle ?**
- ☐ 1 Chaque jour ➔ G7
- ☐ 2 Presque chaque jour ➔ G7
- ☐ 3 Moins souvent ➔ G7
- ☐ 97. Non concerné (ne pas suggérer) ➔ G7
- ☐ 98. Ne sait pas (ne pas suggérer) ➔ G7
- ☐ 99. Refus de réponse (ne pas suggérer) ➔ G7

ANXIETE GENERALISEE

G.7 **G7. Au cours des 12 derniers mois avez vous eu une période d'au moins un mois où vous vous sentiez presque tout le temps inquiet, tendu ou anxieux?**
- ☐ 1 Oui ➔ G8
- ☐ 2Non ➔ G9
- ☐ 97. Non concerné (ne pas suggérer) ➔ G9
- ☐ 98. Ne sait pas (ne pas suggérer) ➔ G9
- ☐ 99. Refus de réponse (ne pas suggérer) ➔ G9

G.8 **Combien de mois cet épisode a-t-il duré ?**
ENQ : SI NSP, NOTER 99
/__/__/ mois

Si moins de 6 mois (< 6) passez à G9
Sinon passer à G11

G9. Dans une situation donnée, tout le monde ne s'inquiète pas de la même manière. Durant les 12 derniers mois avez vous eu le sentiment de vous faire beaucoup plus de soucis que la plupart des gens dans la même situation ?

- ☐ 1Oui ➔ G10
- ☐ 2 Non ➔ G11
- ☐ 97. Non concerné (ne pas suggérer) ➔ G11
- ☐ 98. Ne sait pas (ne pas suggérer) ➔ G11
- ☐ 99. Refus de réponse (ne pas suggérer) ➔ G11

G10. Combien de mois cet épisode a-t-il duré ?
ENQ : SI NSP, NOTER 99
/__/__/ mois

NEVEROSE POST TRAUMATIQUE

G11. Avez-vous vécu à un moment donné de votre vie un événement terrible, effrayant, horrible qui vous a occasionné des problèmes comme : des souvenirs ou des rêves bouleversants, le sentiment d'être détaché(e) des autres, des problèmes de sommeil ou de concentration, ou bien une nervosité excessive ?
- ☐ 1 Oui
- ☐ 2 Non
- ☐ 97. Non concerné (ne pas suggérer)
- ☐ 98. Ne sait pas (ne pas suggérer)
- ☐ 99. Refus de réponse (ne pas suggérer)

EPISODE DEPRESSIF CARACTERISE- HUMEUR DEPRESSIVE

Poser les questions G12 à G40 uniquement SI G3=1 OU G3=2

G12. Vous nous avez dit avoir vécu une période où vous étiez, constamment triste, déprimé(e), sans espoir ; pendant cette période aviez-vous perdu intérêt pour la plupart des choses comme les loisirs, le travail ou les activités qui vous donnent habituellement du plaisir ?
- ☐ 1 Oui
- ☐ 2 Non
- ☐ 97. Non concerné (ne pas suggérer)
- ☐ 98. Ne sait pas (ne pas suggérer)
- ☐ 99. Refus de réponse (ne pas suggérer)

G13. Pendant cette même période, vous sentiez-vous épuisé(e) ou manquiez-vous d'énergie plus que d'habitude ?
- ☐ 1 Oui
- ☐ 2 Non
- ☐ 97. Non concerné (ne pas suggérer)
- ☐ 98. Ne sait pas (ne pas suggérer)
- ☐ 99. Refus de réponse (ne pas suggérer)

G14. Pendant cette même période avez-vous pris ou perdu du poids sans le vouloir, ou votre poids est-il resté stable ?
- ☐ 1 Vous avez pris du poids ➔**G15**
- ☐ 2 Vous avez perdu du poids ➔ **G15**
- ☐ 3 Votre poids n'a pas changé
- ☐ 97. Non concerné (ne pas suggérer)
- ☐ 98. Ne sait pas (ne pas suggérer)
- ☐ 99. Refus de réponse (ne pas suggérer)

Si G14 = 1 ou 2 poser G15

G15. Combien avez vous pris ou perdu de kilos ?

/_/_/ Kilos /_/_/ grammes

G16. Pendant cette période avez-vous eu plus de difficultés que d'habitude à vous endormir ?
- ☐ 1 Oui
- ☐ 2 Non ➔ **G18**
- ☐ 97. Non concerné (ne pas suggérer) ➔ **G18**
- ☐ 98. Ne sait pas (ne pas suggérer) ➔ **G18**
- ☐ 99. Refus de réponse (ne pas suggérer) ➔ **G18**

G17. Pendant cette période, cela arrivait-il ?
- ☐ 1 Chaque nuit ➔ **G19**
- ☐ 2 Presque chaque nuit ➔ **G19**
- ☐ 3 Moins souvent
- ☐ 97. Non concerné (ne pas suggérer)
- ☐ 98. Ne sait pas (ne pas suggérer)
- ☐ 99. Refus de réponse (ne pas suggérer)

G18. Pendant cette période, aviez-vous d'autres problèmes de sommeil, presque toutes les nuits comme des difficultés à rester endormi(e), ou des réveils beaucoup trop matinaux ou au contraire dormiez-vous trop ou aviez- vous tout le temps envie de dormir ?
- ☐ 1 Oui
- ☐ 2 Non
- ☐ 97. Non concerné (ne pas suggérer)
- ☐ 98. Ne sait pas (ne pas suggérer)
- ☐ 99. Refus de réponse (ne pas suggérer)

G19. Toujours pendant cette période, parliez-vous ou vous déplaciez-vous plus lentement que d'habitude, et ce presque tous les jours ?
- ☐ 1 Oui ➔ **G20**
- ☐ 2 Non ➔ **G21**
- ☐ 97. Non concerné (ne pas suggérer) ➔ **G20**
- ☐ 98. Ne sait pas (ne pas suggérer) ➔ **G20**
- ☐ 99. Refus de réponse (ne pas suggérer) ➔ **G20**

G20. Quelqu'un vous l'a-t-il fait remarquer ?
- ☐ 1 Oui ➔ **G23**
- ☐ 2 Non ➔ **G21**
- ☐ 97. Non concerné (ne pas suggérer) ➔ **G21**
- ☐ 98. Ne sait pas (ne pas suggérer) ➔ **G21**
- ☐ 99. Refus de réponse (ne pas suggérer) ➔ **G21**

G21.Etiez-vous au contraire, beaucoup plus agité(e) ou nerveux (se) que d'habitude au point de ne pas pouvoir tenir en place presque tous les jours ?
- ☐ 1 Oui ➔ **G22**
- ☐ 2 Non ➔ G23
- ☐ 97. Non concerné (ne pas suggérer) ➔ **G22**
- ☐ 98. Ne sait pas (ne pas suggérer) ➔ **G22**
- ☐ 99. Refus de réponse (ne pas suggérer) ➔ **G22**

G22. Quelqu'un vous l'a-t-il fait remarquer ?

- ☐ 1 Oui
- ☐ 2 Non
- ☐ 97. Non concerné (ne pas suggérer)
- ☐ 98. Ne sait pas (ne pas suggérer)
- ☐ 99. Refus de réponse (ne pas suggérer)

G23.Pendant <u>cette période</u> avez-vous eu beaucoup plus de mal que d'habitude à vous concentrer ?

- ☐ 1 Oui ➔ G25
- ☐ 2 Non
- ☐ 97. Non concerné (ne pas suggérer)
- ☐ 98. Ne sait pas (ne pas suggérer)
- ☐ 99. Refus de réponse (ne pas suggérer)

G24. Aviez-vous beaucoup plus de mal que d'habitude à prendre des décisions ou encore vos pensées étaient-elles embrouillées ou beaucoup plus lentes que d'habitude ?

- ☐ 1 Oui
- ☐ 2 Non
- ☐ 97. Non concerné (ne pas suggérer)
- ☐ 98. Ne sait pas (ne pas suggérer)
- ☐ 99. Refus de réponse (ne pas suggérer)

G25. Parfois les gens perdent confiance en eux, se sentent sans valeur inférieurs aux autres ou bons à rien. Pendant <u>cette période</u>, avez-vous ressenti cela ?

- ☐ 1 Oui ➔ G27
- ☐ 2 Non
- ☐ 97. Non concerné (ne pas suggérer)
- ☐ 98. Ne sait pas (ne pas suggérer)
- ☐ 99. Refus de réponse (ne pas suggérer)

G26. Vous sentiez-vous coupable de tout ?

- ☐ 1 Oui
- ☐ 2 Non
- ☐ 97. Non concerné (ne pas suggérer)
- ☐ 98. Ne sait pas (ne pas suggérer)
- ☐ 99. Refus de réponse (ne pas suggérer)

G27. Pendant <u>cette période</u> avez vous beaucoup pensé à la mort, que ce soit votre mort, celle de quelqu'un d'autre ou la mort en général ?

- ☐ 1 Oui
- ☐ 2 Non
- ☐ 97. Non concerné (ne pas suggérer)
- ☐ 98. Ne sait pas (ne pas suggérer)
- ☐ 99. Refus de réponse (ne pas suggérer)

G28. Sur une échelle de 0 à 10 où 0 signifie une absence de gêne et 10 signifie une gêne sévère, quel chiffre décrit à quel point votre humeur triste vous a gêné au cours <u>des 12 derniers mois</u> dans chacune des activités suivantes ?

G28.1 - Vos tâches ménagères (le ménage, les courses ou s'occuper de la maison, de l'appartement) /__/__/

G28.2 - Votre capacité de travail /__/__/

G28.3 - Votre capacité à établir et à maintenir des relations proches avec les autres /__/__/

G28.4 - Votre vie sociale /__/__/

G29. Durant ces <u>12 derniers mois</u>, pendant environ combien de jours avez-vous été totalement incapable de travailler ou d'effectuer vos activités normales à cause de votre humeur triste ?

/__/__/ Jours

G34. Une telle période de grande tristesse est-elle survenue moins de deux mois après un événement qui vous aurait bouleversé ?

- ☐ 1 Oui
- ☐ 2 Non ➔ G36
- ☐ 97. Non concerné (ne pas suggérer) ➔ G36
- ☐ 98. Ne sait pas (ne pas suggérer) ➔ G36
- ☐ 99. Refus de réponse (ne pas suggérer) ➔ G36

G35. Quel était cet ou ces événements ?

PLUSIEURS REPONSES POSSIBLES

- ☐ 1 Décès d'un proche (conjoint, enfant, parents ou ami intime)
- ☐ 2 Séparation, divorce
- ☐ 3 Départ d'un proche
- ☐ 4 Maladie ou accident d'un proche
- ☐ 5 Annonce de votre séropositivité
- ☐ 6 Annonce de la séropositivité d'un proche
- ☐ 7 Perte d'emploi et/ou de revenus
- ☐ 8 Autre événement (ne pas préciser)
- ☐ 97. Non concerné (ne pas suggérer)
- ☐ 98. Ne sait pas (ne pas suggérer)
- ☐ 99. Refus de réponse (ne pas suggérer)

G36. Ces problèmes de tristesse, de découragement ou de perte d'intérêt étaient- ils causés par le VIH ?

- ☐ 1 Toujours
- ☐ 2 Quelquefois
- ☐ 3 Jamais
- ☐ 97. Non concerné (ne pas suggérer)
- ☐ 98. Ne sait pas (ne pas suggérer)
- ☐ 99. Refus de réponse (ne pas suggérer)

G37. Ces problèmes de tristesse, de découragement ou de perte d'intérêt étaient- ils causés par une autre maladie physique, ou par la consommation d'alcool ou de drogue ?
ENQ : ENUMERER
- ☐ 1 Toujours
- ☐ 2 Quelquefois
- ☐ 3 Jamais
- ☐ 97. Non concerné (ne pas suggérer)
- ☐ 98. Ne sait pas (ne pas suggérer)
- ☐ 99. Refus de réponse (ne pas suggérer)

G38. Durant ces 12 derniers mois, pendant environ combien de semaines au total avez-vous ressenti ces problèmes ?
Si non concerné TAPER NR

/__/__/ semaines

G39. Quel âge aviez-vous approximativement quand vous avez vécu une telle période de tristesse profonde accompagnée de ces différents problèmes pour la première fois ?
Si non concerné TAPER NR
/__/__/ Ans

G40. Avez-vous eu une période de quelques jours ou plus où vous étiez tellement nerveux (se) ou excité (e) que cela vous a attiré des ennuis, que les gens se sont inquiétés pour vous, ou qu'un médecin vous a dit que vous étiez dans une phase maniaque ?
- ☐ 1 Oui
- ☐ 2 Non
- ☐ 97. Non concerné (ne pas suggérer)
- ☐ 98. Ne sait pas (ne pas suggérer)
- ☐ 99. Refus de réponse (ne pas suggérer)

EPISODE DEPRESSIF CARACTERISE – ANHEDONIE

Poser les questions G41 à G68 uniquement si G6 = 1 ou 2

G41. Pendant cette période où vous avez perdu tout intérêt et plaisir pour la plupart des choses comme les loisirs, le travail ou les activités qui vous donnent habituellement du plaisir, vous sentiez-vous épuisé(e) ou manquiez-vous d'énergie plus que d'habitude ?
- ☐ 1 Oui
- ☐ 2 Non
- ☐ 97. Non concerné (ne pas suggérer)
- ☐ 98. Ne sait pas (ne pas suggérer)
- ☐ 99. Refus de réponse (ne pas suggérer)

G42. Pendant cette même période avez-vous pris ou perdu du poids sans le vouloir, ou votre poids est-il resté stable ?
- ☐ 1 Vous avez pris du poids ➔ **G43**
- ☐ 2 Vous avez perdu du poids ➔ **G43**
- ☐ 3 Votre poids n'a pas changé
- ☐ 97. Non concerné (ne pas suggérer)
- ☐ 98. Ne sait pas (ne pas suggérer)
- ☐ 99. Refus de réponse (ne pas suggérer)

G43. Combien avez-vous pris ou perdu de kilos ?
/_/_/ Kilos /_/_/ grammes

G44. Pendant cette période avez-vous eu plus de difficultés que d'habitude à vous endormir ?
- ☐ 1 Oui
- ☐ 2 Non ➔ G46
- ☐ 97. Non concerné (ne pas suggérer) ➔ G46
- ☐ 98. Ne sait pas (ne pas suggérer) ➔ G46
- ☐ 99. Refus de réponse (ne pas suggérer) ➔ G46

G45. Pendant cette période, cela arrivait-il…
- ☐ 1 Chaque nuit ➔ G47
- ☐ 2 Presque chaque nuit ➔ G47
- ☐ 3 Moins souvent
- ☐ 97. Non concerné (ne pas suggérer)
- ☐ 98. Ne sait pas (ne pas suggérer)
- ☐ 99. Refus de réponse (ne pas suggérer)

G46. Pendant cette période, aviez-vous d'autres problèmes de sommeil, presque toutes les nuits comme des difficultés à rester endormi(e), ou des réveils beaucoup trop matinaux ou au contraire dormiez-vous trop ou aviez- vous tout le temps envie de dormir ?
- ☐ 1 Oui
- ☐ 2 Non
- ☐ 97. Non concerné (ne pas suggérer)
- ☐ 98. Ne sait pas (ne pas suggérer)
- ☐ 99. Refus de réponse (ne pas suggérer)

G47. Toujours pendant cette même période, parliez-vous ou vous déplaciez-vous plus lentement que d'habitude, et ce presque tous les jours ?
- ☐ 1 Oui
- ☐ 2 Non ➔ G49
- ☐ 97. Non concerné (ne pas suggérer) ➔ G49
- ☐ 98. Ne sait pas (ne pas suggérer) ➔ G49
- ☐ 99. Refus de réponse (ne pas suggérer) ➔ G49

G48. Quelqu'un vous l'a-t-il fait remarquer ?
- ☐ 1 Oui
- ☐ 2 Non
- ☐ 97. Non concerné (ne pas suggérer)
- ☐ 98. Ne sait pas (ne pas suggérer)
- ☐ 99. Refus de réponse (ne pas suggérer)

G49. Etiez-vous au contraire, beaucoup plus agité(e) ou nerveux (se) que d'habitude au point de ne pas pouvoir tenir en place presque tous les jours?

- ☐ 1 Oui
- ☐ 2 Non ➔ G51
- ☐ 97. Non concerné (ne pas suggérer) ➔ G51
- ☐ 98. Ne sait pas (ne pas suggérer) ➔ G51
- ☐ 99. Refus de réponse (ne pas suggérer) ➔ G51

G50. Quelqu'un vous l'a-t-il fait remarquer ?

- ☐ 1 Oui
- ☐ 2 Non
- ☐ 97. Non concerné (ne pas suggérer)
- ☐ 98. Ne sait pas (ne pas suggérer)
- ☐ 99. Refus de réponse (ne pas suggérer)

G51. Pendant cette période avez-vous eu beaucoup plus de mal que d'habitude à vous concentrer ?

- ☐ 1 Oui ➔ G53
- ☐ 2 Non
- ☐ 97. Non concerné (ne pas suggérer)
- ☐ 98. Ne sait pas (ne pas suggérer)
- ☐ 99. Refus de réponse (ne pas suggérer)

G52. Aviez-vous beaucoup plus de mal que d'habitude à prendre des décisions ou encore vos pensées étaient-elles embrouillées ou beaucoup plus lentes que d'habitude ?

- ☐ 1 Oui
- ☐ 2 Non
- ☐ 97. Non concerné (ne pas suggérer)
- ☐ 98. Ne sait pas (ne pas suggérer)
- ☐ 99. Refus de réponse (ne pas suggérer)

G53. Parfois les gens perdent confiance en eux, se sentent sans valeur inférieurs aux autres ou bons à rien. Pendant cette période, avez-vous ressenti cela ?

- ☐ 1 Oui ➔ G55
- ☐ 2 Non
- ☐ 97. Non concerné (ne pas suggérer)
- ☐ 98. Ne sait pas (ne pas suggérer)
- ☐ 99. Refus de réponse (ne pas suggérer)

G54. Vous sentiez-vous coupable de tout ?

- ☐ 1 Oui
- ☐ 2 Non
- ☐ 97. Non concerné (ne pas suggérer)
- ☐ 98. Ne sait pas (ne pas suggérer)
- ☐ 99. Refus de réponse (ne pas suggérer)

G55. Pendant cette période avez vous beaucoup pensé à la mort, que ce soit votre mort, celle de quelqu'un d'autre ou la mort en général ?

- ☐ 1 Oui
- ☐ 2 Non
- ☐ 97. Non concerné (ne pas suggérer)
- ☐ 98. Ne sait pas (ne pas suggérer)
- ☐ 99. Refus de réponse (ne pas suggérer)

G56. Sur une échelle de 0 à 10 où 0 signifie une absence de gêne et 10 signifie une gêne sévère, quel chiffre décrit à quel point votre humeur triste vous a gêné au cours des 12 derniers mois dans chacune des activités suivantes ?

G56.1 - Vos tâches ménagères (le ménage, les courses ou s'occuper de la maison, de l'appartement /__/__/

G56.2 - Votre capacité de travail /__/__/

G56.3 - Votre capacité à établir et à maintenir des relations proches avec les autres /__/__/

G56.4 - Votre vie sociale /__/__/

G57. Durant <u>ces 12 derniers mois</u>, pendant environ combien de jours avez-vous été totalement incapable de travailler ou d'effectuer vos activités normales à cause de votre humeur triste ?

/__/__/ Jours

G62. Une telle période était-elle survenue (<u>moins de deux mois</u>) après un événement qui vous aurait bouleversé

- ☐ 1 Oui
- ☐ 2 Non ➔ G64
- ☐ 97. Non concerné (ne pas suggérer) ➔ G64
- ☐ 98. Ne sait pas (ne pas suggérer) ➔ G64
- ☐ 99. Refus de réponse (ne pas suggérer) ➔ G64

G63. Quel était cet ou ces événement(s) ?

UNE SEULE REPONSE POSSIBLE

- ☐ 1 Décès d'un proche (conjoint, enfant, parents ou ami intime)
- ☐ 2 Séparation, divorce
- ☐ 3 Départ d'un proche
- ☐ 4 Maladie ou accident d'un proche
- ☐ 5 Annonce de votre séropositivité
- ☐ 6 Annonce de la séropositivité d'un proche
- ☐ 7 Perte d'emploi et/ou de revenus
- ☐ 8 Autre événement (ne pas préciser)
- ☐ 97. Non concerné (ne pas suggérer)
- ☐ 98. Ne sait pas (ne pas suggérer)
- ☐ 99. Refus de réponse (ne pas suggérer)

G64. Ces problèmes de tristesse, de découragement ou de perte d'intérêt étaient- ils causés par le VIH ?

- ☐ 1 Toujours
- ☐ 2 Quelquefois
- ☐ 3 Jamais
- ☐ 97. Non concerné (ne pas suggérer)
- ☐ 98. Ne sait pas (ne pas suggérer)
- ☐ 99. Refus de réponse (ne pas suggérer)

G65. Ces problèmes de tristesse, de découragement ou de perte d'intérêt étaient- ils causés par une autre maladie physique, ou par la consommation d'alcool ou de drogue ?

- ☐ 1 Toujours
- ☐ 2 Quelquefois
- ☐ 3 Jamais
- ☐ 97. Non concerné (ne pas suggérer)
- ☐ 98. Ne sait pas (ne pas suggérer)
- ☐ 99. Refus de réponse (ne pas suggérer)

G66. Durant ces 12 derniers mois, pendant environ combien de semaines au total avez-vous ressenti ces problèmes ?

/__/__/ semaines

G67. Quel âge aviez-vous approximativement quand vous avez vécu une telle période de tristesse profonde accompagnée de ces différents problèmes pour la première fois ?

/__/__/ Ans

G68. Avez-vous eu une période de quelques jours ou plus où vous étiez tellement nerveux (se) ou excité (e) que cela vous a attiré des ennuis, que les gens se sont inquiétés pour vous, ou qu'un médecin vous a dit que vous étiez dans une phase maniaque ?

- ☐ 1 Oui
- ☐ 2 Non
- ☐ 97. Non concerné (ne pas suggérer)
- ☐ 98. Ne sait pas (ne pas suggérer)
- ☐ 99. Refus de réponse (ne pas suggérer)

NEVROSE POST TRAUMATIQUE

Poser uniquement si G11=1

G 84. Au cours de votre vie, avez-vous, vécu un des événements suivants ?

G84.1 - Eté victime d'un vol avec effraction ou d'une menace à main armée

- ☐ 1 Oui
- ☐ 2 Non
- ☐ 97. Non concerné (ne pas suggérer)
- ☐ 98. Ne sait pas (ne pas suggérer)
- ☐ 99. Refus de réponse (ne pas suggérer)

G84.2 - Eté sévèrement agressé(e) par un proche

- ☐ 1 Oui
- ☐ 2 Non
- ☐ 97. Non concerné (ne pas suggérer)
- ☐ 98. Ne sait pas (ne pas suggérer)
- ☐ 99. Refus de réponse (ne pas suggérer)

G84.3 - Eu un accident ou une maladie qui mettait en jeu votre vie

- ☐ 1 Oui ➔ **G84.3B**
- ☐ 2 Non
- ☐ 97. Non concerné (ne pas suggérer)
- ☐ 98. Ne sait pas (ne pas suggérer)
- ☐ 99. Refus de réponse (ne pas suggérer)

Si G84.3=1

G84.3B. S'agit-il de l'annonce du VIH »

- ☐ 1 Oui
- ☐ 2 Non
- ☐ 97. Non concerné (ne pas suggérer)
- ☐ 98. Ne sait pas (ne pas suggérer)
- ☐ 99. Refus de réponse (ne pas suggérer)

G84.4 - Eté violé(e) ou agressé(e) sexuellement

- ☐ 1 Oui
- ☐ 2 Non
- ☐ 97. Non concerné (ne pas suggérer)
- ☐ 98. Ne sait pas (ne pas suggérer)
- ☐ 99. Refus de réponse (ne pas suggérer)

G84.5 - Eté pris dans une catastrophe comme un incendie, une inondation

- ☐ 1 Oui
- ☐ 2 Non
- ☐ 97. Non concerné (ne pas suggérer)
- ☐ 98. Ne sait pas (ne pas suggérer)
- ☐ 99. Refus de réponse (ne pas suggérer)

G84.6 – Eté pris dans un tremblement de terre, un glissement de terrain ou un ouragan

- ☐ 1 Oui
- ☐ 2 Non
- ☐ 97. Non concerné (ne pas suggérer)
- ☐ 98. Ne sait pas (ne pas suggérer)
- ☐ 99. Refus de réponse (ne pas suggérer)

G84.7 - Vécu la mort inattendue, subite d'un ami intime ou d'un membre de votre famille
☐ 1 Oui
☐ 2 Non
☐ 97. Non concerné (ne pas suggérer)
☐ 98. Ne sait pas (ne pas suggérer)
☐ 99. Refus de réponse (ne pas suggérer)

G84.8 - Fait quelque chose qui a abouti à la blessure ou au décès d'une autre personne
☐ 1 Oui
☐ 2 Non
☐ 97. Non concerné (ne pas suggérer)
☐ 98. Ne sait pas (ne pas suggérer)
☐ 99. Refus de réponse (ne pas suggérer)

G84.9 – Vécu un autre évènement que nous n'avons pas cité ?
☐ 1 Oui → **poser G85**
☐ 2 Non
☐ 97. Non concerné (ne pas suggérer)
☐ 98. Ne sait pas (ne pas suggérer)
☐ 99. Refus de réponse (ne pas suggérer)

Si G84.9 = 1, poser G85
G85. Quel est cet autre évènement ?

Poser G85.bis si au moins un code 1 (oui) de la sq G84.1 à G84.9 et G85
G. 85 bis, Parmi les événements que vous avez cité, quel a été l'événement le plus marquant pour vous ? ………………………………………………………
ENQ : seul cet événement sera considéré dans les questions suivantes :
Afficher seulement les réponses citées (code 1) par le répondant de la sq G84.1 à G84.9 et G85 si la personne a répondu autre aussi

G85.1bis - Eté victime d'un vol avec effraction ou d'une menace à main armée
G85.2 bis - Eté sévèrement agressé(e) par un proche
G85.3 bis - Eu un accident ou une maladie qui mettait en jeu votre vie
G85.3B bis**. S'agit-il de l'annonce du VIH »**
G85.4 bis - Eté violé(e) ou agressé(e) sexuellement
G85.5 bis - Eté pris dans une catastrophe comme un incendie, une inondation
G85.6 bis – Eté pris dans un tremblement de terre, un glissement de terrain ou un ouragan
G85.7 bis - Vécu la mort inattendue, subite d'un ami intime ou d'un membre de votre famille
G85.8 bis - Fait quelque chose qui a abouti à la blessure ou au décès d'une autre personne
G85.9 bis – Vécu un autre évènement que nous n'avons pas cité ? Afficher réponse G85

Nous allons donc parler de cet évènement dans les questions suivantes :

G86. Quand vous avez été confronté à cet évènement, avez-vous eu un sentiment de peur très intense, d'impuissance, d'horreur ?
☐ 1 Oui
☐ 2 Non
☐ 97. Non concerné (ne pas suggérer)
☐ 98. Ne sait pas (ne pas suggérer)
☐ 99. Refus de réponse (ne pas suggérer)

G87. Quel âge aviez-vous quand cet évènement est arrivé?
ENQ : si plusieurs évènements, on évoque celui qui a le plus marqué l'interviewé
/__/__/ Ans

G88. A la suite de cet événement….

G88.1 - Y pensiez-vous de façon répétitive sans le vouloir ?
☐ 1 Oui
☐ 2 Non
☐ 97. Non concerné (ne pas suggérer)
☐ 98. Ne sait pas (ne pas suggérer)
☐ 99. Refus de réponse (ne pas suggérer)

G88.2 - Avez-vous eu des rêves ou des cauchemars répétitifs concernant cet événement ?
☐ 1 Oui
☐ 2 Non
☐ 97. Non concerné (ne pas suggérer)
☐ 98. Ne sait pas (ne pas suggérer)
☐ 99. Refus de réponse (ne pas suggérer)

G88.3 - Avez-vous eu des palpitations, des sueurs ou des malaises physiques lorsque quelqu'un vous rappelait l'événement ?
☐ 1 Oui
☐ 2 Non
☐ 97. Non concerné (ne pas suggérer)
☐ 98. Ne sait pas (ne pas suggérer)
☐ 99. Refus de réponse (ne pas suggérer)

G88.4 - Avez-vous eu plus de difficultés que d'habitude à vous endormir ou à rester endormi(e)?
☐ 1 Oui
☐ 2 Non
☐ 97. Non concerné (ne pas suggérer)
☐ 98. Ne sait pas (ne pas suggérer)
☐ 99. Refus de réponse (ne pas suggérer)

G88.5 - Etes-vous devenu plus irritable ?
☐ $_1$ Oui
☐ $_2$ Non
☐ $_{97}$. Non concerné (ne pas suggérer)
☐ $_{98}$. Ne sait pas (ne pas suggérer)
☐ $_{99}$. Refus de réponse (ne pas suggérer)

G88.6 - Eprouviez-vous plus de difficultés que d'habitude à vous concentrer sur ce que vous faisiez?
☐ $_1$ Oui
☐ $_2$ Non
☐ $_{97}$. Non concerné (ne pas suggérer)
☐ $_{98}$. Ne sait pas (ne pas suggérer)
☐ $_{99}$. Refus de réponse (ne pas suggérer)

G88.7 - Etes-vous devenu nerveux (se) ou sursautiez-vous facilement en réaction à des bruits ou des mouvements ordinaires ?
☐ $_1$ Oui
☐ $_2$ Non
☐ $_{97}$. Non concerné (ne pas suggérer)
☐ $_{98}$. Ne sait pas (ne pas suggérer)
☐ $_{99}$. Refus de réponse (ne pas suggérer)

G88.8 - Etiez vous constamment sur le qui vive ?
☐ $_1$ Oui
☐ $_2$ Non
☐ $_{97}$. Non concerné (ne pas suggérer)
☐ $_{98}$. Ne sait pas (ne pas suggérer)
☐ $_{99}$. Refus de réponse (ne pas suggérer)

G88.9 - Avez-vous eu des flash-backs c'est-à-dire que vous agissiez ou que vous vous sentiez comme si l'évènement était en train de se reproduire
☐ $_1$ Oui
☐ $_2$ Non
☐ $_{97}$. Non concerné (ne pas suggérer)
☐ $_{98}$. Ne sait pas (ne pas suggérer)
☐ $_{99}$. Refus de réponse (ne pas suggérer)

A la suite de cet événement….

G89.1 - Tentiez-vous d'éviter les endroits, les gens ou les situations susceptibles de vous rappeler cet événement ?
☐ $_1$ Oui
☐ $_2$ Non
☐ $_{97}$. Non concerné (ne pas suggérer)
☐ $_{98}$. Ne sait pas (ne pas suggérer)
☐ $_{99}$. Refus de réponse (ne pas suggérer)

G89.2 - Avez-vous fait des efforts pour ne pas y penser ou éviter les conversations s'y rapportant
☐ $_1$ Oui
☐ $_2$ Non
☐ $_{97}$. Non concerné (ne pas suggérer)
☐ $_{98}$. Ne sait pas (ne pas suggérer)
☐ $_{99}$. Refus de réponse (ne pas suggérer)

G89.3 - Avez-vous eu des trous de mémoire à propos de cet événement, c'est-à-dire, l'aviez-vous oublié en tout ou en partie ?
☐ $_1$ Oui → **poser G89.4**
☐ $_2$ Non → **poser G89.5**
☐ $_{97}$. Non concerné (ne pas suggérer)
☐ $_{98}$. Ne sait pas (ne pas suggérer)
☐ $_{99}$. Refus de réponse (ne pas suggérer)

Si Oui en G89.3, poser G89.4

G89.4 - Etait-ce parce que vous étiez blessé(e) ou aviez perdu connaissance lors de cet événement que vous aviez oublié tout ou une partie de celui-ci ?

☐ $_1$ Oui
☐ $_2$ Non
☐ $_{97}$. Non concerné (ne pas suggérer)
☐ $_{98}$. Ne sait pas (ne pas suggérer)
☐ $_{99}$. Refus de réponse (ne pas suggérer)

G89.5 - Avez-vous perdu intérêt pour des activités qui étaient importantes ou agréables pour vous auparavant ?
☐ $_1$ Oui
☐ $_2$ Non
☐ $_{97}$. Non concerné (ne pas suggérer)
☐ $_{98}$. Ne sait pas (ne pas suggérer)
☐ $_{99}$. Refus de réponse (ne pas suggérer)

G89.6 - Avez-vous commencé à vous sentir plus isolé (e) ou distant (e) des autres ?
☐ $_1$ Oui
☐ $_2$ Non
☐ $_{97}$. Non concerné (ne pas suggérer)
☐ $_{98}$. Ne sait pas (ne pas suggérer)
☐ $_{99}$. Refus de réponse (ne pas suggérer)

G89.7 - Trouviez-vous qu'il vous était plus difficile d'éprouver de la tendresse ou de l'affection pour les autres ?
- ☐ 1 Oui
- ☐ 2 Non
- ☐ 97. Non concerné (ne pas suggérer)
- ☐ 98. Ne sait pas (ne pas suggérer)
- ☐ 99. Refus de réponse (ne pas suggérer)

G89.8 - Aviez-vous commencé à sentir qu'il ne servait à rien de faire des projets pour l'avenir ?
- ☐ 1 Oui
- ☐ 2 Non
- ☐ 97. Non concerné (ne pas suggérer)
- ☐ 98. Ne sait pas (ne pas suggérer)
- ☐ 99. Refus de réponse (ne pas suggérer)

G89.9 - Avez-vous eu un autre problème que nous n'avons pas évoqué ?
- ☐ 1 Oui → **poser G90**
- ☐ 2 Non → **poser G91**
- ☐ 97. Non concerné (ne pas suggérer)
- ☐ 98. Ne sait pas (ne pas suggérer)
- ☐ 99. Refus de réponse (ne pas suggérer)

Si oui en G89.9, poser G90
G90. Quel est cet autre problème ?

Attention les questions de la G91 à la G99.3 nous parlons de l'événement principal traumatisant (cf G85bis)

G91. Combien de temps après cet événement avez-vous commencé à avoir l'un des problèmes ou l'une des réactions ou manifestations que nous venons d'évoquer ?
- ☐ 1 Moins d'un mois
- ☐ 2 Entre 1 mois et moins de 6 mois
- ☐ 3 Entre 6 mois et moins d'1 an
- ☐ 4 Entre 1 an et moins de 2 ans
- ☐ 5 Entre 2 ans et moins de 5 ans
- ☐ 6 5 ans ou plus
- ☐ 97. Non concerné (ne pas suggérer)
- ☐ 98. Ne sait pas (ne pas suggérer)
- ☐ 99. Refus de réponse (ne pas suggérer)

G92. Combien de temps ont duré ces réactions ?
- ☐ 1 Moins d'un mois
- ☐ 2 Entre 1 mois et moins de 6 mois
- ☐ 3 Entre 6 mois et moins d'1 an
- ☐ 4 Entre 1 an et moins de 2 ans
- ☐ 5 Entre 2 ans et moins de 5 ans
- ☐ 6 5 ans ou plus
- ☐ 97. Non concerné (ne pas suggérer)
- ☐ 98. Ne sait pas (ne pas suggérer)
- ☐ 99. Refus de réponse (ne pas suggérer)

G93. Au cours des douze derniers mois, avez-vous connu une période d'un mois ou plus où vous aviez certains des problèmes que nous venons d'évoquer à cause de ce que vous avez vécu ?
- ☐ 1 Oui
- ☐ 2 Non
- ☐ 97. Non concerné (ne pas suggérer)
- ☐ 98. Ne sait pas (ne pas suggérer)
- ☐ 99. Refus de réponse (ne pas suggérer)

G94. Sur une échelle de 0 à 10 où 0 signifie une absence de gêne et 10 signifie une gêne sévère, quel chiffre décrit le mieux la gêne occasionnée par vos réactions à l'évènement traumatisant que vous avez vécu dans chacune des activités suivantes au cours des 12 derniers mois?

94.1 - Vos tâches ménagères (le ménage, les courses ou s'occuper de la maison, de l'appartement /__/__/

94.2 - Votre capacité de travail /__/__/

94.3 - Votre capacité à établir et à maintenir des relations proches avec les autres /__/__/

94.4 - Votre vie sociale /__/__/

G95. Durant ces 12 derniers mois, pendant environ combien de jours avez-vous été totalement incapable de travailler ou d'effectuer vos activités normales à cause de vos réactions à cet évènement ?

/__/__/ Jours

SI G3=1ou2 OU SI G6= 1ou2 OU SI G11=1 Alors poser G96

G96. Avez-vous parlé à un médecin de ce ou ces problèmes?
- ☐ 1 Oui → **poser G97**
- ☐ 2 Non → **poser G98**
- ☐ 97. Non concerné (ne pas suggérer)
- ☐ 98. Ne sait pas (ne pas suggérer)
- ☐ 99. Refus de réponse (ne pas suggérer)

Si G96 = 1 poser G97
G97. S'agissait-il d'un médecin ?

G97.1 Généraliste
- ☐ 1 Oui
- ☐ 2 Non
- ☐ 97. Non concerné (ne pas suggérer)
- ☐ 98. Ne sait pas (ne pas suggérer)
- ☐ 99. Refus de réponse (ne pas suggérer)

G97.1bis du médecin qui vous suit pour le VIH ?

- ☐ $_1$ Oui
- ☐ $_2$ Non
- ☐ $_{97}$. Non concerné (ne pas suggérer)
- ☐ $_{98}$. Ne sait pas (ne pas suggérer)
- ☐ $_{99}$. Refus de réponse (ne pas suggérer)

G97.2 Psychiatre

- ☐ $_1$ Oui
- ☐ $_2$ Non
- ☐ $_{97}$. Non concerné (ne pas suggérer)
- ☐ $_{98}$. Ne sait pas (ne pas suggérer)
- ☐ $_{99}$. Refus de réponse (ne pas suggérer)

G97.3 D'un autre médecin

- ☐ $_1$ Oui
- ☐ $_2$ Non
- ☐ $_{97}$. Non concerné (ne pas suggérer)
- ☐ $_{98}$. Ne sait pas (ne pas suggérer)
- ☐ $_{99}$. Refus de réponse (ne pas suggérer)

G98. Et en avez-vous parlé à une autre personne ?

G98.1 – Un psychologue

- ☐ $_1$ Oui
- ☐ $_2$ Non
- ☐ $_{97}$. Non concerné (ne pas suggérer)
- ☐ $_{98}$. Ne sait pas (ne pas suggérer)
- ☐ $_{99}$. Refus de réponse (ne pas suggérer)

G98.2 – Un psychothérapeute qui n'était ni un médecin ni un psychologue

- ☐ $_1$ Oui
- ☐ $_2$ Non
- ☐ $_{97}$. Non concerné (ne pas suggérer)
- ☐ $_{98}$. Ne sait pas (ne pas suggérer)
- ☐ $_{99}$. Refus de réponse (ne pas suggérer)

G98.3 – Une assistante sociale ou une infirmière ou un autre intervenant

- ☐ $_1$ Oui
- ☐ $_2$ Non
- ☐ $_{97}$. Non concerné (ne pas suggérer)
- ☐ $_{98}$. Ne sait pas (ne pas suggérer)
- ☐ $_{99}$. Refus de réponse (ne pas suggérer)

G99. A cause de ce/ces problèmes, avez-vous pris plus d'une fois l'un ou plusieurs des produits suivants ?

G99.1 – Des médicaments ?

- ☐ $_1$ Oui
- ☐ $_2$ Non
- ☐ $_{97}$. Non concerné (ne pas suggérer)
- ☐ $_{98}$. Ne sait pas (ne pas suggérer)
- ☐ $_{99}$. Refus de réponse (ne pas suggérer)

G99.2 – De la drogue ?

- ☐ $_1$ Oui
- ☐ $_2$ Non
- ☐ $_{97}$. Non concerné (ne pas suggérer)
- ☐ $_{98}$. Ne sait pas (ne pas suggérer)
- ☐ $_{99}$. Refus de réponse (ne pas suggérer)

G99.3 – De l'alcool ?

- ☐ $_1$ Oui
- ☐ $_2$ Non
- ☐ $_{97}$. Non concerné (ne pas suggérer)
- ☐ $_{98}$. Ne sait pas (ne pas suggérer)
- ☐ $_{99}$. Refus de réponse (ne pas suggérer)

Tous

G100. Au cours de votre vie, avez-vous déjà pensé au suicide ?

- ☐ $_1$ Oui **➔ Posez G101**
- ☐ $_2$ Non **➔FIN du module**
- ☐ $_{97}$. Non concerné (ne pas suggérer)
- ☐ $_{98}$. Ne sait pas (ne pas suggérer)
- ☐ $_{99}$. Refus de réponse (ne pas suggérer)

G101. Y avez-vous pensé au cours des 12 derniers mois ?

- ☐ $_1$ Oui
- ☐ $_2$ Non
- ☐ $_{97}$. Non concerné (ne pas suggérer)
- ☐ $_{98}$. Ne sait pas (ne pas suggérer)
- ☐ $_{99}$. Refus de réponse (ne pas suggérer)

G102. <u>Au cours de votre vie</u>, vous est-il arrivé de faire des projets de suicide ?
- ☐ 1 Oui → **Poser G103**
- ☐ 2 Non → **Poser G105**
- ☐ 97. Non concerné (ne pas suggérer)
- ☐ 98. Ne sait pas (ne pas suggérer)
- ☐ 99. Refus de réponse (ne pas suggérer)

G103. Cela vous est-il arrivé au cours des <u>12 derniers mois</u> ?
- ☐ 1 Oui
- ☐ 2 Non
- ☐ 97. Non concerné (ne pas suggérer)
- ☐ 98. Ne sait pas (ne pas suggérer)
- ☐ 99. Refus de réponse (ne pas suggérer)

G104. La première fois ou vous avez fait des projets de suicide, était est ce avant ou après la découverte de votre séropositivité ?
- ☐ 1 Avant la découverte
- ☐ 2 Après la découverte
- ☐ 97. Non concerné (ne pas suggérer)
- ☐ 98. Ne sait pas (ne pas suggérer)
- ☐ 99. Refus de réponse (ne pas suggérer)

G105. Avez-vous déjà fait une tentative de suicide ?
- ☐ 1 Oui → **Poser G106**
- ☐ 2Non → **FIN du module**
- ☐ 97. Non concerné (ne pas suggérer)
- ☐ 98. Ne sait pas (ne pas suggérer)
- ☐ 99. Refus de réponse (ne pas suggérer)

G106. Cela vous est-il arrivé plusieurs fois ?
- ☐ 1 Oui
- ☐ 2 Non
- ☐ 97. Non concerné (ne pas suggérer)
- ☐ 98. Ne sait pas (ne pas suggérer)
- ☐ 99. Refus de réponse (ne pas suggérer)

G107. La première fois où vous avez fait une tentative de suicide, était est ce avant ou après la découverte de votre séropositivité ?
- ☐ 1 Avant la découverte
- ☐ 2 Après la découverte
- ☐ 97. Non concerné (ne pas suggérer)
- ☐ 98. Ne sait pas (ne pas suggérer)
- ☐ 99. Refus de réponse (ne pas suggérer)

G108. Cela vous est-il arrivé au cours <u>des 12 derniers mois</u> ?
- ☐ 1 Oui
- ☐ 2 Non
- ☐ 97. Non concerné (ne pas suggérer)
- ☐ 98. Ne sait pas (ne pas suggérer)
- ☐ 99. Refus de réponse (ne pas suggérer)

MODULE H : RELATIONS AVEC LES ASSOCIATIONS DE LUTTE CONTRE LE SIDA

Pour finir, nous allons parler des associations de lutte contre le sida.

H.1. **<u>Au cours des 12 derniers mois</u>, avez-vous donné de l'argent pour la lutte contre le sida ?**

☐ 1 Oui
☐ 2 Non
☐ 97. Non concerné (ne pas suggérer)
☐ 98. Ne sait pas (ne pas suggérer)
☐ 99. Refus de réponse (ne pas suggérer)

H.2. **<u>Au cours des 12 derniers mois</u>, avez-vous participé aux activités d'associations de lutte contre le sida (groupe de paroles, soirées, ateliers…) ?**

☐ 1 Oui
☐ 2 Non
☐ 97. Non concerné (ne pas suggérer)
☐ 98. Ne sait pas (ne pas suggérer)
☐ 99. Refus de réponse (ne pas suggérer)

H.3. **<u>Au cours des 12 derniers mois</u>, avez-vous été volontaire ou militant(e) d'associations de lutte contre le sida ?**

☐ 1 Oui
☐ 2 Non
☐ 97. Non concerné (ne pas suggérer)
☐ 98. Ne sait pas (ne pas suggérer)
☐ 99. Refus de réponse (ne pas suggérer)

H.4. **<u>Au cours des 12 derniers mois</u>, avez-vous été un(e) permanent(e) ou un(e) salarié(e) d'associations de lutte contre le sida ?**

☐ 1 Oui
☐ 2 Non
☐ 97. Non concerné (ne pas suggérer)
☐ 98. Ne sait pas (ne pas suggérer)
☐ 99. Refus de réponse (ne pas suggérer)

Je vous remercie d'avoir répondu à nos questions. D'ici quelques mois les résultats de cette étude seront disponibles dans les journaux associatifs et sous forme de brochures dans les services de soins.

☐ **Remarques enquêteur/trice sur la conduite du questionnaire :**

……
……
……
……
……
……
……
……………………………………………………………………………
